# Indigenous Foods of India: A Comprehensive Narrative Review of Nutritive Values, Antinutrient Content and Mineral Bioavailability of Traditional Foods Consumed by Indigenous Communities of India

**DOI:** 10.3389/fsufs.2022.696228

**Published:** 2022-04-28

**Authors:** Ridhima Kapoor, Manisha Sabharwal, Suparna Ghosh-Jerath

**Affiliations:** 1 Indian Institute of Public Health-Delhi, Public Health Foundation of India, Gurgaon, India; 2 Department of Food and Nutrition, Lady Irwin College, University of Delhi, New Delhi, India

**Keywords:** indigenous food, nutrient composition, antinutrient components, mineral bioavailability, Indian tribes, molar ratio, traditional foods

## Abstract

India is endowed with several indigenous foods (IFs), that hold special cultural significance among local and ethnic caommunities, yet no attempts have been made till date to systematically compile their nutritive values. As per FAO’s recent mandate on creation of “*Global-Hub on Indigenous Food Systems,”* IFs have received renewed global recognition for their potential to contribute to improved food security while enhancing biodiversity across the world. Hence, the useful properties of wild IFs require proper study and documentation in order to bridge the gap between scientific evidence generation and indigenous peoples’ ancestral knowledge. For this purpose, we conducted a literature search in two scientific databases: PubMed and Google Scholar, between July 2020 and December 2021, to identify studies reporting nutritive values and/or antinutrient content of IFs (not included in Indian food composition database), consumed by Indian indigenous communities. A total of 52 Indian research articles were included, from which data was selected and extracted, to create a compendium on nutrient (*n* = 508) and antinutrient (*n* = 123) content of IFs, followed by computation of antinutrient-to-mineral molar ratios for 98 IFs to predict their mineral bioavailability. Maximum nutritive values were available for green leafy vegetables (*n* = 154), followed by other vegetables (*n* = 98), fruits (*n* = 66), cereals (*n* = 63), roots & tubers (*n* = 51) and nuts and legumes (*n* = 36). Several IFs seen to have better nutritional content than conventional foods and were found to be rich (i.e., >20% Indian recommended dietary allowances per reference food serve) in iron (54%), calcium (35%), protein (30%), vitamin C (27%), vitamin A (18%), zinc (14%) and folate (13%). Some IFs displayed high levels of antinutrients, however, anti-nutrient-to-mineral molar ratios were found to be low (for mainly leafy vegetables, other vegetables, and roots and tubers), thus indicating high mineral bioavailability. Hence, efforts are desirable to encourage the inclusion of these nutritionally superior IFs into the usual diets of indigenous communities. The IF database collated in our review can serve as a resource for researchers and policymakers to better understand the nutritional properties of region-specific IFs and promote them through contextual food-based interventions for improved dietary quality and nutrition outcomes in indigenous population of India.

## Introduction

Traditional food systems, which include all the foods available from local natural resources, are unprocessed, small-scale and low-technology human managed biophysical systems, with short farm-to-plate value chains (Kuhnlein et al., 2006; Burlingame et al., 2012; Dubé et al., 2014). These food systems consist of harvesting, foraging, hunting, fishing and gathering of plant and animal foods, and are often shaped by diverse eating practices, ecological features, geographical variations and socio-cultural as well as historical experiences (Kuhnlein et al., 2013). Indigenous peoples who form distinct social and cultural groups, and have strong links with their territories and surrounding natural resources, are often the sole custodians of traditional food systems and the ancestral ecological knowledge associated with them (Kuhnlein, 2009). The food systems of indigenous people have sustained them for thousands of generations, generating food in harmony while preserving the local biodiversity. Indigenous foods or IFs, an integral part of traditional food systems, are central to indigenous people’s culture and identity and contribute to their physical, mental, spiritual and economic wellbeing. These foods, include wild, domesticated or cultivated plant, animal and/or fungi species, that are derived from surrounding natural environment, and act as a crucial source of nourishment, and material subsistence for people belonging to the indigenous communities (Kuhnlein et al., 2013; Settee and Shukla, 2020).

During the past decade, IFs have received renowned global recognition for their potential to contribute to improved food security while enhancing biodiversity across the world. They are known to be of high nutritive value and their potential contribution to improved health and nutrition is significant (Kuhnlein et al., 2019). Research in Sub-Saharan African and South American countries have reported better intake of protein, fiber, vitamin A, iron, calcium and riboflavin among people consuming IFs (Akinola et al., 2020; Lugo-Morin, 2020). Further, ample evidence from the literature suggests that indigenous crops have a critical role in addressing malnutrition and hunger (Shackleton et al., 1998; Steyn et al., 2001; Yiridoe and Anchirinah, 2005; Modi et al., 2006) and may contribute to more diverse diets at both household and individual levels (Trichopoulou et al., 2006; Frison et al., 2011; Powell et al., 2011; Burlingame et al., 2012). Realizing the extreme potential of IFs in contributing to optimal health and nutrition, Food and Agriculture Organization (FAO) in 2018, collaborated with other member organizations, to create a “*Global-Hub on Indigenous Food Systems,*” which aims to preserve and promote the indigenous peoples’ food systems, through creation of knowledge bearer’s platform, online database, technical advice and creation of synergies. This platform further proposes to bring together universities, research centers, indigenous organizations and FAO Members around a common platform for exchange of evidence-based knowledge to inform better nutrition and sustainable practices and policies worldwide (FAO, 2018).

India, a country of 1.2 billion people, is home to 705 indigenous communities (Census, 2011), who have their distinct food systems with several IFs adding to dietary diversity (Misra et al., 2008; Ghosh-Jerath et al., 2020a). These communities are locally recognized as scheduled tribes and live in environments characterized by defined regions with specific food habits, dialects, cultural homogeneity, and a unified social organization (Ministry of Tribal Affairs, Government of India, 2020). Many of these indigenous communities often reside in areas surrounded by lush green and dense forests, hills and rivers, and are hence exposed to a rich biodiversity, which they manage and utilize for food, livelihood and income generation (MoHF and Ministry of Tribal Affairs, 2019). They produce and collect local IFs from several food sources in the form of edible greens (leaves, stems, shoots, including marine algae); root vegetables (including true roots and underground storage organs like bulbs, corms, tubers and rhizomes); fleshy fruits (berries, pomes, drupes); edible wild mushrooms, grains, seeds, and nuts, as well as wild-harvested fish, insects and game (Agrahar-Murugkar and Subbulakshmi, 2005; Chyne et al., 2019; Ghosh-Jerath et al., 2020a, 2021). Other Indian indigenous communities residing in deserts and barren areas also skillfully manage their natural resources, and utilize a wide range of edible herbs, shrubs and trees that are well-adapted to some of the most extreme weather conditions of the hot desert (Mukhopadhyay, 2008; Bhansali, 2011). Many of the IFs accessed by indigenous communities, are sourced from the natural environment, i.e., wild (forests, disturbed habitats, open pastures, ponds and rivers) and cultivated food environment (fields, orchards and backyard gardens), are deemed to be nutritious, palatable and easily harvested, and are often adapted to unique climatic and environmental conditions (Agrahar-Murugkar and Subbulakshmi, 2005; Ghosh-Jerath et al., 2020a, 2021).

However, despite having access to rich dietary diversity from these sources, indigenous communities in India consume poor quality diets and display high levels of malnutrition. Almost one-third of the indigenous women in India are underweight while 4.7 million indigenous children under 5 years of age suffer from chronic nutrition deprivation, with 43% children stunted, 27% wasted and 45.3% underweight (NFHS-4, 2015). More than half the women and children are anemic, and about 40% children are folate deficient while almost one-fifth children suffer from vitamin A, zinc and vitamin B12 deficiencies (NFHS-4, 2015). Furthermore, with urbanization and ongoing nutrition transition, the agricultural and natural resource management practices of indigenous communities, i.e., their traditional ecological knowledge (TEK) is becoming lost, a trend that may be responsible for reduced IF consumption and poorer nutrition (Kuhnlein et al., 2004; Ghosh-Jerath et al., 2021). Hence, there exists an urgent need to document this TEK, explore the nutrient potential of community specific IFs and promote their consumption which can potentially alleviate malnutrition and hunger in these vulnerable indigenous communities.

Although many studies have listed several IFs of indigenous communities in India, but to date no attempts have been made to systematically compile the nutritive values of these foods. The useful properties of wild IFs requires proper study and documentation in order to bridge the gap between scientific evidence generation and indigenous peoples’ ancestral knowledge (Alexander et al., 2019). Moreover, there is limited information available on anti-nutrient content of IFs, which may have an impact on the nutrient bioavailability of these foods. Therefore, the objective of this review is to compile the available information on different IFs of indigenous communities across different states in India, and create a compendium with specific information on their (a) macro- and micro-nutrient contents (b) content of anti-nutritional components and (c) mineral bioavailability.

## Methods

### Literature Search

**Figure 1:**
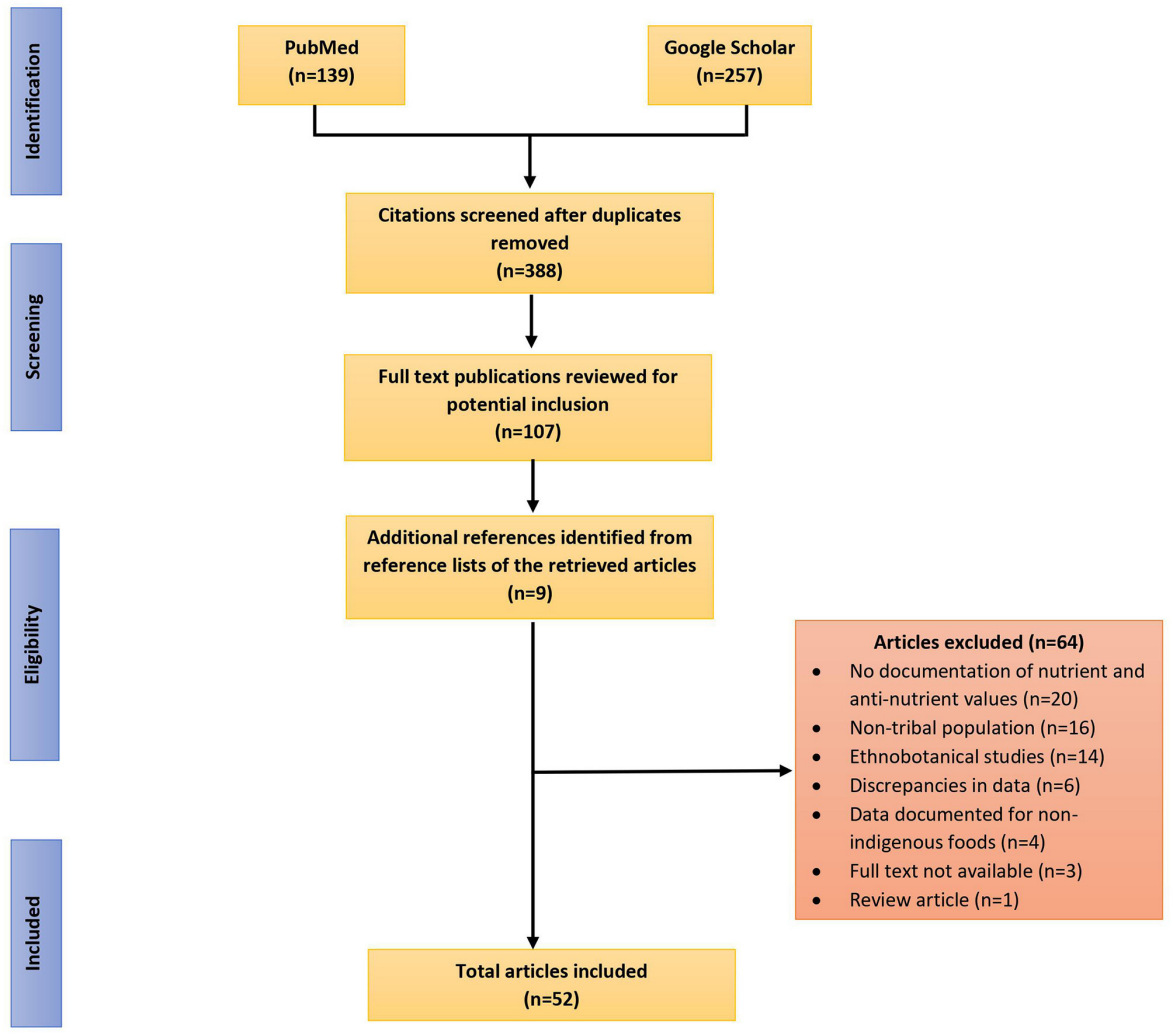
Flow diagram reporting the screening and selection process used in identification of research studies reporting nutritive values and antinutritional content of indigenous foods consumed by and known to indigenous communities of India.

The data sources for this review included published research articles and reports that were selected through two phases. In the first phase, a literature search was conducted between July, 2020 and February 2021 in well-known databases such as PubMed and Google Scholar. Searches were performed using various combinations of keywords like “nutritive value,” “nutrition,” “anti-nutritional factors,” “indigenous foods,” “traditional foods,” “wild foods,” “tribal communities,” and “India” to identify relevant studies. Articles were scanned and checked against the inclusion criteria, duplicate citations were removed and those that met the inclusion criteria were read and assessed for inclusion. In the second phase, by utilizing snowballing technique, additional relevant studies were identified from the reference lists of all included papers identified in the initial search. Figure 1 presents the step-wise methodological approach followed in selection of articles for the review.

### Selection Criteria

In the present review, we have used the term “Indigenous foods or IFs” to denote foods that are native or have been introduced in the region a long time back, and are accessed from the natural food environment (i.e., either wild or cultivated food environment). Considering the globalization of current food systems, these foods may also be purchased from informal built food environment, i.e., local markets or small shops (Kuhnlein, 2009; Kasimba et al., 2019; Settee and Shukla, 2020). Based on this definition, we selected articles following these eligibility criteria: (i) original full-text articles in English, published till 1^st^ February, 2021, (ii) research studies conducted in Indian states and/or Union Territories (UT), and (iii) research studies reporting nutritive values and/or anti-nutrient content of IFs consumed and known to indigenous communities [scheduled tribes as per Article 342 of Indian constitution (Ministry of Tribal Affairs, Government of India)]. We defined nutrient composition data as inclusive of macronutrients (energy, protein, fat) and/or micronutrients (vitamins A, B1, B2, B3, B6, B9, C, iron, calcium, and zinc) while, anti-nutrient data was defined as inclusive of different anti-nutritional factors (tannins, saponins, phytates, oxalates, total free phenols, trypsin inhibitor, amylase inhibitor and/or hydrogen cyanide).

### Data Extraction and Preparation

After reviewing different scientific sources based on the selection criteria, a final inventory of 52 articles were prepared. The data and units were extracted from each study for all macronutrients, micronutrients and anti-nutrients, critically reviewed and arranged systematically according to the food groups. Indian food composition table (IFCT) prepared by National Institute of Nutrition (Longvah et al., 2017) was also referred to document additional nutrient and anti-nutrient composition data (if available) on IFs that were mentioned in the included research articles. All information on IFs such as their scientific names, vernacular as well as common names were gathered and compiled. The scientific names of the foods were verified from The Plant List (2020). The foods with nutrient content (protein and micronutrients) higher than 20% of Recommended dietary allowances (RDA) for moderately active Indian adult (per reference amount) (ICMR-NIN, 2020) were categorized as “rich” sources of nutrients (United States Food and Drug Administration, 2019). The nutrient and anti-nutrient data from included research studies were further utilized to compute the anti-nutrient-to-mineral molar ratio indices. For this purpose, molar ratio indices of phytate-to-minerals and oxalate-to-minerals [i.e., phytate/calcium (Phy/Ca), phytate/iron (Phy/Fe), phytate/zinc (Phy/Zn), phytate x calcium/zinc (Phy x Ca/Zn), oxalate/calcium (Ox/Ca)] were obtained after dividing the mole of phytate and oxalate with the mole of minerals (Morris and Ellis, 1989; Norhaizan and Nor Faizadatul Ain, 2009). Standard critical values of molar ratio indices defined by existing literature were used [i.e., > 0.24 for Phy/Ca (Morris and Ellis, 1985), >1 for Phy/Fe (Hallberg et al., 1989), >15 for Phy/Zn (Turnlund et al., 1984; Sandberg et al., 1987; Morris and Ellis, 1989), >200 for Phy x Ca/Zn (Mills et al., 1985; Bindra et al., 1986) and > 2.5 for Ox/Ca (Mills et al., 1985; Bindra et al., 1986)] to predict low mineral bioavailability in IFs.

## Results

### Description of Studies

Out of 52 studies included in the review, majority of the studies were carried out in North-east India (*n* = 25), followed by southern (*n* = 11), eastern (*n* = 10), western (*n* = 3), central (*n* = 2) and northern (*n* = 1) regions of the country. Estimation of macronutrients and/or micronutrients was reported in all the studies while only 11 studies reported the estimation of anti-nutritional factors (ANFs) in IFs. Out of 52 studies, 48 studies reported the methodology of food sample collection; the IFs were mostly collected from areas of natural vegetation (such as farms, forests, near river banks etc.), were cleaned/washed to remove dirt and packed for further analysis. The methodology of taxonomic identification was available in 40 studies; food samples were either prepared as herbariums and sent to botanical/zoological laboratories for identification or identified by a taxonomist at the field sites and/or with help of photographs. All the studies followed standard analytical procedures for determination of proximate compositions, micronutrients, as well as anti-nutrient components (details in Supplementary Table 1).

### Nutritive Values of Indigenous Foods of India

In the present review, we have compiled the nutritive values of 508 IFs, consumed by indigenous communities across 15 states and 1 UT of India. Majority of IFs with their nutritive values estimated, were documented from Jharkhand (28%), followed by Meghalaya (15%) and Kerala (11%). These IFs have been classified into the following categories: (a) 63 cereals (b) 36 nuts and legumes, (c) 154 green leafy vegetables (GLVs), (d) 98 other vegetables (including mushrooms), (e) 51 roots & tubers, (f) 66 fruits, (g) 40 flesh foods (including livestock and wild game). Supplementary Data Sheet 1 presents the common names of these IFs, with additional information on their local and scientific names, edible part(s) consumed, indigenous communities accessing the foods and their state of residence. The nutritive values of IFs have been compiled food group wise in Supplementary Table 2. Upon review, several IFs are seen to be rich in iron (54%) and calcium (35%), and nearly one-third of them are protein-rich (30%). Some IFs are also rich in other micronutrients like vitamin C (27%), vitamin A (18%), zinc (14%) and folate (13%). Nutritive values of some nutrient rich IFs are provided in Table 1. Despite their rich nutritional content, about one-third of the studies (Gupta et al., 2005; Vadivel and Janardhanan, 2005; Agrahar-Murugkar, 2006; Arinathan et al., 2009; Shajeela et al., 2011; Jain and Tiwari, 2012; Das et al., 2015; Ghosh-Jerath et al., 2015, 2016, 2020a, 2021; Horo and Topno, 2015; Pradeepkumar et al., 2015; Terangpi and Teron, 2015; Singh et al., 2018; Chyne et al., 2019; Longvah et al., 2020) (33%) have reported under-utilization of most IFs in the indigenous communities.

**Figure 2:**
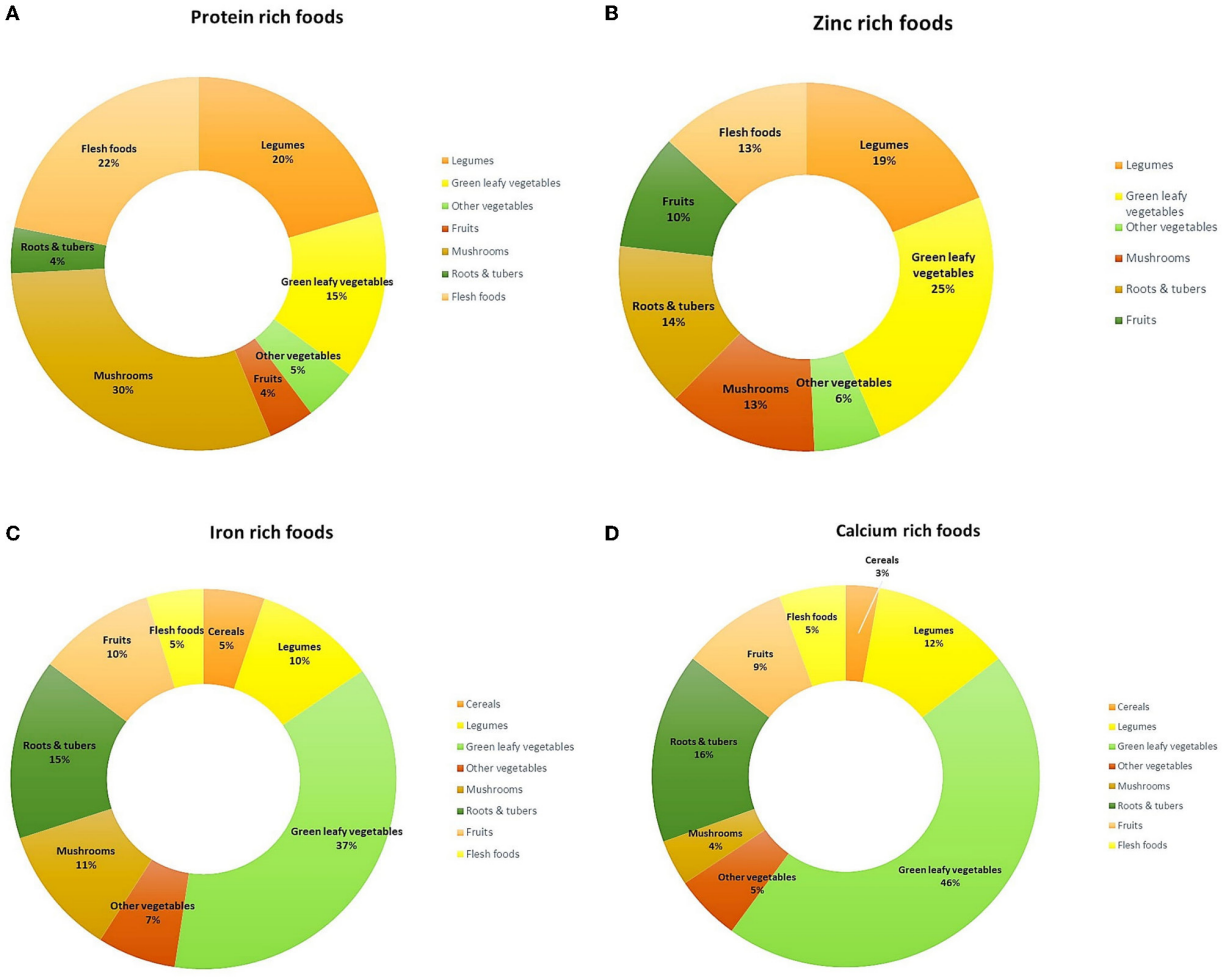

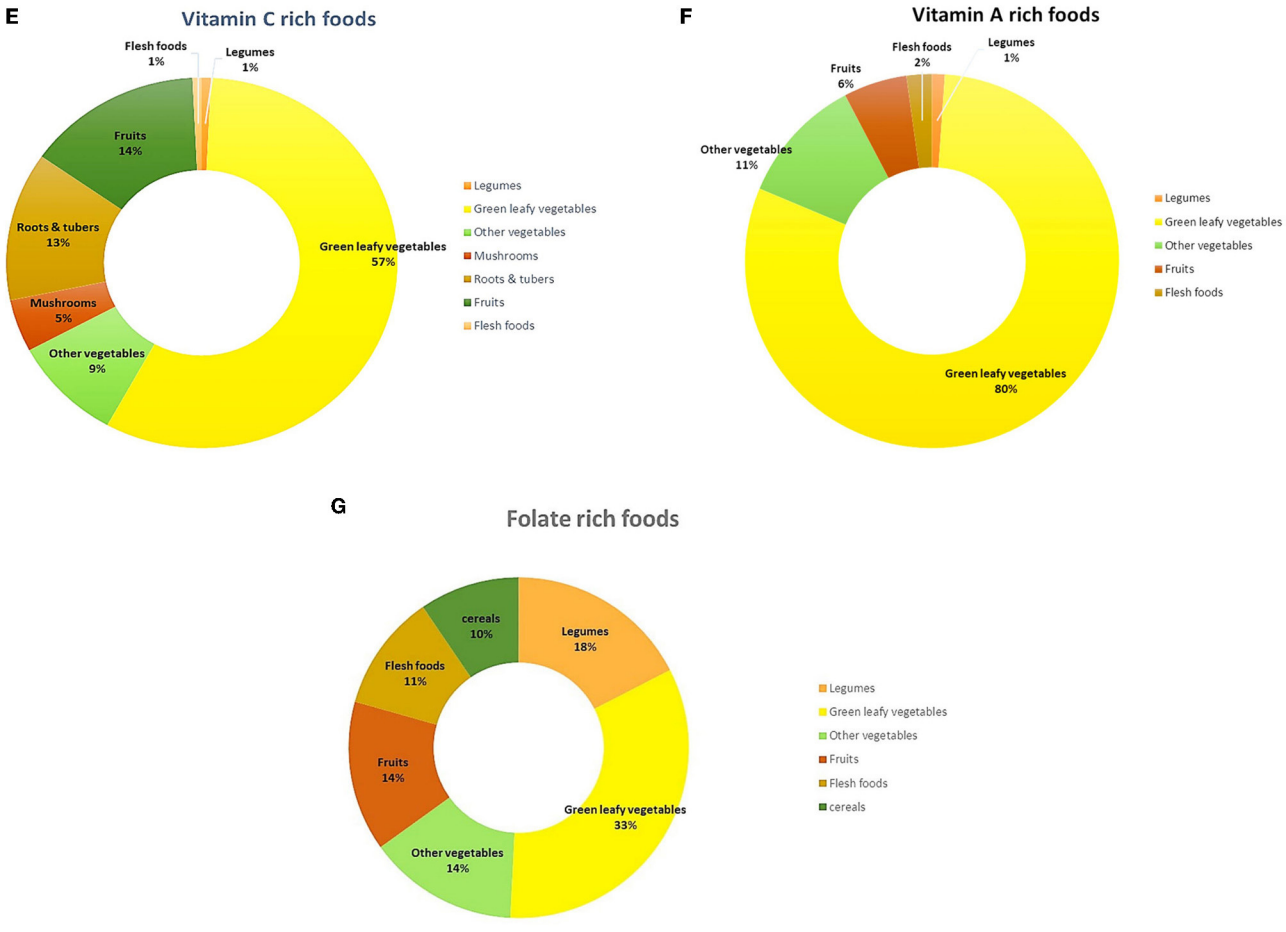
**(A–G)** Distribution of protein and micronutrient-rich indigenous foods in each food group.

Several micronutrient-rich foods belong to the GLV (28%) food group, followed by other vegetables (13%) and wild roots & tubers (8%) (Figure 2). Nonetheless, other food groups have also been found to contain rich amounts of several nutrients. Among cereals, nutritive values of 49 local rice landraces have been documented, which are cultivated and consumed by Santhals, Sauria Paharias, Oraons and Mundas (Jharkhand), Savera, Jatapu, Gadabe and Kondadora (Andhra Pradesh), Garo and Khasi (Meghalaya) and indigenous communities of Himachal Pradesh. A high iron (3.1–6.1 mg/100 g) and folate content (65–1,203 μg/100 g) was observed in some varieties of red rice (*Kba-baswoit, Ladhan, Kba-bakut, Kba-Iwai, Kba-stem)* and sticky rice (*Kba-shulia)*, that are consumed in North-east India. and folate. Many millet varieties also showed high content of iron (3.9–19.3 mg/100 g) and calcium (264–364 mg/100 g). These include, Indian goosegrass (*Eleusine indica*), Japanese millet *(Echinochloa frumentacea)*, *Salharkutki* [scientific name not available (NA)], Italian millet/*Kangni* (*Setaria italica*), Little millet (*Panicum antidotale*) and indigenous varieties of Finger millet (*Eleusine coracana*).

In case of indigenous legumes, the usual protein content varies in the range of 18.3–35 g/100 g, with the highest content in Jack bean (*Canavalia ensiformis*), a wild legume consumed by indigenous communities of Kerala. Several legumes reported from Jharkhand, Kerala and Andhra Pradesh have also been found to be rich sources of calcium (260–945.4 mg/100 g), iron (3.6–38.6 mg/100 g) and zinc (3.4–7.7 mg/100 g), respectively. Among indigenous nuts, exceptionally high levels of calcium (1,020–1,540 mg/100 g) are reported in varieties consumed by Khasi tribe of Meghalaya, with the highest content in Indian Chestnut (*Castanopsis indica*). Perilla (*Perilla frutescens*), an important oilseed of Meghalaya and Manipur, has been found to have high levels of iron (8.3–9 mg/100 g) and zinc (4.7–5.02 mg/100 g).

Majority of the indigenous GLVs are found to be rich sources of iron (*n* = 101), calcium (*n* = 82), vitamin C (*n* = 75), vitamin A (*n* = 73), folate (*n* = 21) and zinc (*n* = 17). Most of these micronutrient rich leafy vegetables are reported from north-eastern part of India, in states of Assam, Manipur, Arunachal Pradesh and Meghalaya. Many common GLVs consumed by different indigenous communities across the country, have also high content of several essential micronutrients. For instance, Basella leaves (*Basella alba*), an indigenous GLV consumed by Lodha tribe of West Bengal and Nicobari tribe of Andaman & Nicobar Islands, is found to be rich in iron (4.2–9.4 mg/100 g), vitamin A (2,473 μg/100 g) and vitamin C (63.4–297 mg/100 g). Gogu leaves *(Hibiscus sabdariffa)*, a common leafy vegetable consumed by indigenous communnities of Maharashtra, Jharkhand, Meghalaya and Andaman and Nicobar Islands, is an excellent source of zinc (15.8 mg/100 g). Similarly, Drumstick leaves (*Moringa oleifera*), a popular leafy vegetable across various indigenous communities, is a rich source of calcium (range: 314–440 mg/100 g), vitamin A (595–17,542 μg/100 g) and vitamin C (range: 991–30 mg/100 g), respectively. Different varieties of Amaranth leaves such as red Amaranth leaves (*Amaranthus gangeticus*), green leaves of Amaranth spinosus (*Amaranthus spinosus*), Chinese spinach (*Amaranthus tricolor*) and Purple Amaranth (*Amaranthus lividus*) which are consumed by the indigenous communities of Jharkhand, Andaman and Nicobar Islands, and Kerala, are specifically found to be rich sources of vitamin C (range: 77.3–308 mg/100 g).

Many varieties of other vegetables like Kattian/Kasai (*Bridelia retusa*), Hairy-fruited eggplant (*Solanum lasiocarpum*), Thai eggplant (*Solanum virginianum*), and indigenous Bittergourd (*Momordica charantia*) are found to be rich sources of vitamin C (range 277–826.4 mg/100 g), which are consumed by indigenous communities in Meghalaya and West Bengal, while high folate levels (106–413 μg/100 g) are reported for indigenous vegetables like Bitter tomato (*Solanum aethiopicum*), Tree tomato (*Cyphomandra betacea*), Wild bean (*Canavalia cathartica*), Broad bean (*Vicia faba)*, *Fejia* (*Wendlandia glabrata*) and Wild banana stem (*Musa acuminate*), that are consumed by indigenous communities of Manipur. Flowers of many wild plants, particularly flowers of wild plantain (*Ensete Superbum*) and Dhawal *(Woodfordia fruticose)*, consumed by indigenous communities of Maharashtra, are rich in both iron (range 55.1–518 mg/100 g) and calcium (range 219.4–665.5 mg/100 g), respectively. Apart from wild vegetables, indigenous communities of India also consume a wide variety of mushrooms, usually during the monsoon period. In our review, we have compiled nutritive values of 50 indigenous mushrooms of India, most of which (*n* = 46) are rich sources of protein (range: 15.3–39.1 g/100 g), with the maximum content in *Vellathazan Kumizh (Pleurotus sajor caju),* a wild edible mushroom consumed by Kaani community of Tamil Nadu. Most of the iron rich mushrooms (range 14.4–47.6 mg/100 g) are reported from West Bengal where they are consumed by Santhal and Lodha communities while, all mushroom varieties documented for Khasi communnity of Meghalaya are rich in calcium (range 420– 1,910 mg/100 g), zinc (6.8–39.4 mg/100 g) and vitamin C (range 14.9–41.9 mg/100 g), respectively.

Several indigenous roots and tubers documented in our review display high levels of micronutrients; about 29 varieties of indigenous roots and tubers are rich sources of calcium (range 218.1–784.2 mg/100 g), 34 are rich in iron (range: 4.7–124.3 mg/100 g) and 17 varieties are rich in vitamin C (range: 18.1– 250 mg/100 g). Most of these tubers (*n* = 13) belong to *Dioscorea* species, which are consumed across different regions in the country, including states of Tamil Nadu, Kerala, Andhra Pradesh, Jharkhand, West Bengal and Andaman and Nicobar Islands. High levels of niacin (19.9–88.4mg/100 g) are reported in roots & tubers consumed by Palliyar communtiy of Tamil Nadu, with the highest content in *Nurai* (*Dioscorea tomentosa*). Some of the wild root vegetables known to the indigenous communities of Tamil Nadu (Palliyars, Kanikkars, and Valiyans), West Bengal (Lodhas) and Kerala (Saveras, Jatapus, Gadabes and Kondadoras) are found to have rich amounts of macro-as well as micronutrients. These include, Indian yam (*Dioscorea oppositifolia*), *Mou Alu* (*Dioscorea wallichii*) and *Kedrostis foetidissima*, which are rich in protein (range 10.8–13.8 g/100 g), calcium(230–748.3mg/100 g), and iron (20.1–40.1mg/100 g), respectively. Among indigenous fruits, many varieties are rich in iron (*n*=26), vitamin C (*n*=19) and calcium (*n*=16). Several indigenous fruits consumed by Lodha community of West Bengal are reported to have rich amounts of vitamin C (96–600 mg/100 g), while many seasonal wild fruits consumed by Khasi communnity of Meghalaya, are rich in calcium (range 206–1,176 mg/100 g). Few indigenous fruits of Arunachal Pradesh, such as *Machilus robusta*, *Ocotea lancifolia* and Autumn olive (*Elaeagnus umbellate*), which are consumed by indigenous communities like Aka, Bugun, Miji, Monpa, Sherdukpen, Memba and Khamba, have been found to contain high protein content in the range of 12.7–15.2 g/100 g.

All the indigenous flesh foods (meat, poultry and fish) are found to be protein-rich (range-13.1–68.8 g/100 g). High calcium levels (370–690 mg/100 g) are observed in several small local fishes, consumed by indigenous communities of Jharkhand and Manipur. Entomophagy or insect consumption is reported among indigenous communities of North-east India (Manipur, Assam and Arunachal Pradesh) (Chakravorty et al., 2011, 2014; Ganguly et al., 2013). These insects are extremely nutritious, and are found to be rich in protein (range 10.6–65.7 g/100 g), iron (range 7.3–461 mg/100 g) and zinc (range 5.8–29.5 mg/100 g). Grasshopper *(Chondacris rosea)*, in particular, has exceptionally high amount of protein (69.8 g/100 g), calcium (340 mg/100 g), iron (7.8 mg/100 g) and zinc (10.8 mg/100 g).

### Anti-nutritional Content in Indigenous Foods of India

ANFs have been documented for 123 IFs (7 cereals, 17 legumes, 47 GLVs, 13 other vegetables, 30 roots and tubers, three fruits and six flesh foods) (Supplementary Table 3). Among cereals, most of the millets have high levels of phytate, with the highest content in Sorghum (549 mg/100g). Total oxalate levels in cereals are mostly low, with particularly low content observed in Rice (1.9 mg/100 g), Kodo millet (3.5 mg/100 g) and Little millet (6.7 mg/100 g). In case of legumes, all the varieties with documented ANFs have phytate content >300mg, with the highest in field bean varieties (791–799 mg/100 g). Indigenous varieities of Horse gram has the highest total oxalate level (181 mg/100 g) while, Red gram and Field beans (black, white and red) have markedly lower levels (1.2–1.4 mg/100 g). Least trypsin inhibitor activity is found in Pot Cassia (*Senna obtusifolia*) (13.5 TIU/mg protein) while highest is observed in *Mucuna monosperma* (65.4 TIU/mg protein).

Among GLVs, Ponnaginni (*Alternanthera sessilis*) and Agathi leaves (*Sesbania grandiflora*) have the highest saponin levels (880 mg/100 g) while Malabar spinach (*Basella rubra*) has the highest phytate content (287 mg/100 g). Although, there are about 34 GLVs with low phytate levels (<50 mg/100 g). such as, Colocasia leaves (*Colocasia esculenta*) (1–18 mg/100 g), Chinese spinach (2–18 mg/100 g), Beng leaves (*Centella asiatica*) (2.1–17 mg/100 g), Kena leaves (*Commelina benghalensis*) (2.4 mg/100 g), Lesua leaves (*Digera muricata*) (2.5 mg/100 g), Balae leaves (*Polygala erioptera*) (3.4 mg/100 g) and Red amaranth leaves (4.9 mg/100 g). Total oxalates are low in most GLVs, with few exceptions such as Lesua (1,410 mg/100 g), Chinese spinach (1,270 mg/100 g), Gadhakand leaves (*Boerhavia diffusa*) (1,250 mg/100 g) and Black pigweed leaves (*Trianthema portulacastrum*) (1,080 mg/100 g). Tannins ranged between 15 and 1,330 mg/100 g, with highest content in Yellow Gulmohar leaves (*Delonix elata*) and lowest content in Mexican mint leaves (*Plectranthus amboinicus*). Among vegetables, relatively lower phytate levels are reported, with the highest content of 68.8 mg/100 g in Jackfruit. Saponin levels are documented for only 5 vegetable varieties, with the highest content of 237 mg/100 g in Plantain flowers (*Musa* × *paradisiaca)*. Indian shot (*Canna indica*), in particular, has high content of anti-nutrients, with total oxalate levels of 940 mg/100 g, tannin content of 1,830 mg/100 g and total free phenols of 1,470 mg/100 g. On the other hand, vegetables like Ash gourd (*Benincasa hispida*), Kovai (*Coccinia grandis*), bamboo stems and Field beans/ (*Lablab purpureus*) have reported low content of anti-nutrients.

About 10 indigenous roots and tubers are found to contain high levels of total oxalates (>400mg), with maximum levels observed in *Parthenocissus neilgherriensis* (2,750 mg/100 g). Potato yam (*Dioscorea bulbifera*), particularly, has high content of total oxalates (780 mg/100 g), total free phenols (220–24,802 mg/100 g), amylase inhibitor activity (400mg AIU 100 g) and tannins (70–2,550 mg/100 g). High levels of tannins (245–528.6 mg/100 g) and total free phenols (141–268.6 mg/100 g) are present in various indigenous insects. No substantial levels of anti-nutrients are reported in fruits.

### Mineral Bioavailability in Indigenous Foods of India

The mineral bioavailability in IFs was predicted using phytate-to-mineral (calcium, iron and zinc) and oxalate-to-mineral (calcium) molar ratios. Secondary data on both mineral (calcium, iron and zinc) as well as anti-nutrient content (phytate and oxalate) was available for only 98 IFs (seven cereals, 10 legumes, 43 GLVs, 13 other vegetables, 21 roots and tubers, three fruits and one flesh food), which were utilized for computing molar ratios of Phy/Ca, Phy/Fe, Phy/Zn, Phy x Ca/Zn, and Ox/Ca, respectively (Table 2). Through our review, we found that most indigenous cereals (*n* = 7) have phytate-to-mineral molar ratios higher than the critical values while, values of Ox/Ca molar ratio are lower in all varieties. Finger millet emerged as one of the exceptions, which despite having a high phytate content of 306 mg/100 g, reported a low Phy/Ca molar ratio of 0.05.

Among legumes, high Phy/Fe molar ratios are observed in nine varieties, followed by high Phy/Ca and Phy/Zn molar ratios in six varieties. High bioavailability of calcium was reported in Horse-gram, a calcium-rich (260 mg/100 g) source, with a low Phy/Ca molar ratio of 0.08. Many micronutrient-rich GLVs are found to have exceptionally low molar ratios for Phy/Ca, Phy/Fe, Phy/Zn, Ox/Ca and/or Phy x Ca/Zn. Some of these GLVs include Colocasia leaves (*Colocasia esculenta*), Red amaranth leaves, Amaranth spinosus, Ponnagini (*Alternanthera sessilis*), Pumpkin leaves (*Cucurbita maxima*), Beng leaves (*Centella asiatica*), Basella leaves, Malabar spinach and many more. High mineral bioavailability is also observed in other indigenous vegetables. Some of these varieties are particularly rich sources of minerals; for instance, iron-rich sources like Breadfruit and Kovai have a low Phy/Fe molar ratio in the range of 0.21–0.49 while, Spinegourd, a calcium-rich source, has a molar Ox/Ca molar ratio of 0.41, respectively. For most calcium and iron-rich roots and tubers, phytate content was not available and hence, phytate-to-mineral molar ratios could not be calculated. However, Ox/Ca molar ratios were documented for 23 varieties, out of which most roots and tubers (*n* = 19) reported low values. Molar ratio indices in fruits could be documented for only 3 varieties, among which molar ratios of Phy/Ca and Phy x Ca/Zn are seen to be lower than the critical values. Although none of these varieties are rich sources of calcium, iron or zinc.

## Discussion

India is endowed with a rich reservoir of wild and cultivated IFs, which are adapted to the local natural ecosystems and hold special cultural significance among various indigenous communities. For generations, the indigenous communities have utilized these food sources for their food and livelihood. In the present review, we have made an attempt to create a repository of nutritive values of the IFs consumed by indigenous communities of India, along with information on their anti-nutrient content and mineral bioavailability. For this purpose, 52 Indian research studies were reviewed, from which a total of 508 IFs were documented with nutrient values, consumed by different indigenous communities across India. Out of 508 IFs, anti-nutritional values were documented for 123 IFs and anti-nutrient-to-mineral molar ratios were computed for 98 IFs.

In our review, we found that many IFs are a rich storehouse of both macro- as well as micronutrients. The traditional rice varieties, consumed by indigenous communities of Jharkhand, Meghalaya, Himachal Pradesh and Arunachal Pradesh, were found to have better nutritional content than conventional varieties. For instance, local varieties of white rice (*Pundi Goda),* red rice (*Lalhat (desi)* and *Laldhan),* brown rice (*Gopalbhok)* and sticky rice (*Minil-michudari)* display calcium content in the range of 14–21 mg/100 g, which is much higher than the calcium content of raw milled rice variety (7.49 mg/100 g) reported in IFCT (Longvah et al., 2017). Most of the Indian traditional rice varieties have protein levels comparable to local rice varieties of Africa and Thailand (Ebuehi and Oyewole, 2007), although the calorific value and iron content are higher in Indian varieties. Apart from superior nutritional qualities, the colored traditional rice varieties (such as Red rice, Brown rice and Black rice) also contain high levels of anthoxanthin and anthocyanin, and are thus known for their anti-oxidant, anti-inflammatory and anti-carcinogenic effects (Rathna Priya et al., 2019). Studies from Assam, Chhattisgarh, Meghalaya and Jharkhand have further documented that indigenous rice varieties are drought tolerant, disease resistant and require less labor and farm inputs and hence provide stable grain yield as compared to high yielding hybrid rice varieties (Das and Das, 2014; Dwivedi et al., 2017; Borah et al., 2018; Nahar et al., 2018; Ghosh-Jerath et al., 2020a, 2021). Despite the favorable nutritional and agro-ecological attributes associated with indigenous rice varieties, their cultivation is on decline due to preference for agronomically improved varieties for higher yield. Hence, efforts must be taken to reinforce the cultivation of local varieties, through conservation of TEK associated with traditional agro-ecosystems and production practices in the community.

Our review also suggests superior mineral profile in indigenous millets as compared to rice and other cereals. Existing literature indicates that while millets are consumed by different indigenous communities across India, their overall consumption (both frequency and amount) is mostly low, especially in communities where rice production and consumption has preceded dominance over other coarse cereals (DeFries et al., 2018). Current scientific evidence has established a link between reduced consumption levels of coarse cereals and the low iron intake in the Indian population (DeFries et al., 2018). Hence, this trend may not only result in poor diet quality, but may also have implications on the overall micronutrient profile of the vulnerable communities. From a production perspective, millets offer a range of benefits for the farmers-they require less water, are more adaptable to climate variation, and thus provide assured yield even in case of climate stresses (Davis et al., 2018; DeFries et al., 2018). Thus, measures must be directed toward scaling up of millet cultivation, which will help revive their consumption in the habitual diets of the people. We also found that most millets contain high phytate levels, and have high molar ratios indices of phytate-to-mineral, thus indicating the possible inhibitory effect of phytate on mineral bioavailability in these foods. Therefore, optimal food processing methods must be followed to increase the nutrient bioavailability in millets.

Indigenous legumes were not only found to be protein rich, but also were rich sources of folate, calcium and iron. In fact, some of the under-utilized legumes consumed by indigenous communities in Kerala (like Jack bean, Velvet bean, and *Mucuna monosperma*) were found to contain higher protein, iron, calcium and zinc content than staple legumes (Red gram, Green gram, Lentils) (Longvah et al., 2017). The levels of trypsin inhibitors documented for wild legumes (13.5–65.4 TIU/mg proteins) were found to be lower than the trypsin inhibitor activity in conventional legumes like pigeon pea (67.1–71.3 TIU/mg proteins) and kidney bean (79.7–87.6 TIU/mg proteins) (Singh and Eggum, 1984; Vadivel and Janardhanan, 2005). Several indigenous GLVs were found to be rich in different micronutrients, especially in comparison to the popular GLVs like Spinach, Mustard leaves and Fenugreek leaves consumed across the country (Longvah et al., 2017). Among these, many GLVs have similar or better nutritional qualities in comparison to indigenous leafy vegetables consumed in other parts of the world. For instance, comparable micronutrient levels are reported in indigenous GLVs (like Drumstick leaves, Pot cassia, Pumpkin leaves and Amaranth leaves) of Sub-Saharan Africa, while micronutrient levels (calcium, iron, zinc and/or beta-carotene) of some Indian Indigenous GLVs (Sweet potato leaves, Gogu leaves, Garkha leaves, Colocasia leaves and Purslane) are seen to be higher as compared to their African counterparts (Nutritional Contributions of Important African Indigenous Vegetables, 2009; Uusiku et al., 2010; Schönfeldt and Pretorius, 2011; Traore et al., 2018; Catarino et al., 2019). Through our review, we also found that most indigenous GLVs contain ANFs in low amounts and have phytate and oxalate-to-mineral molar ratios lower than critical values, thus indicating low inhibitory effect of phytate and oxalate on mineral bioavailability.

Different varieties of indigenous vegetables were seen to be rich in vitamin C, iron and calcium. Wild bean, an indigenous variety consumed in Manipur, was found to have a folate content of 193 μg/100 g, which is much higher than the folate content of French beans (47 μg/100 g), Field beans (127 μg/100 g) and Broad beans (20.46 μg/100 g), respectively (Longvah et al., 2017). Comparable levels of micronutrients are reported in few vegetables (Bitter gourd, Tree bean and Bitter tomato) of Thailand and Sub-Saharan Africa (Kongkachuichai et al., 2015). We also observed exceptionally high protein and calcium content in indigenous mushrooms of India, some of which (Straw mushroom, White rot fungus mushroom and *Termitomyces microcopus*) have either better or equivalent nutrient content in comparison to African varieties (Mshandete and Cuff, 2007; Nakalembe et al., 2015). It was interesting to note that the protein, calcium, zinc and vitamin C content in all indigenous mushrooms were seen to be particularly higher than Button mushrooms (Longvah et al., 2017), a common variety consumed in urban regions of India. Many indigenous fruits documented in our review were found to be micronutrient-rich, although some varieties (like Monkey Jack, Java Plum and Indian gooseberry) contained relatively lower micronutrient content as compared to native varieties in other South-Asian countries (Tariqul Islam Shajib et al., 2013).

The calcium and iron content of most insect varieties documented in our review were found to be comparable to conventional meats such as poultry, beef, pork etc., while some of the insects such as Grasshopper, Scarlet skimmer and mole cricket, had protein content higher than most flesh foods. Since most indigenous communities (specially in North-eastern and eastern parts of India), do not consume dairy products (Malhotra and Sailo, 2016), consumption of these wild insects can hence be recommended and promoted to combat the problem of malnutrition, particularly protein deficiency (Ganguly et al., 2013). Apart from insects, other bush meats such as indigenous fishes and wild meats were also found to be rich in different micronutrients and contained nutrient levels comparable to other similar varieties consumed in different countries (Kuhnlein et al., 2002; Fanzo et al., 2013).

It is also important to mention that few variations were observed in the nutritional content of similar foods consumed across different regions in India. This could perhaps be due to the different protocols followed for nutrient analysis by different laboratories as well as due to the actual variances in chemical properties of the food samples (owing to genetic factors, soil mineral content and related properties) (Oko et al., 2012). Despite their nutritional benefits and cultural importance, majority of the IFs remain underutilized in the usual diets of the indigenous communities. Studies included in our review have reported a large set of factors leading to underutilization, such as habitat destruction due to industrial activities, agricultural expansion, changing social values and lack of awareness among the younger genaration (Bhattacharjee et al., 2009; Ghosh-Jerath et al., 2020a). The consumption of indigenous and traditional crops in many communities worldwide has particularly been affected by the general perception of these foods as “poverty foods,” especially by the younger generations (Cloete and Idsardi, 2013). Moreover, due to the high opportunity cost in terms of time and effort required for sourcing wild foods and non-participation of younger generation in collection and processing of IF resources, the TEK relating to IFs is fast eroding, thus threatening their use and consumption (Ghosh-Jerath et al., 2020a). The fast growing urbanization is further leading to easy access of market foods, which has rendered most ethnic communities with altered food habits and overdependence on cheap quality energy dense foods (Mungreiphy and Kapoor, 2010; Ghosh-Jerath et al., 2020b, 2021). As documented in our review, many conventional foods are not as nutritious as IFs, and thus contribute to inferior diet quality. Additionally, the current national programmes (such as Targeted Public Distribution System) and agricultural policies are promoting the production and consumption of conventional energy dense cereals (wheat and rice) among vulnerable section of the population, which is leading to overall reduced dietary diversity (Welthungerlife, 2016). In this context, a detailed and focused research is needed to establish the differences in the nutritional and anti-nutritional content of IFs vs. the conventional staple foods, in order to mainstream the IF consumption in day-to-day diets.

Efforts are desirable to promote and integrate the IFs back into the diets of the indigenous communities. Since most indigenous communites in India are smallholder subsistence farmers (MoHF and Ministry of Tribal Affairs, 2019), domestic cultivation of indigenous varieties can be the most useful strategy for IF promotion. Collection, preservation and use of indigenous seeds in agriculture must be encouraged as much as possible among the indigenous communities, who are gradually shifting to high yielding hybrid seeds (Singh et al., 2018; Ghosh-Jerath et al., 2020a, 2021). Under Horticulture Mission [Mission for Integrated Development of Horticulture (MIDH), 2021], a centrally sponsored scheme by Indian Government, homestead production of local fruits, vegetables, mushrooms and roots and tubers can further be reinforced for encouraging IF production. There is an also an important need to make people aware about the nutritional and ecological importance of including wild IFs in local diets, through effective information, education and behavior change communication activities (Bhattacharjee et al., 2009). With Indian Government’s recent initiative under Poshan Atlas (2019), that aims to map nutritionally superior crops and food grains across different regions of India, many additional IF varieties can be identified in near future. The use of ancestral TEK among the elders, could further be leveraged for utilization of these foods from surrounding areas. In addition to this, cooking demonstrations could be conducted to give knowledge about various recipes and food preservation techniques using diverse list of IFs as well as to educate about processing techniques for removal of anti-nutrients. Most of the toxic and anti-nutritional factors could be removed by several processing methods (such as soaking, germination, blanching, fermentation and many others) but extensive research is still required to discover elimination methods for heat stable anti-nutrients and their impact on nutrient retention.

### Study Limitations

Due to the narrative nature of the present review, no criteria were established to evaluate the methodological quality of the studies included in the review. Although we have reported the methodology descriptions of the included studies, with specific information on the analytical procedures followed for nutritional and antinutriitonal analysis of the foods.

## Conclusion

A diverse variety of nutrient-dense IFs are available in India which have the potential to alleviate the persisting problem of hunger and malnutrition in Indian indigenous communities. However, until recently, these foods did not receive the desirable attention among policymakers and programme practitioners. The IF database collated in our review can thus serve as a resource for nutrition researchers, experts and practitioners to better understand the diversity of region-specific IFs and to advocate their incorporation in food based nutrition programmes. This review further highlights the need for a more detailed research on the nutrient and anti-nutrient values of IFs and its potential role in alleviating malnutrition, especially among vulnerable segments of the population. Although the consumption of IFs among indigenous communities has eroded over time, it could be resurrected with contextual food-based interventions through proper framework, policy, and information dissemination. Documenting and popularizing the nutritional benfits of IFs will be crucial for enhancing their consumption, and improving their demand in the global food systems. At the national level, holistic government policies in India like POSHAN Atlas and Horticulture Mission, which are aimed at identification and production of region specific foods, will be imperative to shift our focus from a small subset of foods to a diverse food base contextualized to regional, cultural and traditional food habits that may potentially enrich the dietary quality of the vulnerable population. We therefore, advocate the recognition of these foods and highlight the need for researchers, universities and government agencies to implement coordinated efforts to maximize the nutritional potential of IFs in terms of production, post-harvest and processing, marketing, and consumption, while understanding the ecological, social, cultural and economic context of IFs for their sustainable utilization and consumption Globally, a right step in this direction could lead to the diversification of existing global food systems, that may potentially reward us with diets that provide better nutrition, have low environmental impacts, are culturally acceptable and economically accessible to all.

## Supplementary Material

Data sheet 1

Table 1

Table 2

Table 3

## Figures and Tables

**Table 1:** Nutritive values of selected indigenous foods of India (*n* = 69).

Common name	Botanical name	Vernacular names	Energy (kcal/100g)	Protein (g/100g)	Fat (g/100g)	Ca (mg/100 g)	Iron (mg/100 g)	Zinc (mg/100 g)	Vit A (μg/100g)	Vit C (mg/100 g)	B1 (mg/100 g)	B2 (mg/100 g)	B3 (mg/100 g)	B6 (mg/100 g)	B9 (μg/100g)	References
**Cereals**																
Red rice	*Oryza sativa* L.	*Kba-bakut*	344	7.1	0.7	11.5	3.02	1.6			0.2	0.1	1.1	0.1	1,203	Chyne et al., 2019
Italian millet	*Setaria italica* (L.) P.Beauv.	*Kangni*	336	12.3	4.3	31.0	2.8	-	-	-	-	-	-	-	-	Sebestianus Lakra, 2019
			357	11.2	2.6	21.0	10.2	-	-	-	0.2	-	2.3	-	-	Rajyalakshmi and Geervani, 1994
Finger millet	*Eleusine coracana* (L.) Gaertn.	*Tella*	352	7.4	1.8	302	19.3	-	-	-	0.4	-	1.2	-	-	Rajyalakshmi and Geervani, 1994
		*Punasa*	331	7.0	1.8	307	18.8	-	-	-	0.3	-	1.0	-	-	Rajyalakshmi and Geervani, 1994
		*Burada*	347	7.0	1.4	264	8.6	-	-	-	0.3	-	1.0	-	-	Rajyalakshmi and Geervani, 1994
		*Mandua/* *Kodde/* *Naglana*	321	7.2	1.9	364	4.6	2.5	2	-	0.4	0.2	1.3	0.1	35	Longvah et al., 2017
**Nuts & legumes**																
Horse gram	*Dolichos biflorus* L	*Kulthi/Kulad*	321	22.0	0.5	269.	8.8	2.7	59	-	0.3	0.2	1.8	0.2	163	Longvah et al., 2017
		*Kulthi (Black variety)*	349	22.2	3.0	263.0	38.6	-	-	-	0.3	-	3.5	-	-	Rajyalakshmi and Geervani, 1994
		*Kulthi (white variety)*	348	22.8	2.9	351.0	37.5	-	-	-	0.2	-	3.0	-	-	Rajyalakshmi and Geervani, 1994
Hairy wetch	*Vicia hirsuta* (L.) Gray	*Baturi/Teeri reeti*	361	26.9	1.9	21	7.8	4.1	550	23	-	-	2.0	-	7	Ghosh-Jerath et al., 2016
Field Bean	*Dolichos lablab* L.	Field bean	347	24.9	0.8	60.0	9.3	-	-	-	-	-	-	-	-	Sebestianus Lakra, 2019
		Field bean (Black variety)	276	19.9	0.9	78.2	4.5	2.4	-	-	0.4	0.1	1.9	0.4	291	Longvah et al., 2017
			345	20.3	1.4	61.0	10.4	-	-	-	0.1	-	1.7	-	-	Rajyalakshmi and Geervani, 1994
		Field bean (white variety)	280	19.8	0.9	77.2	5.5	2.8	-	-	0.4	0.1	2.0	0.4	289	Longvah et al., 2017
			355	20.3	2.4	62.0	6.1	-	-	-	0.1	-	1.1	-	-	Rajyalakshmi and Geervani, 1994
		Field bean (red variety)	283	19.9	0.9	75.2	4.9	2.4	-	-	0.3	0.1	2.0	0.4	292	Longvah et al., 2017
			364	20.3	2.9	78.0	11.8	-	-	-	0.2	-	2.5	-	-	Rajyalakshmi and Geervani, 1994
Rice bean	*Phaseolus calcaratus* Roxb.	*Sutro/sutri*	332	21.5	0.3	302.0	-	-	-	-	-	-	-	-	-	National Institute of Nutrition et al., 1978
Jack bean	*Canavalia ensiformis* (L.) DC.		375	35	4.3	497.9	5.2	4.3	-	-	-	-	-	-	-	Vadivel and Janardhanan, 2005
Sword bean	*Canavalia gladiata* (Jacq.) DC.	*Badi sem*	379	25.5	3.3	510.1	10.9	6.6	-	-	-	-	-	-	-	Vadivel and Janardhanan, 2005
Pot Casia	*Senna obtusifolia* (L.) H.S. Irwin & Barneby		389	20.3	7.4	572.2	10.7	30	-	-	-	-	-	-	-	Vadivel and Janardhanan, 2005
Job’s tears	*Coix lacryma* var. *stenocarpa* Oliv.	*Riew magain*	-	13.3	7	1,100	2.4	5.1	-	-	-	-	-	-	-	Agrahar-Murugkar and Subbulakshmi, 2005
Perilla	*Perilla frutescens* (L.) Britton	*Nei lieh*	484	23.9	23.8	336	8.3	5.02	-	-	0.3	0.1	1.7	0.1	178	Chyne et al., 2019
		*Hanshi*	615	17.4	51.7	269	9	4.7	-	-	-	-	-	-	-	Longvah and Deosthale, 1991
**Green leagy vegetables**																
Malabar spinach	*Basella rubra* L.	*Poi saag/*	-	5.2	0.9	-	-	-	-	83.7	-	-	-	-	-	Bhardwaj et al., 2009
		*Pondka saag/*	-	-	-	203.0	8.4	-	179	138.0	-	-	-	-	-	Singh et al., 2018
		*Poi saag*	21	3.1	-	160.5	3.4	0.4	62	3.0	-	-	-	-	-	Ghosh-Jerath et al., 2020a
Basella leaves	*Basella alba* L.	*Bon Pui Sak*	-	2.8	0.4	-	-	-	-	87.0	-	-	-	-	-	Jana, 2004
			-	-	-	138.0	9.4	-	786	297.0	-	-	-	-	-	Singh et al., 2018
			20	1.5	0.4	93.8	4.2	0.4	2,473	63.4	0.1	0.2	0.5	0.2	90	Longvah et al., 2017
Vegetable fern	*Diplazium esculentum* (Retz.) Sw.	*Dhekia/* *Churuli/* *Tyrkhang*	-	7.4	0.8	-	-	-	-	18.6	-	-	-	-	-	Bhardwaj et al., 2009
			-	3.6	0.4	112	1.8	0.9	833	32.0	-	-	-	-	-	Pradeepkumar et al., 2015
			-	17.4	5.6	1,290	-	-	-	-	-	-	-	-	-	Tag et al., 2014
			-	-	-	47.9	1.6	-	1	-	-	-	-	-	-	Agrahar-Murugkar, 2006
			-	-	-	-	2.9	3.8	-	147.0	-	-	-	-	-	Medak and Singha, 2018
*Gaam oying*	*Glochidion multiloculare* (Rottler ex Willd.) Voigt	*Gaam oying*	-	9.2	3.6	-	-	-	-	61.3	-	-	-	-	-	Bhardwaj et al., 2009
Weaver’s Beam tree	*Schrebera swietenioides Roxb.*	*Mokha*	111	3.5	0.9	831.0	5.1	0.7	-	-	-	-	-	-	-	Bhattacharjee et al., 2009
*Polpala/* *Kapurijadi*	*Aerva lanata* (L.) Juss.	*Lapongarak/* *Lupu ara*	56	4.6	-	322	22.1	0.7	21 760	19	-	-	7.0	-	41	Ghosh-Jerath et al., 2016
Pot Casia	*Senna obtusifolia* (L.) H.S. Irwin & Barneby	*Chakod ara/ Chakod/ Kanyur aa/ Thakara/ Panwar*	49	5.0	0.8	520.0	12.4	-	10,512	0.1	0.2	0.8	-	-	-	National Institute of Nutrition et al., 1978
			-	5.3	0.9	720	6.7	1.4	1,822	151.8	-	-	-	-	-	Pradeepkumar et al., 2015
			-	20.3	23.0	-	-	-	-	-	-	-	-	-	-	Jain and Tiwari, 2012
Red amaranth Drumstick leaves	*Amaranthus* *gangeticus* L. *Moringa oleifera* Lam.	*Lal Bhaji* *SajneSak/ Sahjana/Muriara/ Mungaara/ Sanjhodi ghasi/ Munga saag/* *Munga-arak/* *Mulga aa/* *Sehjana saag*	33	3.9	0.6	245.0	7.3	1.4	8,457	86.2	0.0	0.3	0.6	0.22	82	Longvah et al., 2017
			-	6.7	1.7	-	-	-	-	220	-	-	-	-	-	Jana, 2004
			-	-	-	18	2.8	-	595	130	-	-	-	-	-	Singh et al. 2018
			67	6.4	1.6	314	4.6	0.7	17,542	108	0.1	0.5	-	-	43	Longvah et al. 2017
			92	6.1	-	440	0.9	-	5,520	99	-	-	-	-	-	Horo and Topno 2015
			-	19.5	22.8	-	-	-	-	-	-	-	-	-	-	Jain and Tiwari, 2012
Agathi leaves	*Sesbania grandiflora* (L.) Pers	*Agathi*	71	8.0	1.4	901	4.4	0.5	12,582	121.0	0.3	0.3	1.2	0.2	120	Longvah et al., 2017
			-	-	-	404	5.0	-	1,946	304.0	-	-	-	-	-	Singh et al., 2018
Kantha leaves	*Euphorbia granulate* Forssk.	*Daav ghasi/* *Kantha-arak*	46	3.5	-	425.0	81.1	1.0	11,680	9.0	3.1	-	-	-	7	Ghosh-Jerath et al., 2016
Himalayan mayflower	*Maianthemum purpureum* (Wall.) LaFrankie	-	-	27.2	5.5	1,090	-	-	-	-	-	-	-	-	-	Tag et al., 2014
Yellow Gulmohar	*Delonix elata* (L.) Gamble	*Vayunarayani*	-	7.1	-	112.0	6.2	0.6	10,510	295.0	0.3	-	-	-	-	Gupta et al., 2005
*Kaattupaaval*	*Momordica sahyadrica* Kattuk. and V.T.Antony	*Kaattupaaval*	-	2.6	0.6	1,360	5.2	1.7	1,684	54.9	-	-	-	-	-	Pradeepkumar et al., 2015
Lesua	*Digera muricata* (L. Mart.)	*Gurchi*	-	4.3	-	506	17.7	0.6	3,360	49	0.1	-	-	-	-	Gupta et al., 2005
Star Gooseberry	*Sauropus androgynus* (L.) Merr.	*Chakurmani*	-	-	-	409	21	-	195	314	-	-	-	-	-	Singh et al., 2018
**Other vegetables**																
Kachnar flower	*Bauhinias variegata* L.	*Kachna* *Phool/Burju* *Baha*	83	2.9	-	404.9	3.4	0.6	416	2.5	-	0.3	-	-	-	Ghosh-Jerath et al., 2020a
Sanai Flower	*Crotalaria juncea* L.	*Sonpu* *Phool/Jiri Ba*	120	2.9	-	320.2	7.6	0.2	1,113	1.8	3.1	-	-	-	-	Ghosh-Jerath et al., 2020a
Kattian/Kasai	*Bridelia retusa* (L.) A.Juss.	*Bon Chalta*	-	1.0	0.2	-	-	-	-	277.0	-	-	-	-	-	Jana, 2004
Breadfruit	*Artocarpus altilis* (Parkinson ex F.A.Zorn) Fosberg	*Bilayati katahal*	-	-	-	40.0	3.8	-	682	87.0	-	-	-	-	-	Singh et al., 2018
Spiny bitter gourd	*Momordica cochinchinensis* (Lour.) Spreng	*Jangli kakrol*	-	-	-	110.0	12.8	-	463	210.0	-	-	-	-	-	Singh et al., 2018
Spiny bitter gourd	*Momordica subangulata* subsp renigera (Wall. ex G.Don) W.J.de Wilde	*Kakrol*	-	-	-	198.0	15.7	-	289	88.0	-	-	-	-	-	Singh et al., 2018
Wild plantain, flower	Ensete Superbum (Roxb.) Cheesuran	*Ran-keli,* *Chaveli-keli*	-	-	-	665.6	518	3.8	-	-	-	-	-	-	-	Mahadkar et al., 2012
Dhawal	*Woodfordia fruticosa* (L.) Kurz	*Dhayati*	-	-	-	219.4	55.1	1.6	-	-	-	-	-	-	-	Mahadkar et al., 2012
Thai eggplant	*Solanum virginianum* L	*Sohthang*	-	11.3	1.5	990	3.2	1.8	-	321.5	-	-	-	-	-	Agrahar-Murugkar and Subbulakshmi, 2005
Plantain, flower	*Musa* × *paradisiaca* L.	*Tarkari kela*	-	-	-	32	76	-	292	249	-	-	-	-	-	Singh et al., 2018
			21	1.5	0.6	34.1	0.4	0.4	35	6.5	0.0	0.0	0.3	0.1	49	Longvah et al., 2017
**Mushrooms**																
-	*Pleurotus sajor-caju* (Fr.) Singer		-	37.9	1.0	-	-	-	-	-	-	-	-	-	-	Saha et al., 2014
-	*Lactarius quieticolor* Romagn.		-	19.0	2.6	1,460	19.4	39.4	-	18.1	-	-	-	-	-	Agrahar-Murugkar and Subbulakshmi, 2005
Girolle	*Cantharellus cibarius*		-	21.1	1.6	420.0	53.5	6.8	-	41.9	-	-	-	-	-	Agrahar-Murugkar and Subbulakshmi, 2005
Gray coral	*Clavulina cinerea*		-	27.5	2.5	1,910	75.2	11.1	-	41.8	-	-	-	-	-	Agrahar-Murugkar and Subbulakshmi, 2005
Flat Bulb mushroom	*Agaricus abruptibulbus*		343	20.3	1.8	152	-	0.1	-	-	-	-	-	-	-	Sudheep and Sridhar, 2014
-	*Termitomyces* *globulus*		373	23.8	4.3	101	0.2	1.2	-	-	-	-	-	-	-	Sudheep and Sridhar, 2014
Russula	*Russula integra*		-	21.1	4.5	1,270	56.2	10.5	-	19.6	-	-	-	-	-	Agrahar-Murugkar and Subbulakshmi, 2005
Wooly chanterelle	*Gomphus floccosus*		-	21.2	5.3	1,370	22.3	13	-	25.8	-	-	-	-	-	Agrahar-Murugkar and Subbulakshmi, 2005
**Roots & tubers**																
Indian yam	*Dioscorea oppositifolia* L.	*Pan Alu/* *Arika tega/* *Paani Alu*	409	13.8	6.3	230.0	49.1	1.4	-	104.7	-	-	17.6	-	-	Arinathan et al., 2009
			406	13.4	7.4	294.1	32.1	1.6	-	96.4	-	-	37.1	-	-	Shajeela et al., 2011
			350	6.3	2.5	880.6	32.0	5.2	-	80.6	-	-	64.7	-	-	Arinathan et al., 2009
			369	8.4	4.4	646.2	40.8	6.3	-	90.5	-	-	44.3	-	-	Shajeela et al., 2011
			386	7.0	6.9	680.6	22.0	3.2	-	-	-	-	-	-	-	Mohan and Kalidass, 2010
			-	1.4	0.1	-	-	-	-	2.0	-	-	-	-	-	Jana, 2004
			100	1.8	1.1	45.0	4.7	-	-	-	0.0	0.0	0.3	-	-	Rajyalakshmi and Geervani, 1994
			-	9.5	1.6	-	-	-	-	5.7	-	-	-	-	-	Padhan et al., 2020
Five leaf yam	*Dioscorea pentaphylla* L.	*Nappe/Hasaer Sanga/Kanta* *Alu* *Pandimukku tega/Panja sanga*	395	5.4	6.0	640.1	113.4	3.2	-	91.7	-	-	53.5	-	-	Arinathan et al., 2009
			388	6.5	6.2	444.2	66.3	3.4	-	96.6	-	-	62.1	-	-	Shajeela et al., 2011
			72	4.4	-	33.2	55.9	0.6	-	3.1	1.1	-	-	-	-	Ghosh-Jerath et al., 2020a
			384	9.2	4.8	632.1	103	3.1	-	-	-	-	-	-	-	Mohan and Kalidass, 2010
			-	1.5	0.3	-	-	-	-	7.0	-	-	-	-	-	Jana, 2004
			72	2.8	0.7	139.0	7.2	-	-	-	0.0	0.0	0.1	-	-	Rajyalakshmi and Geervani, 1994
			-	9.2	0.6	-	-	-	-	4.2	-	-	-	-	-	Padhan et al., 2020
Nurai	*Dioscorea tomentosa*		409	8.5	5.9	240.3	23.7	6.2	-	55.7	-	-	88.4	-	-	Arinathan et al., 2009
	J.Koenig ex Spreng.		402	9.5	6.0	266.4	28.5	5.4	-	65.2	-	-	74.1	-	-	Shajeela et al., 2011
			389	8.3	6.8	272.1	24.6	5.2	-	-	-	-	-	-	-	Mohan and Kalidass, 2010
Floating lace plant	*Aponogeton natans* (L.) Engl. & K.Krause		386	5.3	2.9	312.3	30.1	2.2	-	-	-	-	-	-	-	Mohan and Kalidass, 2010
Swallow root	*Decalepis hamiltonii* Wight & Arn.		399	4.4	10.2	386.1	51.1	2.2	-	-	-	-	-	-	-	Mohan and Kalidass, 2010
Anantmul	*Hemidesmus indicus* (L.) R. Br. ex Schult.		405	4.4	6.2	432.0	44.1	2.2	-	-	-	-	-	-	-	Mohan and Kalidass, 2010
Aromatic ginger	*Kaempferia galanga* L.	*Sying smoh*	-	4.7	10.2	950	69.9	8.4	182	-	-	-	-	-	-	Agrahar-Murugkar and Subbulakshmi, 2005
**Fruits**																
Banyan fruit	*Ficus benghalensis L.*	*Pakkedi/Baadi*	72	1.7	2.0	364.0	-	-	-	-	-	-	-	-	-	-	National Institute of Nutrition et al., 1978
*Zoge*	*Melodinus cochinchinensis* (Lour.) Merr	*Zoge*	405	7.2	6.4	1,207	22	43	-	-	-	-	-	-	-	Seal et al., 2016
Bird cherry	*Prunus bracteopadus* Koehne	*Sohlang*	-	4.6	0.7	1,220	10.7	1.5	257	608.9	-	-	-	-	-	Agrahar-Murugkar and Subbulakshmi, 2005
*Sohngang rit*	*Solanum indicum* L.*Sohngang rit*	-	14.1	18.8	1,300	4.5	3.5	38	826.4	-	-	-	-	-	-	Agrahar-Murugkar and Subbulakshmi, 2005
*Soh Priam* *Khlaw*	*Helicia nilagirica* Bedd.	*Soh Priam* *Khlaw*	378	3.3	1.1	206	15	27	-	-	-	-	-	-	-	Seal and Chaudhuri, 2014
*Soh Phoh* *Khlaw*	*Ilex venulosa* Hook.f.	*Soh Phoh* *Khlaw*	374	3.3	1.3	918	22	48	-	-	-	-	-	-	-	Seal and Chaudhuri, 2014
*Soh-mlum/* *Sohma*	*Rhus chinensis* Mill.	*Soh-mlum/* *Sohma*	385	7.9	5.8	335	14	40	-	-	-	-	-	-	-	Seal and Chaudhuri, 2014
*Sohthliem*	*Gomphogyne* *cissiformis*	*Sohthliem*	-	14.4	3.6	1,170	6.2	3.8	-	273.5	-	-	-	-	-	Agrahar-Murugkar and Subbulakshmi, 2005
**Flesh foods**																
Singhi	*Saccobranchus* *fossilis*	*Singhi*	124	22.8	0.6	670.0	2.3	-	-	-	-	0.8	-	-	-	National Institute of Nutrition et al., 1978
Freshwater Eel	*Anguilla Anguilla*	*Gacchi*	108	20.4	2.6	53.0	1.5	2.2	866	-	-	0.3	-	-	1,294	Longvah et al., 2017
Guntea loach	*Lepidocephalichthys guntea*	Ngakijou				2,150	13.6	3.1								Shantosh and Sarojnalini, 2018
Grasshopper	*Chondacris rosea*	*Mirbo/ ‘Takam/ Kamrak*	373	68.8	7.8	340.0	7.8	10.8	-	-	-	-	-	-	-	Chakravorty et al., 2014
-	*Oedaleus abruptus* (Thunberg)	-	587	60	-	-	0.1	0.2	4,640	6.3	0.5	1	6	-	-	Ganguly et al., 2013
Scarlet skimmer	*Crocothemis servilia* (Drury)	-	497	70.5	4.9	86.5	11.3	9.3								Shantibala et al., 2014
Giant water bug	*Lethocerus indicus* (Lepeletier and Serville)	-	632	22.6	13.8	96	410	29.5	-	-	-	-	-	-	-	Shantibala et al., 2014
Water scorpion	*Laccotrephes maculatus* (F.)	-	585	25.1	6.9	24.3	461	11.8	-	-	-	-	-	-	-	Shantibala et al., 2014

**Table 2:** Molar ratio indices of Indigenous foods consumed in India (*n* = 98).

Food Item	Botanical name	Phytate/ calcium	Phytate/ iron	Phytate/ zinc	Oxalate/ calcium	Phytate x Calcium/zinc
**Cereals**						
White Rice	*Oryza sativa* L	2.15	34.7	21.8	0.12	4.09
Bajra	*Pennisetum typhoideum* Rich.	1.09	6.33	17.06	0.89	11.51
Sorghum	*Sorghum vulgare* Pers.	1.21	11.94	28.46	0.47	19.64
Maize, dry	*Zea mays* L.	4.4	21.9	27.6	0.78	6.15
Ragi	*Eleusine coracana* (L.) Gaertn	0.05	5.64	12.05	0.05	109.70
Little millet	*Panicum antidotale* Retz.	1.00	18.74	14.50	0.19	5.84
Kodo millet	*Paspalum scrobiculatum* L.	1.80	16.67	26.19	0.10	9.95
**Pulses**						
Cowpea, brown	*Vigna catjang* (L.) Walp.	0.41	7.91	15.93	0.08	32.54
Cowpea, white	*Dolichos catjang* Burm.f	0.41	9.72	15.68	0.09	32.96
Field bean, black	*Dolichos lablab* L.	0.59	14.31	31.15	0.01	60.89
Field bean, brown	*Dolichos lablab* L.	0.63	12.33	28.10	0.01	54.24
Field bean, white	*Dolichos lablab* L.	0.64	13.70	32.46	0.01	61.02
Horse gram, whole	*Dolichos biflorus* L	0.08	3.27	12.37	0.31	83.16
Lentil dal	*Lens culinaris* Medik.	0.30	2.61	5.96	0.11	6.60
Kidney beans	*Phaseolus vulgaris* L.	0.11			0.08	
Black gram, dal	*Phaseolus mungo* L	0.63	10.45	19.01	0.36	26.47
Red gram, dal	*Cajanus cajan* (L.) Millsp.	0.13	3.85	10.49	0.01	33.05
**Green leafy vegetables**						
Colocasia leaves	*Colocasia esculenta* (L.) Schott	0.00	0.36	1.77	1.48	9.57
					2.51	
		0.00	0.01		0.06	
Drumstick leaves	*Moringa oleifera* Lam.	0.02	2.36	18.01	0.17	141.37
		0.06	0.55		0.40	
Amaranth leaves, red	*Amaranthus gangeticus* L.	0.00	0.06	0.34	1.53	2.11
Amaranth spinosus, leaves, green	*Amaranthus spinosus* L.			0.00	1.36	
Agathi leaves	*Sesbania grandiflora* (L.) Pers.	0.00	1.22	12.47	0.09	280.85
		0.00	0.24		0.04	
Ponnaganni	*Alternanthera sessilis* (L.) R.Br. ex DC.	0.01	0.70	3.15	0.54	30.59
					0.49	
Tamarind leaves, tender	*Tamarindus indica* L.	0.03	1.02	3.70	1.02	6.19
Gogu leaves, red	*Hibiscus sabdariffa* L.	0.03	1.11	0.27	0.98	0.58
		0.00	0.01		0.03	
Pumpkin leaves	*Cucurbita maxima* L.	0.01	0.58	4.19	0.02	28.39
		0.00	0.18	1.52	0.30	11.44
Curry leaves	*Murraya koenigii* (L.) Spreng.	0.00	0.40	3.36	0.11	55.30
		0.00	0.07		0.01	
Basella leaves	*Basella alba* L.	0.03	1.00	12.19	0.82	28.58
		0.01	0.17		0.13	
Prickly Chaff Flower	*Achyranthes aspera* L.				0.19	
Pot Casia leaves	*Senna obtusifolia* (L.) H.S.Irwin & Barneby				0.38	
Beng leaves	*Centella asiatica (L.)* Urb.				0.85	1.01
		0.00	0.01	0.23	0.16	
		0.00	0.01		0.02	
Vegetable fern	*Diplazium esculentum* (Retz.) Sw.				0.88	
Common Leucas	*Leucas aspera* (Willd.) Link.				0.59	
*Kaattupaaval*	*Momordica sahyadrica* Kattuk. and V.T.Antony				0.07	
Purslane	*Portulaca oleracea* L.				0.22	
		0.00	0.01		0.11	
Black night shade	*Solanum nigrum* L.				0.84	
Chinese Spinach	*Amaranthus tricolor* L				0.88	
		0.00	0.01	0.32	2.42	1.91
		0.00	0.04		0.04	
Malabar spinach	*Basella rubra* L.	0.09	2.90		0.06	
Water spinach	*Ipomoea aquatica* Forssk.^€^	0.01	0.04		0.06	
Alligator weed	*Alternanthera philoxeroides* (Mart.) Griseb.	0.00	0.02		0.06	
Purple amaranth	*Amaranthus lividus* L.	0.00	0.01		0.08	
Slender amaranth	*Amaranthus viridis* L.	0.00	0.05		0.03	
		0.00	0.01		1.06	
White jute	*Corchorus capsularis* L.	0.01	0.20		0.05	
Male fern	*Dryopteris filix-mas* (L.) Schott.	0.02	0.32		0.47	
*Helencho*	*Enhydra fluctuans* Lour.	0.01	0.02		0.03	
Wild coriander	*Eryngium foetidum* L.	0.01	0.03		0.06	
*Kulekhara*	*Hygrophila auriculata* (Schumach.) Heine	0.01	0.04		0.04	
Water primrose	*Jussiaea repens* L.	0.01	0.06		0.06	
Wild Betel	*Piper sarmentosum* Roxb.	0.01	0.73		0.02	
Star Gooseberry	*Sauropus androgynus* (L.) Merr.	0.01	0.14		0.03	
Black pigweed leaves	*Trianthema portulacastrum* L.	0.00	0.04	0.50	9.44	0.65
Garkha leaves	*Celosia argentea* L.	0.00	0.03	1.45	2.06	7.36
Balae leaves	*Polygala erioptera* DC.	0.00	0.06	0.48	0.67	0.49
Gadhakand leaves	*Boerhavia diffusa* L.	0.00	0.04	1.00	1.72	8.29
Mexican mint	*Plectranthus amboinicus* (Lour.) Spreng.	0.00	0.03	0.30	0.14	1.19
Lesua	*Digera muricata* (L.) Mart.	0.00	0.01	0.41	1.27	5.17
Javanada leaves	*Cocculus hirsutus* (L.) W.Theob.	0.00	0.04	0.72	0.83	2.28
Kena leaves	*Commelina benghalensis* L.	0.00	0.03	0.39	1.57	1.11
Gandhuli	*Cleome gynandra* L.	0.01	0.23	2.57	0.08	7.27
Yellow Gulmohar leaves	*Delonix elata* (L.) Gamble	0.00	0.07	0.84	0.37	2.35
**Other vegetables**						
Ash gourd	*Benincasa hispida* (Thunb.) Cogn.	0.05	2.48	14.38	0.12	6.94
Bamboo shoot, tender	*Bambusa vulgaris Schrad. ex J.C. Wendl.*	0.12	5.60	4.88	3.90	1.22
Bittergourd	*Momordica charantia* L.	0.05	1.10	3.52	1.35	1.43
Kovai, big	*Coccinia grandis* (L.) Voigt	0.02	2.77	9.65	0.09	8.95
		0.01	0.21		0.06	
Ridge gourd	*Luffa acutangula* (L.) Roxb.	0.07	3.16	7.34	0.98	2.51
Ridge gourd, smooth skin	*Luffa acutangula* (L.) Roxb.	0.06	2.53	7.34	1.09	2.73
Spine gourd	Momordica diocia Roxb ex Willd				0.41	
	*Caralluma adscendens* var. *attenuata* (Wight) Grav. & Mayur				0.49	
	*Caralluma pauciflora* (Wight) N.E.Br.				0.37	
Indian shot	Canna indica L.				2.77	
Plantain, flower	*Musa* × *paradisiaca* L.	0.02	0.01		0.61	
		0.00	0.53	0.62	2.25	0.53
Jackfruit	*Artocarpus heterophyllus* Lam.	0.03	0.48		0.43	38.71
		0.09	19.46	33.88	0.10	
Breadfruit	*Artocarpus altilis* (Parkinson ex F.A.Zorn) Fosberg	0.03	0.49		0.41	
**Roots & tubers**						
Potato Yam	*Dioscorea bulbifera* L.				2.95	
Indian yam	*Dioscorea oppositifolia* L.				0.25	
					0.91	
					0.19	
Five leaf yam	*Dioscorea pentaphylla* L.				0.32	
					0.41	
Nurai	*Dioscorea tomentosa* J.Koenig ex Spreng.				0.05	
					0.53	
Colocasia	*Colocasia esculenta* (L.) Schott	0.03	1.64	3.34	0.73	2.52
					0.52	
		0.01	0.08		0.18	
Tapioca	*Manihot esculenta* Crantz.	0.15	6.83	63.44	0.30	41.08
Floating lace plant	*Aponogeton natans* (L.) Engl. & K.Krause				0.33	
Boerhavia chinensis	*Boerhavia chinensis* (L.) Rottb.				0.14	
Hadjod/Veldt grape	Cissus quadrangularis L.				0.19	
Kattumunthiri	Cissus vitiginea L.				0.14	
Queen sago	*Cycas circinalis* L.				0.85	
Swallow root	*Decalepis hamiltonii* Wight & Arn.				0.33	
Athikizhangu	Dioscorea spicata Roth				0.87	
					0.85	
Anantmul	*Hemidesmus indicus* (L.) R. Br. ex Schult.				0.19	
Ipomoea sumatrana	*Ipomoea sumatrana* (Miq.) Ooststr.				0.85	
Kedrostis foetidissima	*Kedrostis foetidissima* (Jacq.) Cogn.				0.10	
Maerua oblongifolia	*Maerua oblongifolia* (Forssk.) A.Rich.				0.54	
Koka	*Nymphaea pubescens* Willd.				0.53	
Water lily	Nymphaea rubra Roxb. ex Andrews				0.54	
	*Parthenocissus neilgherriensis* Planch.				3.36	
Purple Yam	*Dioscorea alata* L.				0.59	
		0.10	0.32		0.07	
**Fruits**						
Goosberry	*Phyllanthus emblica* L.	0.15	3.22	48.55	0.18	24.4
Wood Apple	*Aegle marmelos* (L.) Correa	0.13	42.85	99.47	0.53	119.11
Indian Jujube	*Ziziphus jujuba* Mill.	0.11	18.16	84.30	0.03	98.21
**Flesh foods**						
Grasshopper	*Oedaleus abruptus* (Thunberg)		7.21	4.19		
