# Supplementary material for: Indigenous Foods of India: A Comprehensive Narrative Review of Nutritive Values, Antinutrient Content and Mineral Bioavailability of Traditional Foods Consumed by Indigenous Communities of India
Source: Front Sustain Food Syst. Author manuscript; Available in PMC 2022 May 22. (PMC7612755; doi:10.3389/fsufs.2022.696228)
Supplement: Data sheet 1 [file EMS145028-supplement-Data_sheet_1.pdf]

Table 1: Indigenous foods consumed by local and ethnic communities of India (n=508)

Common name	Botanical name	Part consumed	Vernacular name	Accessed by indigenous community	Reference
1-3. White Rice	*Oryza sativa* L.	Grain	*Jadhan*	Santhal (Jharkhand)	(Ghosh-Jerath et al., 2016)
			Pundi Goda	Munda (Jharkhand)	(Ghosh-Jerath et al., 2021)
			Dhan	Sauria Paharia(Jharkhand)	(Ghosh-Jerath et al., 2020a)
4-15 Red rice	*Oryza sativa* L.	Grain	*Kba-lwai, Kba-bakut, Kba-baswoit*	Khasi (Meghalaaya)	(Chyne et al., 2019)
			Karad, HPR-2143, Matali, Begmi, Bhrigu dhan, Sukara, Chohartu	Indigenous communitiesof Himachal Pradesh	(Sharma et al., 2012)
			Laldhan	Savera, Jatapu, Gadabe and Kondadora (Andhra Pradesh)	(Rajyalakshmi and Geervani, 1994)
			Lalhat, desi	Oraon (Jharkhand)	(Ghosh-Jerath et al., 2015b)
16-40. Brown rice	*Oryza sativa* L.	Grain	*Gopalbhok, Hathidat gipok, Hathidatgitchak, Hati bandha, Jahagipok, Jahagisim, Kala jira jaha, Khisore, Kutchigisim, Maigothi, Mandami, Mi katchi, Mi-bi-sa, Mibol, Miganggetlmi chambak, Mima, Mimittim midokru, Mima gisim, Mimittim midokru, Mim-kudep, Ranga bhok jaha, Sarangma bolma, Silgothi, Taramon*	Garo (Meghalaya)	(Longvah et al., 2020)
			Kba-stem	Khasi (Meghalaaya)	(Chyne et al., 2019)
41-49. Sticky rice	*Oryza sativa var. glutinosa*	Grain	*Dudh binni, Maubinni, Minil gisim, Minil gitchak, Minil jaha, Minil na,singket, Minil-michudari, Raga-binn*	Garo (Meghalaya)	(Longvah et al., 2020)
			Kba-shulia	Khasi (Meghalaaya)	(Chyne et al., 2019)
50. Pearl millet	*Pennisetum typhoideum* Rich.	Kernel	*Bajra*	Bhil (Gujarat), Savera, Jatapu, Gadabe and Kondadora (Andhra Pradesh)	(Rajyalakshmi and Geervani, 1994; Bhattacharjee et al., 2009)
51. Sorghum	*Sorghum vulgare* Pers.	Grain	*Jowar*	Bhil (Gujarat)	(Bhattacharjee et al., 2009)
52. Maize	*Zea mays* L.	Cob	*Jondra/ Makai/Makka*	Sauria Paharia,Munda,Santhal, Oraon (Jharkhand), Bhil (Gujarat)	(Bhattacharjee et al., 2009; Ghosh-Jerath et al., 2015b, 2016, 2020a, 2021)
53. Indian goosegrass	*Eleusine indica* (L.) Gaertn.	Grain	*Madiya*	Baiga (Madhya Pradesh) (Madhya Pradesh)	(Sebestianus Lakra, 2019)
54. Italian millet	*Setaria italica* (L.) P.Beauv.	Grain	*Kangni* (Baiga (Madhya Pradesh)	Baiga (Madhya Pradesh) (Madhya Pradesh), Savera, Jatapu, Gadabe and Kondadora (Andhra Pradesh)	(Rajyalakshmi and Geervani, 1994; Sebestianus Lakra, 2019)
55. Salharkutki	NA	Grain		Baiga (Madhya Pradesh)	(Sebestianus Lakra, 2019)
56-57. Little millet	*Panicum antidotale* Retz.	Grain	*Samai (Parboiled)*	Savera, Jatapu, Gadabe and Kondadora (Andhra Pradesh)	(Rajyalakshmi and Geervani, 1994)
			Samayi/Bhadeli Kutki/Gundli/Salkaya	Oraon, Munda (Jharkhand)<br>Baiga (Madhya Pradesh) (Madhya Pradesh)	(Ghosh-Jerath et al., 2015b, 2021; Sebestianus Lakra, 2019)

Common name	Botanical name	Part consumed	Vernacular name	Accessed by indigenous community	Reference
58. Kodo millet	*Paspalum scrobiculatum* L.	Grain	*Varagu*	Savera, Jatapu, Gadabe and Kondadora (Andhra Pradesh)	(Rajyalakshmi and Geervani, 1994)
59. Japenese millet	*Echinochloa frumentacea* Lin k	Grain	*Sanwan/ Sanwa* (Baiga)	Baiga (Madhya Pradesh), Savera, Jatapu, Gadabe and Kondadora (Andhra Pradesh)	(Rajyalakshmi and Geervani, 1994; Sebestianus Lakra, 2019)
60-63. Finger millet	*Eleusine coracana* (L.) Gaertn.	Grain	*Tella/ Punasa/ Burada*	Savera, Jatapu, Gadabe and Kondadora (Andhra Pradesh)	(Rajyalakshmi and Geervani, 1994)
			Mandua/Kodde	Santhals, Sauria Paharia, Oraon (Jharkhand)	(Ghosh-Jerath et al., 2015b, 2016, 2020a)
			Naglano	Bhil (Gujarat)	(Bhattacharjee et al., 2009)
64. Cowpea, brown	*Vigna catjang* (L.) Walp.	Seed	*Ghangra/Dangudi*	Santhals, Sauria Paharia, Munda (Jharkhand)	(Ghosh-Jerath et al., 2016, 2020a, 2021)
			Chowli	Bhil (Gujarat)	(Bhattacharjee et al., 2009)
65. Cowpea, white	*Dolichos catjang* Burm.f	Seed	*Barbatti/Bodi*	Baiga (Madhya Pradesh), Munda, Sauria Paharia, Oraon (Jharkhand)	(Ghosh-Jerath et al., 2015b, 2020a, 2021; Sebestianus Lakra, 2019)
66. Hairy wetch	*Vicia hirsuta* (L.) Gray	Seed	*Baturi/Teeri reeti*	Santhals, Munda (Jharkhand)	(Ghosh-Jerath et al., 2016, 2021)
67-69. Horse gram	*Dolichos biflorus* L	Seed	*Kulthi*	Oraon,Munda,Santhal,Sauria Paharia (Jharkhand)	(Ghosh-Jerath et al., 2015b, 2016, 2020a, 2021)
			Kulthi (Black & white variety)	Savera, Jatapu, Gadabe and Kondadora (Andhra Pradesh)	(Rajyalakshmi and Geervani, 1994)
			Kulad	Bhil (Gujarat)	(Bhattacharjee et al., 2009)
70. Velvet Bean	*Mucuna pruriens* (L.) DC.	Seed	*Kusa*	Sauria Paharia (Jharkhand)	(Ghosh-Jerath et al., 2020a)
			Dukka chikkudu	Savera, Jatapu, Gadabe and Kondadora (Andhra Pradesh), Nicobari (Andaman & Nicobar Islands), Indigenous communities of Kerala	(Rajyalakshmi and Geervani, 1994; Vadivel and Janardhanan, 2005)
71. -	*Mucuna pruriens* var. *utilis* (Wall. ex Wight) L.H.Bailey	Seed	-	Indigenous communities of Kerala	(Vadivel and Janardhanan, 2005)
72. Black gram dal	*Phaseolus mungo* L	Seed	*Rambada /Urad dal*	Baiga (Madhya Pradesh), Munda (Jharkhand), Bhil (Gujarat)	(Bhattacharjee et al., 2009; Sebestianus Lakra, 2019; Ghosh-Jerath et al., 2021)
73. Grass pea	*Lathyrus sativus* L	Seed	*Khesari dal*	Baiga (Madhya Pradesh),Sauria Paharia (Jharkhand)	(Sebestianus Lakra, 2019; Ghosh-Jerath et al., 2020a)
74. Lentil	*Lens culinaris* Medik.	Seed	*Masoor*	Baiga (Madhya Pradesh), Bhil (Gujarat)	(Bhattacharjee et al., 2009; Sebestianus Lakra, 2019)
75. Kidney beans	*Phaseolus vulgaris* L.	Seed	*Rajma*	Baiga (Madhya Pradesh)	(Sebestianus Lakra, 2019)
76-78. Red gram	*Cajanus cajan* (L.) Millsp.	Seed	*Rahar/Rehad/Arhar*	Baiga (Madhya Pradesh)	(Sebestianus Lakra, 2019)
			Arhar (Dry land & hill cultivated)	Savera, Jatapu, Gadabe and Kondadora (Andhra Pradesh)	(Rajyalakshmi and Geervani, 1994)
			Tuver	Bhil (Gujarat)	(Bhattacharjee et al., 2009)
79-82. Field Bean	*Dolichos lablab* L.	Seed	-	Baiga (Madhya Pradesh)	(Sebestianus Lakra, 2019)
			Field bean (Black variety)	Savera, Jatapu, Gadabe and Kondadora (Andhra Pradesh)	(Rajyalakshmi and Geervani, 1994)

Common name	Botanical name	Part consumed	Vernacular name	Accessed by indigenous community	Reference
			Field bean (white variety)	Savera, Jatapu, Gadabe and Kondadora (Andhra Pradesh)	(Rajyalakshmi and Geervani, 1994)
			Field bean (red variety)	Savera, Jatapu, Gadabe and Kondadora (Andhra Pradesh)	(Rajyalakshmi and Geervani, 1994)
83. Black eyed-pea	*Vigna unguiculata* (L.) Walp.	Seed	*Judumulu*	Savera, Jatapu, Gadabe and Kondadora (Andhra Pradesh)	(Rajyalakshmi and Geervani, 1994)
84. Rice bean	Phaseolus calcaratus Roxb.	Seed	*Sutro/sutri*	Sauria Paharia,Munda,Santhal (Jharkhand)	(Ghosh-Jerath et al., 2016, 2020a, 2021)
85. *Mahul/Seldey*	*Bauhinia vahlii* Wight & Arn.	Seed	*Mahul/Seldey*	Gond, Saharia (Madhya Pradesh)	(Jain and Tiwari, 2012)
86. Prickly chaff flower	*Achyranthes aspera* L.	Seed	*Latjeera/Chirchita*	Gond, Saharia (Madhya Pradesh)	(Jain and Tiwari, 2012)
87. Common oak	*Quercus robur* L.	Nuts	*Soh ot*	Khasi (Meghalaya)	(Agrahar-Murugkar and Subbulakshmi, 2005b)
88. Chinquapin	*Castanopsis indica* (Roxb. ex Lindl.) A.DC.	Nuts	*Soh ot rit/Soh ot*	Khasi (Meghalaya)	(Agrahar-Murugkar and Subbulakshmi, 2005b; Chyne et al., 2019)
89. Nutgall	*Rhus chinensis* Mill.	Nuts	*Sohma*	Khasi (Meghalaya)	(Chyne et al., 2019)
90-91. Perilla	*Perilla frutescens* (L.) Britton	Nuts	*Nei lieh*	Khasi (Meghalaya)	(Chyne et al., 2019)
			Hanshi	Tribes of Manipur	(Longvah and Deosthale, 1991)
92. Sesame seeds (black)	*Sesamum indicum* L.	Nuts	*Nei iong*	Khasi (Meghalaya)	(Chyne et al., 2019)
93. Chalmogra	*Gynocardia odorata* R.Br.	Nuts	*Soh liang*	Khasi (Meghalaya)	(Chyne et al., 2019)
94. Nutgall	*Rhus chinensis* Mill.	Nuts	*Sohma*	Khasi (Meghalaya)	(Agrahar-Murugkar and Subbulakshmi, 2005b)
95. Job's tears	*Coix lacryma* var. *stenocarpa* Oliv.	Nuts	*Riew magain*	Khasi (Meghalaya)	(Agrahar-Murugkar and Subbulakshmi, 2005b)
96. Jack bean	*Canavalia ensiformis* (L.) DC.	Seed	-	Indigenous communities of Kerala	(Vadivel and Janardhanan, 2005)
97. Sword bean	*Canavalia gladiata* (Jacq.) DC.	Seed	*Badi sem*	Indigenous communities of Tamil Nadu	(Vadivel and Janardhanan, 2005)
98. -	*Cassia floribunda* Collad.	Seed	-	Indigenous communities of Karnataka	(Vadivel and Janardhanan, 2005)
99-100. Pot Casia	*Senna obtusifolia* (L.) H.S.Irwin & Barneby	Seed	-	Indigenous communities of Tamil Nadu	(Vadivel and Janardhanan, 2005)
		Leaves	*Chakod ara/Chakod/Kanyur aa*	Oraon, Munda, Ho (Jharkhand)	(Ghosh-Jerath et al., 2015b, 2021; Horo and Topno, 2015)
			Thakara	Kurichiyars, Adiyas, Kattunaikkan, Kuruman, Paniyas and Urali (Kerala)	(Pradeepkumar et al., 2015)
			Panwar	Gond, Saharia (Madhya Pradesh) (Madhya Pradesh)	(Jain and Tiwari, 2012)
101. -	*Mucuna monosperma* Wight	Seed		Indigenous communities of Karnataka	(Vadivel and Janardhanan, 2005)
102. Goose weed	Sphenoclea zeylanica Gaertn	Leaves	*Jheel-morich*	Bodo (Assam)	(Basumatary and Narzary, 2017)
103. Pepper weed	*Cardamine hirsuta* L	Leaves		Bodo (Assam)	(Basumatary and Narzary, 2017)

Common name	Botanical name	Part consumed	Vernacular name	Accessed by indigenous community	Reference
104. *Aso-pat/Ouput*	*Natsiatum herpeticum* Buch. -Ham. ex Arn.	Leaves	*Aso-pat/Ouput*	Bodo (Assam)	(Basumatary and Narzary, 2017)
105. -	*Sphaerantus peguensis* Kurtz ex C.B. Clark	Leaves		Bodo (Assam)	(Basumatary and Narzary, 2017)
106. Bankundri	*Melothria perpusilla* (Blume) Cogn.	Leaves		Bodo (Assam)	(Basumatary and Narzary, 2017)
107. Chinese Knotweed	*Persicaria chinensis* (L) H. Gross	Leaves	*Wnkham khalai*	Bodo (Assam)	(Basumatary and Narzary, 2017)
108. Malabar spinach	*Basella rubra* L.	Leaves	*Poi saag/ Pondka saag*	Adi (Arunachal Pradesh),Sauria Paharia (Jharkhand), Nicobari (Andaman & Nicobar Islands)	(Bhardwaj et al., 2009; Singh et al., 2018; Ghosh-Jerath et al., 2020a)
109. Basella leaves	*Basella alba* L.	Leaves	*Bon Pui Sak*	Lodha (West Bengal), Nicobari (Andaman & Nicobar Islands)	(Jana, 2004; Singh et al., 2018)
110. East Indian Glory Bower	*Clerodendrum colebrookianum* Walp.	Leaves	*Oen/ Ongin*	Adi (Arunachal Pradesh)	(Bhardwaj et al., 2009)
111. Crookneck pumpkin leaves	*Cucurbita moschata* Duchesne	Leaves	*Tapa*	Adi (Arunachal Pradesh)	(Bhardwaj et al., 2009)
112. Vegetable fern	*Diplazium esculentum* (Retz.) Sw.	Leaves	*Dhekia*	Adi (Arunachal Pradesh)	(Bhardwaj et al., 2009)
			Churuli	Kurichiyars, Adiyas, Kattunaikkan, Kuruman, Paniyas and Urali (Kerala)	(Pradeepkumar et al., 2015)
			-	Monpas, Memba and Khamba (Arunachal Pradesh)	(Tag et al., 2014)
			Tyrkhang	Khasi (Meghalaya)	(Agrahar-Murugkar, 2006)
				Nyishi (Arunachal Pradesh)	(Medak and Singha, 2018)
113. Hill Gynura	*Gynura cusimbua* (D.Don) S.Moore	Leaves	*Ogen*	Adi (Arunachal Pradesh)	(Bhardwaj et al., 2009)
114. *Gaam oying*	*Glochidion multiloculare* (Rottler ex Willd.) Voigt	Leaves	*Gaam oying*	Adi (Arunachal Pradesh)	(Bhardwaj et al., 2009)
115. East Himalayan Mussaenda	*Mussaenda roxburghii* Hook.f.	Leaves	*Aksap*	Adi (Arunachal Pradesh)	(Bhardwaj et al., 2009)
116.	Pouzolzia bennettiana	Leaves	*Oyik*	Adi (Arunachal Pradesh)	(Bhardwaj et al., 2009)
117. Mokha	*Schrebera swietenioides Roxb.*	Leaves	*Mokha*	Bhil (Gujarat)	(Bhattacharjee et al., 2009)
118. Terena leaves		Leaves	*Terena*	Bhil (Gujarat)	(Bhattacharjee et al., 2009)
119. Water celery	*Oenanthe javanica* (Blume) DC.	Leaves	*Komprek*	Monpas, Memba and Khamba (Arunachal Pradesh), Tribes of Manipur	(Tag et al., 2014; Loukrakpam et al., 2019)
120. Black Pig Weed leaves	*Trianthema portulacastrum* L.	Leaves	*Ohoi-arak/ Ohio ara /Naolo Ghasi*	Santhals, Sauria Paharia,Munda (Jharkhand)	(Ghosh-Jerath et al., 2015b, 2016, 2020a, 2021)
			Adachitkana	Indigenous communities of Karnataka	(Gupta et al., 2005)
121. Polpala/Kapurijadi	*Aerva lanata* (L.) Juss.	Leaves	*Lapongarak/ Lupu ara*	Santhals,Munda (Jharkhand)	(Ghosh-Jerath et al., 2016, 2021)
122-124. Colocasia		Leaves	*Saaru saag*	Oraon (Jharkhand)	(Ghosh-Jerath et al., 2015b)

Common name	Botanical name	Part consumed	Vernacular name	Accessed by indigenous community	Reference
	Colocasia esculenta (L.) Schott		*Vayalthaalu*	Kurichiyars, Adiyas, Kattunaikkan, Kuruman, Paniyas and Urali (Kerala)	(Pradeepkumar et al., 2015)
			Saaru saag/saru ara/saru-arak/Makedi Ghasi	Sauria Paharia, Santhals, Munda, Oraons (Jharkhand)	(Ghosh-Jerath et al., 2015b, 2016, 2020a, 2021)
		Tuber	-	Valiyans (Kerala)	(Mohan and Kalidass, 2010)
			Saaru/Toti	Munda (Jharkhand)	(Ghosh-Jerath et al., 2021)
			Ghuniya	Nicobari (Andaman & Nicobar Islands)	(Singh et al., 2018)
			Aloknala	Bhil (Gujarat)	(Bhattacharjee et al., 2009)
	Colocasia antiquorum Schott	Leaves	*Pechki aa*	Ho (Jharkhand)	(Horo and Topno, 2015)
			Karinthaalu	Kurichiyars, Adiyas, Kattunaikkan, Kuruman, Paniyas and Urali (Kerala)	(Pradeepkumar et al., 2015)
125. Chimti leaves	*Polygonum abbreviatum* Kom .	Leaves	*Chimti sag*	Oraon (Jharkhand)	(Ghosh-Jerath et al., 2015b)
126. Mountain ebony	Bauhinia variegata L.	Leaves	*Konar sag*	Oraon (Jharkhand)	(Ghosh-Jerath et al., 2015b)
127. Kachnar flower	Bauhinias variegata L.	Flower	*Kachna Phool/Burju Baha*	Sauria Paharia,Munda (Jharkhand)	(Ghosh-Jerath et al., 2020a, 2021)
128. Purple orchid tree/Koinaar leaves	*Bauhinia purpurea* L..	Leaves	*Komo Ghasi/Sing ara*	Sauria Paharia (Jharkhand)	(Ghosh-Jerath et al., 2020a)
			Sing aa	Ho (Jharkhand)	(Horo and Topno, 2015)
129. Garkha leaves	*Celosia argentea* L.	Leaves	*Siliary saag/Sirgiti-arak*	Oraon/Santhals (Jharkhand)	(Ghosh-Jerath et al., 2015b, 2016)
			Sinduar saag /Chilo ghasi	Sauria Paharia (Jharkhand)	(Ghosh-Jerath et al., 2020a)
			Sirgiti aa/ Siliary ara	Munda (Jharkhand)	(Ghosh-Jerath et al., 2021)
			Sirgiti aa	Ho (Jharkhand)	(Horo and Topno, 2015)
			Annae	Indigenous communities of Karnataka	(Gupta et al., 2005, 20)
130. Slender amaranth	*Amaranthus viridis* L.	Leaves	*Bhaji saag*	Oraon (Jharkhand)	(Ghosh-Jerath et al., 2015b)
			Bon Notey Sak	Lodha (West Bengal)	(Jana, 2004)
			Marsha bhaji	Nicobari (Andaman & Nicobar Islands)	(Singh et al., 2018)
			Jada saw	Khasi (Meghalaya)	(Agrahar-Murugkar, 2006)
			Chaulai	Gond, Saharia (Madhya Pradesh)	(Jain and Tiwari, 2012)
131. Red amaranth	*Amaranthus gangeticus* L.	Leaves	*Lal Bhaji*	Oraon (Jharkhand)	(Ghosh-Jerath et al., 2015b)
132. -	*Amaranthus gangeticus* Roxb	Leaves	*Leper aa*	Ho (Jharkhand)	(Horo and Topno, 2015)
133. Potato leaves	*Solanum tuberosum* L.	Leaves	*Aloo saag, Aloo Ghasi,Aloo arak, Aloo aa*	Oraon, Sauria Paharia,Munda, Ho (Jharkhand)	(Ghosh-Jerath et al., 2015b, 2016, 2020a, 2021; Horo and Topno, 2015)
134. Mata leaves	*Antidesma diandrum* (Roxb.) B.Heyne ex Roth	Leaves	*Mata sag/Matha-arak (lupu)*	Oraon, Ho (Jharkhand) Lodha (West Bengal)	(Ghosh-Jerath et al., 2015b, 2016)
			Tisso Ghasi	Sauria Paharia (Jharkhand)	(Ghosh-Jerath et al., 2020a)
			Ching yensil	Indigenous communities of Manipur	(Loukrakpam et al., 2019)
135. Katai leaves	*Meyna pubescens* (Kurz.) Robyns	Leaves	*Sarli saag/sarla ara/sarli aa*	Oraon,Munda,Ho (Jharkhand)	(Ghosh-Jerath et al., 2015b, 2016, 2021; Horo and Topno, 2015)
136. Kena leaves	*Commelina benghalensis* L.	Leaves	*Berbayo Ghasi*	Sauria Paharia (Jharkhand)	(Ghosh-Jerath et al., 2020a)
			Upundu ara	Munda (Jharkhand)	(Ghosh-Jerath et al., 2021)
			Bat pied	Khasi (Meghalaya)	(Agrahar-Murugkar, 2006)
			Kena	Indigenous communities of Maharashtra	(Mahadkar et al., 2012)

Common name	Botanical name	Part consumed	Vernacular name	Accessed by indigenous community	Reference
			Kanne	Indigenous communities of Karnataka	(Gupta et al., 2005)
137. Aradiyo leaves	NA	Leaves	*Aradiyo Ghasi*	Sauria Paharia (Jharkhand)	(Ghosh-Jerath et al., 2020a)
138. Sunsuni leaves	*Marsilea minuta* L.	Leaves	*Junjuni/ Susni ara/Chatom ara*	Sauria Paharia,Munda (Jharkhand)	(Ghosh-Jerath et al., 2020a)
			Sunsuniya saag/susni saag/Chatom aa	Oraon,Ho (Jharkhand)	(Ghosh-Jerath et al., 2015b; Horo and Topno, 2015)
139. *Mannadro*	NA	Leaves	*Mannadro*	Sauria Paharia (Jharkhand)	(Ghosh-Jerath et al., 2020a)
140. Khesari leaves	*Lathyrus sativus* L.	Leaves	*Khesari saag/Khesari ara*	Sauria Paharia (Jharkhand)	(Ghosh-Jerath et al., 2020a)
			Kansari aa	Ho (Jharkhand)	(Horo and Topno, 2015)
141. Gadhakand leaves	*Boerhavia diffusa* L.	Leaves	*Kecho aa*	Ho (Jharkhand)	(Horo and Topno, 2015)
			Punarnava	Gond, Saharia (Madhya Pradesh)	(Jain and Tiwari, 2012)
			Bagargunchi	Indigenous communities of Karnataka	(Gupta et al., 2005)
142. Khapra leaves	*Boerhavia procumbens* Banks ex Roxb	Leaves	*Kecho ara/Khapra saag*	Munda,Oraon (Jharkhand)	(Ghosh-Jerath et al., 2015b, 2021)
143. Wild mustard	*Cleome viscosa* L.	Leaves	*Hurhuria aa*	Ho (Jharkhand)	(Horo and Topno, 2015)
144. Asthma plant	*Euphorbia hirta* L.	Leaves	*Towa aa*	Ho (Jharkhand)	(Horo and Topno, 2015)
145. Common knotweed	*Polygonum plebeium* R.Br.	Leaves	*Mui aa*	Ho,Munda (Jharkhand)	(Horo and Topno, 2015; Ghosh-Jerath et al., 2021)
146. Chukra leaves	*Rumex vesicarius* L.	Leaves	*Tissa palak aa*	Ho (Jharkhand)	(Horo and Topno, 2015)
147. Drumstick leaves	*Moringa oleifera* Lam.	Leaves	*Sajne Sak*	Lodha (West Bengal)	(Jana, 2004)
			Sahjana	Nicobari (Andaman & Nicobar Islands)	(Singh et al., 2018)
			Muri ara/Munga ara/Sanjhodi ghasi/Munga saag/Munga-arak/Mulga aa	Sauria Paharia, Santhals, Munda, Oraons, Ho (Jharkhand)	(Ghosh-Jerath et al., 2015b, 2016, 2020a, 2021; Horo and Topno, 2015)
			Sehjana saag	Gond, Saharia (Madhya Pradesh)	(Jain and Tiwari, 2012)
148. Gogu leaves, red stem	*Hibiscus sabdariffa* L.	Leaves	*Ambad bhaji*	Banjara, Gond, Mana, Dhivar and Pardhi (Maharashtra)	(Laddha et al., 2015)
			Khatta Bhaji	Nicobari (Andaman & Nicobar Islands)	(Singh et al., 2018)
			Jarsong	Khasi (Meghalaya)	(Agrahar-Murugkar, 2006)
			Epil ara/ /kudrum	Munda (Jharkhand)	(Longvah et al., 2017)
149. -	*Brassaiopsis hainla* (Buch.-Ham.) Seem.	Leaves	*Lainong*	Rongmei (Manipur)	(Panmei et al., 2016)
150. Melinjo	*Gnetum gnemon* L.	Leaves	*Ganmakhen*	Rongmei (Manipur)	(Panmei et al., 2016)
151. Himalayan clearweed	*Pilea scripta* (Buch.-Ham. ex D. Don) Wedd.	Leaves	*Turingnong*	Rongmei (Manipur)	(Panmei et al., 2016)
152. *Gankarek*	*Rhynchotechum ellipticum* (W all. ex D. Dietr.) A. DC.	Leaves	*Gankarek*	Rongmei (Manipur)	(Panmei et al., 2016)
153. Dogal tree leaves	*Sarcochlamys pulcherrima* G audich.	Leaves	*Goibalei*	Rongmei (Manipur)	(Panmei et al., 2016)
154. Prickly Chaff Flower	*Achyranthes aspera* L.	Leaves	*Vankadalaadi*	Kurichiyars, Adiyas, Kattunaikkan, Kuruman, Paniyas and Urali (Kerala)	(Pradeepkumar et al., 2015)

Common name	Botanical name	Part consumed	Vernacular name	Accessed by indigenous community	Reference
155. Ponnaginni	*Alternanthera sessilis* (L.) R.Br. ex DC.	Leaves	*Ponnaamkanni*	Kurichiyars, Adiyas, Kattunaikkan, Kuruman, Paniyas and Urali (Kerala)	(Pradeepkumar et al., 2015)
			Garundi / Gundri ara/Garundi arak/Gundri saag/ Garundi aa	Sauria Paharia, Santhals, Munda, Oraons, Ho (Jharkhand)	(Ghosh-Jerath et al., 2015b, 2016, 2020a)
156. Amaranth spinosus, leaves, green	*Amaranthus spinosus* L.	Leaves	*Mullancheera, Mullukeera*	Kurichiyars, Adiyas, Kattunaikkan, Kuruman, Paniyas and Urali (Kerala)	(Pradeepkumar et al., 2015)
			Leped ara/ Gandhari-arak/Gandhari saag/Adro Ghasi/ Leper aa	Sauria Paharia, Santhals, Munda, Oraons, Ho (Jharkhand)	(Horo and Topno, 2015; Ghosh-Jerath et al., 2016, 2020a)
157. Matla leaves	NA	Leaves	*Matla Bhaji*	Bhil (Gujarat)	(Bhattacharjee et al., 2009)
158. Common Leucas	*Leucas aspera* (Willd.) Link.	Leaves	*Thumba*	Kurichiyars, Adiyas, Kattunaikkan, Kuruman, Paniyas and Urali (Kerala)	(Pradeepkumar et al., 2015)
159. -	*Momordica sahyadrica* Kattuk. and V.T.Antony	Leaves	*Kaattupaaval*	Kurichiyars, Adiyas, Kattunaikkan, Kuruman, Paniyas and Urali (Kerala)	(Pradeepkumar et al., 2015)
160. Black night shade	*Solanum nigrum* L.	Leaves	*Mudungachappu*	Kurichiyars, Adiyas, Kattunaikkan, Kuruman, Paniyas and Urali (Kerala)	(Pradeepkumar et al., 2015)
			How-ore	Adi and Nyishi (Arunachal Pradesh)	(Seal et al., 2016)
161. *Saambaarcheera*	*Talinum portulacifolium* (Forssk.) Asch. ex Schweinf.	Leaves	*Saambaarcheera*	Kurichiyars, Adiyas, Kattunaikkan, Kuruman, Paniyas and Urali (Kerala)	(Pradeepkumar et al., 2015)
162. Chinese Spinach	*Amaranthus tricolor* L	Leaves	-	Kurichiyars, Adiyas, Kattunaikkan, Kuruman, Paniyas and Urali (Kerala)	(Pradeepkumar et al., 2015)
			Marsha bhaji	Nicobari (Andaman & Nicobar Islands)	(Singh et al., 2018)
			Kilkeerae	Indigenous communities of Karnataka	(Gupta et al., 2005)
163. Curry leaves	*Murraya koenigii* (L.) Spreng.	Leaves	*Curry patta*	Nicobari (Andaman & Nicobar Islands)	(Singh et al., 2018, 201)
164. Agathi leaves	*Sesbania grandiflora* (L.) Pers.	Leaves	*Agathi*	Nicobari (Andaman & Nicobar Islands)	(Singh et al., 2018)
165. Alligator weed	*Alternanthera philoxeroides* (Mart.) Griseb.	Leaves	*Madrasi Bhaji*	Nicobari (Andaman & Nicobar Islands)	(Singh et al., 2018)
			Ong put	Khasi (Meghalaya)	(Agrahar-Murugkar, 2006)
166. Purple amaranth	*Amaranthus lividus* L.	Leaves	*Marsha bhaji*	Nicobari (Andaman & Nicobar Islands)	(Singh et al., 2018)
167. Water hyssop/Brahmi	*Bacopa monnieri* (L.) Wettst.	Leaves	*Brahmi*	Nicobari (Andaman & Nicobar Islands)	(Singh et al., 2018)
			Dali aa	Ho (Jharkhand)	(Horo and Topno, 2015)
168. White jute	*Corchorus capsularis* L.	Leaves	*Patt*	Nicobari (Andaman & Nicobar Islands)	(Singh et al., 2018)
169. Male fern	*Dryopteris filix-mas* (L.) Schott.	Leaves	*Deki Bhaji*	Nicobari (Andaman & Nicobar Islands)	(Singh et al., 2018)
170. Helencho	*Enhydra fluctuans* Lour.	Leaves	*Helencho*	Nicobari (Andaman & Nicobar Islands)	(Singh et al., 2018)
			Hirmichiya saag	Oraon (Jharkhand)	(Ghosh-Jerath et al., 2015b)
171. Wild coriander/ Curantro	*Eryngium foetidum* L.	Leaves	*Burma Dhaniya*	Nicobari (Andaman & Nicobar Islands)	(Singh et al., 2018)
			Duhania Khlaw/Dhaniya khasi	Khasi (Meghalaya)	(Agrahar-Murugkar, 2006; Chyne et al., 2019)
172. Kulekhara	*Hygrophila auriculata* (Schu mach.) Heine	Leaves	*Kulekhara*	Nicobari (Andaman & Nicobar Islands)	(Singh et al., 2018)

Common name	Botanical name	Part consumed	Vernacular name	Accessed by indigenous community	Reference
173. Water primrose	*Jussiaea repens* L.	Leaves	*Malencho*	Nicobari (Andaman & Nicobar Islands)	(Singh et al., 2018)
174. Wild Betel	*Piper sarmentosum* Roxb.	Leaves	*Pipali sag*	Nicobari (Andaman & Nicobar Islands)	(Singh et al., 2018)
175. Star Gooseberry	*Sauropus androgynus* (L.) Merr.	Leaves	*Chakurmani*	Nicobari (Andaman & Nicobar Islands)	(Singh et al., 2018)
176. -	*Tragia lassia* Radcl.-Sm. & Govaerts	Leaves	-	Karbi (Assam)	(Terangpi and Teron, 2015)
177. -	*Premna latifolia* Roxb.	Leaves	-	Karbi (Assam)	(Terangpi and Teron, 2015)
178. Kantha leaves	*Euphorbia granulate* Forssk.	Leaves	*Daav ghasi/Kantha-arak*	Sauria Paharia, Santhals (Jharkhand)	(Ghosh-Jerath et al., 2016, 2020a)
179. Bottle gourd leaves	*Lagenaria siceraria* (Molina) Standl.	Leaves	*Kaddu ara/Kaddu arak/Lol ghasi*	Sauria Paharia, Santhals, Munda (Jharkhand)	(Ghosh-Jerath et al., 2016, 2020a, 2021)
180. Dhurup leaves	*Leucas lavandulifolia* Sm.	Leaves	*Khadia ara/Kondi ghasi/Dhurup-arak*	Sauria Paharia, Santhals, Munda (Jharkhand)	(Ghosh-Jerath et al., 2016, 2020a, 2021)
181. Banyan leaves	*Ficus benghalensis* L.	Leaves	*Hesa ara/Hesak-arak/Pakkedi Ghasi*	Sauria Paharia, Santhals, Munda (Jharkhand)	(Ghosh-Jerath et al., 2016, 2020a, 2021)
182. Bengal gram leaves	*Cicer arietinum* L.	Leaves	*Boot ara/Boot ghasi/Chana saag/But-arak/ But aa*	Sauria Paharia, Santhals, Munda, Ho (Jharkhand)	(Horo and Topno, 2015; Ghosh-Jerath et al., 2016, 2020a, 2021)
		Leaves	*Chana bhaji*	Bhil (Gujarat)	(Bhattacharjee et al., 2009)
183. Garlic leaves	*Allium sativum* L.	Leaves	*Lahsun saag/Nasni Ghasi*	Sauria Paharia, Santhals, Munda, Oraons (Jharkhand)	(Ghosh-Jerath et al., 2015b, 2016, 2020a; Horo and Topno, 2015)
184. Field mustard	*Brassica campestris* L.	Leaves	*Chiniya saag*	Sauria Paharia, Santhals, Oraons (Jharkhand)	(Ghosh-Jerath et al., 2015b, 2016, 2020a)
185. Mustad leaves	Brassica *juncea* (L.) Czern.	Leaves	*Lotni saag*	Oraon (Jharkhand)	(Ghosh-Jerath et al., 2015b)
186. Tamarind leaves	*Tamarindus indica* L.	Leaves	*Jojo-ara*	Munda (Jharkhand)	(Ghosh-Jerath et al., 2021)
187. Water spinach	*Ipomoea aquatica* Forssk. [€]	Leaves	*Kalmi ara*	Munda (Jharkhand)	(Ghosh-Jerath et al., 2021)
		Leaves	*Nalli Bhaji*	Nicobari (Andaman & Nicobar Islands)	(Singh et al., 2018)
188. Beng leaves	*Centella asiatica (L.)* Urb.	Leaves	*Beng saag/ Chokke ara*	Munda,Oraon (Jharkhand)	(Ghosh-Jerath et al., 2015b)
			Muthil	Kurichiyars, Adiyas, Kattunaikkan, Kuruman, Paniyas and Urali (Kerala)	(Pradeepkumar et al., 2015)
			Medak bhaji	Nicobari (Andaman & Nicobar Islands)	(Singh et al., 2018)
			Khlien syiar	Khasi (Meghalaya)	(Agrahar-Murugkar, 2006)
			Brahmi	Indigenous communities of Karnataka	(Gupta et al., 2005)
189. Amaranth,tender, red leaves	*Amaranthus retroflexus* L.	Leaves	*Lal Bhaji/Lal saag*	Munda (Jharkhand)	(Ghosh-Jerath et al., 2021)
190. Phutkal leaves (dried)	*Ficus virens Aiton*	Leaves	*Phutkal ara/Phutkal saag*	Munda,Oraon (Jharkhand)	(Ghosh-Jerath et al., 2015b, 2021)
191. Pumpkin leaves	*Cucurbita maxima* L.	Leaves	*Kakaru ara*	Munda (Jharkhand)	(Ghosh-Jerath et al., 2021)
			-	Indigenous communities of Karnataka	(Gupta et al., 2005)
192. Nunia leaves	*Portulaca quadrifida* L.	Leaves	*Dali/Dail ara*	Munda (Jharkhand)	(Ghosh-Jerath et al., 2021)
193. Dheniani	*Olax scandens* Roxb.	Leaves	*Soredhe/ Bir/Rimil arak* [¥]	Munda (Jharkhand)	(Ghosh-Jerath et al., 2021)
194. Khatta saag	*Cissus auriculata* Roxb.	Leaves	*Budilaie arak*	Munda (Jharkhand)	(Ghosh-Jerath et al., 2021)

Common name	Botanical name	Part consumed	Vernacular name	Accessed by indigenous community	Reference
195. Kauwa leaves	*Rungia quinqueangularis* Koen.	Leaves	*Kauwa saag*	Munda (Jharkhand)	(Ghosh-Jerath et al., 2021)
196. Purslane	*Portulaca oleracea* L.	Leaves	*Uri le ara*	Munda (Jharkhand)	(Ghosh-Jerath et al., 2021)
			Kozhuppacheera	Kurichiyars, Adiyas, Kattunaikkan, Kuruman, Paniyas and Urali (Kerala)	(Pradeepkumar et al., 2015)
			Nuna Bhaji	Nicobari (Andaman & Nicobar Islands)	(Singh et al., 2018)
197. Netho leaves	*Medicago lupulina* Linn.	Leaves	*Piring ara*	Munda (Jharkhand)	(Ghosh-Jerath et al., 2021)
198. Arrow head	*Sagittaria latifolia* L.	Leaves	*Tir ara/ Lochkor ara*	Munda (Jharkhand)	(Ghosh-Jerath et al., 2021)
199. Sweet potato leaves	*Ipomoea batatas (L.)* Lam.	Leaves	*Sanga ara/Kanda saag*	Munda,Oraon (Jharkhand)	(Ghosh-Jerath et al., 2015b)
200. Patsan	*Hibiscus cannabinus* L.	Leaves	*Kotle ara*	Munda (Jharkhand)	(Ghosh-Jerath et al., 2021)
201. Ash gourd leaves	*Benincasa hispida* (Thunb.) Cogn.	Leaves	*Kohna/ Ketha ara*	Munda (Jharkhand)	(Ghosh-Jerath et al., 2021)
202. Kharika leaves	*Spergula pentandra* L.	Leaves	*Chaari ara*	Munda (Jharkhand)	(Ghosh-Jerath et al., 2021)
203. Akra	*Vicia sativa* L.	Leaves	*Chiringid arak*	Munda (Jharkhand)	(Ghosh-Jerath et al., 2021)
204. Amrit sak	*Oxalis corniculata* L.	Leaves	*Bir chhatom ara*	Munda (Jharkhand)	(Ghosh-Jerath et al., 2021)
			Kynbat Dkhiew	Khasi (Meghalaya)	(Agrahar-Murugkar, 2006)
			Khatti buti	Gond, Saharia (Madhya Pradesh)	(Jain and Tiwari, 2012)
205. Himayalan mayflower	*Maianthemum purpureum* (W all.) LaFrankie	Leaves		Monpas, Memba and Khamba (Arunachal Pradesh)	(Tag et al., 2014)
206. Chives	*Allium schoenoprasum* L.	Leaves	*Jaut*	Khasi (Meghalaya)	(Agrahar-Murugkar, 2006)
207. Milk Weed	*Sonchus oleraceus* (L.) L.	Leaves	*Jalyngiar*	Khasi (Meghalaya)	(Agrahar-Murugkar, 2006)
208. Broadleaf Plantain	*Plantago major*	Leaves		Khasi (Meghalaya)	(Agrahar-Murugkar, 2006)
209-210. Fish mint	*Houttuynia cordata* Thunb.	Leaves	*Jamyrdoh*	Khasi (Meghalaya)	(Agrahar-Murugkar, 2006; Chyne et al., 2019)
			-	Aka, Minji, Monpa, Sherdukpen and Bugun (Arunachal Pradesh)	(Saha et al., 2014)
		Root	*Jamyrdoh*	Khasi (Meghalaya)	(Seal, 2011)
211. *Jarain*	*Fagopyrum acutatum* (Lehm.) Mansf. ex K.Hammer	Leaves	*Jarain*	Khasi (Meghalaya)	(Agrahar-Murugkar, 2006)
212. Perennial sow thistle	*Sonchus arvensis* L.	Leaves	*Jakhain/ Jalynniar*	Khasi (Meghalaya)	(Agrahar-Murugkar, 2006; Seal, 2011; Chyne et al., 2019)
213. Cat's ear	*Hypochaeris radicata*	Leaves	*Jakhain*	Khasi (Meghalaya)	(Chyne et al., 2019)
214. *Jatira*	*Corydalis sibirica* (L.f.) Pers.	Leaves	*Jatira*	Khasi (Meghalaya)	(Agrahar-Murugkar, 2006)
	Oenanthe linearis Wall. ex DC.			Khasi (Meghalaya)	(Seal, 2011)
215. Wild leek	*Allium ampeloprasum* L.	Leaves	*Jyllan*	Khasi (Meghalaya)	(Agrahar-Murugkar, 2006)
216. Jangew	NA	Leaves	*Jangew*	Khasi (Meghalaya)	(Agrahar-Murugkar, 2006)
217. *Mahong*	*Spilanthes acmella* (L.) L.	Leaves	*Mahong*	Khasi (Meghalaya)	(Agrahar-Murugkar, 2006)

Common name	Botanical name	Part consumed	Vernacular name	Accessed by indigenous community	Reference
218. *Bat saw*	*Polygonum alatum* Buch.-Ham. ex D. Don	Leaves	*Bat saw*	Khasi (Meghalaya)	(Agrahar-Murugkar, 2006)
219. Hirankhuri leaves	*Emilia sonchifolia* (L.) DC. ex DC.	Leaves	*Jalang shor*	Khasi (Meghalaya)	(Agrahar-Murugkar, 2006)
220. *Jalong*	*Vernonia altissimifolia*	Leaves	*Jalong*	Khasi (Meghalaya)	(Agrahar-Murugkar, 2006)
221. East Himalayan Begonia	*Begonia roxburghii* A.DC	Leaves	*Dieng jajew*	Khasi (Meghalaya)	(Agrahar-Murugkar, 2006)
222. *Thylleij masi*	*Ficus cunea*	Leaves	*Thylleij masi*	Khasi (Meghalaya)	(Agrahar-Murugkar, 2006)
223. Jajew maw	*Begonia rubrovenia* Hook.	Leaves	*Jajew maw*	Khasi (Meghalaya)	(Agrahar-Murugkar, 2006)
224. Trysim khlieng	*Commelina diffusa* Burm.f.	Leaves	*Trysim khlieng*	Khasi (Meghalaya)	(Agrahar-Murugkar, 2006)
225. Elephant apple	*Dillenia indica* L.	Leaves	*Jamahek*	Khasi (Meghalaya)	(Agrahar-Murugkar, 2006)
226. Watercress	*Nasturtium officinale* R.Br.	Leaves	*Jhur kteih*	Khasi (Meghalaya)	(Agrahar-Murugkar, 2006)
227. Lobed Leaf Knotweed	*Polygonum runcinatum* Buch. -Ham. ex D. Don	Leaves	*Rungri*	Nyishi (Arunachal Pradesh)	(Medak and Singha, 2016, 2018)
228. Gungi	*Pilea bracteosa* Wedd.	Leaves	*Gungi*	Nyishi (Arunachal Pradesh)	(Medak and Singha, 2016, 2018)
229. Huj	*Elatostema platyphyllum* Wed d.	Leaves	*Huj*	Nyishi (Arunachal Pradesh)	(Medak and Singha, 2016, 2018)
230. Edible Gynura	*Gynura bicolor* (Roxb. ex Willd.) DC.	Leaves	-	Nyishi (Arunachal Pradesh)	(Medak and Singha, 2018)
231. -	*Plantago asiatica* subsp. *eros a* (Wall.) Z.Yu Li	Leaves	-	Nyishi (Arunachal Pradesh)	(Medak and Singha, 2018)
232. Pokeweed	*Phytolacca acinosa Roxb.*	Leaves	*Papok*	Adi and Nyishi (Arunachal Pradesh)	(Seal et al., 2016)
233. Raro	*Piper pedicellatum* C. DC.	Leaves	*Raro*	Adi and Nyishi (Arunachal Pradesh)	(Seal et al., 2016)
234. 'Huaig	*Pouzolzia hirta* Blume ex Hassk.	Leaves	*'Huaig*	Adi and Nyishi (Arunachal Pradesh)	(Seal et al., 2016)
235. Chopchini	*Smilax zeylanica* L	Leaves	*Chopchini*	Indigenous communities of Maharashtra	(Mahadkar et al., 2012)
236. Kokam	*Garcinia indica* (Thouars) Choisy	Leaves	*Kokam*	Indigenous communities of Maharashtra	(Mahadkar et al., 2012)
237. Balae	*Polygala erioptera* DC.	Leaves	*Balae*	Indigenous communities of Karnataka	(Gupta et al., 2005)
238. Mexican mint	*Plectranthus amboinicus* (Lou r.) Spreng.	Leaves	*Doddipatre*	Indigenous communities of Karnataka	(Gupta et al., 2005)
239. Lesua	*Digera muricata* (L.) Mart.	Leaves	*Gurchi*	Indigenous communities of Karnataka	(Gupta et al., 2005)
240. *Javanada*	*Cocculus hirsutus* (L.) W.Theob.	Leaves	*Javanada*	Indigenous communities of Karnataka	(Gupta et al., 2005)
241. Gandhuli	*Cleome gynandra* L.	Leaves	*Naribalae*	Indigenous communities of Karnataka	(Gupta et al., 2005)
242. Yellow Gulmohar leaves	*Delonix elata* (L.) Gamble	Leaves	*Vayunarayani*	Indigenous communities of Karnataka	(Gupta et al., 2005)

Common name	Botanical name	Part consumed	Vernacular name	Accessed by indigenous community	Reference
243. Jarem/ Phuinam	*Clerodendrum glandulosum* L indl.	Leaves	*Jarem/ Phuinam*	Khasi (Meghalaya)	(Seal, 2011)
244. Jaiur	*Zanthoxylum acanthopodium* DC.	Leaves	*Jaiur*	Khasi (Meghalaya)	(Seal, 2011)
245. Greenbriers	*Smilax elegans* Wall. ex Kunth	Leaves	*Sla sohkrot*	Khasi (Meghalaya)	(Chyne et al., 2019)
246. Giant taro leaves	Alocasia macrorrhizos (L.) G.Don	Leaves	*Sla wang bam im*	Khasi (Meghalaya)	(Chyne et al., 2019)
247. *Jathang*	Neilla thyrsiflora	Leaves	*Jathang*	Khasi (Meghalaya)	(Chyne et al., 2019)
248. Hooker chives	*Allium hookeri* Thwaites	Leaves	*Napakpi*	Indigenous communities of Manipur	(Loukrakpam et al., 2019)
249. Water mimosa	*Neptunia oleracea* Lour.	Leaves	*Ekaithabi*	Indigenous communities of Manipur	(Loukrakpam et al., 2019)
250. Malabar nut leaves	*Justicia adhatoda* L.	Leaves	*Nongmakha mapal*	Indigenous communities of Manipur	(Loukrakpam et al., 2019)
251. *Yelang*	*Persicaria barbata* (L.) H.Hara	Leaves	*Yelang*	Indigenous communities of Manipur	(Loukrakpam et al., 2019)
252. Chickweed	*Stellaria media* (L.) Vill.	Leaves	*Yerum kairum*	Indigenous communities of Manipur	(Loukrakpam et al., 2019)
253. *Fakpai*	*Polygonum posumbu* Buch.-Ham. ex D. Don	Leaves	*Fakpai*	Indigenous communities of Manipur	(Loukrakpam et al., 2019)
254. Garlic chives	*Allium tuberosum* Rottler ex Spreng.	Leaves	*Nakuppi*	Indigenous communities of Manipur	(Loukrakpam et al., 2019)
255. Phunil	*Anaphalis subdecurrens* (DC.) Gamble	Leaves	*Phunil*	Indigenous communities of Manipur	(Loukrakpam et al., 2019)
256. Kengoi	*Persicaria posumba*	Leaves	*Kengoi*	Indigenous communities of Manipur	(Loukrakpam et al., 2019)
257. Mahua seeds	*Bassia latifolia Roxb.*	Seeds	*Doli mahuda seeds*	Bhil (Gujarat)	(Bhattacharjee et al., 2009)
258. Cowpea	*Vigna catjang* (L.) Walp.	Vegetable	*Bada ghangra/Barbatti/Bodi*	Santhals, Sauria Paharia (Jharkhand)	(Ghosh-Jerath et al., 2016, 2020a)
259-260. Wild Brinjal	*Solanum torvum* Sw.	Unripe fruit	*Koppi*	Adi (Arunachal Pradesh)	(Bhardwaj et al., 2009)
	Solanum Khasi (Meghalaya)anum C.B. Clarke		*Koppir*	Adi (Arunachal Pradesh)	(Bhardwaj et al., 2009)
261. Pindra/Pinra	*Flacourtia indica* (Burm.f.) Merr.	Unripe fruit	*Pindra/Pinra*	Sauria Paharia (Jharkhand)	(Ghosh-Jerath et al., 2020a)
262. Ber alli	*Dioscorea* spp.	Unripe fruit	*Ber alli*	Sauria Paharia (Jharkhand)	(Ghosh-Jerath et al., 2020a)
263. Kachnar flower	Bauhinias variegata L.	Flower	*Kachna Phool/Burju Baha*	Sauria Paharia,Munda (Jharkhand)	(Ghosh-Jerath et al., 2020a)
264. Sanai Flower	*Crotalaria juncea* L.	Flower	*Sonpu Phool/Jiri Ba*	Sauria Paharia,Munda (Jharkhand)	(Ghosh-Jerath et al., 2020a)
265. Drumstick flower	*Moringa oleifera* Lam	Flower	*Sanjhori Phool*	Sauria Paharia (Jharkhand)	(Ghosh-Jerath et al., 2020a)
		Flower	*Sehjana Phool*	Gond, Saharia (Madhya Pradesh)	(Jain and Tiwari, 2012)
266. Kattian/Kasai	*Bridelia retusa* (L.) A.Juss.	Unripe fruit	*Bon Chalta*	Lodha (West Bengal)	(Jana, 2004)
267. Marine seaweed	*Prasiola crispa* f.	Unripe fruit	-	Aka, Bugun, Miji, Monpa and Sherdukpen (Arunachal Pradesh)	(Saha et al., 2014)

Common name	Botanical name	Part consumed	Vernacular name	Accessed by indigenous community	Reference
268. -	*Wallichia disticha* T.Anderso n	Unripe fruit	-	Aka, Bugun, Miji, Monpa and Sherdukpen (Arunachal Pradesh)	(Saha et al., 2014)
269. Bitter gourd	*Momordica charantia* L..	Unripe fruit	*Bir Karela/Jungli Karela*	Munda, Sauria Paharia, Oraon, Santhals (Jharkhand)	(Ghosh-Jerath et al., 2015b, 2016, 2020a)
			Bon Kankrol	Lodha (West Bengal)	(Jana, 2004)
270. Ridge gourd	*Luffa acutangula* (L.) Roxb..	Unripe fruit	*Jhinga*	Sauria Paharia (Jharkhand)	(Ghosh-Jerath et al., 2020a)
			Dodo/ Doro	Munda (Jharkhand)	(Ghosh-Jerath et al., 2021)
271. Bamboo	*Bambusa vulgaris* Schrad. ex J.C. Wendl.	Tender shoots	*Maas adro/Karu/Bans*	Munda, Sauria Paharia, Nicobari (Andaman & Nicobar Islands)	(Singh et al., 2018; Ghosh-Jerath et al., 2020a, 2021)
272. Ash gourd	*Benincasa hispida* (Thunb.) Cogn.	Unripe fruit	*Ketha/Zarkunda*	Munda, Sauria Paharia (Jharkhand)	(Ghosh-Jerath et al., 2020a, 2021)
273. Spine gourd	*Momordica diocia* Roxb ex Willd	Unripe fruit	*Kokri*	Sauria Paharia (Jharkhand)	(Ghosh-Jerath et al., 2020a)
			-	Valiyans (Kerala)	(Mohan and Kalidass, 2010)
274. Kovai	*Coccinia grandis* (L.) Voigt	Unripe fruit	*Kundri/Kundur*	Munda, Sauria Paharia (Jharkhand)	(Ghosh-Jerath et al., 2020a, 2021)
			Bon Kudri	Lodha (West Bengal)	(Jana, 2004)
			Kundru	Nicobari (Andaman & Nicobar Islands)	(Singh et al., 2018)
275. Turkey Berry	*Solanum torvum Swartz.*	Unripe fruit	*Kutumba/ Hanjen*	Munda (Jharkhand)	(Ghosh-Jerath et al., 2021)
276. -	*Caralluma adscendens* var. *at tenuata* (Wight) Grav. & Mayur.	Unripe fruit	-	Valiyans (Kerala)	(Mohan and Kalidass, 2010)
277. -	*Caralluma pauciflora* (Wight) N.E.Br.	Unripe fruit	-	Valiyans (Kerala)	(Mohan and Kalidass, 2010)
278. Indian shot	Canna indica L.	Unripe fruit	-	Valiyans (Kerala)	(Mohan and Kalidass, 2010)
279. Jackfruit	*Artocarpus heterophyllus* La m.	Unripe fruit	*Katahal*	Nicobari (Andaman & Nicobar Islands)	(Singh et al., 2018)
280. Breadfruit	*Artocarpus altilis* (Parkinson ex F.A.Zorn) Fosberg	Unripe fruit	*Bilayati katahal*	Nicobari (Andaman & Nicobar Islands)	(Singh et al., 2018)
281. Plantain, flower	*Musa × paradisiaca* L.	Flower	*Tarkari kela*	Nicobari (Andaman & Nicobar Islands)	(Singh et al., 2018)
282-283. Spiny bitter gourd	*Momordica cochinchinensis* (Lour.) Spreng	Unripe fruit	*Jangli kakrol*	Nicobari (Andaman & Nicobar Islands)	(Singh et al., 2018)
	Momordica subangulata subs p. *renigera* (Wall. ex G.Don) W.J.de Wilde		*Kakrol*	Nicobari (Andaman & Nicobar Islands)	(Singh et al., 2018)
284. Amaltas flower	*Cassia fistula* L.	Unripe fruit	*Amaltas Phool*	Gond, Saharia (Madhya Pradesh)	(Jain and Tiwari, 2012)
285. Ghaf	*Prosopis cineraria* (L.) Druce	Bark	*Shami, Kshenkar*	Gond, Saharia (Madhya Pradesh)	(Jain and Tiwari, 2012)
286. Wild plantain, flower	*Ensete Superbum* (Roxb.) Cheesuran	Flower	*Ran-keli, Chaveli-keli*	Indigenous communities of Maharashtra	(Mahadkar et al., 2012)
287. Dhawal	*Woodfordia fruticosa* (L.) Kurz	Unripe fruit	*Dhayati*	Indigenous communities of Maharashtra	(Mahadkar et al., 2012)
288. Hyacinth bean	*Lablab purpureus* (L.)	Unripe fruit	*Sem/Simbi/Manal*	Munda, Sauria Paharia, Oraon, Santhals (Jharkhand)	(Ghosh-Jerath et al., 2015b, 2016, 2020a, 2021)

Common name	Botanical name	Part consumed	Vernacular name	Accessed by indigenous community	Reference
289. Hyacint beans (red)			*Ri Saw*	Indigenous communities of Manipur	(Chyne et al., 2019)
290. Hyacint beans (curve, green)			*RiKdor*	Indigenous communities of Manipur	(Chyne et al., 2019)
291. Hyacint beans (green)			*RiJyrngam*	Indigenous communities of Manipur	(Chyne et al., 2019)
292. Bitter tomato	*Solanum aethiopicum* L.	Unripe fruit	*Sohngang heh/ Soh-ngang*	Indigenous communities of Manipur	(Chyne et al., 2019)
293. Wild banana stem	*Musa acuminata* Colla	Unripe fruit	*Nudkait*	Indigenous communities of Manipur	(Chyne et al., 2019)
294. Tree tomato	*Cyphomandra betacea*	Unripe fruit	*Soh baingon dieng*	Indigenous communities of Manipur	(Chyne et al., 2019)
295-296. Hairy-fruited eggplant	*Solanum lasiocarpum* Dunal	Unripe fruit	*Soh ngang rit*	Indigenous communities of Manipur	(Chyne et al., 2019)
	Solanum indicum L.	Unripe fruit	*Sohngang rit*	Khasi (Meghalaya)	(Agrahar-Murugkar and Subbulakshmi, 2005a)
297. Thai eggplant	*Solanum virginianum* L	Unripe fruit	*Sohthang*	Khasi (Meghalaya)	(Agrahar-Murugkar and Subbulakshmi, 2005a)
298. -	*Dendrocalamus hamiltonii* Nees & Arn. ex Munro	Unripe fruit	*Soidon*	Indigenous communities of Manipur	(Loukrakpam et al., 2019)
299. Wild bean	*Canavalia cathartica* Thouars	Unripe fruit	*Tebi*	Indigenous communities of Manipur	(Loukrakpam et al., 2019)
300. Fox nut	*Euryale ferox* Salisb.	Unripe fruit	*Thangjing*	Indigenous communities of Manipur	(Loukrakpam et al., 2019)
301. Broad bean	*Vicia faba* L.	Unripe fruit	*Hawaimubi*	Indigenous communities of Manipur	(Loukrakpam et al., 2019)
302. Tree bean	*Parkia timoriana* (DC.) Merr.	Unripe fruit	*Yongchak*	Indigenous communities of Manipur	(Loukrakpam et al., 2019)
303. *Feija*	*Wendlandia glabrata* DC.	Unripe fruit	*Feija*	Indigenous communities of Manipur	(Loukrakpam et al., 2019)
304. Mushroom, dry	*Agaricus bisporus*	Mushroom	*Kukkagodugu*	Savera, Jatapu, Gadabe and Kondadora (Andhra Pradesh)	(Bhattacharjee et al., 2009)
305-308.	*Amanita* sp.1,2,3,4	Mushroom		Lodha and Santhals (West Bengal), Aka, Bugun, Miji, Monpa and Sherdukpen (Arunachal Pradesh)	(Saha et al., 2014; Das et al., 2015)
309. -	*Astraeus* sp.	Mushroom		Lodha and Santhals (West Bengal)	(Das et al., 2015)
310-312. -	*Termitomyces* sp.1,2,3	Mushroom		Lodha and Santhals (West Bengal)	(Das et al., 2015)
313.	*Volvariella* sp	Mushroom		Lodha and Santhals (West Bengal)	(Das et al., 2015)
314-315. *Bali Chhatu*	*Agaricus* sp.1, 2	Mushroom	*Bali Chhatu*	Lodha and Santhals (West Bengal)	(Jana, 2004; Das et al., 2015)
316. -	*Calvatia* sp	Mushroom		Lodha and Santhals (West Bengal)	(Das et al., 2015)
317. -	*Lentinus* sp	Mushroom		Lodha and Santhals (West Bengal)	(Das et al., 2015)
318-321. -	*Russula* sp. 1,2,3,4	Mushroom		Lodha and Santhals (West Bengal)	(Das et al., 2015)
322. -	*Marasmius* sp	Mushroom	*Putca Chhatu*	Lodha (West Bengal)	(Jana, 2004)
323. Field mushroom	*Agaricus campestris*	Mushroom	*Parab Chhatu*	Lodha (West Bengal)	(Jana, 2004)
324. Branched Collybia	*Agaricus racemosus*	Mushroom	*Patra Chhatu*	Lodha (West Bengal)	(Jana, 2004)
325. -	*Psalliota* sp	Mushroom	*Mura Chhatu*	Lodha (West Bengal)	(Jana, 2004)
326. Puffball mushroom	*Calvatia gigantean*	Mushroom		Khasi (Meghalaya)	(Agrahar-Murugkar and Subbulakshmi, 2005a)
327. Gray coral	*Clavulina cinerea*	Mushroom		Khasi (Meghalaya)	(Agrahar-Murugkar and Subbulakshmi, 2005a)

Common name	Botanical name	Part consumed	Vernacular name	Accessed by indigenous community	Reference
328. Girolle	*Cantharellus cibarius*	Mushroom		Khasi (Meghalaya)	(Agrahar-Murugkar and Subbulakshmi, 2005a)
329. -	*Ramaria brevispora* Corner, K.S. Thind & Dev	Mushroom		Khasi (Meghalaya)	(Agrahar-Murugkar and Subbulakshmi, 2005a)
330. Russula	*Russula integra*	Mushroom		Khasi (Meghalaya)	(Agrahar-Murugkar and Subbulakshmi, 2005a)
331. Woolly chanterelle	*Gomphus floccosus*	Mushroom		Khasi (Meghalaya)	(Agrahar-Murugkar and Subbulakshmi, 2005a)
332. -	*Lactarius quieticolor* Romagn.	Mushroom		Khasi (Meghalaya)	(Agrahar-Murugkar and Subbulakshmi, 2005a)
333. Rugda	*Geastrum* sp.	Mushroom	*Rugda*	Munda (Jharkhand)	(Ghosh-Jerath et al., 2021)
334. Gitilud	NA	Mushroom	*Gitilud*	Munda (Jharkhand)	(Ghosh-Jerath et al., 2021)
335. Indiud	*Termitomyces albuminosa*	Mushroom	*Indiud*	Munda (Jharkhand)	(Ghosh-Jerath et al., 2021)
336. White rot fungus	*Termitomyces clypeatus*	Mushroom	*Koodeud/ Kundaud*	Munda (Jharkhand)	(Ghosh-Jerath et al., 2021)
337. -	*Pleurotus sajor-caju* (Fr.) Singer	Mushroom		Aka, Bugun, Miji, Monpa and Sherdukpen (Arunachal Pradesh)	(Saha et al., 2014)
338. -	*Laetiporus sulphureus* (Bull.) Murill	Mushroom		Aka, Bugun, Miji, Monpa and Sherdukpen (Arunachal Pradesh)	(Saha et al., 2014)
339. Jew's ear	*Auricularia auricular-judae* (Bull). J. Schrot	Mushroom		Aka, Bugun, Miji, Monpa and Sherdukpen (Arunachal Pradesh)	(Saha et al., 2014)
		Mushroom	*Murukan Kumizh*	Kaani (Tamil Nadu)	(Johnsy et al., 2011)
340. Vellathazan Kumizh	*Pleurotus roseus*	Mushroom	*Vellathazan Kumizh*	Kaani (Tamil Nadu)	(Johnsy et al., 2011)
341. Oyster mushroom	*Pleurotus ostreatus*	Mushroom	*Vellathazan Kumizh*	Kaani (Tamil Nadu)	(Johnsy et al., 2011)
342. Vellathazan Kumizh	*Pleurotus sajor caju*	Mushroom	*Vellathazan Kumizh*	Kaani (Tamil Nadu)	(Johnsy et al., 2011)
343. Ari kumizh	*Termitomyces microcarpus*	Mushroom	*Ari kumizh*	Kaani (Tamil Nadu)	(Johnsy et al., 2011)
344. Puttu Kumizh	*Termitomyces heimii*	Mushroom	*Puttu Kumizh*	Kaani (Tamil Nadu)	(Johnsy et al., 2011)
345. Straw mushroom	*Volvariella volvacea*	Mushroom	*Vaikol Kumizh*	Kaani (Tamil Nadu)	(Johnsy et al., 2011)
346. *Kollaan Kumizh*	*Lentinus squarrosulus*	Mushroom	*Kollaan Kumizh*	Kaani (Tamil Nadu)	(Johnsy et al., 2011)
347. King tuber mushroom	*Lentinus tuberegium*	Mushroom	*Mulan Kumizh*	Kaani (Tamil Nadu)	(Johnsy et al., 2011)
348. Sheep's head	*Grifola frondosa*	Mushroom	*Vella Murukan Kumizh*	Kaani (Tamil Nadu)	(Johnsy et al., 2011)
349. Flat Bulb mushroom	*Agaricus abruptibulbus*	Mushroom	-	Tribes of Karnataka	(Sudheep and Sridhar, 2014)
350. -	*Termitomyces globulus*	Mushroom	-	Tribes of Karnataka	(Sudheep and Sridhar, 2014)
351. Splitgill mushroom	*Schizophykm commune*	Mushroom	-	Naga (Manipur)	(Longvah and Deosthale, 1998)
352. Shiitake	*Lentinus edodes*	Mushroom	-	Naga (Manipur)	(Longvah and Deosthale, 1998)
353. Shatavri	*Asparagus racemosus* Willd.	Tuber	-	Palliyar,Bhil (Gujarat)	(Arinathan et al., 2009; Bhattacharjee et al., 2009)
354. Kali Musli	*Curculigo orchioides* Gaertn.	Tuber	-	Palliyar (Kerala)	(Arinathan et al., 2009)
355. Potato Yam	*Dioscorea bulbifera* L.	Tuber	-	Palliyar (Kerala)	(Arinathan et al., 2009)
			-	Kanikkars and Palliyars (Kerala)	(Shajeela et al., 2011)

Common name	Botanical name	Part consumed	Vernacular name	Accessed by indigenous community	Reference
			Haranbho /Piski sanga/Gethi Kanda	Munda, Oraon (Jharkhand)	(Ghosh-Jerath et al., 2015b, 2021)
			Pita Alu	Lodha (West Bengal), Tribes of Odisha	(Jana, 2004) (Padhan et al., 2020)
			Chedu dumpa	Savera, Jatapu, Gadabe and Kondadora (Andhra Pradesh)	(Rajyalakshmi and Geervani, 1994)
356. Indian yam	*Dioscorea oppositifolia* L.	Tuber	-	Kanikkars, Valiyans and Palliyars (Kerala)	(Arinathan et al., 2009; Mohan and Kalidass, 2010; Shajeela et al., 2011)
			Pan Alu	Lodha (West Bengal)	(Jana, 2004)
			Arika tega	Savera, Jatapu, Gadabe and Kondadora (Andhra Pradesh)	(Rajyalakshmi and Geervani, 1994)
			Paani Alu	Indigenous communities of Odisha	(Padhan et al., 2020)
357. Five leaf yam	*Dioscorea pentaphylla* L.	Tuber	-	Kanikkars, Valiyans and Palliyars (Kerala)	(Arinathan et al., 2009; Mohan and Kalidass, 2010; Shajeela et al., 2011)
			Nappe/Hasaer Sanga	Sauria Paharia, Munda (Jharkhand)	(Ghosh-Jerath et al., 2020a, 2021)
			Kanta Alu	Lodha (West Bengal)	(Jana, 2004)
			Pandimukku tega	Savera, Jatapu, Gadabe and Kondadora (Andhra Pradesh)	(Rajyalakshmi and Geervani, 1994)
			Panja sanga	Indigenous communities of Odisha t	(Padhan et al., 2020)
358. Nurai	*Dioscorea tomentosa J.Koenig ex Spreng*	Tuber	-	Kanikkars, Valiyans and Palliyars (Kerala)	(Arinathan et al., 2009; Mohan and Kalidass, 2010; Shajeela et al., 2011)
359. Wild butter bean	*Dolichos trilobus* L.	Tuber	*Minnikishangu*	Palliyar (Kerala)	(Arinathan et al., 2009)
360. Red Ginger	*Zingiber* sp	Tuber	*Kekir*	Adi (Arunachal Pradesh)	(Bhardwaj et al., 2009)
361. Ginger	*Zingiber officinale* Roscoe	Tuber	*Takeng*	Adi (Arunachal Pradesh)	(Bhardwaj et al., 2009)
362. Junglikhand		Tuber	*Cooked*	Bhil (Gujarat)	(Bhattacharjee et al., 2009)
			Raw	Bhil (Gujarat)	(Bhattacharjee et al., 2009)
363. Alli		Tuber	*Alli*	Sauria Paharia (Jharkhand)	(Ghosh-Jerath et al., 2020a)
364. Chalangan/ Chalango		Tuber	*Chalangan/ Chalango*	Sauria Paharia (Jharkhand)	(Ghosh-Jerath et al., 2020a)
365. Elephant foot yam	*Amorphophalluspaeoniifolius* (Dennst.) Nicolson	Tuber	*Singla*	Sauria Paharia, Munda (Jharkhand)	(Ghosh-Jerath et al., 2020a, 2021)
366. Alligator yam	*Ipomea digitate*	Tuber	*Bhui Kumro*	Lodha (West Bengal)	(Jana, 2004)
367. Purple Yam	*Dioscorea alata* L.	Tuber	*Khama Alu*	Lodha (West Bengal)	(Jana, 2004)
			-	Kanikkars and Palliyars (Kerala)	(Shajeela et al., 2011)
			Haatikata/ Aaru/ Jat sanga	Munda (Jharkhand)	(Ghosh-Jerath et al., 2021)
			-	Nicobari (Andaman & Nicobar Islands)	(Singh et al., 2018)
			Khamba aalu	Indigenous communities of Odisha	(Padhan et al., 2020)
368. Chun Alu	*Dioscorea floribunda* M.Martens & Galeotti	Tuber	*Chun Alu*	Lodha (West Bengal)	(Jana, 2004)
369. Churka Alu	*Dioscorea glabra* Roxb.	Tuber	*Churka Alu*	Lodha (West Bengal)	(Jana, 2004)
			-	Indigenous communities of Odihsa	(Padhan et al., 2020)
370. -	*Dioscorea wallichii* Hook.f.	Tuber	*Mou Alu*	Lodha (West Bengal)	(Jana, 2004)

Common name	Botanical name	Part consumed	Vernacular name	Accessed by indigenous community	Reference
			-	Kanikkars and Palliyars (Kerala)	(Shajeela et al., 2011)
			-	Indigenous communities of Odisha	(Padhan et al., 2020)
371. *Bon Ol*	*Amorphophallus sylvaticus* (Roxb.) Kunth	Tuber	*Bon Ol*	Lodha (West Bengal)	(Jana, 2004)
372. Floating lace plant	*Aponogeton natans* (L.) Engl. & K.Krause	Tuber	-	Valiyans (Kerala)	(Mohan and Kalidass, 2010)
373. -	*Boerhavia chinensis* (L.) Rottb.	Root	-	Valiyans (Kerala)	(Mohan and Kalidass, 2010)
374. Hadjod/Veldt grape	*Cissus quadrangularis* L.	Rhizome	-	Valiyans (Kerala)	(Mohan and Kalidass, 2010)
375. Kattumunthiri,	*Cissus vitiginea* L.	Tuber	-	Valiyans (Kerala)	(Mohan and Kalidass, 2010)
376. Queen sago	*Cycas circinalis* L.	Tuber	-	Valiyans (Kerala)	(Mohan and Kalidass, 2010)
377. -	*Cyphostemma setosum* (Roxb.) Alston	Tuber	-	Valiyans (Kerala)	(Mohan and Kalidass, 2010)
378. Swallow root	*Decalepis hamiltonii* Wight & Arn.	Tuber	-	Valiyans (Kerala)	(Mohan and Kalidass, 2010)
379. -	*Dioscorea hamiltonii* Hook.f.	Tuber	-	Indigenous communities of Odisha	(Padhan et al., 2020)
380. *Athikizhangu*	*Dioscorea spicata* Roth	Tuber	*Athikizhangu*	Valiyans (Kerala)	(Mohan and Kalidass, 2010; Shajeela et al., 2011)
381. Anantmul	*Hemidesmus indicus* (L.) R. Br. ex Schult.	Root	-	Valiyans (Kerala)	(Mohan and Kalidass, 2010)
382. -	*Ipomoea sumatrana* (Miq.) Ooststr.	Root	-	Valiyans (Kerala)	(Mohan and Kalidass, 2010)
383. -	*Kedrostis foetidissima* (Jacq.) Cogn.	Tuber	-	Valiyans (Kerala)	(Mohan and Kalidass, 2010)
384. -	*Maerua oblongifolia* (Forssk.) A.Rich.	Tuber	-	Valiyans (Kerala)	(Mohan and Kalidass, 2010)
385. Koka	*Nymphaea pubescens* Willd.	Tuber	-	Valiyans (Kerala)	(Mohan and Kalidass, 2010)
386. Water lily	*Nymphaea rubra* Roxb. ex Andrews	Tuber	-	Valiyans (Kerala)	(Mohan and Kalidass, 2010)
387. -	*Parthenocissus neilgherriensis* Planch.	Tuber	-	Valiyans (Kerala)	(Mohan and Kalidass, 2010)
388. Hooker chives	*Allium hookeri* Thwaites	Tuber	-	Adi (Arunachal Pradesh)	(Payum et al., 2015)
389. Indian three-leaved yam	*Dioscorea hispida* Dennst.	Tuber	*Pulidumpa*	Savera, Jatapu, Gadabe and Kondadora (Andhra Pradesh)	(Rajyalakshmi and Geervani, 1994)
			Banya aalu	Indigenous communities of Odisha	(Padhan et al., 2020)
390. Indiatic yam/Karen potato	*Dioscorea esculenta* (Lour.) Burkill	Tuber	-	Kanikkars and Palliyars (Kerala)	(Shajeela et al., 2011)
			Kankholi	Bhil (Gujarat)	(Bhattacharjee et al., 2009)
391. -	*Panax bipinnatifidus* Seem.	Tuber	-	Monpas, Memba and Khamba (Arunachal Pradesh)	(Tag et al., 2014)
392. *Kukai sanga*	*Dioscorea pubera* Blume	Tuber	*Kukai sanga*	Karbi (Assam)	(Terangpi and Teron, 2015)
				Indigenous communities of Odisha	(Padhan et al., 2020)

Common name	Botanical name	Part consumed	Vernacular name	Accessed by indigenous community	Reference
393. Tapioca	*Manihot esculenta* Crantz.	Tuber	*Adel sanga*	Munda (Jharkhand)	(Ghosh-Jerath et al., 2021)
394. Aromatic ginger	*Kaempferia galanga* L.	Root	*Sying smoh*	Khasi (Meghalaya)	(Agrahar-Murugkar and Subbulakshmi, 2005b)
395. *Lynniang*	*Potentilla lineata* Trevir.	Root	*Lynniang*	Khasi (Meghalaya)	(Seal, 2011)
396. Flemingia root	*Flemingia procumbens* Roxb	Tuber	*Soh phlang*	Khasi (Meghalaya)	(Agrahar-Murugkar and Subbulakshmi, 2005b; Chyne et al., 2019)
397. Cinquefoil roots	*Potentilla polyphylla* Wall. ex Lehm.	Root	*Lynniang*	Khasi (Meghalaya)	(Chyne et al., 2019)
398. Potato	*Solanum tuberosum* L.	Tuber	*Phan san minit*	Khasi (Meghalaya)	(Chyne et al., 2019)
399. -	*Alocasia acuminata* Schott.	Root	*Shriew khmat blang*	Khasi (Meghalaya)	(Chyne et al., 2019)
400. Sweet potato (white)	*Ipomoea batatas* (L.) Lam.	Root	*Phan karo lieh*	Khasi (Meghalaya)	(Chyne et al., 2019)
401. Sweet potato (red)	*Ipomoea batatas* (L.) Lam.	Root	*Phan karo saw*	Khasi (Meghalaya)	(Chyne et al., 2019)
402. Indian goosberry	*Phyllanthus emblica* L.	Fruit	*Amla*	Bhil (Gujarat)	(Bhattacharjee et al., 2009)
			Amlaki	Lodha (West Bengal)	(Jana, 2004)
403-404. Indian jujube	*Ziziphus jujuba* Mill.	Fruit	*Bore/Godaari/Ilkarpu/Ber*	Bhil (Gujarat)/Munda/Sauria Paharia	(Bhattacharjee et al., 2009; Ghosh-Jerath et al., 2020a)
	Zizyphus sp		*Charkul*	Lodha (West Bengal)	(Jana, 2004)
	Zizyphus sp.		*Kankul*	Lodha (West Bengal)	(Jana, 2004)
405. Sandpaper fig	*Ficus exasperata* Vahl.	Fruit	*Haani*	Sauria Paharia (Jharkhand)	(Ghosh-Jerath et al., 2020a)
406. Monkey jack	*Artocarpus lakoocha* Roxb.	Fruit	*Dahu*	Sauria Paharia,Munda,Oraon,Santhal (Jharkhand)	(Ghosh-Jerath et al., 2015b, 2016, 2020a, 2021)
407. Wild Fig	*Ficus racemosa* L.	Fruit	*Dumari/Dumar/Loa*	Sauria Paharia (Jharkhand)	(Ghosh-Jerath et al., 2020a)
			Umbara	Bhil (Gujarat)	(Bhattacharjee et al., 2009)
408. Kusum	*Schleichera oleosa* (Lour.) Merr.	Fruit	*Pusra/Baru*	Sauria Paharia,Munda, Oraon, Santhal (Jharkhand)	(Ghosh-Jerath et al., 2015b, 2016, 2020a, 2021)
409. Bhui-gular/ Khaina/ Khunia	*Ficus semicordata* Buch.-Ham. Ex Sm.	Fruit	*Anni/Aanri*	Sauria Paharia, Munda (Jharkhand)	(Ghosh-Jerath et al., 2020a)
410. *Ashfal*	*Euphorbia ingens* E.Mey. ex Boiss**.**	Fruit	*Ashfal*	Lodha (West Bengal)	(Jana, 2004)
411. Wood apple	*Aegle marmelos* (L.) Correa	Fruit	*Bel*	Lodha (West Bengal)	(Jana, 2004)
			Sinju/Bel	Munda, Sauria Paharia (Jharkhand)	(Ghosh-Jerath et al., 2020a)
			Billa	Bhil (Gujarat)	(Bhattacharjee et al., 2009)
412. Date	*Phoenix* sp.	Fruit	*Bon Khejur*	Lodha (West Bengal)	(Jana, 2004)
413. *Bainch Kul*	*Flacourtia amalotricha* A.C.S m.	Fruit	*Bainch Kul*	Lodha (West Bengal)	(Jana, 2004)
414. Cape jasmine	*Gardenia gummifera* L.f.	Fruit	*Bhurur*	Lodha (West Bengal)	(Jana, 2004)
415. Java Plum	*Syzygium cumini* (L.) Skeels	Fruit	*Jam*	Lodha (West Bengal)	(Jana, 2004)
416. Tumki	*Diospyros melanoxylon* Roxb.	Fruit	*Kendu*	Lodha (West Bengal)	(Jana, 2004)
			Tiril / Kendu/Kaanda	Sauria Paharia (Jharkhand)	(Ghosh-Jerath et al., 2020a)

Common name	Botanical name	Part consumed	Vernacular name	Accessed by indigenous community	Reference
417. *Tirphal/Chirphal.*	*Zanthoxylum rhetsa* DC	Fruit	*Tirphal/Chirphal.*	Tribes of Maharashtra	(Mahadkar et al., 2012)
418. Himalayan strawberry	*Cornus capitata* Wall.	Fruit	-	Aka, Bugun, Miji, Monpa and Sherdukpen (Arunachal Pradesh)	(Saha et al., 2014)
419. Malabar ebony	*Diospyros malabarica* (Desr.) Kostel.	Fruit	-	Aka, Bugun, Miji, Monpa and Sherdukpen (Arunachal Pradesh)	(Saha et al., 2014)
420. -	*Holboellia latifolia* Wall.	Fruit	-	Aka, Bugun, Miji, Monpa and Sherdukpen (Arunachal Pradesh)	(Saha et al., 2014)
421. Lissi	*Illicium griffithii* Hook.f. & Thomson	Fruit	-	Aka, Bugun, Miji, Monpa and Sherdukpen (Arunachal Pradesh)	(Saha et al., 2014)
422. -	*Machilus robusta* W.W. Sm.	Fruit	-	Aka, Bugun, Miji, Monpa and Sherdukpen (Arunachal Pradesh)	(Saha et al., 2014)
423. -	*Ocotea lancifolia* (Schott) Mez	Fruit	-	Aka, Bugun, Miji, Monpa and Sherdukpen (Arunachal Pradesh)	(Saha et al., 2014)
424. Sikkim crabapple	*Malus sikkimensis* (Wenz.) Koehne ex C.K.Schneid.	Fruit	-	Aka, Bugun, Miji, Monpa and Sherdukpen (Arunachal Pradesh)	(Saha et al., 2014)
425. Autumn Olive	*Elaeagnus umbellata* Thunb.	Fruit	-	Monpas, Memba and Khamba (Arunachal Pradesh)	(Tag et al., 2014)
426. Ambada	*Spondias pinnata* (L. f.) Kurz.	Fruit	*Amda*	Sauria Paharia, Munda, Santhal (Jharkhand)	(Ghosh-Jerath et al., 2015b, 2016, 2020a, 2021)
427. Mahua, ripe	*Madhuca longifolia* (J. Koenig. ex L.) J.F. Macbr.		*Mahua*	Sauria Paharia, Munda, Santhal, Oraon (Jharkhand)	(Ghosh-Jerath et al., 2015b, 2016, 2020a, 2021)
			Mahvoda	Bhil (Gujarat)	(Bhattacharjee et al., 2009)
428. Marking nut (kernel)	*Semecarpus anacardium* L.f.	Fruit	*Kero/Keero Toso/Soso/Bhelua*	Sauria Paharia, Munda, Santhals, Oraon (Jharkhand)	(Ghosh-Jerath et al., 2015b, 2016, 2020a, 2021)
429. Palmyra fruit, ripe (mesocarp)	*Borassus flabellifer* L.	Fruit	*Talmi/Tamras*	Sauria Paharia (Jharkhand)	(Ghosh-Jerath et al., 2020a)
430. Banyan fruit	*Ficus benghalensis* L.	Fruit	*Pakkedi/Baadi*	Sauria Paharia, Munda, Oraon (Jharkhand)	(Ghosh-Jerath et al., 2015b, 2020a, 2021)
431. *Zoge*	*Melodinus cochinchinensis* (L our.) Merr	Fruit	*Zoge*	Adi and Nyishi (Arunachal Pradesh)	(Seal et al., 2016)
432. Black current	*Antidesma ghaesembilla* Gaer tn.	Fruit	*Nuniari*	Indigenous communities of Odisha	(Nayak and Basak, 2015)
			Kattu-pulinchi	Kannikar, Malampandarangal, Paniyar and Kattunaykar (Kerala)	(Nazarudeen, 2010)
433. Wild guava	*Careya arborea* Roxb.	Fruit	*Kumbhi*	Indigenous communities of Odisha	(Nayak and Basak, 2015)
434. Fukian tea	*Ehretia microphylla* Lam.	Fruit	*Kujipana*	Indigenous communities of Odisha	(Nayak and Basak, 2015)
435. -	*Calamus guruba* Buch. -Ham. ex Mart.	Fruit	*Kanta beta*	Indigenous communities of Odisha	(Nayak and Basak, 2015)
436. Ram Phal	*Dillenia pentagyna* Roxb.	Fruit	*Rai*	Indigenous communities of Odisha	(Nayak and Basak, 2015)
437. Phalgu	*Ficus hispida* L.f.	Fruit	*Dimiri*	Indigenous communities of Odisha	(Nayak and Basak, 2015)
438. *Dimur*	NA	Fruit	*Dimur*	Nicobari (Andaman & Nicobar Islands)	(Singh et al., 2018)
439. Malabar melastome	*Melastoma malabathricum* L.	Fruit	*Karati*	Indigenous communities of Odisha	(Nayak and Basak, 2015)
440. Sewra	*Streblus asper* Lour.	Fruit	*Sahara*	Indigenous communities of Odisha	(Nayak and Basak, 2015)

Common name	Botanical name	Part consumed	Vernacular name	Accessed by indigenous community	Reference
441. Ottanghadi, Kilikhuti-ppazham	*Alangium salviifolium* subsp. *hexapetalum* (Lam.) Wangerin	Fruit	*Ottanghadi, Kilikhuti-ppazham*	Malampandarangal and Kattunaykar (Kerala)	(Nazarudeen, 2010)
442. Ponvetti, Vetti	*Aporosa cardiosperma* (Gaertn.) Merr.	Fruit	*Ponvetti, Vetti*	Kannikar and Malampandarangal (Kerala)	(Nazarudeen, 2010)
443. Mootippuli, Mootti	*Baccaurea courtallensis* (Wight) Müll.Arg.	Fruit	*Mootippuli, Mootti*	Kannikar, Malampandarangal and Paniyar (Kerala)	(Nazarudeen, 2010)
444. Wild Rhea	*Debregeasia longifolia* (Burm.f.) Wedd.	Fruit	*Neerinch, Monilli*	Mathuvar and Malampandaranga (Kerala)	(Nazarudeen, 2010)
		Fruit	*Jallatyrsim*	Khasi (Meghalaya)	(Seal and Chaudhuri, 2014)
445. Malabar tamarind	*Garcinia gummi-gutta* (L.) Roxb.	Fruit	*Kodampuli, Pinampuli*	Kannikar, Mathuvar, Malampandarangal and Paniyar (Kerala)	(Nazarudeen, 2010)
446. Jungle geranium	*Ixora coccinea* L.	Fruit	*Thetti, Chethi, Thechi*	Kannikar, Mathuvar, Malampandarangal and Paniyar (Kerala)	(Nazarudeen, 2010)
447. Spanish cherry/Maulsari	*Mimusops elengi* L.	Fruit	*Elengi*	Kannikar, Mathuvar, Malampandarangal and Paniyar (Kerala)	(Nazarudeen, 2010)
448. Palai	*Palaquium ellipticum* (Dalzell) Baill.	Fruit	*Pali, Bali*	Malampandaranga, Irular, Kadar and Paniyar (Kerala)	(Nazarudeen, 2010)
449. Tamilnadia	*Tamilnadia uliginosa* (Retz.) Tirveng. & Sastre	Fruit	*Pindichakka*	Paniyar and Kattunaykar (Kerala)	(Nazarudeen, 2010)
450. Soh Priam Khlaw	*Helicia nilagirica* Bedd.	Fruit	*Soh Priam Khlaw*	Khasi (Meghalaya)	(Seal and Chaudhuri, 2014)
451. Soh Phoh Khlaw	*Ilex venulosa* Hook.f.	Fruit	*Soh Phoh Khlaw*	Khasi (Meghalaya)	(Seal and Chaudhuri, 2014)
452. Soh-mlum/Sohma	*Rhus chinensis* Mill.	Fruit	*Soh-mlum/Sohma*	Khasi (Meghalaya)	(Seal and Chaudhuri, 2014)
453. Raitung	*Rhus parviflora* Roxb	Fruit	*Raitung*	Gond, Saharia (Madhya Pradesh)	(Jain and Tiwari, 2012)
454. Gamari	*Gmelina arborea* Roxb.	Fruit	*Shivan*	Indigenous communities of Maharashtra	(Mahadkar et al., 2012)
455. Indian trumpet flower	*Oroxylum indicum* (L.) Kurz	Fruit	*Tetu*	Indigenous communities of Maharashtra	(Mahadkar et al., 2012)
456. Gurial	*Bauhinia racemosa* Lam.	Fruit	*Apata*	Indigenous communities of Maharashtra	(Mahadkar et al., 2012)
457. Jaggery palm	*Caryota urens* L.	Fruit	*Ardhashishi*	Indigenous communities of Maharashtra	(Mahadkar et al., 2012)
458. -	*Viburnum corylifolium* Hook. f. & Thomson	Fruit	*Sohlang*	Khasi (Meghalaya)	(Agrahar-Murugkar and Subbulakshmi, 2005a)
459. Bird cherry	*Prunus bracteopadus* Koehne	Fruit	*Sohlang*	Khasi (Meghalaya)	(Agrahar-Murugkar and Subbulakshmi, 2005a)
460. Maiyan	*Meyna spinosa* Roxb. ex Link	Fruit	*Sohmaten*	Khasi (Meghalaya)	(Agrahar-Murugkar and Subbulakshmi, 2005a)
461. Sohthliem	*Gomphogyne cissiformis*	Fruit	*Sohthliem*	Khasi (Meghalaya)	(Agrahar-Murugkar and Subbulakshmi, 2005a)
462-463. Bayberry	*Myrica esculenta* Buch.-Ham. ex D. Don	Fruit	*Soh phie heh*	Khasi (Meghalaya)	(Chyne et al., 2019)
	Myrica nagi Thunb.	Fruit	*Soh phie nam*	Khasi (Meghalaya)	(Chyne et al., 2019)

Common name	Botanical name	Part consumed	Vernacular name	Accessed by indigenous community	Reference
464. Indian wild Pear	*Pyrus pashia* Buch.-Ham. ex D.Don	Fruit	*Soh jhur*	Khasi (Meghalaya), Monpas, Memba, Khamba, Aka, Bugun, Miji, Monpa and Sherdukpen (Arunachal Pradesh),	(Saha et al., 2014; Tag et al., 2014; Chyne et al., 2019)
465. Cane fruit	*Calamus erectus* Roxb.	Fruit	*Soh thri*	Khasi (Meghalaya)	(Chyne et al., 2019)
466. Rough lemon	*Citrus jambhiri*	Fruit	*Soh jalia*	Khasi (Meghalaya)	(Chyne et al., 2019)
467. Himalayan evergreen viburnum	*Viburnum foetidum* Wall.	Fruit	*Soh lang*	Khasi (Meghalaya)	(Chyne et al., 2019)
468. Korean mulberry	*Morus australis* Poir.	Fruit	*Soh lang dkhur*	Khasi (Meghalaya)	(Chyne et al., 2019)
469. Snail, big	*Pila globoasa*	Meat	*Loa suti*	Oraon,Munda,Sauria Paharia (Jharkhand)	(Ghosh-Jerath et al., 2015b, 2020a, 2021)
470. Red ants with eggs	*Oceophylla smaragdina*	Eggs	*Demta/Hau anda/ Chetado ka anda*	Oraon,Munda,Sauria Paharia (Jharkhand)	(Ghosh-Jerath et al., 2015b, 2020a, 2021)
471. Field rat’s meat	*Rattus argentiventer*	Meat	*Moosa/ Gudu*	Oraon,Munda,Sauria Paharia (Jharkhand)	(Ghosh-Jerath et al., 2015b, 2020a, 2021)
			Onder	Bhil (Gujarat)	(Bhattacharjee et al., 2009)
472. Pigeon	*Columba livia domestica*	Meat	*Kabutar/ Pervan*	Oraon,Munda,Sauria Paharia (Jharkhand)	(Ghosh-Jerath et al., 2015b, 2020a, 2021)
			Kabotar	Bhil (Gujarat)	(Bhattacharjee et al., 2009)
473. Puti	*Burbus* spp.	Meat	*Pothi hako/ Potha Hako*	Oraon,Munda,Sauria Paharia (Jharkhand)	(Ghosh-Jerath et al., 2015b, 2020a, 2021)
474. Freshwater mussels	*Margaritifera margaritifera*	Meat	*Setua/ Keyosuti/ Maako/jhinuk*	Oraon,Munda,Sauria Paharia,Santhal (Jharkhand)	(Ghosh-Jerath et al., 2015b, 2016, 2020a, 2021)
475. Prawn	*Macrobrachium* sp	Meat	*Chingra*	Oraon (Jharkhand)	(Ghosh-Jerath et al., 2015b)
476. Singhi	*Saccobranchus fossilis*	Meat	*Singhi*	Oraon,Sauria Paharia (Jharkhand)	(Ghosh-Jerath et al., 2015b, 2020a)
477. Crab	*Paratephusa spinigera*	Meat	*Kenkda*	Oraon (Jharkhand)	(Ghosh-Jerath et al., 2015b)
		Meat	*Karachala*	Bhil (Gujarat)	(Bhattacharjee et al., 2009)
478. Turtle’s meat	Testudinata	Meat	*Kachua*	Oraon (Jharkhand)	(Ghosh-Jerath et al., 2015b)
479. Beef, chops	*Bos Taurus*	Meat	*Gai*	Bhil (Gujarat)	(Bhattacharjee et al., 2009)
480. Duck	*Anas platyrhynchos*	Meat	*Batakh*	Oraon (Jharkhand)	(Ghosh-Jerath et al., 2015b)
481. Egg, duck	*Anas platyrhynchos*	Egg	*Batakh anda*	Oraon (Jharkhand)	(Ghosh-Jerath et al., 2015b)
482. Bombay duck	*Harpodon nehereus*	Meat	*Bubla*	Bhil (Gujarat)	(Bhattacharjee et al., 2009)
483. Walking catfish	*Clarias batrachus*	Meat	*Magur/Mangri*	Oraon,Munda,Sauria Paharia (Jharkhand)	(Ghosh-Jerath et al., 2015b, 2020a, 2021)
484. Wallago	*Wallago attu*	Meat	*Boari*	Sauria Paharia (Jharkhand)	(Ghosh-Jerath et al., 2020a)
485. Freshwater Eel	*Anguilla Anguilla*	Meat	*Gacchi*	Sauria Paharia (Jharkhand)	(Ghosh-Jerath et al., 2020a)
486. Catfish	*Mystus vittatus*	Meat	*Tengra/Tonger*	Sauria Paharia (Jharkhand)	(Ghosh-Jerath et al., 2020a)
487. Shark	*Carcharhinu ssorrah*	Meat	*Khari Machli*	Bhil (Gujarat)	(Bhattacharjee et al., 2009)
488. Silhan	*Silonia silondia*	Meat	*Silong*	Sauria Paharia,Munda (Jharkhand)	(Ghosh-Jerath et al., 2020a, 2021)
489. Bele fish	*Glossogoboius giuris*	Meat	*Bale Machli*	Sauria Paharia,Munda (Jharkhand)	(Ghosh-Jerath et al., 2020a, 2021)
490. -	*Botia berdmorei*	Meat	-	Indigenous communities of Manipur	(Sarojnalini, 2010)

**Common name**	**Botanical name**	**Part consumed**	**Vernacular name**	**Accessed by indigenous community**	**Reference**
491. Guntea loach	*Lepidocephalichthys guntea*	*Meat*	*Ngakijou*	Indigenous communities of Manipur	(Shantosh and Sarojnalini, 2018)
492. *Nganap*	*Pangio pangia*	Meat	*Nganap*	Indigenous communities of Manipur	(Shantosh and Sarojnalini, 2018)
493. *Sareng-Khoibi*	*Syncrossus berdmorei*	Meat	*Sareng-Khoibi*	Indigenous communities of Manipur	(Shantosh and Sarojnalini, 2018)
494. Fowl/Rooster	*Galloanserae*	Meat	*Jangli Murgi*	Sauria Paharia,Munda,Oraon,Santhals (Jharkhand)	(Ghosh-Jerath et al., 2015b, 2016, 2020a, 2021)
495. Quail	*Coturnix coturnix*	Meat	*Bater*	Sauria Paharia,Munda (Jharkhand)	(Ghosh-Jerath et al., 2020a, 2021)
496. Pig	*Sus scrofa*	Meat	*Jangli suar//kissu*	Sauria Paharia,Munda,Oraon,Santhals (Jharkhand)	1–3,5)
			Jangli Bhund	Bhil (Gujarat)	(Bhattacharjee et al., 2009)
497-498. Grasshopper	*Chondacris rosea*	Meat	*mirbo'/'takam/kamrak*	Galo, Adi, Nyishi, Apatani, Deori, Wancho, Singpho, Chaknma (Arunachal Pradesh)	(Chakravorty et al., 2014)
	Oedaleus abruptus (Thunberg)		-	Indigenous communities of West Bengal	(Ganguly et al., 2013)
499. Mole cricket	*Brachytrupes orientalis*	Meat	*'takam hilak'/'takam pario tokcho'/'komdruk*	Galo, Adi, Nyishi, Apatani, Deori, Wancho, Singpho, Chaknma (Arunachal Pradesh)	(Chakravorty et al., 2014)
500. Stink Bug	*Aspongopus nepalensis* Westwood 1837	Meat	*Gandhipuk/tari/Gondhibug*	Nyshi, Adi, Apatani, Nocte, and Wangchoo (Arunachal Pradesh)	(Chakravorty et al., 2011)
501. Mulberry silkworm	*Bombyx mori*	Meat	-	Ahom, Sonowal Kachahari, Muttock (Assam)	(Mishra et al., 2003)
502-503. Non-mulberry silkworm	*Attacus ricinii*	Meat	*Eri*	Ahom, Sonowal Kachahari, Muttock (Assam)	(Mishra et al., 2003)
	Antherae assama		*Muga*	Ahom, Sonowal Kachahari, Muttock (Assam)	(Mishra et al., 2003)
504. Giant water bug	*Lethocerus indicus* (Lepeletier and Serville)	Meat	-	Indigenous communities of Manipur	(Shantibala et al., 2014)
505. Water scorpion	*Laccotrephes maculatus* (F.)	Meat	-	Indigenous communities of Manipur	(Shantibala et al., 2014)
506. Water scavenger beetles	*Hydrophilus olivaceous* (F.)	Meat	-	Indigenous communities of Manipur	(Shantibala et al., 2014)
507. Beetle	*Cybister tripunctatus* (Olivier)	Meat	-	Indigenous communities of Manipur	(Shantibala et al., 2014)
508. Scarlet skimmer	*Crocothemis servilia* (Drury)	Meat	-	Indigenous communities of Manipur	(Shantibala et al., 2014)
