# Supplementary material for: Indigenous Foods of India: A Comprehensive Narrative Review of Nutritive Values, Antinutrient Content and Mineral Bioavailability of Traditional Foods Consumed by Indigenous Communities of India
Source: Front Sustain Food Syst. Author manuscript; Available in PMC 2022 May 22. (PMC7612755; doi:10.3389/fsufs.2022.696228)
Supplement: Table 1 [file EMS145028-supplement-Table_1.docx]

**Supplementary table 1: Summary of methodology followed in studies included in the review**

| **First author (year)** | **Broad region** | **Methodology** | | |
| --- | --- | --- | --- | --- |
|  |  | **Food sample collection & taxonomic identification** | **Macronutrient & Micronutrient estimation** | **ANF estimation** |
| 1. (Agrahar-Murugkar and Subbulakshmi, 2005a) | North-east | - 500g of each food sample was collected and sent for taxonomic identification to National Bureau of Plant Genetic Resources, Indian Council of Agricultural Research (ICAR), Barapani, Meghalaya and BSI, Shillong, Meghalaya, India | - Protein estimated by Kjeldahl method (AOAC, 1984), fat determined by extracting a known weight of powdered plant sample with petroleum ether using the Labconco ether extract apparatus (AOAC, 1984). - The micronutrients (Fe and Zn) were determined by (AAS). Vitamin C was determined by a reduction method of (Raghuramulu et al. 1983). The Vitamin A (β-carotene) was estimated using the method of (Tee et al., 1995) | - |
| 1. (Arinathan et al., 2009) | South | - Food samples were collected using multistage sampling technique in three consecutive rainy seasons. - No methodology reported for taxonomic identification | - Protein was calculated by multiplying the per cent Kjeldahl (Humphries, 1956) nitrogen with the factor 6.25. The remaining components of proximate composition were estimated by AOAC methods (Horwitz et al., 1970). The energy content of the tuber was determined by multiplying the amount of crude protein, crude fat and nitrogen free extractives with the factors 16.7,37.7,16.7 respectively (Siddhuraju et al., 2002). - Vitamins C and B3 were extracted and estimated following the method of (Sadasivam, and Manickam, 2008). Fe, Ca and Zn were analysed using AAS (Isaac and Johnson, 1975). | - Anti-nutritional factors like total free phenols (Sadasivam, and Manickam, 2008) tannins (Burns, 1971) and hydrogen cyanide (Jackson, 1967), were quantified. |
| 1. (Basumatary and Narzary, 2017) | North-east | - Food samples were collected during their seasonal availability and voucher specimens of all the plant species were submitted to BSI, Shillong, Meghalaya and authenticated. | - Crude fat was determined by extracting with petroleum ether using a Soxhlet apparatus. Crude protein was estimated by the Kjeldahl method. Total protein was calculated by multiplying the evaluated nitrogen by a protein conversion factor of 6.25. Energy value of the sample was calculated on the basis of data of proximate analysis (Narzary and Basumatary, 2015). - Micronutrients (Ca, Fe and Zn) were determined using Graphite Furnace-AAS Vitamin C was estimated using 2, 6-dichlorophenol indophenol by titration method (Narzary and Basumatary, 2015). | - |
| 1. (Bhardwaj et al., 2009) | North-east | - FGDs were conducted to identify the commonly consumed IFs. Food samples were collected and photographed, and herbarium specimens were prepared for identification at the herbarium of the botany department of the Rajiv Gandhi University and at the herbaria of the BSI, Itanagar, Arunachal Pradesh. - Around 2kg of food samples were collected from each location. 3-4 samples were collected in all at different times of the cropping season. Collected samples were brought to the laboratory within six hours of harvesting. - No methodology reported for taxonomic identification | - Crude protein and crude fat were analysed as described in (AOAC, 1990). Crude protein was estimated by determining total nitrogen using Kjeldahl digestion and distillation methods. Total protein was calculated by multiplying percent nitrogen by 6.25. Crude fat was estimated by extracting with petroleum ether in Soxhlet apparatus. - Vitamin C was analysed by atomic absorption spectrophotometry by the method of (Jagota and Dani, 1982) using folin-phenol reagent. | - |
| 1. (Bhattacharjee et al., 2009) | West | - Field visits and rigorous food consumption survey, adapted from standard tools, were conducted to identify the commonly consumed IFs. - No methodology reported for food sample collection and taxonomic identification. | - Nutrient values (energy, protein, fat, Fe, Ca, Zn, B-groups vitamins, vitamin A and C) were calculated using the Indian Food Composition Tables (Gopalan et al., 1978). Food samples with no documentation in the Indian Food Composition Tables were analysed at the National Institute of Nutrition in Hyderabad, India. | - |
| 1. (Chakravorty et al., 2011) | North-east | - Live adult insect specimens were collected from vegetation along the banks of the river and were taken to the Biochemical Nutrition Laboratory of Rajiv Gandhi University in chilled freeze-boxes. The insects were scientifically identified at Zoological Survey of India, Kolkata, India | - The crude protein content of the samples was estimated by the macro-Kjeldahl method. Fat was extracted by Soxhlet method - Ca, Fe and Zn were determined by AAS after dry-ashing and acid dilution (AOAC, 1990). Vitamin A,B1,B2,B3,B6,C were determined according to (Raghuramulu et al., 1983). | - |
| 1. (Chakravorty et al., 2014) | North-east | - Adult insect specimens were collected from agricultural fields and bushes alongside the roads. All specimens were taken to the Biochemical Nutrition Laboratory of Rajiv Gandhi University in chilled freeze-boxes. The insects were taxonomically identified in the laboratory, confirmed by the Zoological Survey of India in Kolkata. | - Using the techniques recommended by the (AOAC, 1990), crude protein and crude fat were estimated. Crude protein was determined by the Kjeldahl method and total protein content was calculated as the amount of total N determined multiplied by nitrogen-to-protein conversion factor of 6.25. Fat percentage was calculated by drying fats after extraction in a Soxhlet using petroleum benzene. Energy value was calculated by multiplying the values obtained for carbohydrate, protein and fat with 4, 4 and 9 respectively and adding up the values. | - |
| 1. (Chyne et al., 2019) | North-east | - Food sampling was done following the prime objectives set by FAO. 42 samples were collected separately (about 500g portion of each sample) from each of the selected villages and were transported to the laboratory for nutrient analyses. The edible portion of food samples were separately cleaned and processed immediately for the nutrient analyses. Herbarium specimens of the samples were prepared and the botanical identification was done at the BSI, Shillong, Meghalaya | - Standard AOAC methods (Horwitz et al., 2006) were used for analysis of crude protein (984.13) by Kjehdahl method, ash (942.05) using a Muffle furnance and fat by solvent extraction method, using chloroform: methanol (2:1) (v/v). - Flame-AAS used for estimation of minerals. - Analysis of water soluble vitamins (Vitamin C, B6, B5 and B9) was carried out using U-HPLC. Vitamin C was quantified according to (Phillips et al., 2010). |  |
| 1. (Das et al., 2015) | East | - Sixteen morphologically different types of wild edible mushrooms from natural habitat were collected during rainy and post rainy seasons of 3 consecutive years. - No methodology reported on taxonomic identification. | - Total protein and estimation as per (Lowry et al., 1951), and fat (Bligh and Dyer, 1959) contents were quantified in each mushroom sample. - Micronutrients (Ca, Fe, and Zn) analysed using a graphite furnace and AAS and flame photometer | - |
| 1. (Ganguly et al., 2013) | East | - 987 adult insect specimens were collected from the nearby grasslands and croplands by sweeping technique, using standard insect nets. They were freeze killed prior to the estimation of wet body weight. Then they were oven dried (Indian instrument manufacturing company, Kolkata, India) at 60°C until the dry body weight became constant. - No methodology reported for taxonomic identification. | - Nutrient composition was estimated by standard procedures according to (AOAC, 1990) on dry matter basis. Percentage nitrogen content was estimated by Micro-Kjeldahl method using Tecator Kjeltec system (Sweden). Nitrogen content was converted into crude protein (%) using the factor N × 6.25. Crude fat was estimated by ether extraction method, using Soxhlet apparatus. - Mineral contents such as Ca, Fe and Zn, were estimated by Varian Techtron AAS using standard reference chemicals according to (Anand et al., 2008). | - Anti-nutritional factor of phenolic polymers like tannin was determined chemically with vanillin-HCl reagent and catechin solution according to (Mandal et al., 2018). Content of oxalate was determined by simple titration using methyl red as indicator following the procedures proposed by (Mandal et al., 2018). Titration was again used to measure the amount of phytin phosphorus using ferric chloride (FeCl3) as indicator according to (Agbede and Aletor, 2004). Phytin content was calculated with a multiplication of the value of phytin phosphous by 3.55 to (Agbede and Aletor, 2004). |
| 1. (Ghosh-Jerath et al., 2015) | East | - Free listing and focus group discussions (FGDs) were used to assess the range of available foods. Food samples were either provided by participants or were collected by the research team; these samples were then sent for classification to a team of experts at the Botany department of Birsa Agricultural University, Ranchi. The photographed food item (around 50–100 g; all parts) was collected, wrapped in paper towels, placed in a well perforated polythene bag and sent to the botanists for identification/confirmation of the botanical classification. - The Indian Food Composition tables were checked for availability of the nutritive values of the identified foods and finally a list of the items not available in the Indian food composition tables was prepared for collection for nutrient analysis. - 500g food sample was collected, dusted to remove excess soil/dirt taking care to avoid mechanical damage and air dried to remove extraneous moisture. The samples were then weighed, wrapped in clean paper towels, placed in well-perforated polythene bags, and placed in a carrier lined with ice packs before being transported to NABL laboratory for nutrient analysis.. | - The parameters analyzed included energy (IS 14433: 2007); Clause 6.10.1 C; IS 1656: 2007 Appx. C.), protein (titrimetric), total fat (IS: 4684: 1975). - Micronutrients analysed were vitamin A (β-carotene), vitamin B1,B2B3 (AACC.1995.86–90 and Roche Analytical Manual), vitamin C, B9, Ca, Fe and Zn (AOAC 999.10 and AOAC 999.11). | - |
| 1. (Ghosh-Jerath et al., 2016) | East | - Free listing and focus group discussions (FGDs) were used to assess the range of available foods. - Based on the data, a list of commonly consumed IFs was compiled and a literature search was done to identify the taxonomic classification based on the common names provided by the community. Samples were collected for those food items whose taxonomic names were not found, and these were sent for classification to an expert team at the Department of Botany, Birsa Agricultural University, Ranchi, Jharkhand. - The Indian Food Composition tables were checked for availability of the nutritive values of the identified foods and finally a list of the items not available in the Indian food composition tables was prepared for collection for nutrient analysis. - 500g food sample was collected, dusted to remove excess soil/dirt taking care to avoid mechanical damage and air dried to remove extraneous moisture. The samples were then weighed, wrapped in clean paper towels, placed in well-perforated polythene bags, and placed in a carrier lined with ice packs before being transported to NABL laboratory for nutrient analysis. | - .The samples were analysed for parameters including energy (Codex Guidelines for Nutritional Labelling _CAC/GL 2-1985) (Joint FAO/WHO Codex Alimentarius Commission et al., 2007) protein (IS 7219- 1973) (Bureau of Indian Standards, 1973) and total fat (IS-4684- 1975) (Indian Standards 4684–1975, 2000). - The vitamins including vitamin A (as β-carotene) (Shahidan et al., 2012), vitamin B1 (Nielsen, 2010) B2, (Shahidan et al., 2012) B3 (Nielsen, 2010) were estimated by HPLC based UV-visual detection, vitamin C (Food Hygiene, Sampling and Analysis Sectional Committee, 2005) by titration (IS 5838-1970) and vitamin B9 by the BioRad ELISA kit, MA USA. The minerals i.e. Ca, Fe and Zn were analyzed based on the AOAC 999.10 methodology (AOAC, 2002) | - |
| 1. (Ghosh-Jerath et al., 2020) | East | - FGDs were used to elicit a detailed description of food availability, access, and utilization in the community. Based on the data, a list of commonly consumed foods was compiled and a literature search based on the common names of the IFs and their photographs (in some cases) was done for taxonomic classification with key inputs from Plant Taxonomists and Botanists. Some food samples whose taxonomic classifications were not available, were collected based on seasonal availability and herbariums were prepared. The sample collection was carried out following the standard protocols. Standard protocols were followed for herbarium preparation and these herbariums were sent to an expert team at BSI. - After the taxonomic classification of IFs, Indian Food composition tables and other literature sources were searched to identify pre-existing information on their nutritive values. Food samples with missing or no available nutritive values in the secondary literature were collected by the field team subject to their seasonal availability and sent to a NABL accredited laboratory for nutrient analysis. | - Nutrient analysis was done according to standard reference protocols developed as part of the study in consultation with the laboratory. Energy and fat, were analysed using gravimetric method, protein via titrimetric method. - The vitamins (A, B1, B2, B3, B6, B9, C) were assessed using High-performance Liquid Chromatography (HPLC) and the minerals (Fe, Ca, Zn) by Inductively Coupled Plasma Mass Spectrometry (ICP-MS). | - |
| 1. (Ghosh-Jerath et al., 2021) | East | - A total of nine FGDs and six interviews were conducted to do free-listing of all indigenous foods known to the community. The listed foods were identified through their common names as well as photographs and verified by an ethnobotanist in the team, who has extensively worked on taxonomic classification of IFs of Mundas. The nutritive value of these identified IFs were searched in the Indian food composition database and other secondary literature and were collated. Foods with no secondary data on nutritional values, were collected from field sites using standard protocols and nutrient analysis was conducted according to standard reference procedures in consultation with the NABL accredited laboratory. | - Nutrient analysis was done according to standard reference protocols developed as part of the study in consultation with the laboratory. Energy and fat, were analysed using gravimetric method, protein via titrimetric method. - The vitamins (A, B1, B2, B3, B6, B9, C) were assessed using High-performance Liquid Chromatography (HPLC) and the minerals (Fe, Ca, Zn) by Inductively Coupled Plasma Mass Spectrometry (ICP-MS). | - |
| 1. (Gupta et al., 2005) | South | - Thirteen GLVs were selected for the study. They were identified by a taxonomist. The fresh leaves were procured from the local markets or field locations. The leaves were separated from roots, washed under running water, followed by double glass distilled water. They were drained completely and used for analysis. | - The nitrogen content was estimated by Kjeldahl method, using the formula, protein=nitrogen x 6.25. - Vitamin C was estimated by visual titration method of reduction of 2,6-dicholorophenol-indophenol dye. Total carotene was extracted in acetone; β-carotene was separated by column chromatography and estimated colorimetrically. volume (Ranganna and Ranganna, 2003). Vitamin B1 was analysed by oxidation to thiochrome, which fluoresces in UV light (Raghuramulu et al., 1983). Fe was estimated colorimetrically by a-adipyridyl method (Taussky and Shorr, 1953; Horwitz et al., 1970). Ca was analysed by precipitation as calcium oxalate and subsequent titration by potassium permanganate (Hawk & Oser 1965) Samples for determination of minerals (Fe and Zn) were digested using nitric/sulphuric acid mixtures and diluted to a known volume (Ranganna and Ranganna, 2003). | - Total oxalate was analysed by extraction with hydrochloric acid and soluble oxalate with water followed by precipitation with calcium oxalate from deproteinized extract and subsequent titration with potassium permanganate (Baker, 1952). Tannins were extracted in methanol and read colorimetrically by using vanillinhydrochloride method (Burns 1971). Phytic acid was extracted and determined. The conversion factor 3.55 for phosphorus to phytic acid was used. |
| 1. (Horo and Topno, 2015) | East | - Interviews were undertaken to explore the wild edible plants consumed within the community. Carefully field notes were taken about the botanical name, family, local name, flowering and fruiting times, part of the plant used as food and their mode of uses. The collected plants were identified based on the flora of relevant scientific literature and standard floras. Plant samples were collected from several places of W. Singhbhum district and taxonomically identified. | - Analysis of nutritional values protein, calories, Ca, Fe, vitamin A and vitamin C were determined using standard methods of (AOAC International and Latimer, 2012; Mahapatra et al., 2012). Protein were determined using the neutral detergent (Gopalan et al., 1978) | - |
| 1. (Jain and Tiwari, 2012) | Central | - No methodology reported for food sample collection and taxonomic collection. | - Protein (Lowry et al., 1951) and lipids (Bligh and Dyer, 1959) have been estimated by standard methods | - |
| 1. (Jana, 2004) | East | - Interviews and FGDs were conducted to identify the local foods consumed within the community. - No methodology reported for food sample collection and taxonomic classification | - Protein and fat estimated by standard procedures by National Institute of Nutrition - No methodology reported for micronutrient estimation. | - |
| 1. (Johnsy et al., 2011) | South | - Field trips and personal interviews were conducted with local indigenous people to identify the usage of local mushrooms. - Mushrooms were collected from four different locations from nearby forests and hills. The specimens were carefully uprooted by gently lifting them up and holding the stipe gently but firmly close to the rhizomorph, thus carrying some soil along with it. This is to avoid damaging the tissue of the mushroom. Each specimen was carefully labeled before transporting to the laboratory in the town. The specimens were air-dried and stored in transparent polythene bags that were loosely kept to allow for proper aeration of the specimens. - The collected mushrooms were identified by CAS in Botany, University of Madras, Chennai, Tamil Nadu | - Protein content was determined using Folin phenol reagent (Kadiri and Fasidi, 2012) . | - |
| 1. (Laddha et al., 2015) | West | - Field survey, collection of leafy vegetable, and its related data, were carried out in different seasons. Ethnic informers were selected to locate and collect the plant along with the other informants. Useful information was gathered by interviewing the local people and elderly persons. The specimens were identified by carefully matching them in the herbarium and authentically certified, by the Department of Botany Hislop College, Nagpur (Specimen no. 6821). | - The protein was determined using micro Kjeldahl method. Fat content was determined by extracting sample with petroleum ether in a Soxhlet extractor (Chopra and Kanwar, 1991). Energy value was determined by multiplying the percentages of crude protein, crude lipid and NFE by the factors 16.7, 37.7 and 16.7, respectively (Indrayan et al., 2005). - For mineral estimation (Fe, Ca and Zn), standard solution of each element was prepared and calibration curves were drawn for each element using AAS/FPM. | - |
| 1. (Sebestianus Lakra, 2019) | Central | - No methodology reported for food sample collection and taxonomic collection. | - Nutrient values (energy, protein, fat, Fe, Ca, Zn, B-groups vitamins, vitamin A and C) were calculated using the Indian Food Composition Tables (Gopalan et al., 1978). |  |
| 1. (Longvah and Deosthale, 1991) | North-east | - Samples were purchased from the local market of Ukhrul, Manipur, and transported by air to the laboratory for analysis. - No methodology reported for taxonomic identification | - Protein content (N x 6.25) was estimated by the Kjeldahl method; fat and ash were determined by AOAC methods. - Minerals were determined in AAS. |  |
| 1. (Longvah and Deosthale, 1998) | North-east | - Dried mushrooms were purchased from the local markets. The whole mushrooms (i.e. pileus + stipe) were powdered to pass through a 40 mesh sieve and stored until analysis. - No methodology reported for taxonomic identification | - Proximate analysis, including crude fat, crude protein (N x 6.25) and ash, were performed according to (AOAC, 1990) procedure. - Minerals were determined by atomic absorption spectrophotometer after dry-ashing the samples (AOAC, 1990) |  |
| 1. (Longvah et al., 2020) | North-east | - Dry paddy of 32 rice landraces including 8 glutinous varieties were sampled from different locations. The rice landraces were selected according to the indigenous farmers’ overall preference for its yield and taste at the place of sampling. - No methodology reported for taxonomical classification | - Total nitrogen content was determined by titrimetry in an automated Kjeldahl instrument which was multiplied by a conversion factor of 5.95 to obtain the protein content. Total fat content was determined after acid hydrolysis using classic Soxhlet apparatus (AOAC International and Latimer, 2012). - Vitamin B1 was determined from the fluorescence intensity of its oxidation product, thiochrome (AOAC 942.23). Vitamin B2 and B3 in the test samples were estimated by microbiological assay (AOAC 940.33, 960.46 and 985.34). Total vitamin B6 representing the sum of pyridoxal, pyridoxine and pyridoxamine were extracted by acidified organic solvent and quantified by RP-HPLC (AOAC 985.32). The minerals were determined by AAS using airacetylene flame at suitable detection wavelengths. |  |
| 1. (Loukrakpam et al., 2019) | North-east | - The samples selected for the study were those available during the pre-monsoon season. Fresh vegetable samples were collected from local market and village households and were transported to laboratory for analysis. The samples were cleaned and edible portion were processed for comprehensive nutrient analysis. | - Standard AOAC methods (Official Methods of Analysis, 2005) were used to determine the proximate composition. Protein values were calculated from the estimated nitrogen (Kjedahl method) Jones conversion factor i.e. 6.25. The total fat content of the samples was determined by gravimetric method using a mixed solvent of chloroform and methanol (AOAC 963.15). - Water soluble vitamins such as vitamin C, B9 (total folate), and B6 were determined by U-HPLC. Vitamin C was quantified according to (Phillips et al., 2010). Vitamin B9 was determined using trienzymatic extraction and quantification by U-HPLC- Ultra Violet (UV) or fluorescence detection (Rader et al., 1998; Brouwer et al., 1999). Vitamin B6 in samples was extracted using metaphosphoric acid and analysed by U-HPLC technique (Valls et al., 2001). Minerals were determined by AAS-Flame. | - |
| 1. (Mahadkar et al., 2012) | West | - Samples were collected from various sites and. washed to remove dirt and dried at room temperature. Samples were then transferred to grinding machine to make powder and these dried powder was then used for acid digestion. - No methodology reported for taxonomic identification | - The acid digestion method of (Toth et al., 1948) has been followed for the analysis of inorganic constituents. Ca, Fe and Zn were estimated by using AAS. | - |
| 1. (Medak and Singha, 2016) | North-east | - Randomly sampled fresh edible parts of the three selected wild plants were collected from their natural habitats. The samples were thoroughly washed with distilled water and oven dried using paper envelop at 70 ± 5 0C for a week. Dried samples were ground into fine powder using an electric grinder and stored in room temperature in airtight container for detail chemical analysis. - No methodology reported for taxonomic identification | - Crude fat and crude protein in the plant samples were determined following standard methods as outlined in (AOAC, 1990). Energetic value of edible portion of the plants was determined by multiplying the protein, fat and carbohydrate content by 4.0, 9.0 and 4.0, respectively (MacLean et al., 2003) - Ca was determined using flame photometer | - |
| 1. (Medak and Singha, 2018) | North-east | - Randomly sampled fresh edible parts were collected from natural habitats. Plant samples were thoroughly washed with distilled water and dried in a hot air oven at 70 ± 50C for a week. The dried samples were finely ground into powder for the analysis of trace elements and antioxidant activities - No methodology reported for taxonomic identification | - Trace elements, such as, Fe and Zn were determined through AAS after digestion of the plant samples in tri-acid following standard methods as outlined by (Allen et al., 1974). Vitamin C was determined following standard method as outline by (Sadasivam, and Manickam, 2008) using fresh tissue. | - |
| 1. (Mishra et al., 2003) | North-east | - Cocoons of non-mulberry silkworms and mulberry silkworm were purchased from the villages during winter season and were in their pupal stages of life cycle. - No methodology reported for taxonomic identification. | - The cocoons were processed before analyzing for food value. They were boiled for 30 minutes at 100°C. The pupae were taken out from cocoons, blotted on filter paper and dissected to remove intestine and waste material present. - The crude protein was estimated by the Kjeldahl method (% protein 61%N 6.25) (method 14.063); the crude fat was estimated using dried ether by Soxhlet extraction; The energy content was determined by multiplying percentage of crude fat, crude protein, and carbohydrate by factors of 37.7, 16.7 and 16.7 respectively. | - |
| 1. (Mohan and Kalidass, 2010) | South | - Wild edible tubers, rhizome, corm, roots and stems grown in sandy loam soil were collected using multistage sampling technique in three consecutive rainy seasons (each seasons three samples- sample size 2 kg). - No methodology reported for taxonomic identification. | - The nitrogen content was estimated by the micro Kjeldahl method (Humphries, 1956) and the crude protein content was calculated (N x 6.25). Crude lipid content was determined using Soxhlet apparatus (Official Methods of Analysis, 2005). - The energy value of the seed (kJ) was estimated by multiplying the percentages of crude protein, crude lipid and NFE by the factors 16.7, 37.7 and 16.7, respectively (Siddhuraju et al., 2002). - Vitamin C and B3 were extracted and estimated as per the method given by (Sadasivam, and Manickam, 2008) | - The anti-nutritional compounds, total free phenols (Bray and Thorpe, 2006), tannins (Burns, 1971), hydrogen cyanide (Jackson, 1967) and total oxalate (AOAC: Official Methods of Analysis (Volume 1), 1984) were quantified. Trypsin inhibitor activity was determined by the enzyme assay of (Kakade et al., 1974) by using benzoil-DL-arginin-pnitroanilide (BAPNA) as a substrate. Amylase inhibitor activity was determined by the method of (Rekha & Padmaja 2002) by using 0.5% soluble starch as substrate. |
| 1. (Agrahar-Murugkar and Subbulakshmi, 2005b) | North-east | - Rapid rural appraisal method was used to identify the most commonly consumed IFs in the area. These samples were then gathered from different areas and processed before analysis. - No methodology reported for taxonomic identification. | - The crude protein content of the samples was estimated by macro Kjeldahl method (AOAC, 1990). Fat in the samples was determined by extracting a known weight of powdered plant sample with petroleum ether, using the Labconco (Germany) ether extract apparatus (AOAC, 1990). - The micronutrients (Fe and Zn) were determined by AAS method. Ca was precipitated in acidic medium as insoluble calcium oxalate by adding saturated ammonium oxalate solution. Vitamin C was determined by a reduction method using a dye (2,6 di-chloro-phenol indophenol) (Raghuramulu et al., 1983). |  |
| 1. (Agrahar-Murugkar, 2006) | North-east | - Seven unconventional mushrooms were identified with the help of a rapid rural appraisal survey and these were collected from the forests and local markets. The mushrooms were scientifically identified at the National Bureau of Plant Genetic Resources, ICAR, Barapani, Meghalaya and BSI, Shillong, Meghalaya, India. | - Fe were determined by AAS. Ca was precipitated in acidic medium as insoluble calcium oxalate by adding saturated ammonium oxalate solution (Raghuramulu et al., 1983). Vitamin A (β- carotene) was estimated by the method given by (Tee et al., 1995) | - |
| 1. (Nayak and Basak, 2015) | East | - Fresh and disinfected ripe fruits were collected from various forest reserves. Fruits were botanically identified with the help of secondary literature and also compared with authentic herbarium sheets belonging to the institutional library of Regional Plant Resource Centre, Bhubaneswar, Odisha. The selected wild edible fruit samples were washed, properly cleaned in running water and then wiped in tissue paper and preserved at -20ºC until analysis. | - Protein estimation was carried out following the method described by (Lowry et al., 1951). - The digested samples were used for elemental analysis. Fe and Zn were determined using AAS and powdered form of fruit sample was used for estimation of Ca using Flame photometer. | - |
| 1. (Nazarudeen, 2010) | South | - Local indigenous fruits were identified using field surveys and the fruit samples for nutritional analysis were collected fresh from the forest. Flowering and fruiting specimens were also collected as vouchers for herbarium that on processing were correctly identified and matched with authentically identified sheets deposited in Botanical laboratory. | - The digested samples were used for elemental analysis. Fe and Zn were determined using AAS and powdered form of fruit sample was used for estimation of Ca using Flame photometer. | - |
| 1. (Padhan et al., 2020) | East | - The mature tubers of different species were collected from the medicinal plant garden of the Department of Biodiversity and Conservation of Natural Resources, Central University of Orissa, Koraput, India, which were all grown with the same climatic and agronomic condition. - Uniform sized tubers of each species were directly sown in 30 kg polythene bags filled with a mixture of farm soil collected from the experimental garden of Central University of Orissa and farmyard manure (cow dung) with a 3:1 ratio. After collection, the tubers were washed, peeled, cut into slices and shade dried at 25 ± 2 °C for 4–6 days. The dried tuber samples were mechanically ground in a pulverizer (SK-03, Global Instrumentation, Visakhapatnam, India) into powder and sieved (2 mm particle size) and stored in airtight containers for a maximum of 8 wk. | - The crude fat contents of the samples were determined by continuous extraction in a lipid extractor (AOAC International and Latimer, 2012)The crude protein was determined using Kjeldahl method. - Vitamin C content was estimated using the method of (Omaye et al., 1979). | - The phenol content was determined using the method of (Sadasivam, and Manickam, 2008) Total tannin content was determined using the Folin Dennis spectrophotometric procedure described by of (Sadasivam, and Manickam, 2008). The saponin content was determined using the method of (Nahapetian and Bassiri, 1975). The α-amylase inhibitor was evaluated using the spectrophotometric method of (Alonso et al., 1998). Trypsin inhibitor activity (TIA) was determined using the method of (Smith et al., 1980). |
| 1. (Panmei et al., 2016) | North-east | - Extensive field surveys were carried out in 10 villages and 4 local market of the district. The selection of plants for nutritional analysis was based on the preferences and recommendation by the local community, market demand and availability in the natural habitat. Plants were collected, photographed and herbarium specimens were prepared adopting standard methodology. Collected plants were identified with the help of secondary literature, verified with International Plant Names Index, The Plant Lists and Tropicos. The Vouchers specimens were deposited in the herbarium of Department of Forestry, NERIST Arunachal Pradesh. | - Crude Protein was determined by micro-Kjeldahl method. Crude fat in plant samples was determined by extracting a known weight of powdered plant material with petroleum ether using Soxhlet apparatus (Sadasivam, and Manickam, 2008) - Vitamin C was estimated by volumetric method using 4 % oxalic acid and dye solution (Sadasivam, and Manickam, 2008). | - |
| 1. (Payum et al., 2015) | North-east | - No methodology reported for food sample collection and taxonomic identification | - Proximate and mineral study was carried out by following methods given by (Thimmaiah, 1999). | - |
| 1. (Pradeepkumar et al., 2015) | South | - Structured and pretested questionnaires were used to identify types of indigenous foods consumed. Food samples were collected from the tribal areas with the help of indigenous people residing in the respective regions. Most of these plants were identified with the help of a taxonomist. Among them, the plants cited by the highest number of people in the ethno botanical questionnaire were selected and analysed. Samples were grouped together after identification (at least about 1,500–2,000 g of each leafy vegetable) and divided in three parts for the analysis. | - Protein and fat were analysed using standard procedures (AOAC: Official Methods of Analysis (Volume 1), 1984) - Standard procedures used for estimation of vitamin A (β-carotene) (AOAC, 1984), vitamin C (Sadasivam, and Manickam, 2008), Ca (Hesse, 1971), Fe (Raghuramulu et al., 1983) and Zn (Elmer, 1982). | - Oxalate was analysed by procedures stated in (Marderosian et al., 1980). |
| 1. (Rajyalakshmi and Geervani, 1994) | South | - Food samples were collected from the households of the study blocks. The collection of foods was done thrice (in different seasons), in order to cover most of the foods consumed by the indigenous people. Samples were collected from 5-10 households at random from all the villages surveyed in about 100-250 g. The foods thus collected were pooled together into one lot and cleaned to remove dirt and sealed in polythene bags and stored in deep freeze for one week to ten days till analysis. - No methodology reported for taxonomic identification | - The foods were analysed for the proximate principles moisture, protein (N x 6.25), crude fat (ether extractives), ash and crude fibre (AOAC, 12^th^ edition, 1975). Energy value was calculated by multiplying the values obtained for carbohydrate, protein and fat with 4, 4 and 9 respectively and adding up the values. - The foods were analysed for Fe (colorimetric) (AOAC, 12^th^ edition, 1975) Ca was estimated by titrimetric method (Hawk et al., 1954). Vitamin B1 was estimated using modified thiochrome method (Leveille, 1972) as modified (Ramasastri, 1976). Vitamin B2 & B3 were determined microbiologically (AOAC, 12^th^ edition, 1975 using L. casei and L. arabinosis respectively. |  |
| 1. (Saha et al., 2014) | North-east | - Information on diversity of indigenous foods, local names, consumption pattern and mode of collection was collected from indigenous people. Samples of each species was collected and made into herbarium and subsequently identified to genera and species with the help of experts of BSI and State Forest Research Institute, Itanagar, Arunachal Pradesh. The herbariums and voucher specimens of species were deposited in the Department of Ecology and Environmental Science, Assam University, Silchar as well as G B Pant Institute of Himalayan Environment and Development, North-east Unit, Itanagar, Arunachal Pradesh. | - The nitrogen content was estimated by the micro Kjeldahl method (Humphries, 1956) and the crude protein content was calculated (N x 6.25). Crude lipid content was determined using Soxhlet apparatus (Official Methods of Analysis, 2005). | - |
| 1. (Seal, 2011) | North-east | - The plant materials were purchased from different local markets. The voucher specimens were preserved in the Plant Chemistry department of author’s office. The plant parts were shed-dried, pulverized and stored in an airtight container, and proximate composition, and mineral contents were carried out in the laboratory. | - Determination of mineral elements (Ca, Fe and Zn) was done through AAS (Indrayan et al., 2005). | - The amount of total phenolic content of crude extracts was determined according to Folin-Ciocalteu procedure (Singleton and Rossi, 1965) |
| 1. (Seal and Chaudhuri, 2014) | North-east | - The plant materials were purchased from different local markets. The voucher specimens were preserved in the Plant Chemistry department of author’s office. The plant parts were shed-dried, pulverized and stored in an airtight container. and proximate composition, and mineral contents were carried out in the laboratory | - The crude protein was determined using micro Kjeldahl method. The crude fat contents of the samples were determined by continuous extraction in a lipid extractor (AOAC, 1990). Energy value was calculated by multiplying the values obtained for carbohydrate, protein and fat with 4, 4 and 9 respectively and adding up the values. - Determination of mineral elements (Ca, Fe and Zn) was done through AAS. | - |
| 1. (Seal et al., 2016) | North-east | - The plant materials were collected from different locations and authenticated in BSI, Howrah, India. The voucher specimens of the plants were preserved at the Plant Chemistry department. The plant parts were shed-dried, pulverized and stored in an airtight container and proximate composition, and mineral contents were carried out in the laboratory. | - The crude protein was determined using micro Kjeldahl method. The crude fat contents of the samples were determined by continuous extraction in a lipid extractor. Energy value was calculated by multiplying the values obtained for carbohydrate, protein and fat with 4, 4 and 9 respectively and adding up the values. | - |
| 1. (Shajeela et al., 2011) | South | - Wild tubers were collected using multistage sampling technique in three consecutive rainy seasons. - No methodology reported on taxonomic identification. | - Nitrogen content was estimated by the micro-Kjeldahl method (Humphries, 1956) and crude protein was calculated (N x 6.25). The contents of crude lipid, was estimated by AOAC (2005) methods (Official Methods of Analysis, 2005). Nitrogen free extract (NFE) was obtained by difference method by subtracting the sum of the protein, fat, ash and fibre from the total dry matter (Muller and Tobin, 1980). The energy value of the tuber was estimated by multiplying the percentages of crude protein, crude lipid and NFE by the factors 16.7, 37.7 and 16.7 respectively (Siddhuraju et al., 2002). - From the triple acid digested sample, Fe, Ca and Zn were analysed using AAS (Isaac and Johnson, 1975). | - The anti-nutritional factors, total free phenols (Sadasivam, and Manickam, 2008), tannins (Burns, 1971), hydrogen cyanide (Jackson, 1967), total oxalate (AOAC: Official Methods of Analysis (Volume 1), 1984, 19), trypsin inhibitor activity (Sadasivam, and Manickam, 2008), and amylase inhibitor activity (Rekha and Padmaja, 2002). |
| 1. (Shantibala et al., 2014) | North-east | - The insects were collected from the ponds and submerged paddy fields. - No methodology reported on taxonomic identification | - Nitrogen was determined by the micro-Kjeldahl method (Pearson, 1976). Total crude fat content was determined by homogenizing and soaking the sample with chloroform-methanol mixture (2:1 v/v). The sample was then dried, giving the lipid content of the sample (Folch et al., 1957). The energy content was estimated using a digital bomb calorimeter, model RSB-3/5/6/6A designed in accordance with the specifications of the Institute of Petroleum and British Standard Institution (IS: 1350- 1966). - The mineral content (Fe, Ca and Zn) was determined after wet digestion of sample with a mixture of sulphuric, nitric, and perchloric acids at the ratio of 1:10:4 using AAS | - Tannin content was determined by the qualitative method using tannic acid as standard solution (Enujiugha and Ayodele‐Oni, 2003). Total phenolic content was estimated by using Folin-Ciocalteu reagent (Kähkönen et al., 1999). |
| 1. (Shantosh and Sarojnalini, 2018) | North-east | - Freshly collected fish samples were bought with proper care in cold chain to Fishery laboratory, Department of Life Science, Manipur University and washed immediately in running tap water and taken as a whole for undergoing various analyses. - No methodology for taxonomic classification | - Analysis of mineral elements Ca, Fe and Zn were done by AAS following the methods of (Elmer, 1982). | - |
| 1. (Sharma et al., 2012) | North | - Local rice landraces were collected from the different diversity rich areas of district Chamba (Himachal Pradesh, India), having different altitudinal range varying from 1100-2400 m. Seeds of an improved white rice variety HPR-2143 which is also cultivated in these areas, were also collected to constitute the material for study. | - Macro and micro minerals were estimated by the methods of (Jackson, 1964) by using AAS (Perkin Elmer Spectrophotometer). |  |
| 1. (Singh et al., 2018) | South | - FGD and PRA techniques were conducted to identify commonly and rarely consumed Indigenous foods. Taxonomic information for families, genus and species was generated by the Regional Station of the Botanical Survey of India, Port Blair, and through the use of information available on the internet or in published documents | - Vitamin C was estimated using the standard procedure described by (Sadasivam, and Manickam, 2008). Ca and Fe were determined with AAS according to procedures described by (Sadasivam, and Manickam, 2008). Total carotenoid content was determined using the equation described by (Lichtenthaler and Buschmann, 2001). | - The anti-nutrients: phytate, nitrate, saponins and oxalate were estimated using titration methods described by (Hassan et al., 2011), and saponin content was determined by standard AOAC methods (AOAC: Official Methods of Analysis (Volume 1), 1984). |
| 1. (Sudheep and Sridhar, 2014) | South | - The mushrooms were collected and identified based on their morphological features and following herbarium specimens of the Department of Botany, Goa University, Goa, India. | - The crude protein (N × 6.25) of the mushroom flours was estimated by micro-Kjeldahl method (Humphries, 1956). The total lipid content was determined gravimetrically by extraction in petroleum ether using Soxhlet extractor (AOAC, 1990). - The mineral content (Ca, Fe and Zn) was determined using AAS (AOAC, 1990). | - |
| 1. (Tag et al., 2014) | North-east | - Quantitative ethnobotanical field methods were followed to assess the diversity and use of ethnobotanical resources and traditional knowledge status of local community. The food samples were identified and authenticated by BSI, Arunachal Regional Centre, Itanagar. Voucher specimen of each species were collected and deposited at Plant Systematic and Pharmacognosy Research Laboratory, Department of Botany, Rajiv Gandhi University, Doimukh, Arunachal Pradesh. Based on ethnobotanical survey report gathered from 66 villages including both rural and sub-urban areas, only six plants with highest use value index (UVI) among total wild edible plants collected were considered for nutrient analysis. | - Crude fat was estimated following the procedure of AOAC (Horwitz and Association of Official Analytical Chemists, 2000). The crude protein was determined using micro Kjeldahl titration method by multiplying the factor 6.25 with nitrogen evaluated by the methods of AOAC (Horwitz and Association of Official Analytical Chemists, 2000) - AOAC method (AOAC, 1990) was followed for determination of Ca. |  |
| 1. (Terangpi and Teron, 2015) | North-east | - Ethnobotanical information on famine indigenous foods was gathered through FGDs, semi-structured interview and personal observations. The plant species, along with its flowering twigs were collected from local forests and photographed. The collected plant specimens were placed in a polythene bags to prevent loss of moisture during transportation to the laboratory. The plant was identified using secondary literature and deposited in the herbarium with voucher specimens in the Department of Life Science, Assam University Diphu campus. | - Estimation of the amount of protein in the sample was done by standard Lowry’s method (Sadasivam, and Manickam, 2008). | - |
| 1. (Vadivel and Janardhanan, 2005) | South | - All the investigated seven wild legume species were collected from different agro-climatic regions of South India. All seed samples were collected from tropical forests of Western Ghats. The wild legumes were identified by the standard methodology reported in literature. | - Nitrogen content in the powdered seed samples was estimated by the micro-kjeldahl method and crude protein was calculated (N × 6.25). Crude lipid was determined by exhaustively extracting 2 g of sample with petroleum ether, using a Soxhlet apparatus. The energy value of the seed was estimated (in kJ) by multiplying the percentages of crude protein, crude lipid, and carbohydrate by the factors 16.7, 37.7, and 16.7, respectively (Siddhuraju et al., 2002). - 500 mg of the ground legume seed was digested with a mixture of 10 ml concentrated nitric acid, 4 ml of 60% perchloric acid, and 1 ml of concentrated sulphuric acid. After cooling, the digest was diluted with 50 ml deionized distilled water, filtered with Whatman No. 42 filter paper and filtrates made up to 100 ml in a glass volumetric flask with deionized distilled water. All the minerals, were analyzed from triple acid digested sample by AAS (Isaac and Johnson, 1975) | - The anti-nutritional compounds, total free phenolics (Bray and Thorpe, 2006) and tannins (Burns, 1971) were quantified. Trypsin inhibitor activity was determined by the enzyme assay of (Kakade et al., 1974) by using benzoyl-DL-arginine-pnitroanilide (BAPNA) as a substrate. |

BSI-Botanical survey of India, U-HPLC-Ultra-High Performance Liquid Chromatography techniques, AAS-Atomic Absorption Spectrometry, FAO-Food and agriculture organization; Ca-Calcium, Fe-Iron, Zn-Zinc

**References**

Agbede, J. O., and Aletor, V. A. (2004). Chemical characterization and protein quality evaluation of leaf protein concentrates from Glyricidia sepium and Leucaena leucocephala. *International Journal of Food Science & Technology* 39, 253–261. doi:10.1111/j.1365-2621.2004.00779.x.

Agrahar-Murugkar, D. (2006). Interventions Using Wild Edibles to Improve the Nutritional Status of Khasi tribal Women. *Human Ecology*, 83–88.

Agrahar-Murugkar, D., and Subbulakshmi, G. (2005a). Nutritional value of edible wild mushrooms collected from the Khasi hills of Meghalaya. *Food Chemistry* 89, 599–603. doi:10.1016/j.foodchem.2004.03.042.

Agrahar-Murugkar, D., and Subbulakshmi, G. (2005b). Nutritive values of wild edible fruits, berries, nuts, roots and spices consumed by the Khasi tribes of India. *Ecology of Food and Nutrition* 44, 207–223. doi:10.1080/03670240590953025.

Allen, S. E., Grimshaw, H. M., Parkinson, J. A., and Quarmby, C. (1974). Chemical analysis of ecological materials. *Chemical analysis of ecological materials.* Available at: https://www.cabdirect.org/cabdirect/abstract/19751431633 [Accessed October 24, 2020].

Alonso, R., Orúe, E., and Marzo, F. (1998). Effects of extrusion and conventional processing methods on protein and antinutritional factor contents in pea seeds. *Food Chemistry* 63, 505–512. doi:10.1016/S0308-8146(98)00037-5.

Anand, H., Das, S., Ganguly, A., and Haldar, P. (2008). Biomass Production of Acridids as Possible Animal Feed Supplement. *Journal of Environment and Sociobiology* 5, 181–190.

AOAC (2002). *Official Methods of Analysis: Lead, Cadmium, Zinc, Copper, and Iron in Foods*. 17th ed. Arlington, VA, USA: Association of Official Analytical Chemists Available at: https://img.21food.cn/img/biaozhun/20100108/177/11285281.pdf [Accessed October 24, 2020].

AOAC International, and Latimer, G. W. (2012). *Official methods of analysis of AOAC International*. Gaithersburg, Md.: AOAC International.

AOAC: Official Methods of Analysis (Volume 1) (1984). 771.

Arinathan, V., Mohan, V. R., and Maruthupandian, A. (2009). NUTRITIONAL AND ANTI-NUTRITIONAL ATTRIBUTES OF SOME UNDERâ€“UTILIZED TUBERS. *Tropical and Subtropical Agroecosystems* 10, 273–278.

Baker, C. J. L. (1952). The determination of oxalates in fresh plant material. *Analyst* 77, 340–344. doi:10.1039/AN9527700340.

Basumatary, S., and Narzary, H. (2017). Nutritional value, phytochemicals and antioxidant property of six wild edible plants consumed by the Bodos of North-East India. *Mediterranean Journal of Nutrition and Metabolism* 10, 259–271. doi:10.3233/MNM-17168.

Bhardwaj, R., Singh, R. K., Sureja, A. K., Tag, H., and Singh, A. (2009). Nutritionally rich wild vegetables of tribal communities of Northeast India: gaining insights on valuable traditional biocultural resources. *Acta horticulturae*. Available at: https://agris.fao.org/agris-search/search.do?recordID=US201301615971 [Accessed October 14, 2020].

Bhattacharjee, L., kothari, G., Priya, V., and Nandi, B. K. (2009). “The Bhil food system: links to food security, nutrition and health,” in *Indigenous people’ food systems & well-being: interventions & policies for healthy communities*, 209–230.

Bligh, E. G., and Dyer, W. J. (1959). A rapid method of total lipid extraction and purification. *Can J Biochem Physiol* 37, 911–917. doi:10.1139/o59-099.

Bray, H. G., and Thorpe, W. V. (2006). “Analysis of Phenolic Compounds of Interest in Metabolism,” in *Methods of Biochemical Analysis* (John Wiley & Sons, Ltd), 27–52. doi:10.1002/9780470110171.ch2.

Brouwer, I. A., van Dusseldorp, M., West, C. E., Meyboom, S., Thomas, C. M., Duran, M., et al. (1999). Dietary folate from vegetables and citrus fruit decreases plasma homocysteine concentrations in humans in a dietary controlled trial. *J Nutr* 129, 1135–1139. doi:10.1093/jn/129.6.1135.

Bureau of Indian Standards (1973). *IS 7219: Method for determination of protein in foods and feeds*. Available at: http://archive.org/details/gov.in.is.7219.1973 [Accessed October 24, 2020].

Burns, R. E. (1971). Method for Estimation of Tannin in Grain Sorghum1. *Agronomy Journal* 63, 511–512. doi:10.2134/agronj1971.00021962006300030050x.

Chakravorty, J., Ghosh, S., Jung, C., and Meyer-Rochow, V. B. (2014). Nutritional composition of Chondacris rosea and Brachytrupes orientalis: Two common insects used as food by tribes of Arunachal Pradesh, India. *Journal of Asia-Pacific Entomology* 17, 407–415. doi:10.1016/j.aspen.2014.03.007.

Chakravorty, J., Ghosh, S., and Meyer-Rochow, V. B. (2011). Chemical composition of Aspongopus nepalensis Westwood 1837 (Hemiptera; Pentatomidae), a common food insect of tribal people in Arunachal Pradesh (India). *Int J Vitam Nutr Res* 81, 49–56. doi:10.1024/0300-9831/a000050.

Chopra, S. L., and Kanwar, J. S. (1991). *Analytical Agricultural Chemistry*. New Delhi: Kalyani Publishers.

Chyne, D. A. L., Ananthan, R., and Longvah, T. (2019). Food compositional analysis of Indigenous foods consumed by the Khasi of Meghalaya, North-East India. *Journal of Food Composition and Analysis* 77, 91–100. doi:10.1016/j.jfca.2019.01.008.

Das, S. K., Mandal, A., Datta, A. K., Das, D., Paul, R., Saha, A., et al. (2015). Identification of Wild Edible Mushrooms from Tropical Dry Deciduous Forest of Eastern Chota Nagpur Plateau, West Bengal, India. *Proc. Natl. Acad. Sci., India, Sect. B Biol. Sci.* 85, 219–232. doi:10.1007/s40011-014-0330-y.

Elmer, P. (1982). Analytical Methods for Atomic Absorption Spectroscopy. USA: Perkin-Elmer Corporation Available at: http://www1.lasalle.edu/~prushan/Intrumental%20Analysis_files/AA-Perkin%20Elmer%20guide%20to%20all!.pdf [Accessed October 25, 2020].

Enujiugha, V. N., and Ayodele‐Oni, O. (2003). Evaluation of nutrients and some anti-nutrients in lesser-known, underutilized oilseeds. *International Journal of Food Science & Technology* 38, 525–528. doi:10.1046/j.1365-2621.2003.00698.x.

Folch, J., Lees, M., and Sloane Stanley, G. H. (1957). A simple method for the isolation and purification of total lipides from animal tissues. *J Biol Chem* 226, 497–509.

Food Hygiene, Sampling and Analysis Sectional Committee (2005). IS 5838 (1970): Methods for estimation of Vitamin C in foodstuffs. New Delhi: Indian Standard Institution.

Ganguly, A., Chakravorty, R., Das, M., Gupta, M., Mandal, D. K., Haldar, P., et al. (2013). A preliminary study on the estimation of nutrients and anti-nutrients in Oedaleus abruptus (Thunberg) (Orthoptera: Acrididae). *International Journal of Nutrition and Metabolism* 5, 50–65.

Ghosh-Jerath, S., Kapoor, R., Barman, S., Singh, G., Singh, A., Downs, S., et al. (2021). Traditional Food Environment and Factors Affecting Indigenous Food Consumption in Munda Tribal Community of Jharkhand, India. *Front. Nutr.* 7. doi:10.3389/fnut.2020.600470.

Ghosh-Jerath, S., Kapoor, R., Singh, A., Downs, S., Barman, S., and Fanzo, J. (2020). Leveraging Traditional Ecological Knowledge and Access to Nutrient-Rich Indigenous Foods to Help Achieve SDG 2: An Analysis of the Indigenous Foods of Sauria Paharias, a Vulnerable Tribal Community in Jharkhand, India. *Front. Nutr.* 7, 61. doi:10.3389/fnut.2020.00061.

Ghosh-Jerath, S., Singh, A., Kamboj, P., Goldberg, G., and Magsumbol, M. S. (2015). Traditional Knowledge and Nutritive Value of Indigenous Foods in the Oraon Tribal Community of Jharkhand: An Exploratory Cross-sectional Study. *Ecology of Food and Nutrition*. Available at: https://www.tandfonline.com/doi/abs/10.1080/03670244.2015.1017758 [Accessed June 10, 2019].

Ghosh-Jerath, S., Singh, A., Magsumbol, M. S., Kamboj, P., and Goldberg, G. (2016). Exploring the Potential of Indigenous Foods to Address Hidden Hunger: Nutritive Value of Indigenous Foods of Santhal Tribal Community of Jharkhand, India. *J Hunger Environ Nutr* 11, 548–568. doi:10.1080/19320248.2016.1157545.

Gopalan, C., Rama Sastri, B. V., and Balasubramanian, S. C. (1978). *Nutritive value of Indian foods*. Hyderabad, India: National Institute of Nutrition, Indian Council of Medical Research Available at: http://catalog.hathitrust.org/api/volumes/oclc/5950952.html [Accessed June 16, 2019].

Gupta, S., Jyothi Lakshmi, A., Manjunath, M. N., and Prakash, J. (2005). Analysis of nutrient and antinutrient content of underutilized green leafy vegetables. *LWT - Food Science and Technology* 38, 339–345. doi:10.1016/j.lwt.2004.06.012.

Hassan, L. G., Umar, K. J., Dangoggo, S. M., and A.S, M. (2011). Anti-nutrient Composition and Bioavailability Prediction as Exemplified by Calcium, Iron and Zinc in Melocia corchorifolia Leaves. *Pakistan Journal of Nutrition* 10. doi:10.3923/pjn.2011.23.28.

Hawk, P. P., Oser, B. L., and Summerson, W. H. (1954). Practical Physiological Chemistry. *J. Phys. Chem.* 51, 1214–1215. doi:10.1021/j150455a015.

Hesse, P. R. (1971). *A textbook of soil chemical analysis*. London: J. Murray.

Horo, S., and Topno, S. (2015). Study and analysis of nutritional value of some wild and semi wild edible plants consumed by “HO” tribes of W. Singhbhum district, Jharkhand, India. *Int. J. Herb. Med.* 3, 25–32.

Horwitz, W. and Association of Official Analytical Chemists (2000). *Official methods of analysis of AOAC International*. Gaithersburg, Md.: Association of Official Analytical Chemists Available at: http://books.google.com/books?id=TDAbAQAAMAAJ [Accessed October 24, 2020].

Horwitz, W., Chichilo, P., and Reynolds, H. (1970). Official methods of analysis of the Association of Official Analytical Chemists. *Official methods of analysis of the Association of Official Analytical Chemists.* Available at: https://www.cabdirect.org/cabdirect/abstract/19720492404 [Accessed October 23, 2020].

Horwitz, W., Latimer, G. W., and Association of Official Analytical Chemists International (2006). *Official methods of analysis of AOAC International*. Gaithersburg, Maryland: AOAC International.

Humphries, E. C. (1956). “Mineral Components and Ash Analysis,” in *Modern Methods of Plant Analysis / Moderne Methoden der Pflanzenanalyse* Modern Methods of Plant Analysis / Moderne Methoden der Pflanzenanalyse., eds. K. Paech and M. V. Tracey (Berlin, Heidelberg: Springer), 468–502. doi:10.1007/978-3-642-80530-1_17.

Indian Standards 4684–1975 (2000). Available at: https://www.services.bis.gov.in:8071/php/BIS/bisconnect/pow/is_details?IDS=MTE0Mjk%3D [Accessed October 24, 2020].

Indrayan, A. K., Sharma, S., Durgapal, D., Kumar, N., and Kumar, M. (2005). Determination of nutritive value and analysis of mineral elements for some medicinally valued plants from Uttaranchal. *Current Science* 89, 1252–1255.

Isaac, R. A., and Johnson, W. C. (1975). Collaborative study of wet and dry ashing techniques for the elemental analysis of plant tissue by atomic absorption spectrophotometry. *J Assoc Off Anal Chem*. Available at: https://agris.fao.org/agris-search/search.do?recordID=US201302737874 [Accessed October 23, 2020].

Jackson, M. L. (1964). *Soil Chemical Analysis*. Prentice-Hall.

Jackson, M. L. (1967). Cyanide in plant tissue. In: Soil chemical analysis. ,. *Asia Publishing House*, 337. doi:10.1002/jpln.19590850311.

Jagota, S. K., and Dani, H. M. (1982). A new colorimetric technique for the estimation of vitamin C using Folin phenol reagent. *Analytical Biochemistry* 127, 178–182. doi:10.1016/0003-2697(82)90162-2.

Jain, A. K., and Tiwari, P. (2012). Nutritional value of some traditional edible plants used by tribal communities during emergency with reference to Central India. *Indian Journal of Traditional Knowledge* 11, 7.

Jana, S. (2004). Dietary Practices of the Lodhas and the Nutrient Content of Unconventional Foods Consumed: An Anthropological Study. *Vanyajati, New Delhi* XLXII, 29–38.

Johnsy, G., Sargunam, S. D., Dinesh, M. G., and Kaviyarasan, V. (2011). Nutritive Value of Edible Wild Mushrooms Collected from the Western Ghats of Kanyakumari District. *Botany Research International* 4, 69–74.

Joint FAO/WHO Codex Alimentarius Commission, World Health Organization, Food and Agriculture Organization of the United Nations, and Joint FAO/WHO Codex Alimentarius Commission eds. (2007). *Food labelling*. 5th ed. Rome: World Health Organization : Food and Agriculture Organization of the United Nations.

Kadiri, M., and Fasidi, I. O. (2012). Variations in chemical composition chlorophyllum molybodies (Mayerex. Fr) Massres pleurotus tuberregium (fries) during fruitbody development. *Nigerian J. Science* 24, 86–89.

Kähkönen, M. P., Hopia, A. I., Vuorela, H. J., Rauha, J. P., Pihlaja, K., Kujala, T. S., et al. (1999). Antioxidant activity of plant extracts containing phenolic compounds. *J Agric Food Chem* 47, 3954–3962. doi:10.1021/jf990146l.

Kakade, M. L., Rackis, J. J., McGhee, J. E., and Puski, G. (1974). Determination of trypsin inhibitor activity of soy products: a collaborative analysis of an improved procedure. Available at: https://pubag.nal.usda.gov/catalog/31302 [Accessed October 24, 2020].

Laddha, C., Itankar, P., and Tauqeer, M. (2015). Nutritional and Phytochemical Analysis of Wild Leafy Vegetable Hibiscus sabdariffa L. (Ambadi Bhaji) Used By the Tribes of Bhiwapur Tehsil Nagpur District, India. *Indian Jounral of Researches in Biosciences, Agriculture & Technology*, 6.

Leveille, G. A. (1972). Modified thiochrome procedure for the determination of urinary thiamin. *Am J Clin Nutr* 25, 273–274. doi:10.1093/ajcn/25.3.273.

Lichtenthaler, H. K., and Buschmann, C. (2001). Chlorophylls and Carotenoids: Measurement and Characterization by UV-VIS Spectroscopy. *Current Protocols in Food Analytical Chemistry* 1, F4.3.1-F4.3.8. doi:10.1002/0471142913.faf0403s01.

Longvah, T., and Deosthale, Y. G. (1991). Chemical and nutritional studies on hanshi (perilla frutescens), a traditional oilseed from northeast india. *J Am Oil Chem Soc* 68, 781–784. doi:10.1007/BF02662172.

Longvah, T., and Deosthale, Y. G. (1998). Compositional and nutritional studies on edible wild mushroom from northeast India. *Food Chemistry* 63, 331–334. doi:10.1016/S0308-8146(98)00026-0.

Longvah, T., Vooradi Sathya Sai, P., Rajendran, A., Kharkhonger, G. C., and Rangad, C. (2020). In situ nutrient variability in rice landraces from garo Hills, meghalaya in North East India. *Journal of Food Composition and Analysis* 92, 103543. doi:10.1016/j.jfca.2020.103543.

Loukrakpam, B., Rajendran, A., Chyne, D. A. L., and Longvah, T. (2019). 12th IFDC 2017 Special Issue - Nutrient and phytonutrient profiles of some indigenous vegetables of Manipur, Northeast India. *Journal of Food Composition and Analysis* 79, 12–22. doi:10.1016/j.jfca.2019.03.003.

Lowry, O. H., Rosebrough, N. J., Farr, A. L., and Randall, R. J. (1951). Protein measurement with the Folin phenol reagent. *J Biol Chem* 193, 265–275.

MacLean, W. C., Warwick, P., and Food and Agriculture Organization of the United Nations eds. (2003). *Food energy: methods of analysis and conversion factors: report of a technical workshop, Rome, 3-6 December 2002*. Rome: Food and Agriculture Organization of the United Nations.

Mahadkar, S., Valvi, S., and Rathod, V. (2012). Nutritional assessment of some selected wild edible plants as a good source of mineral. *Asian Journal of Plant Science and Research* 2, 468–472.

Mahapatra, A. K., Mishra, S., Basak, U. C., and Panda, P. C. (2012). Nutrient Analysis of Some Selected Wild Edible Fruits of Deciduous Forests of India: an Explorative Study towards Non Conventional Bio-Nutrition. 7.

Mandal, A. B., Khatta, V. K., Biswas, A., Sihag, S., and Singh, R. (2018). *Analytical Techniques in Animal Nutrition*. Satish Serial Publishing House.

Marderosian, A. D., Beutler, J., Pfendner, W., Chambers, J., Yoder, R., Weinsteiger, E., et al. (1980). Nitrate and oxalate content of vegetable amaranth. *Rodale Research Report*. Available at: https://www.cabdirect.org/cabdirect/abstract/19806735440 [Accessed October 26, 2020].

Medak, B., and Singha, L. (2016). Nutritional Profile of a few selected wild edible plants of Papum Pare district of Arunachal Pradesh. *Journal of Bioresources* 3, 17–21.

Medak, B., and Singha, L. (2018). Trace elements and antioxidant activity of six wild edible plants that are widely consumed by ethnic tribes of Arunachal Pradesh, India. *Indian Journal Of Agricultural Research* 52, 85–88. doi:10.18805/IJARe.A-4755.

Mishra, N., Hazarika, N. C., Narain, K., and Mahanta, J. (2003). Nutritive value of non-mulberry and mulberry silkworm pupae and consumption pattern in Assam, India. *Nutrition Research* 23, 1303–1311. doi:10.1016/S0271-5317(03)00132-5.

Mohan, V. R., and Kalidass, C. (2010). NUTRITIONAL AND ANTINUTRITIONAL EVALUATION OF SOME UNCONVENTIONAL WILD EDIBLE PLANTS. *Tropical and Subtropical Agroecosystems* 12, 495–506.

Muller, H. G., and Tobin, G. (1980). *Nutrition and food processing*. London: Croom Helm. Available at: https://www.cabdirect.org/cabdirect/abstract/19810468076 [Accessed October 24, 2020].

Nahapetian, A., and Bassiri, A. (1975). Changes in concentrations and interrelationships of phytate, phosphorus, magnesium, calcium, and zinc in wheat during maturation. *J Agric Food Chem* 23, 1179–1182. doi:10.1021/jf60202a014.

Narzary, H., and Basumatary, A. (2015). Proximate and vitamin C analysis of wild edible plants consumed by Bodos of Assam, India. *J Mol Pathophysiol* 4, 128. doi:10.5455/jmp.20151111030040.

Nayak, J., and Basak, U. (2015). Analysis of some nutritional properties in eight wild edible fruits of Odisha, India. *International Journal of current science* 14, 55–62.

Nazarudeen, A. (2010). Nutritional composition of some lesser-known fruits used by the ethnic communities and local folks of Kerala. *Indian Journal of Traditional Knowledge* 9, 398–402.

Nielsen, S. S. ed. (2010). *Food Analysis*. Boston, MA: Springer US doi:10.1007/978-1-4419-1478-1.

Official Methods of Analysis (2005). 18th edition. AOAC International Available at: http://sutlib2.sut.ac.th/sut_contents/H125800.pdf [Accessed October 24, 2020].

Omaye, S. T., Turnbull, J. D., and Sauberlich, H. E. (1979). Selected methods for the determination of ascorbic acid in animal cells, tissues, and fluids. *Methods Enzymol* 62, 3–11. doi:10.1016/0076-6879(79)62181-x.

Padhan, B., Biswas, M., and Panda, D. (2020). Nutritional, anti-nutritional and physico-functional properties of wild edible yam (Dioscorea spp.) tubers from Koraput, India. *Food Bioscience* 34, 100527. doi:10.1016/j.fbio.2020.100527.

Panmei, R., Gajurel, P., and Singh, B. (2016). ETHNOBOTANY AND NUTRITIONAL VALUES OF SOME SELECTED WILD EDIBLE PLANTS USED BY RONGMEI TRIBE OF MANIPUR NORTHEAST INDIA. *International Journal of Applied Biology and Pharmaceutical Industry* 7, 1–9. doi:10.21276/ijabpt.2016.7.4.1.

Payum, T., Das, A., and Shankar, R. (2015). Phytochemistry, Pharmacognosy and Nutritional Composition of Allium Hookeri: an Ethnic Food Plant Used Among Adi Tribe of Arunachal Pradesh, India. *American Journal of PharmaTech Research* 5, 465–477.

Pearson, D. (1976). *The Chemical Analysis of Foods*. Churchill Livingstone.

Phillips, K. M., Tarragó-Trani, M. T., Gebhardt, S. E., Exler, J., Patterson, K. Y., Haytowitz, D. B., et al. (2010). Stability of vitamin C in frozen raw fruit and vegetable homogenates. *Journal of Food Composition and Analysis* 23, 253–259. doi:10.1016/j.jfca.2009.08.018.

Pradeepkumar, T., Indira, V., and Sankar, M. (2015). Nutritional Evaluation of Wild Leafy Vegetables Consumed by Tribals in the Wayanad District of Kerala. *Proc. Natl. Acad. Sci., India, Sect. B Biol. Sci.* 85, 93–99. doi:10.1007/s40011-013-0271-x.

Rader, J. I., Weaver, C. M., and Angyal, G. (1998). Use of a microbiological assay with tri-enzyme extraction for measurement of pre-fortification levels of folates in enriched cereal-grain products. *Food Chemistry* 62, 451–465. doi:10.1016/S0308-8146(98)00089-2.

Raghuramulu, N., Madhavan Nair, K., and Kalyanasundaram, S. (1983). *A manual of laboratory techniques.* Hyderabad: National Institute of Nutrition, Indian Council of Medical Research.

Rajyalakshmi, P., and Geervani, P. (1994). Nutritive value of the foods cultivated and consumed by the tribals of south India. *Plant Foods Hum Nutr* 46, 53–61. doi:10.1007/BF01088461.

Ramasastri, B. V. (1976). A simple method for estimation of thiamine in foods using base-exchange on sand instead of Decalso. *Int J Vitam Nutr Res* 46, 403–411.

Ranganna, S., and Ranganna, S. (2003). *Handbook of analysis and quality control for fruit and vegetable products*. New Delhi: Tata McGraw-Hill.

Rekha, M. R., and Padmaja, G. (2002). Alpha-amylase inhibitor changes during processing of sweet potato and taro tubers. *Plant Foods Hum Nutr* 57, 285–294. doi:10.1023/A:1021837115267.

Sadasivam, S., and Manickam, A. (2008). *Biochemical Methods*. New Age International Available at: https://www.newagepublishers.com/servlet/nagetbiblio?bno=000091 [Accessed October 23, 2020].

Saha, D., Sundriyal, M., and Sundriyal, R. C. (2014). Diversity of food composition and nutritive analysis of edible wild plants in a multi-ethnic tribal land, Northeast India: an important facet for food supply. *IJTK* 13, 698–705.

Seal, T. (2011). Determination of Nutritive Value, Mineral Contents and Antioxidant Activity of Some Wild Edible Plants from Meghalaya State, India. *Asian Journal of Applied Sciences* 4, 238–246.

Seal, T., and Chaudhuri, K. (2014). Ethnobotanical importance and nutritional potential of wild edible fruits of Meghalaya state in India. *Journal of Chemical and Pharmaceutical Research* 6. Available at: https://www.jocpr.com/abstract/ethnobotanical-importance-and-nutritional-potential-of-wild-edible-fruits-of-meghalaya-state-in-india-5318.html [Accessed October 16, 2020].

Seal, T., Pillai, B., and Chaudhuri, K. (2016). Evaluation of Nutritional Potential of Five Unexplored Wild Edible Plants Consumed by the Tribal People of Arunachal Pradesh State in India. *Journal of Food and Nutrition Research* 5, 1–5. doi:10.12691/jfnr-5-1-1.

Sebestianus Lakra, S. J. (2019). Sustainable Resource Management through Indigenous Knowledge and Practices – A Case of Food Security among the Baiga Tribe in India. *EJSD* 8, 233–233. doi:10.14207/ejsd.2019.v8n4p233.

Shahidan, N., j.irwandi, Othman, R., and Hashim, Y. (2012). Scheme of obtaining β-carotene standard from pumpkin (Cucurbita moschata) flesh. *International Food Research Journal* 19, 531–535.

Shajeela, P. S., Mohan, V. R., Jesudas, L. L., and Soris, P. T. (2011). NUTRITIONAL AND ANTINUTRITIONAL EVALUATION OF WILD YAM (Dioscorea spp.). *Tropical and Subtropical Agroecosystems* 14, 723–730.

Shantibala, T., Lokeshwari, R. K., and Debaraj, H. (2014). Nutritional and antinutritional composition of the five species of aquatic edible insects consumed in Manipur, India. *J Insect Sci* 14. doi:10.1093/jis/14.1.14.

Shantosh, M., and Sarojnalini, C. (2018). Nutritional Quality of Three Cobitid Fishes of Manipur, India: With Special Reference to Essential Mineral Elements. *International Journal of Scientific Research in Biological Sciences* 5, 24–33.

Sharma, J. K., Sharma, T. R., and Sharma, S. K. (2012). Comparative study on macro and micro minerals composition of selective red rice landraces from Chamba district of Himachal Pradesh-India. *World Journal of Agricultural Sciences* 8, 378–380.

Siddhuraju, P., Becker, K., and Makkar, H. P. S. (2002). Chemical composition, protein fractionation, essential amino acid potential and antimetabolic constituents of an unconventional legume, Gila bean (Entada phaseoloides Merrill) seed kernel. *Journal of the Science of Food and Agriculture* 82, 192–202. doi:10.1002/jsfa.1025.

Singh, S., Singh, L. B., Singh, D. R., Chand, S., Zamir Ahmed, S. K., Singh, V. N., et al. (2018). Indigenous underutilized vegetables for food and nutritional security in an island ecosystem. *Food Sec.* 10, 1173–1189. doi:10.1007/s12571-018-0840-1.

Singleton, V. L., and Rossi, J. A. (1965). Colorimetry of Total Phenolics with Phosphomolybdic-Phosphotungstic Acid Reagents. *Am J Enol Vitic.* 16, 144–158.

Smith, C., Megen, W. van, Twaalfhoven, L., and Hitchcock, C. (1980). The determination of trypsin inhibitor levels in foodstuffs. *Journal of the Science of Food and Agriculture* 31, 341–350. doi:10.1002/jsfa.2740310403.

Sudheep, N. M., and Sridhar, K. R. (2014). Nutritional composition of two wild mushrooms consumed by the tribals of the Western Ghats of India. *Mycology* 5, 64–72. doi:10.1080/21501203.2014.917733.

Tag, H., Tsering, J., Hui, P. K., Gogoi, B. J., and Veer, V. (2014). Nutritional Potential and Traditional Uses of High Altitude Wild Edible Plants in Eastern Himalayas, India. *International Journal of Nutrition and Food Engineering* 8, 238–243.

Taussky, H. H., and Shorr, E. (1953). A microcolorimetric method for the determination of inorganic phosphorus. *J Biol Chem* 202, 675–685.

Tee, E. S., Goh, A. H., and Khor, S. C. (1995). Carotenoid composition and content of legumes, tubers and roots by HPLC. *Malays J Nutr* 1, 51–61.

Terangpi, R., and Teron, R. (2015). Nutritional consideration of three important emergency food plants studied among Karbi Tribe of North East India. *Journal of Scientific and Innovative Research* 4, 138–141.

Thimmaiah, S. K. (1999). *Standard Methods of Biochemical Analysis*. Kalyani Pub.

Toth, S. J., Prince, A. L., Wallace, A., and Mikkelsen, D. S. (1948). RAPID QUANTITATIVE DETERMINATION OF EIGHT MINERAL ELEMENTS IN PLANT TISSUE BY A SYSTEMATIC PROCEDURE INVOLVING USE OF A FLAME PHOTOMETER. *Soil Science* 66, 459–466.

Vadivel, V., and Janardhanan, K. (2005). Nutritional and antinutritional characteristics of seven South Indian wild legumes. *Plant Foods Hum Nutr* 60, 69–75. doi:10.1007/s11130-005-5102-y.

Valls, F., Sancho, M. T., Fernández-Muiño, M. A., and Checa, M. A. (2001). Determination of vitamin B(6) in cooked sausages. *J Agric Food Chem* 49, 38–41. doi:10.1021/jf0003202.
