# Supplementary material for: Indigenous Foods of India: A Comprehensive Narrative Review of Nutritive Values, Antinutrient Content and Mineral Bioavailability of Traditional Foods Consumed by Indigenous Communities of India
Source: Front Sustain Food Syst. Author manuscript; Available in PMC 2022 May 22. (PMC7612755; doi:10.3389/fsufs.2022.696228)
Supplement: Table 2 [file EMS145028-supplement-Table_2.pdf]

Supplementary Table 2: Nutritive values of indigenous foods of India (n=508)

Common name	Botanical name	Vernacular name	Energy (kcal /100g)	Protein (g/100g)	Fat (g/ 100g)	Ca (mg /100g)	Iron (mg/ 100g)	Zinc (mg/ 100g)	Vit A (µg/ 100g)	Vit C (mg /100g)	Vit B1 (mg/ 100g)	Vit B2 (mg/ 100g)	Vit B3 (mg/ 100g)	Vit B6 (mg/ 100g)	Vit B9 (µg/ 100g)	Reference
							CEREALS									
**White Rice**	*Oryza sativa* L.	*Jadhan*	351	7.3	0.8	7	1.7	1	-	-	0.7	-	-	-	-	(Ghosh-Jerath et al., 2016)
		Pundi Goda	351	4.3	1.3	20.6	0.4	0.1	-	-	0.4	0.1	-	-	3	(Ghosh-Jerath et al., 2021)
		Dhan	343	7.9	0.5	7.5	0.7	1.2	28	-	0.1	0.1	1.7	0.6	9	(Longvah et al., 2017)
**Red rice**	*Oryza sativa* L.	*Kba-lwai*	340	8.3	0.5	10.6	2.8	1.1			0.2	0.04	0.9	0.03	123	(Chyne et al., 2019)
		Kba-bakut	344	7.1	0.7	11.5	3.02	1.6			0.2	0.1	1.1	0.1	1203	(Chyne et al., 2019)
		Kba-baswoit	356	8.4	0.7	12	3.8	1.7			0.2	0.03	1.2	0.1	128	(Chyne et al., 2019)
		Laldhan	364	6.3	0.7	21	6.1	-	-	-	0.3	-	3.3	-	-	(Rajyalakshmi and Geervani, 1994)
		Lalhat, desi	363	6.6	1.6	14	1.4	0.8	-	3	-	-	-	-	14	(Ghosh-Jerath et al., 2015)
		Karad	-	-	-	-	0.04	0.003	-	-	-	-	-	-	-	(Sharma et al., 2012)
		HPR-2143	-	-	-	-	0.03	0.003	-	-	-	-	-	-	-	(Sharma et al., 2012)
		Matali	-	-	-	-	0.1	0.004	-	-	-	-	-	-	-	(Sharma et al., 2012)
		Begmi	-	-	-	-	0.04	0.003	-	-	-	-	-	-	-	(Sharma et al., 2012)
		Bhrigu dhan	-	-	-	-	0.04	0.004	-	-	-	-	-	-	-	(Sharma et al., 2012)
		Sukara	-	-	-	-	0.03	0.003	-	-	-	-	-	-	-	(Sharma et al., 2012)
		Chohartu	-	-	-	-	0.05	0.004	-	-	-	-	-	-	-	(Sharma et al., 2012)
**Brown rice**	*Oryza sativa* L.	*Gopalbhok*	340	6.4	2.4	15.6	0.9	1.9	-	-	0.3	0.1	3.2	0.1	-	(Longvah et al., 2020)
		Hathidat gipok	345	6.6	3.9	8.2	1.6	2.2	-	-	0.2	0.1	1.7	0.1	-	(Longvah et al., 2020)
		Hathidatgitc hak	345	8.8	3.3	8.4	2	2.3	-	-	0.2	0.1	3.8	0.1	-	(Longvah et al., 2020)
		Hati bandha	343	6.2	2.8	11.9	1.2	2.4	-	-	0.3	0.1	3.1	0.04	-	(Longvah et al., 2020)
		Jahagipok	350	7.3	3.1	9.1	1.1	2.4	-	-	0.2	0.1	3.8	0.1	-	(Longvah et al., 2020)
		Jahagisim	348	7.8	2.7	8.4	1.2	2.2	-	-	0.3	0.1	3.1	0.2	-	(Longvah et al., 2020)
		Kala jira jaha	338	6.6	2.3	13.02	0.9	1.5	-	-	0.4	0.1	2.2	0.1	-	(Longvah et al., 2020)
		Khisore	336	8.6	2.7	7.1	1.1	2.6	-	-	0.3	0.1	2.1	0.1	-	(Longvah et al., 2020)
		Kutchigisim	343	9.02	3.3	6.7	1.3	2.4	-	-	0.2	0.1	3.9	0.1	-	(Longvah et al., 2020)
		Maigothi	350	5.4	3.4	4.8	2.2	1.9	-	-	0.2	0.1	3.8	0.1	-	(Longvah et al., 2020)
		Mandami	328	7.7	2.2	7.6	0.9	2.4	-	-	0.3	0.1	3.3	0.03	-	(Longvah et al., 2020)

Common name	Botanical name	Vernacular name	Energy (kcal /100g)	Protein (g/100g)	Fat (g/ 100g)	Ca (mg /100g)	Iron (mg/ 100g)	Zinc (mg/ 100g)	Vit A (µg/ 100g)	Vit C (mg /100g)	Vit B1 (mg/ 100g)	Vit B2 (mg/ 100g)	Vit B3 (mg/ 100g)	Vit B6 (mg/ 100g)	Vit B9 (µg/ 100g)	Reference
		Mi katchi	342	8.4	2.3	13.3	0.9	2.4	-	-	0.3	0.1	3.1	0.1	-	(Longvah et al., 2020)
		Mi-bi-sa	336	7.7	2.8	9.7	1.2	2.6	-	-	0.3	0.1	2.02	0.1	-	(Longvah et al., 2020)
		Mibol	341	8.6	3.5	11.7	1.4	2.9	-	-	0.3	0.1	2.5	0.1	-	(Longvah et al., 2020)
		Miganggetl mi chambak	334	7.9	3.1	12.3	1.6	2.6	-	-	0.3	0.1	2.6	0.1	-	(Longvah et al., 2020)
		Mima	341	7.9	2.7	10.7	1.02	2.4	-	-	0.3	0.1	2.8	0.1	-	(Longvah et al., 2020)
		Mima gisim	335	9.2	2.4	12.9	1.4	2.9	-	-	0.3	0.1	2.6	0.1	-	(Longvah et al., 2020)
		Mimittim midokru	334	8.4	3.1	9.6	1.4	3.1	-	-	0.4	0.1	2.1	0.1	-	(Longvah et al., 2020)
		Mimittim sokmil	328	8.5	2.7	12.9	1.6	2.9	-	-	0.3	0.1	2.7	0.2	-	(Longvah et al., 2020)
		Mim-kudep	337	8.9	2.8	12.8	1.4	2.9	-	-	0.3	0.1	2.6	0.2	-	(Longvah et al., 2020)
		Ranga bhok jaha	345	6.9	3.1	13.2	0.9	2.1	-	-	0.3	0.1	2.8	0.03	-	(Longvah et al., 2020)
		Sarangma bolma	332	7.7	2.5	7.4	1.5	2.3	-	-	0.3	0.1	2.4	0.1	-	(Longvah et al., 2020)
		Silgothi	338	6.02	2.9	6.1	1.02	2.3	-	-	0.2	0.1	2.1	0.1	-	(Longvah et al., 2020)
		Taramon	342	6.5	2.4	11.4	0.9	2.1	-	-	0.2	0.1	2.8	0.1	-	(Longvah et al., 2020)
		Kba-stem	346	8.2	0.7	9.9	2.4	1.3	-	-	0.1	0.02	1.1	0.1	65	
**Sticky rice**	*Oryza sativa var. glutinosa*	*Dudh binni*	337	7.3	2.7	13.8	1.8	2.3	-	-	0.3	0.1	2.3	0.1	-	(Longvah et al., 2020)
		Maubinni	342	7.1	2.5	13.2	2.7	2.03	-	-	0.3	0.1	1.4	0.04	-	(Longvah et al., 2020)
		Minil gisim	340	7.4	2.2	7.5	1.4	1.9	-	-	0.2	0.1	3.5	0.1	-	(Longvah et al., 2020)
		Minil gitchak	345	6.9	3.4	6.5	0.8	2.01	-	-	0.2	0.1	2.1	0.2	-	(Longvah et al., 2020)
		Minil jaha	337	6.6	2.4	7.1	0.7	2.1	-	-	0.3	0.1	3.03	0.1	-	(Longvah et al., 2020)
		Minil na,singket	342	6.6	2.5	8.9	0.9	1.7	-	-	0.3	0.1	3.3	0.1	-	(Longvah et al., 2020)
		Minil-michudari	331	8.7	2.6	15.4	1.3	2.9	-	-	0.4	0.1	2.9	0.1	-	(Longvah et al., 2020)
		Raga-binn	333	7.3	2.6	11.2	1.4	1.8	-	-	0.4	0.1	2.5	0.1	-	(Longvah et al., 2020)
		Kba-shulia	346	6.5	0.4	12.1	3.1	1.2	-	-	0.2	0.03	1.01	0.04	145	(Chyne et al., 2019)
**Pearl millet**	*Pennisetum typhoideum* Rich.	*Bajra*	348	11.0	5.4	27.3	6.4	2.8	-	-	0.3	0.2	0.9	0.3	36	(Longvah et al., 2017)
			366	8.4	3.1	27	6.5	-	-	-	0.3	-	3.4	-	-	(Rajyalakshmi and Geervani, 1994)

Common name	Botanical name	Vernacular name	Energy (kcal /100g)	Protein (g/100g)	Fat (g/ 100g)	Ca (mg /100g)	Iron (mg/ 100g)	Zinc (mg/ 100g)	Vit A (µg/ 100g)	Vit C (mg /100g)	Vit B1 (mg/ 100g)	Vit B2 (mg/ 100g)	Vit B3 (mg/ 100g)	Vit B6 (mg/ 100g)	Vit B9 (µg/ 100g)	Reference
**Sorghum**	*Sorghum vulgare* Pers.	*Jowar*	334	10.0	1.7	27.6	3.9	1.9	8	-	0.4	0.1	2.1	0.3	39	(Longvah et al., 2017)
**Maize**	*Zea mays* L.	*Jondra/ Makai/Mak ka*	334	3.6	1.4	8.9	2.5	2.3	186	-	0.3	0.1	2.7	0.3	26	(Longvah et al., 2017)
**Indian goosegrass**	*Eleusine indica* (L.) Gaertn.	*Madiya*	328	7.3	1.3	344	3.9	-	-	-	-	-	-	-	-	(Sebestianus Lakra, 2019)
**Italian millet**	*Setaria italica* (L.) P.Beauv.	*Kangni*	336	12.3	4.3	31	2.8	-	-	-	-	-	-	-	-	(Sebestianus Lakra, 2019)
			357	11.2	2.6	21	10.2	-	-	-	0.2	-	2.3	-	-	(Rajyalakshmi and Geervani, 1994)
**Salharkutki**	NA		-	10.6	4.8	38	16.9	-	-	-	-	-	-	-	-	(Sebestianus Lakra, 2019)
**Little millet**	*Panicum antidotale* Retz.	*Samai (Parboiled)*	350	7.0	1.1	21	8.8	-	-	-	0.3	-	2.1	-	-	(Rajyalakshmi and Geervani, 1994)
		Samayi/Bhadeli Kutki/Gundli /Salkaya	346	10.1	3.9	16.1	1.2	1.8	2	-	0.3	0.1	-	-	36	(Longvah et al., 2017)
**Kodo millet**	*Paspalum scrobiculatum* L.	*Varagu/Varagu*	360	7.0	3.2	22	5.8	-	-	-	0.3	-	3.4	-	-	(Rajyalakshmi and Geervani, 1994)
			332	8.9	2.6	15.3	2.3	1.7	1	-	0.3	0.2	1.5	0.1	40	(Longvah et al., 2017)
**Japenese millet**	Echinochloa frumentacea	*Sanwan/ Sanwa*	307	11.2	2.2	11	15.2	-	-	-	-	-	-	-	-	(Sebestianus Lakra, 2019)
			358	10.9	1.2	20	8.6	-	-	-	0.3	-	2.2	-	-	(Rajyalakshmi and Geervani, 1994)
**Finger millet**	*Eleusine coracana* (L.) Gaertn.	*Tella*	352	7.4	1.8	302	19.3	-	-	-	0.4	-	1.2	-	-	(Rajyalakshmi and Geervani, 1994)
		Punasa	331	7.0	1.8	307	18.8	-	-	-	0.3	-	1.0	-	-	(Rajyalakshmi and Geervani, 1994)
		Burada	347	7.0	1.4	264	8.6	-	-	-	0.3	-	1.0	-	-	(Rajyalakshmi and Geervani, 1994)
		Mandua/Kodde/Naglana	321	7.2	1.9	364	4.6	2.5	2	-	0.4	0.2	1.3	0.1	35	(Longvah et al., 2017)
								NUTS AND LEGUMES								
**Cowpea, brown**			365	24.2	2.3	91	6.1	3.8	10	-	-	-	-	-	18	(Ghosh-Jerath et al., 2016)

Common name	Botanical name	Vernacular name	Energy (kcal /100g)	Protein (g/100g)	Fat (g/ 100g)	Ca (mg /100g)	Iron (mg/ 100g)	Zinc (mg/ 100g)	Vit A (µg/ 100g)	Vit C (mg /100g)	Vit B1 (mg/ 100g)	Vit B2 (mg/ 100g)	Vit B3 (mg/ 100g)	Vit B6 (mg/ 100g)	Vit B9 (µg/ 100g)	Reference
	Vigna catjang (L.) Walp.	*Ghangra/Dangudi/chowli*	320	20.3	1.1	81.7	5.9	3.4	7	-	0.3	0.1	1.6	0.3	231	(Longvah et al., 2017)
**Cowpea, white**	*Dolichos catjang* Burm.f	*Barbatti/Bodi*	320	21.3	1.1	84.1	5	3.6	8	-	0.3	0.1	1.5	0.3	249	(Longvah et al., 2017)
**Hairy wetch**	*Vicia hirsuta* (L.) Gray	*Baturi/Teeri reeti*	361	26.9	1.9	21	7.8	4.1	550	23	-	-	2.0	-	7	(Ghosh-Jerath et al., 2016)
**Horse gram**	*Dolichos biflorus* L	*Kulthi/Kulad*	321	22.0	0.5	269.	8.8	2.7	59	-	0.3	0.2	1.8	0.2	163	(Longvah et al., 2017)
		Kulthi (Black variety)	349	22.2	3.0	263	38.6	-	-	-	0.3	-	3.5	-	-	(Rajyalakshmi and Geervani, 1994)
		Kulthi (white variety)	348	22.8	2.9	351	37.5	-	-	-	0.2	-	3.0	-	-	(Rajyalakshmi and Geervani, 1994)
**Velvet Bean**	*Mucuna pruriens* (L.) DC.	*Kusa*	193	18.3	-	945.4	5.3	1.8	22	3	-	-	-	-	-	(Ghosh-Jerath et al., 2020)
		Dukka chikkudu	401	27.3	8.6	30	8.8	-	-	-	0.1	0.1	1.6	-	-	(Rajyalakshmi and Geervani, 1994)
			383	32.4	5.7	304.5	16.4	2.3								(Vadivel and Janardhanan, 2005)
	Mucuna pruriens var. *utilis* (Wall. ex Wight) L.H.Bailey		381	29.3	6.4	393.4	13.4	6.7								(Vadivel and Janardhanan, 2005)
**Black gram dal**	*Phaseolus mungo* L	*Rambada / Urad dal*	324	23.1	1.7	55.7	4.7	3	10	-	0.2	0.1	1.8	0.2	89	(Longvah et al., 2017)
**Grass pea**	*Lathyrus sativus* L	*Khesari dal*	345	28.2	0.6	90	6.3	-	-	-	-	-	-	-	90	(Longvah et al., 2017)
**Lentil**	*Lens culinaris* Medik.	*Masoor*	322	24.3	0.8	44.32	7.1	3.6	6.3	-	0.3	0.2	1.8	0.2	50	(Longvah et al., 2017)
**Kidney beans**	*Phaseolus vulgaris* L.	*Rajma*	346	22.9	1.3	260	-	-	-	-	-	-	-	-	-	(Sebestianus Lakra, 2019)
**Red gram**	*Cajanus cajan* (L.) Millsp.	*Rahar/Rehad/Arhar/Tuvar*	326	21.7	1.6	126	6.1	2.6	127	-	0.5	0.1	2.1	0.2	108	(Longvah et al., 2017)
		Dry land cultivated	345	20.3	2.7	118	4.6	-	-	-	0.4	-	1.5	-	-	(Rajyalakshmi and Geervani, 1994)

Common name	Botanical name	Vernacular name	Energy (kcal /100g)	Protein (g/100g)	Fat (g/ 100g)	Ca (mg /100g)	Iron (mg/ 100g)	Zinc (mg/ 100g)	Vit A (µg/ 100g)	Vit C (mg /100g)	Vit B1 (mg/ 100g)	Vit B2 (mg/ 100g)	Vit B3 (mg/ 100g)	Vit B6 (mg/ 100g)	Vit B9 (µg/ 100g)	Reference
		Hill cultivated	359	20.3	2.6	77	7.2	-	-	-	0.4	-	1.3	-	-	(Rajyalakshmi and Geervani, 1994)
**Field Bean**	*Dolichos lablab* L.	Field bean	347	24.9	0.8	60	9.3	-	-	-	-	-	-	-	-	(Sebestianus Lakra, 2019)
		Field bean (Black variety)	276	19.9	0.9	78.2	4.5	2.4	-	-	0.4	0.1	1.9	0.4	291	(Longvah et al., 2017)
			345	20.3	1.4	61	10.4	-	-	-	0.1	-	1.7	-	-	(Rajyalakshmi and Geervani, 1994)
		Field bean (white variety)	280	19.8	0.9	77.2	5.5	2.8	-	-	0.4	0.1	2.0	0.4	289	(Longvah et al., 2017)
			355	20.3	2.4	62	6.1	-	-	-	0.1	-	1.1	-	-	(Rajyalakshmi and Geervani, 1994)
		Field bean (red variety)	283	19.9	0.9	75.2	4.9	2.4	-	-	0.3	0.1	2.0	0.4	292	(Longvah et al., 2017)
			364	20.3	2.9	78	11.8	-	-	-	0.2	-	2.5	-	-	(Rajyalakshmi and Geervani, 1994)
**Black eyed-pea**	*Vigna unguiculata* (L.) Walp.	*Judumulu*	350	22.0	2.1	20	8	-	-	-	0.1	0.1	1.3	-	-	(Rajyalakshmi and Geervani, 1994)
**Rice bean**	*Phaseolus calcaratus* Roxb.	*Sutro/sutri*	332	21.5	0.3	302	-	-	-	-	-	-	-	-	-	(National Institute of Nutrition et al., 1978)
	Bauhinia vahlii Wight & Arn.	*Mahul/Seldey*	-	24.2	28.5	-	-	-	-	-	-	-	-	-	-	(Jain and Tiwari, 2012)
**Prickly chaff flower**	*Achyranthes aspera* L.	*Latjeera/Chirchita*	-	21.4	18.4	-	-	-	-	-	-	-	-	-	-	(Jain and Tiwari, 2012)
**Common oak**	*Quercus robur* L.	*Soh ot*	-	6.9	1.5	410	4.7	1.6	-	-	-	-	-	-	-	(Agrahar-Murugkar and Subbulakshmi, 2005)
**Chinquapin**	*Castanopsis indica* (Roxb. ex Lindl.) A.DC.	*Soh ot rit/ Soh ot*	-	4.9	0.3	1540	2.6	1.5	155	8.1	-	-	-	-	-	(Agrahar-Murugkar and Subbulakshmi, 2005)
			264	3.04	0.7	985	1.2	0.5	-	-	0.4	0.1	1.9	0.2	5.2	(Chyne et al., 2019)
**Nutgall**	*Rhus chinensis* Mill.	*Sohma*	-	7	12.3	1020	4.2	2.4	15	-	-	-	-	-	-	(Agrahar-Murugkar and Subbulakshmi, 2005)
**Perilla**	*Perilla frutescens* (L.) Britton	*Nei lieh*	484	23.9	23.8	336	8.3	5.02	-	-	0.3	0.1	1.7	0.1	178	(Chyne et al., 2019)
		Hanshi	615	17.4	51.7	269	9	4.7	-	-	-	-	-	-	-	(Longvah and Deosthale, 1991)
**Sesame seeds (black)**	*Sesamum indicum* L.	*Nei iong*	475	37.8	28.2	980	16.3	8.3	-	-	0.2	0.1	0.6	0.1	170	(Chyne et al., 2019)
**Chalmogra**	*Gynocardia odorata* R.Br.	*Soh liang*	147	6.3	6.2	792	8.3	18.6	-	0.1	0.4	0.1	0.5	0.1	8.5	(Chyne et al., 2019)

Common name	Botanical name	Vernacular name	Energy (kcal /100g)	Protein (g/100g)	Fat (g/ 100g)	Ca (mg /100g)	Iron (mg/ 100g)	Zinc (mg/ 100g)	Vit A (µg/ 100g)	Vit C (mg /100g)	Vit B1 (mg/ 100g)	Vit B2 (mg/ 100g)	Vit B3 (mg/ 100g)	Vit B6 (mg/ 100g)	Vit B9 (µg/ 100g)	Reference
**Job's tears**	*Coix lacryma* var. *stenocarpa* Oliv.	*Riew magain*	-	13.3	7	1100	2.4	5.1	-	-	-	-	-	-	-	(Agrahar-Murugkar and Subbulakshmi, 2005)
**Jack bean**	*Canavalia ensifor mis* (L.) DC.		375	35	4.3	497.9	5.2	4.3	-	-	-	-	-	-	-	(Vadivel and Janardhanan, 2005)
**Sword bean**	*Canavalia gladiat a* (Jacq.) DC.	*Badi sem*	379	25.5	3.3	510.1	10.9	6.6	-	-	-	-	-	-	-	(Vadivel and Janardhanan, 2005)
	Cassia floribunda Collad.		358	21.7	3.1	537.8	3.6	2	-	-	-	-	-	-	-	(Vadivel and Janardhanan, 2005)
**Pot Casia**	*Senna obtusifolia* (L.) H.S.Irwin & Barneby		389	20.3	7.4	572.2	10.7	30	-	-	-	-	-	-	-	(Vadivel and Janardhanan, 2005)
	Mucuna monospe rma Wight		388	21.2	9.6	313.8	4.8	7.7	-	-	-	-	-	-	-	(Vadivel and Janardhanan, 2005)
GREEN LEAFY VEGETABLES																
**Goose weed**	*Sphenoclea zeylanica* Gaertn	*Jheel-morich*	24	3.1	0.1	6.2	1.8	0.7	-	40.4	-	-	-	-	-	(Basumatary and Narzary, 2017)
**Pepper weed**	*Cardamine hirsuta* L		22	4.0	0.2	6.2	6.1	0.3	-	35.6	-	-	-	-	-	(Basumatary and Narzary, 2017)
Aso-pat/Ouput	*Natsiatum herpeticum* Buch. -Ham. ex Arn.	*Aso-pat/Ouput*	55	5.4	0.7	5.5	2.5	0.7	-	85.7	-	-	-	-	-	(Basumatary and Narzary, 2017)
	Sphaerantus peguensis Kurtz ex C.B. Clark		28	3.2	0.3	5	2.4	0.2	-	15.2	-	-	-	-	-	(Basumatary and Narzary, 2017)
**Bankundri**	*Melothria perpusilla* (Blume) Cogn.		67	2.6	0.2	6.1	1.7	0.2	-	57.1	-	-	-	-	-	(Basumatary and Narzary, 2017)
**Chinese Knotweed**	*Persicaria chinensis* (L) H. Gross	*Wnkham khalai*	26	3.7	0.3	5.1	1.6	0.3	-	27.8	-	-	-	-	-	(Basumatary and Narzary, 2017)
**Malabar spinach**	*Basella rubra* L.	*Poi saag/Pondk a saag/Poi saag*	-	5.2	0.9	-	-	-	-	83.7	-	-	-	-	-	(Bhardwaj et al., 2009)
			-	-	-	203	8.4	-	179	138	-	-	-	-	-	(Singh et al., 2018)
			21	3.1	-	160.5	3.4	0.4	62	3	-	-	-	-	-	(Ghosh-Jerath et al., 2020)

Common name	Botanical name	Vernacular name	Energy (kcal /100g)	Protein (g/100g)	Fat (g/ 100g)	Ca (mg /100g)	Iron (mg/ 100g)	Zinc (mg/ 100g)	Vit A (µg/ 100g)	Vit C (mg /100g)	Vit B1 (mg/ 100g)	Vit B2 (mg/ 100g)	Vit B3 (mg/ 100g)	Vit B6 (mg/ 100g)	Vit B9 (µg/ 100g)	Reference
**Basella leaves**	*Basella alba* L.	*Bon Pui Sak*	-	2.8	0.4	-	-	-	-	87	-	-	-	-	-	(Jana, 2004)
			-	-	-	138	9.4	-	786	297	-	-	-	-	-	(Singh et al., 2018)
			20	1.5	0.4	93.8	4.2	0.4	2473	63.4	0.1	0.2	0.5	0.2	90	(Longvah et al., 2017)
**East Indian Glory Bower**	*Clerodendrum colebrookianum* Walp.	*Oen/ Ongin*	-	4.8	1.7	-	-	-	-	29.1	-	-	-	-	-	(Bhardwaj et al., 2009)
**Crookneck pumpkin leaves**	*Cucurbita moschata* Duchesne	*Tapa*	-	4.0	1.0	-	-	-	-	11.7	-	-	-	-	-	(Bhardwaj et al., 2009)
**Vegetable fern**	*Diplazium esculentum* (Retz.) Sw.	*Dhekia/Churuli/Tyrkhang*	-	7.4	0.8	-	-	-	-	18.6	-	-	-	-	-	(Bhardwaj et al., 2009)
			-	3.6	0.4	112	1.8	0.9	833	32	-	-	-	-	-	(Pradeepkumar et al., 2015)
			-	17.4	5.6	1290	-	-	-	-	-	-	-	-	-	(Tag et al., 2014)
			-	-	-	47.9	1.6	-	1	-	-	-	-	-	-	(Agrahar-Murugkar, 2006)
			-	-	-	-	2.9	3.8	-	147	-	-	-	-	-	(Medak and Singha, 2018)
**Hill Gynura**	*Gynura cusimbua* (D.Don) S.Moore	*Ogen*	-	5.6	2.9	-	-	-	-	34.6	-	-	-	-	-	(Bhardwaj et al., 2009)
	Glochidion multiloculare (Rottler ex Willd.) Voigt	*Gaam oying*	-	9.2	3.6	-	-	-	-	61.3	-	-	-	-	-	(Bhardwaj et al., 2009)
**East Himalayan Mussaenda**	*Mussaenda roxburghii* Hook.f.	*Aksap*	-	3.5	1.6	-	-	-	-	17.8	-	-	-	-	-	(Bhardwaj et al., 2009)
	Pouzolzia bennettiana	*Oyik*	-	7.4	0.9	-	-	-	-	18.2	-	-	-	-	-	(Bhardwaj et al., 2009)
**Weaver's Beam tree**	*Schrebera swietenioides Roxb.*	*Mokha*	111	3.5	0.9	831	5.1	0.7	-	-	-	-	-	-	-	(Bhattacharjee et al., 2009)
**Terena leaves**	NA	*Terena*	34	1.8	0.8	230	0.9	0.3	-	-	-	-	-	-	-	(Bhattacharjee et al., 2009)
**Water celery**	*Oenanthe javanica* (Blume) DC.	*Komprek*	-	22.5	3.9	1440	-	-	-	-	-	-	-	-	-	(Tag et al., 2014)
			-	1.3	1.6	159	2.4	0.9	-	27.4	-	-	-	0.02	126	(Loukrakpam et al., 2019)
**Black Pig Weed leaves**	*Trianthema portulacastrum* L.	*Ohoi-arak/ Ohio ara /Naolo Ghasi/Adachitkana*	53	3.4	-	202	10.7	0.4	16 010	12	-	-	-	-	22	(Ghosh-Jerath et al., 2016)
			-	2.5	-	52	4.2	0.4	4000	22	0.1	-	-	-	-	(Gupta et al., 2005)
**Polpala/Kapurijadi**	*Aerva lanata* (L.) Juss.	*Lapongarak / Lupu ara*	56	4.6	-	322	22.1	0.7	21 760	19	-	-	7.0	-	41	(Ghosh-Jerath et al., 2016)

Common name	Botanical name	Vernacular name	Energy (kcal /100g)	Protein (g/100g)	Fat (g/ 100g)	Ca (mg /100g)	Iron (mg/ 100g)	Zinc (mg/ 100g)	Vit A (µg/ 100g)	Vit C (mg /100g)	Vit B1 (mg/ 100g)	Vit B2 (mg/ 100g)	Vit B3 (mg/ 100g)	Vit B6 (mg/ 100g)	Vit B9 (µg/ 100g)	Reference
**Pot Casia**	*Senna obtusifolia* (L.) H.S. Irwin & Barneby	*Chakod ara/Chakod /Kanyur aa/Thakara /Panwar*	49	5.0	0.8	520	12.4	-	10512	0.1	0.2	0.8	-	-	-	(National Institute of Nutrition et al., 1978)
			-	5.3	0.9	720	6.7	1.4	1822	151.8	-	-	-	-	-	(Pradeepkumar et al., 2015)
			-	20.3	23.0	-	-	-	-	-	-	-	-	-	-	(Jain and Tiwari, 2012)
**Colocasia leaves**	*Colocasia esculenta* (L.) Schott	*Saaru saag/Vayalt haalu/Saaru saag/saru ara/saru-arak/Maked i Ghasi*	56	3.9	1.5	227	10	-	5920	12	0.2	0.3	1.1	-	-	(National Institute of Nutrition et al., 1978)
			-	2.7	0.3	96	0.6	0.2	972	66.1	-	-	-	-	-	(Pradeepkumar et al., 2015)
			44	3.4	1.4	216	3.4	0.8	146	40.7	0.1	0.1	-	-	159	(Longvah et al., 2017)
	Colocasia antiquo rum Schott	*Pechki aa/Karintha alu*	56	3.9	-	227	10	-	10278	12	-	-	-	-	-	(Horo and Topno, 2015)
			-	2.8	0.8	110	3.8	1.3	908	27.8	-	-	-	-	-	(Pradeepkumar et al., 2015)
**Chimti leaves**	*Polygonum abbre viatum* Kom.	*Chimti sag*	46	3.2	0.7	194	-	-	-	-	-	-	-	-	-	(National Institute of Nutrition et al., 1978)
**Mountain ebony/Kachnar leaves**	*Bauhinia variegata* L.	*Konar sag*	62	3.6	1.0	312	-	-	-	-	-	-	-	-	-	(National Institute of Nutrition et al., 1978)
**Koinaar leaves**	*Bauhinia purpurea* L..	*Komo Ghasi/Sing ara/Sing aa*	85	8.7	-	146.5	4.3	1.4	2935	2.6	15.3	0.7	-	-	-	(Ghosh-Jerath et al., 2020)
			62	3.6	-	212	-	-	-	-	-	-	-	-	-	(Horo and Topno, 2015)
**Garkha leaves**	*Celosia argentea* L .	*Siliary saag/Sirgiti-arak Sinduar saag /Chilo ghasi Sirgiti aa/ Siliary ara Sirgiti aa/Annae*	38	2.0	0.7	323	-	-	-	-	-	-	-	-	-	(Ghosh-Jerath et al., 2016)
			25	2.4	-	150.5	7.9	0.6	4797	3	0.3	-	-	-	-	(Ghosh-Jerath et al., 2020)
			48	4.6	0.4	202.6	7.7	0.2	1157	-	-	0.3	-	-	0	(Gupta et al., 2005)
			45	3.8	-	268	-	-	-	-	-	-	-	-	-	(Horo and Topno, 2015)
			-	3.2	-	188	13.2	0.5	4420	26	0.1	-	-	-	-	(Jain and Tiwari, 2012)

Common name	Botanical name	Vernacular name	Energy (kcal /100g)	Protein (g/100g)	Fat (g/ 100g)	Ca (mg /100g)	Iron (mg/ 100g)	Zinc (mg/ 100g)	Vit A (µg/ 100g)	Vit C (mg /100g)	Vit B1 (mg/ 100g)	Vit B2 (mg/ 100g)	Vit B3 (mg/ 100g)	Vit B6 (mg/ 100g)	Vit B9 (µg/ 100g)	Reference
**Slender amaranth**	*Amaranthus viridis* L.	*Bhaji saag/Bon Notey Sak/Marsha bhaji/Jada saw/Chaulai*	38	5.2	0.3	330	18.7	-	-	179	-	-	-	-	-	(National Institute of Nutrition et al., 1978)
			-	5.2	0.3	-	-	-	-	178	-	-	-	-	-	(Jana, 2004)
			-	-	-	416	34	-	483	252	-	-	-	-	-	(Singh et al., 2018)
			-	-	-	2.2	1.2	-	795	-	-	-	-	-	-	(Agrahar-Murugkar, 2006)
			-	6.3	20.3	-	-	-	-	-	-	-	-	-	-	(Jain and Tiwari, 2012)
**Red amaranth**	*Amaranthus gangeticus* L.	*Lal Bhaji*	33	3.9	0.6	245	7.3	1.4	8457	86.2	0.0	0.3	0.6	0.22	82	(Longvah et al., 2017)
	Amaranthus gangeticus Roxb	*Leper aa*	45	4.0	-	397	6.3	-	520	99	-	-	-	-	-	(Horo and Topno, 2015)
**Potato leaves**	*Solanum tuberosum* L.	*Aloo saag/Aloo Ghasi/Aloo arak/Aloo aa*	40	4.4	0.9	120	-	-	-	-	-	-	-	-	-	(National Institute of Nutrition et al., 1978)
			38	6.2	-	127.9	7.2	0.4	19850	3.6	0.5	-	-	-	-	(Ghosh-Jerath et al., 2020)
			40	4.4	-	120	-	-	-	-	-	-	-	-	-	(Ghosh-Jerath et al., 2020)
**Mata leaves**	*Antidesma diandrum* (Roxb.) B.Heyne ex Roth	*Mata sag/Matha-arak (lupu)/Tisso Ghasi/Ching yensil*	303	7.2	4.8	1717	-	-	-	-	-	-	-	-	-	(National Institute of Nutrition et al., 1978)
			-	2.3	1.2	127	7.1	0.8	-	4.2	-	-	-	0.03	419	(Loukrakpam et al., 2019)
			109	4.7	-	474.3	5.3	0.7	2871	1.3	13.9	2.6	-	-	-	(Ghosh-Jerath et al., 2020)
**Katai leaves**	*Meyna pubescens* (Kurz.) Robyns	Sarli saag/sarla ara/sarli aa	86	4.0	1.1	127	-	-	-	-	-	-	-	-	-	(National Institute of Nutrition et al., 1978)
**Kena leaves**	*Commelina benghalensis* L.	*Berbayo Ghasi/Upundu ara /Bat pied/Kena /Kanne*	27	2.5	-	130.8	42.9	0.8	6742	6	-	-	-	-	1	(Ghosh-Jerath et al., 2020)
			42	3.4	-	121.2	21.9	0.7	1800	1.5	1.8	3.2	-	-	-	(Ghosh-Jerath et al., 2021)
			-	-	-	172.9	1.5	-	14	-	-	-	-	-	-	(Agrahar-Murugkar, 2006)
			-	-	-	1432	116	2.7	-	-	-	-	-	-	-	(Mahadkar et al., 2012)
			-	2.4	-	113	7.1	0.6	3810	46	0.0	-	-	-	-	(Gupta et al., 2005)
Aradiyo Ghasi	NA	*Aradiyo Ghasi*	45	5.8	-	231.8	77.6	1.2	943	6	0.6	0.7	-	-	-	(Ghosh-Jerath et al., 2020)
**Sunsuni leaves**			113	7.4	-	90.1	16.5	3.5	15333	1.3	2.3	2.6	-	-	-	(Ghosh-Jerath et al., 2020)

Common name	Botanical name	Vernacular name	Energy (kcal /100g)	Protein (g/100g)	Fat (g/ 100g)	Ca (mg /100g)	Iron (mg/ 100g)	Zinc (mg/ 100g)	Vit A (µg/ 100g)	Vit C (mg /100g)	Vit B1 (mg/ 100g)	Vit B2 (mg/ 100g)	Vit B3 (mg/ 100g)	Vit B6 (mg/ 100g)	Vit B9 (µg/ 100g)	Reference
	Marsilea minuta L.	*Junjuni/ Susni ara/Chatom ara /Sunsuniya saag/susni saag/Chatom aa*	46	3.7	-	53	2.1	-	-	-	-	-	-	-	-	(National Institute of Nutrition et al., 1978)
Mannadro	NA	*Mannadro*	102	10.7	-	73.3	5.2	2.5	316	3	-	0.8	-	-	-	(Ghosh-Jerath et al., 2020)
**Khesari leaves**	*Lathyrus sativus* L.	*Khesari saag/Khesari ara/Kansari aa*	46	6.2	-	69.5	6.6	0.4	5452	1.2	1.9	0.5	-	-	-	(Ghosh-Jerath et al., 2020)
			55	2.1	-	160	7.3	-	3000	41	-	-	-	-	-	(Horo and Topno, 2015)
**Gadhakand leaves**	*Boerhavia diffusa* L.	*Kecho aa/Punarnava /Bagargunchi*	37	1.2	-	650	-	-	21	70	-	-	-	-	-	(Horo and Topno, 2015)
			-	16.7	20.5	-	-	-	-	-	-	-	-	-	-	(Jain and Tiwari, 2012)
			-	3.0	-	330	7.8	0.4	6730	16	0.1	-	-	-	-	(Gupta et al., 2005)
**Khapra leaves**	*Boerhavia procumbens* Banks ex Roxb	*Kecho ara/Khapra saag*	52	4.4	-	877.9	9.4	1.7	2257	0.7	0.4	1.3	-	0.4	-	(Ghosh-Jerath et al., 2021)
**Wild mustard**	*Cleome viscosa* L.	*Hurhuria aa*	73	5.6	-	881	24.4	-	-	-	-	-	-	-	-	(Horo and Topno, 2015)
**Asthma plant**	*Euphorbia hirta* L.	*Towa aa*	83	4.7	-	456	21.1	-	-	44	-	-	-	-	-	(Horo and Topno, 2015)
**Common knotweed**	*Polygonum plebeium* R.Br.	*Mui aa*	46	3.2	-	194	-	-	-	-	-	-	-	-	-	(Horo and Topno, 2015)
**Chukra leaves**	*Rumex vesicarius* L.	*Tissa palak aa*	15	1.6	-	63	0.8	-	3660	12	-	-	-	-	-	(Horo and Topno, 2015)
**Drumstick leaves**	*Moringa oleifera* Lam.	*Sajne Sak/Sahjana /Muri ara/Munga ara/Sanjhodi*	-	6.7	1.7	-	-	-	-	220	-	-	-	-	-	(Jana, 2004)
			-	-	-	18	2.8	-	595	130	-	-	-	-	-	(Singh et al., 2018)
			67	6.4	1.6	314	4.6	0.7	17542	108	0.1	0.5	-	-	43	(Longvah et al., 2017)
			92	6.1	-	440	0.9	-	5520	99	-	-	-	-	-	(Horo and Topno, 2015)
			-	19.5	22.8	-	-	-	-	-	-	-	-	-	-	(Jain and Tiwari, 2012)

Common name	Botanical name	Vernacular name	Energy (kcal /100g)	Protein (g/100g)	Fat (g/ 100g)	Ca (mg /100g)	Iron (mg/ 100g)	Zinc (mg/ 100g)	Vit A (µg/ 100g)	Vit C (mg /100g)	Vit B1 (mg/ 100g)	Vit B2 (mg/ 100g)	Vit B3 (mg/ 100g)	Vit B6 (mg/ 100g)	Vit B9 (µg/ 100g)	Reference
		ghasi/Munga saag/Munga-arak/Mulgaa/Sehjana saag														
**Gogu leaves, red stem**	*Hibiscus sabdariffa* L.	*Ambad bhaji/Khatta Bhaji/Jarsong/Epilara/kudrum*	133	6.9	1.7	86.7	3.3	15.8	-	-	-	-	-	-	-	(Laddha et al., 2015)
			-	-	-	498	187	-	225	283	-	-	-	-	-	(Singh et al., 2018)
			-	-	-	225.5	0.4	-	22	-	-	-	-	-	-	(Agrahar-Murugkar, 2006)
			36	1.9	1.1	145	7.7	0.7	5285	29.7	0.1	0.1	-	-	75	(Longvah et al., 2017)
	Brassaiopsis hainla (Buch.-Ham.) Seem.	*Lainong*	-	13.8	1.8	-	-	-	-	77.7	-	-	-	-	-	(Panmei et al., 2016)
**Melinjo**	*Gnetum gnemon* L .	*Ganmakhen*	-	20.1	2.1	-	-	-	-	66.7	-	-	-	-	-	(Panmei et al., 2016)
**Himalayan clearweed**	*Pilea scripta* (Buch.-Ham. ex D. Don) Wedd.	*Turingnong*	-	15.3	2.9	-	-	-	-	66.7	-	-	-	-	-	(Panmei et al., 2016)
	Rhynchotechum ellipticum (Wall. ex D. Dietr.) A. DC.	*Gankarek*	-	8.9	1.4	-	-	-	-	33.3	-	-	-	-	-	(Panmei et al., 2016)
**Dogal tree leaves**	*Sarcochlamys pulcherrima* Gaudich.	*Goibalei*	-	20.3	1.5	-	-	-	-	111.1	-	-	-	-	-	(Panmei et al., 2016)
**Prickly Chaff Flower**	*Achyranthes aspera* L.	*Vankadalaadi*	-	4.1	0.4	433	22.6	1.4	1573	43.2	-	-	-	-	-	(Pradeepkumar et al., 2015)
**Ponnaganni**	*Alternanthera sessilis* (L.) R.Br. ex DC.	*Ponnaamkanni/*Garundiaa/*Garundi / Gundri*	-	4.7	0.7	199	14.7	0.5	1079	49	-	-	-	-	-	(Pradeepkumar et al., 2015)
			13	2.5	-	510	60	-	192	17	-	-	-	-	-	(Horo and Topno, 2015)
			51	5.3	0.7	388	3.9	1	5288	103	0.0	0.1	-	-	48	(Ghosh-Jerath et al., 2020)

Common name	Botanical name	Vernacular name	Energy (kcal /100g)	Protein (g/100g)	Fat (g/ 100g)	Ca (mg /100g)	Iron (mg/ 100g)	Zinc (mg/ 100g)	Vit A (µg/ 100g)	Vit C (mg /100g)	Vit B1 (mg/ 100g)	Vit B2 (mg/ 100g)	Vit B3 (mg/ 100g)	Vit B6 (mg/ 100g)	Vit B9 (µg/ 100g)	Reference
		ara/Garundi arak/Gundri saag														
**Amaranth spinosus, leaves, green**	*Amaranthus spinosus* L.	*Leper aa/Mullancheera, Mullukeera/ Leped ara/ Gandhari-arak/Gandhari saag/Adro Ghasi/Matla Bhaji*	43	4.0	-	800	22.9	-	3564	33	-	-	-	-	-	(Horo and Topno, 2015)
			-	4.0	0.1	698	15.4	2.4	2135	105.4	-	-	-	-	-	(Pradeepkumar et al., 2015)
			24	1.6	0.5	359	6.4	1.6	1594	77.3	0.0	0.1	-	-	41	(Longvah et al., 2017)
**Common Leucas**	*Leucas aspera (Willd.)* Link.	*Thumba*	-	2.7	0.6	170	3.5	0.7	1341	34.2	-	-	-	-	-	(Pradeepkumar et al., 2015)
Kaattupaaval	*Momordica sahyadrica* Kattuk. and V.T.Antony	*Kaattupaaval*	-	2.6	0.6	1360	5.2	1.7	1684	54.9	-	-	-	-	-	(Pradeepkumar et al., 2015)
**Black night shade**	*Solanum nigrum* L.	*Mudungachappu*	-	4.3	0.8	346	4.8	1.6	2061	141.5	-	-	-	-	-	(Pradeepkumar et al., 2015)
		How-ore	335	21.2	2.0	2633	13.2	15.3	-	-	-	-	-	-	-	(Seal et al., 2016)
Saambaarcheera	*Talinum portulacifolium* (Forssk.) Asch. ex Schweinf.	*Saambaarcheera*	-	1.9	0.3	198	1.4	0.5	733	175.5	-	-	-	-	-	(Pradeepkumar et al., 2015)
**Chinese Spinach**	*Amaranthus tricolor* L		-	4.0	0.5	397	3.5	0.2	5520	99	-	-	-	-	-	(Pradeepkumar et al., 2015)
		Marsha bhaji	-	-	-	437	35	-	510	245	-	-	-	-	-	(Singh et al., 2018)
		Kilkeerae	-	3.4	-	239	15	0.6	5410	39	0.1	-	-	-	-	(Gupta et al., 2005)
**Curry leaves**	*Murraya koenigii* (L.) Spreng.	*Curry patta*	-	-	-	830	15	-	777	250	-	-	-	-	-	(Singh et al., 2018)
			64	7.4	1.1	659	8.7	1.2	7663	6	0.1	0.1	0.9	0.6	117	(Longvah et al., 2017)
**Agathi leaves**		*Agathi*	71	8.0	1.4	901	4.4	0.5	12582	121	0.3	0.3	1.2	0.2	120	(Longvah et al., 2017)

Common name	Botanical name	Vernacular name	Energy (kcal /100g)	Protein (g/100g)	Fat (g/ 100g)	Ca (mg /100g)	Iron (mg/ 100g)	Zinc (mg/ 100g)	Vit A (µg/ 100g)	Vit C (mg /100g)	Vit B1 (mg/ 100g)	Vit B2 (mg/ 100g)	Vit B3 (mg/ 100g)	Vit B6 (mg/ 100g)	Vit B9 (µg/ 100g)	Reference
	Sesbania grandiflora (L.) Pers.		-	-	-	404	5	-	1946	304	-	-	-	-	-	(Singh et al., 2018)
**Alligator weed**	*Alternanthera philoxeroides* (Mart.) Griseb.	*Madrasi Bhaji/Ong put*	-	-	-	354	104	-	1276	227	-	-	-	-	-	(Singh et al., 2018)
			-	-	-	135.6	1	-	12	-	-	-	-	-	-	(Agrahar-Murugkar, 2006)
**Purple amaranth**	*Amaranthus lividus* L.	*Marsha bhaji*	-	-	-	215	124	-	567	308	-	-	-	-	-	(Singh et al., 2018)
**Water hyssop/Brahmi**	*Bacopa monnieri* (L.) Wettst.	*Brahmi/Daliaa*	-	-	-	250	95	-	745	120	-	-	-	-	-	(Singh et al., 2018)
			3	2.9	-	290	-	-	2803	13	-	-	-	-	-	(Horo and Topno, 2015)
**White jute**	*Corchorus capsularis* L.	*Patt*	-	-	-	298	11	-	384	282	-	-	-	-	-	(Singh et al., 2018)
**Male fern**	*Dryopteris filix-mas* (L.) Schott.	*Deki Bhaji*	-	-	-	40	4.2	-	260	431	-	-	-	-	-	(Singh et al., 2018)
**Helencho**	*Enhydra fluctuans* Lour.	*Helencho/Hirmichiya saag*	-	-	-	365	129	-	188	502	-	-	-	-	-	(Singh et al., 2018)
			38	2.1	-	246	16.9	0.9	980	4	1.0	-	-	-	10	(Ghosh-Jerath et al., 2015)
**Wild coriander/ Curantro/**	*Eryngium foetidum* L.	*Burma Dhaniya/Duhania Khlaw/ Dhania Khasi*	-	-	-	354	106	-	499	285	-	-	-	-	-	(Singh et al., 2018)
			44	2.2	0.8	323	6.6	0.6								(Chyne et al., 2019)
			-	-	-	235	4.6	-	44	-	-	-	-	-	-	(Agrahar-Murugkar, 2006)
**Kulekhara**	*Hygrophila auriculata* (Schumach.) Heine	*Kulekhara*	-	-	-	332	112	-	415	103	-	-	-	-	-	(Singh et al., 2018)
**Water primrose**	*Jussiaea repens* L.	*Malencho*	-	-	-	142	25	-	430	220	-	-	-	-	-	(Singh et al., 2018)
**Wild Betel**	*Piper sarmentosum* Roxb.	*Pipali sag*	-	-	-	280	6.5	-	182	263	-	-	-	-	-	(Singh et al., 2018)
**Star Gooseberry**	*Sauropus androgynus* (L.) Merr.	*Chakurmani*	-	-	-	409	21	-	195	314	-	-	-	-	-	(Singh et al., 2018)
	Tragia lassia Radcl.-Sm. & Govaerts		-	1.9	-	-	-	-	-	-	-	-	-	-	-	(Terangpi and Teron, 2015)

Common name	Botanical name	Vernacular name	Energy (kcal /100g)	Protein (g/100g)	Fat (g/ 100g)	Ca (mg /100g)	Iron (mg/ 100g)	Zinc (mg/ 100g)	Vit A (µg/ 100g)	Vit C (mg /100g)	Vit B1 (mg/ 100g)	Vit B2 (mg/ 100g)	Vit B3 (mg/ 100g)	Vit B6 (mg/ 100g)	Vit B9 (µg/ 100g)	Reference
	Premna latifolia Roxb.		-	2.1	-	-	-	-	-	-	-	-	-	-	-	(Terangpi and Teron, 2015)
**Kantha leaves**	*Euphorbia granulate* Forssk.	*Daav ghasi/Kantha-arak*	46	3.5	-	425	81.1	1	11680	9	3.1	-	-	-	7	(Ghosh-Jerath et al., 2016)
**Bottle gourd leaves**	*Lagenaria siceraria* (Molina) Standl.	*Kaddu ara/Kaddu arak/Lol ghasi*	39	2.3	0.7	80	-	-	-	-	-	-	-	-	-	(National Institute of Nutrition et al., 1978)
**Dhurup leaves**	*Leucas lavandulifolia* Sm.	*Khadia ara/Kondi ghasi/Dhurup-arak*	67	5.7	-	236	20	0.8	18460	8	-	-	-	-	11	(Ghosh-Jerath et al., 2016)
**Banyan leaves**	*Ficus benghalensis* L.	*Hesa ara/Hesak-arak/Pakke di Ghasi*	121	2.9	-	295	2.8	0.8	8200	-	-	-	-	-	4	(Ghosh-Jerath et al., 2016)
**Bengal gram leaves**	*Cicer arietinum* L.	*Chana Bhaji/Boot ara/Boot ghasi/Chana saag/But-arak/But aa*	97	7.0	1.4	340	23.8	-	-	-	-	-	-	-	-	(National Institute of Nutrition et al., 1978)
			97	7.0	-	340	23.8	-	978	61	-	-	-	-	-	(Horo and Topno, 2015)
**Garlic leaves**	*Allium sativum* L.	*Lahsun saag/Nasni Ghasi*	34	3.1	-	221	6	0.2	5100	6	-	-	-	-	3	(Ghosh-Jerath et al., 2015)
**Field mustard**	*Brassica campestris* L.	*Chiniya saag*	31	1.5	-	274	5.9	-	4290	11	-	0.3	-	-	1	(Ghosh-Jerath et al., 2015)
**Mustad leaves**	*Brassica juncea* (L.) Czern.	*Lotni saag*	28	2.2	-	389	19.7	0.8	1750	4	0.8	-	10.5	-	3	(Ghosh-Jerath et al., 2015)
**Tamarind leaves**	*Tamarindus indica L.*	*Jojo-ara*	71	5.8	0.5	66.9	2.8	0.9	168	28.2	0.1	0.0	-	-	92	(Longvah et al., 2017)
**Water spinach**	*Ipomoea aquatica Forssk.* €	*Kalmi ara*	28	2.9	0.2	110	3.9	-	1980	10	0.1	0.1	-	-	-	(National Institute of Nutrition et al., 1978)

Common name	Botanical name	Vernacular name	Energy (kcal /100g)	Protein (g/100g)	Fat (g/ 100g)	Ca (mg /100g)	Iron (mg/ 100g)	Zinc (mg/ 100g)	Vit A (µg/ 100g)	Vit C (mg /100g)	Vit B1 (mg/ 100g)	Vit B2 (mg/ 100g)	Vit B3 (mg/ 100g)	Vit B6 (mg/ 100g)	Vit B9 (µg/ 100g)	Reference
		Nalli Bhaji	-	-	-	286	61	-	510	245	-	-	-	-	-	(Singh et al., 2018)
**Beng leaves**	*Centella asiatica (L.) Urb.*	*Beng saag/ Chokke ara*	54	1.9	-	231	55.7	1.9	500	5	Nil	Nil	-	-	-	(Ghosh-Jerath et al., 2015)
		Muthil	-	3.2	0.3	217	13.6	2	613	56.3	-	-	-	-	-	(Pradeepkumar et al., 2015)
		Medak bhaji	-	-	-	583	179	-	283	262	-	-	-	-	-	(Singh et al., 2018)
		Khlien syiar	-	-	-	204.6	4.7	-	38	-	-	-	-	-	-	(Agrahar-Murugkar, 2006)
		Brahmi	-	2.4	-	174	14.9	0.9	3900	11	0.0	-	-	-	-	(Gupta et al., 2005)
**Amaranth,tender, red leaves**	*Amaranthus retroflexus L.*	*Lal Bhaji/Lal saag*	33	3.9	0.6	245	7.2	1.3	21449	86.2	0.0	0.3	-	-	82	(Longvah et al., 2017)
**Phutkal leaves (dried)**	*Ficus virens Aiton*	*Phutkal ara/Phutkal saag*	324	2.4	1.8	672	4.1	0.3	30	4	-	-	-	-	-	(Ghosh-Jerath et al., 2015)
**Pumpkin leaves**	*Cucurbita maxima* L.	*Kakaru ara*	44	4.2	0.7	271	5.6	0.9	1455	12.3	0.1	0.1	-	-	34	(Longvah et al., 2017)
		-	-	4.1	-	302	4.4	0.6	2270	37	0.2	-	-	-	-	(Gupta et al., 2005)
**Nunia leaves**	*Portulaca quadrifida* L.	*Dali/Dail ara*	19	3.6	-	10.8	31.2	0.8	1015	1.6	0.2	0.7	-	-	-	(Ghosh-Jerath et al., 2021)
**Dheniani**	*Olax scandens* Roxb.	*Soredhe/ Bir/Rimil arak*	98	4.6	-	115.9	7.7	2	1186	3.1	0.3	3.6	-	-	0	(Ghosh-Jerath et al., 2021)
**Khatta saag**	*Cissus auriculata Roxb.*	*Budilaie arak*	26	2.7	-	257.7	1.9	0.8	92	1.7	6.2	0.2	-	-	0	(Ghosh-Jerath et al., 2021)
**Kauwa leaves**	*Rungia quinqueangularis* Koen.	*Kauwa saag*	42	4.7	-	340.4	8.2	18.7	287	-	-	0.7	-	-	0	(Ghosh-Jerath et al., 2021)
**Purslane**	*Portulaca oleracea* L.	*Uri le ara/Kozhup pacheera/Nuna Bhaji*	58	3.4	-	200.2	24.4	1.4	249	-	0.7	0.5	-	-	0	(Ghosh-Jerath et al., 2021)
			-	1.9	0.2	75	11.9	0.8	341	36.8	-	-	-	-	-	(Pradeepkumar et al., 2015)
			-	-	-	115	71	-	290	287	-	-	-	-	-	(Singh et al., 2018)
**Netho leaves**	*Medicago lupulina* Linn.	*Piring ara*	67	1.4	-	293.1	7.8	0.9	3124	-	1.0	0.7	-	-	-	(Ghosh-Jerath et al., 2021)
**Arrow head**	*Sagittaria latifolia* L.	*Tir ara/ Lochkor ara*	51	3.0	-	163.9	5.1	0.3	2561	-	0.3	5.6	-	-	1	(Ghosh-Jerath et al., 2021)

Common name	Botanical name	Vernacular name	Energy (kcal /100g)	Protein (g/100g)	Fat (g/ 100g)	Ca (mg /100g)	Iron (mg/ 100g)	Zinc (mg/ 100g)	Vit A (µg/ 100g)	Vit C (mg /100g)	Vit B1 (mg/ 100g)	Vit B2 (mg/ 100g)	Vit B3 (mg/ 100g)	Vit B6 (mg/ 100g)	Vit B9 (µg/ 100g)	Reference
**Sweet potato leaves**	*Ipomoea batatas (L.) Lam.*	*Sanga ara/Kanda saag*	51	2.6	-	3.5	0.3	0.1	1717	-	1.0	0.1	-	-	-	(Ghosh-Jerath et al., 2021)
**Patsan**	*Hibiscus cannabinus* L.	*Kotle ara*	42	1.5	-	177.9	2.1	0.1	2665	-	0.4	0.2	-	-	-	(Ghosh-Jerath et al., 2021)
**Ash gourd leaves**	*Benincasa hispida* (Thunb.) Cogn.	*Kohna/ Ketha ara*	34	3.5	-	12.9	0.3	0.1	637	-	0.2	0.8	-	-	-	(Ghosh-Jerath et al., 2021)
**Kharika leaves**	*Spergula pentandra* L.	*Chaari ara*	39	0.6	-	90.9	58.7	53.9	83	-	3.9	0.4	-	-	5	(Ghosh-Jerath et al., 2021)
**Akra**	*Vicia sativa* L.	*Chiringid arak*	64	0.7	-	52.5	6.7	0.7	2694	-	1.8	6.8	-	-	8	(Ghosh-Jerath et al., 2021)
**Amrit sak**	*Oxalis corniculata* L.	*Bir chhatom ara/Kynbat Dkhiew/Khatti buti*	54	0.7	-	192.4	2.8	2.3	2600	-	1.6	0.9	-	-	6	(Ghosh-Jerath et al., 2021)
			-	-	-	161.4	16.7	-	50	-	-	-	-	-	-	(Agrahar-Murugkar, 2006)
			-	22.3	23.7	-	-	-	-	-	-	-	-	-	-	(Jain and Tiwari, 2012)
**Himalayan mayflower**	*Maianthemum purpureum* (Wall.) LaFrankie		-	27.2	5.5	1090	-	-	-	-	-	-	-	-	-	(Tag et al., 2014)
**Chives**	*Allium schoenoprasum* L.	*Jaut*	-	-	-	99	0.2	-	7	-	-	-	-	-	-	(Agrahar-Murugkar, 2006)
**Milk Weed**	*Sonchus oleraceus* (L.) L.	*Jalyngiar*	-	-	-	291.5	1.7	-	4052	-	-	-	-	-	-	(Agrahar-Murugkar, 2006)
**Broadleaf Plantain**	*Plantago major*		-	-	-	427.8	8	-	46	-	-	-	-	-	-	(Agrahar-Murugkar, 2006)
**Fish mint**	*Houttuynia cordata* Thunb.	*Jamyrdoh*	-	-	-	89.7	3.3	-	29	-	-	-	-	-	-	(Agrahar-Murugkar, 2006)
			50	3.2	0.6	206	1.9	1	-	9.6	0.02	0.1	1.3	0.6	154	(Chyne et al., 2019)
			-	10.4	3.1	-	-	-	-	-	-	-	-	-	-	(Saha et al., 2014)
Jarain	*Fagopyrum acutatum* (Lehm.) Mansf. ex K.Hammer	*Jarain*	-	-	-	217	1.5	-	2708	-	-	-	-	-	-	(Agrahar-Murugkar, 2006)
**Perennial sow thistle**	*Sonchus arvensis* L.	*Jakhain/Jalynniar*	134	19.5	2.5	2295	65.1	20.1	-	-	-	-	-	-	-	(Seal, 2011)
			-	-	-	170	3.2	-	14	-	-	-	-	-	-	(Agrahar-Murugkar, 2006)
			66	2.4	0.8	285	1.8	1.2	-	15.2	0.1	0.1	1.3	0.1	17.6	(Chyne et al., 2019)

Common name	Botanical name	Vernacular name	Energy (kcal /100g)	Protein (g/100g)	Fat (g/ 100g)	Ca (mg /100g)	Iron (mg/ 100g)	Zinc (mg/ 100g)	Vit A (µg/ 100g)	Vit C (mg /100g)	Vit B1 (mg/ 100g)	Vit B2 (mg/ 100g)	Vit B3 (mg/ 100g)	Vit B6 (mg/ 100g)	Vit B9 (µg/ 100g)	Reference
**Cat's ear**	*Hypochaeris radicata*	*Jakhain*	45	2.5	0.4	177	11.2	0.6	-	18	0.02	0.02	0.4	0.1	15.4	(Chyne et al., 2019)
Jatira	*Corydalis sibirica* (L.f.) Pers.	*Jatira*	-	-	-	99.4	1	-	0	-	-	-	-	-	-	(Agrahar-Murugkar, 2006)
**Wild leek**	*Allium ampelopra sum* L.	*Jyllan*	-	-	-	98.4	-	-	5	-	-	-	-	-	-	(Agrahar-Murugkar, 2006)
Jangew	NA	*Jangew*	-	-	-	200.9	5.7	-	1979	-	-	-	-	-	-	(Agrahar-Murugkar, 2006)
Mahong	*Spilanthes acmell a* (L.) L.	*Mahong*	-	-	-	213.4	1.2	-	6106	-	-	-	-	-	-	(Agrahar-Murugkar, 2006)
Bat saw	*Polygonum alatu m* Buch.-Ham. ex D. Don	*Bat saw*	-	-	-	121.9	1.1	-	66	-	-	-	-	-	-	(Agrahar-Murugkar, 2006)
**Hirankhuri leaves**	*Emilia sonchifolia* (L.) DC. ex DC.	*Jalang shor*	-	-	-	137	3	-	191	-	-	-	-	-	-	(Agrahar-Murugkar, 2006)
	Vernonia altissimifolia	*Jalong*	-	-	-	200.6	1.8	-	23	-	-	-	-	-	-	(Agrahar-Murugkar, 2006)
**East Himalayan Begonia**	*Begonia roxburghi i* A.DC	*Dieng jajew*	-	-	-	336.5	2.2	-	42	-	-	-	-	-	-	(Agrahar-Murugkar, 2006)
Thylleij masi	*Ficus cunea*	*Thylleij masi*	-	-	-	665.8	1.1	-	2538	-	-	-	-	-	-	(Agrahar-Murugkar, 2006)
Jajew maw	*Begonia rubroveni a* Hook.	*Jajew maw*	-	-	-	269	0.6	-	-	-	-	-	-	-	-	(Agrahar-Murugkar, 2006)
Trysim khlieng	*Commelina diffus a* Burm.f	*Trysim khlieng*	-	-	-	86.9	11.6	-	41	-	-	-	-	-	-	(Agrahar-Murugkar, 2006)
**Elephant apple**	*Dillenia indica* L.	*Jamahek*	-	-	-	216.6	0.7	-	-	-	-	-	-	-	-	(Agrahar-Murugkar, 2006)
**Watercress**	*Nasturtium officin ale* R.Br	*Jhur kteih*	-	-	-	92.2	0.6	-	-	-	-	-	-	-	-	(Agrahar-Murugkar, 2006)
**Lobed Leaf Knotweed**	*Polygonum runcin atum* Buch.-Ham. ex D. Don	*Rungri*	163	24.7	1.4	631	-	-	-	-	-	-	-	-	-	(Medak and Singha, 2016)
			-	-	-	-	3	2.5	-	269	-	-	-	-	-	(Medak and Singha, 2018)
Gungi	*Pilea bracteosa* W edd.	*Gungi*	134	16.9	1.5	1091	-	-	-	-	-	-	-	-	-	(Medak and Singha, 2016)
			-	-	-	-	3.7	4.9	-	153	-	-	-	-	-	(Medak and Singha, 2018)
Huj	*Elatostema platyp hyllum* Wedd.	*Huj*	133	18.5	1.3	1412	-	-	-	-	-	-	-	-	-	(Medak and Singha, 2016)
			-	-	-	-	4	3.9	-	75	-	-	-	-	-	(Medak and Singha, 2018)

Common name	Botanical name	Vernacular name	Energy (kcal /100g)	Protein (g/100g)	Fat (g/ 100g)	Ca (mg /100g)	Iron (mg/ 100g)	Zinc (mg/ 100g)	Vit A (µg/ 100g)	Vit C (mg /100g)	Vit B1 (mg/ 100g)	Vit B2 (mg/ 100g)	Vit B3 (mg/ 100g)	Vit B6 (mg/ 100g)	Vit B9 (µg/ 100g)	Reference
**Edible Gynura**	*Gynura bicolor* (Roxb. ex Willd.) DC.		-	-	-	-	3.9	3.6	-	121	-	-	-	-	-	(Medak and Singha, 2018)
	Plantago asiatica subsp. *erosa* (Wall.) Z.Yu Li		-	-	-	-	3.8	5.1	-	187	-	-	-	-	-	(Medak and Singha, 2018)
**Pokeweed**	*Phytolacca acinosa* Roxb.	*Papok*	326	21.9	1.3	2673	52	15.1	-	-	-	-	-	-	-	(Seal et al., 2016)
Raro	*Piper pedicellatum* C. DC.	*Raro*	343	21.2	0.6	2493	41	55	-	-	-	-	-	-	-	(Seal et al., 2016)
Huaig	*Pouzolzia hirta* Blume ex Hassk.	*'Huaig*	331	19.2	1.3	2573	69	13.1	-	-	-	-	-	-	-	(Seal et al., 2016)
Chopchini	*Smilax zeylanica* L	*Chopchini*	-	-	-	828.8	29.9	2	-	-	-	-	-	-	-	(Mahadkar et al., 2012)
**Kokam**	*Garcinia indica* (Thouars) Choisy	*Kokam*	-	-	-	2122.8	20.6	3	-	-	-	-	-	-	-	(Mahadkar et al., 2012)
Balae	*Polygala erioptera* DC.	*Balae*	-	2.2	-	41	4.8	0.7	3830	85	-	-	-	-	-	(Gupta et al., 2005)
**Mexican mint**	*Plectranthus amboinicus* (Lour.) Spreng.	*Doddipatre*	-	0.6	-	158	2.6	0.3	1500	3	0.1	-	-	-	-	(Gupta et al., 2005)
**Lesua**	*Digera muricata* (L.) Mart.	*Gurchi*	-	4.3	-	506	17.7	0.6	3360	49	0.1	-	-	-	-	(Gupta et al., 2005)
Javanada	*Cocculus hirsutus* (L.) W.Theob.	*Javanada*	-	3.9	-	126	9.9	0.6	9200`	28	0.2	-	-	-	-	(Gupta et al., 2005)
**Gandhuli**	*Cleome gynandra* L.	*Naribalae*	-	3.6	-	113	4.8	0.5	5380	42	0.2	-	-	-	-	(Gupta et al., 2005)
**Yellow Gulmohar**	*Delonix elata* (L.) Gamble	*Vayunarayani*	-	7.1	-	112	6.2	0.6	10510	295	0.3	-	-	-	-	(Gupta et al., 2005)
Jarem/ Phuinam	*Clerodendrum glandulosum* Lindl.	*Jarem/ Phuinam*	159	27.6	1.7	190.5	155.5	18.4	-	-	-	-	-	-	-	(Seal, 2011)
Jatira	*Oenanthe linearis* Wall. ex DC.	*Jatira*	127	21.8	1.6	2370	289.3	42.2	-	-	-	-	-	-	-	(Seal, 2011)
Jaiur	*Zanthoxylum acanthopodium* DC.	*Jaiur*	154	28.1	1.9	1435	117.5	86.7	-	-	-	-	-	-	-	(Seal, 2011)

Common name	Botanical name	Vernacular name	Energy (kcal /100g)	Protein (g/100g)	Fat (g/ 100g)	Ca (mg /100g)	Iron (mg/ 100g)	Zinc (mg/ 100g)	Vit A (µg/ 100g)	Vit C (mg /100g)	Vit B1 (mg/ 100g)	Vit B2 (mg/ 100g)	Vit B3 (mg/ 100g)	Vit B6 (mg/ 100g)	Vit B9 (µg/ 100g)	Reference
**Greenbriers**	*Smilax elegans* Wall. ex Kunth	*Sla sohkrot*	28.1	2.4	0.4	1394	10.4	0.5	-	7.2	0.03	0.2	0.5	0.03	158	(Chyne et al., 2019)
**Giant taro leaves**	Alocasia macrorrhizos (L.) G.Don	*Sla wang bam im*	38.7	3.9	0.5	206	14.6	0.5	-	3.4	0.01	0.01	1.3	0.1	19.6	(Chyne et al., 2019)
Jathang	Neilla thyrsiflora	*Jathang*	30.5	1.7	1.4	133	8.04	8.04	-	3.6	0.1	0.1	0.4	0.1	8.2	(Chyne et al., 2019)
**Hooker chives**	*Allium hookeri* Thwaites	*Napakpi*	-	0.7	0.3	-	48.5	23.5	-	-	-	-	-	-	-	(Payum et al., 2015)
			-	1.7	0.6	41.7	2.1	0.5	-	0.1	-	-	-	0.2	177	(Loukrakpam et al., 2019)
**Water mimosa**	*Neptunia oleracea* Lour.	*Ekaithabi*	-	3.5	0.9	142	3.5	0.9	-	0.3	-	-	-	0.3	119	(Loukrakpam et al., 2019)
**Malabar nut leaves**	*Justicia adhatoda* L.	*Nongmakha mapal*	-	5.3	1.6	584	9.5	1.1	-	17.3	-	-	-	0.1	119	(Loukrakpam et al., 2019)
Yelang	*Persicaria barbata* (L.) H.Hara	*Yelang*	-	5.4	0.6	130	7.2	0.9	-	22.4	-	-	-	0.1	287	(Loukrakpam et al., 2019)
**Chickweed**	*Stellaria media* (L.) Vill.	*Yerum kairum*	-	2.5	0.5	139	7.3	0.7	-	0.8	-	-	-	0.02	441	(Loukrakpam et al., 2019)
Fakpai	*Polygonum posumbu* Buch.-Ham. ex D. Don	*Fakpai*	-	1.7	1.5	277	11.4	1.5	-	9.5	-	-	-	0.2	323	(Loukrakpam et al., 2019)
**Garlic chives**	*Allium tuberosum* Rottler ex Spreng.	*Nakuppi*	-	2.9	0.5	70.7	1.3	0.3	-	1.1	-	-	-	0.1	96	(Loukrakpam et al., 2019)
Phunil	*Anaphalis subdecurrens* (DC.) Gamble	*Phunil*	-	4.1	0.5	187	15.9	1.1	-	0.1	-	-	-	0.1	175	(Loukrakpam et al., 2019)
Kengoi	*Persicaria posumba*	*Kengoi*	-	2.8	0.8	54.2	6.8	0.4	-	32.6	-	-	-	0.1	205	(Loukrakpam et al., 2019)
							OTHER VEGETABLES									
**Mahua seeds**	*Bassia latifolia Roxb.*	*Doli mahuda seeds*	559	9.0	64.0	7.3	1.3	-	-	-	-	-	-	-	-	(Bhattacharjee et al., 2009)
**Cowpea**	*Vigna catjang* (L.) Walp.	*Bada ghangra/Barbatti/Bodi*	49	3.7	-	41.0	1	0.6	36	9	-	-	-	-	7	(Ghosh-Jerath et al., 2016)
**Wild Brinjal**		*Koppi*	-	4.8	1.0	-	-	-	-	17.6	-	-	-	-	-	(Bhardwaj et al., 2009)

Common name	Botanical name	Vernacular name	Energy (kcal /100g)	Protein (g/100g)	Fat (g/ 100g)	Ca (mg /100g)	Iron (mg/ 100g)	Zinc (mg/ 100g)	Vit A (µg/ 100g)	Vit C (mg /100g)	Vit B1 (mg/ 100g)	Vit B2 (mg/ 100g)	Vit B3 (mg/ 100g)	Vit B6 (mg/ 100g)	Vit B9 (µg/ 100g)	Reference
	Solanum torvum Sw.	*Singkhangkha*	-	3.9	1.4	36.2	1.1	0.7	-	5.9	-	-	-	0.2	327	(Loukrakpam et al., 2019)
	Solanum khasianum C.B. Clarke	*Koppir*	-	6.3	2.5	-	-	-	-	20	-	-	-	-	-	(Bhardwaj et al., 2009)
**Pindra/Pinra**	*Flacourtia indica* (Burm.f.) Merr.	*Pindra/Pinra*	73	4.5	-	22.5	2.1	0.3	10.7	6.2	-	-	-	-	-	(Ghosh-Jerath et al., 2020)
**Ber alli**	*Dioscorea* spp.	*Ber alli*	112	2.9	-	32.7	2	0.3	-	1.8	-	-	-	-	-	(Ghosh-Jerath et al., 2020)
**Kachnar flower**	*Bauhinias variegata* L.	*Kachna Phool/Burju Baha*	83	2.9	-	404.9	3.4	0.6	416	2.5	-	0.3	-	-	-	(Ghosh-Jerath et al., 2020)
**Sanai Flower**	*Crotalaria juncea* L.	*Sonpu Phool/Jiri Ba*	120	2.9	-	320.2	7.6	0.2	1113	1.8	3.1	-	-	-	-	(Ghosh-Jerath et al., 2020)
**Drumstick flower**	*Moringa oleifera* Lam	*Sanjhori Phool/Sehjana Phool*	71	4.6	-	80.5	5.2	0.7	261	2.4	0.5	0.6	-	-	-	(Ghosh-Jerath et al., 2020)
			-	18.9	21.5	-	-	-	-	-	-	-	-	-	-	(Jain and Tiwari, 2012)
**Amaltas Flower**	*Cassia fistula* L.	*Amaltas Phool*	-	13.1	23.7	-	-	-	-	-	-	-	-	-	-	(Jain and Tiwari, 2012)
**Kattian/Kasai**	*Bridelia retusa* (L.) A.Juss.	*Bon Chalta*	-	1.0	0.2	-	-	-	-	277	-	-	-	-	-	(Jana, 2004)
**Marine seaweed**	*Prasiola crispa* f.		-	37.9	14.5	-	-	-	-	-	-	-	-	-	-	(Saha et al., 2014)
	Wallichia disticha T.Anderson		-	1.3	0.5	-	-	-	-	-	-	-	-	-	-	(Saha et al., 2014)
**Bitter gourd**	*Momordica charantia* L..	*Bir Karela/Jungli Karela*	19	1.3	0.2	16.3	1.1	0.4	126	50.9	0.1	0.0	-	-	51	(Longvah et al., 2017)
		Bon Kankrol	-	1.5	0.5	-	-	-	-	278	-	-	-	-	-	(Jana, 2004)
**Hyacinth bean**	*Lablab purpureus* (L.)	*Sem/Simbi/ Manal*	48	3.8	0.1	210	0.8	0.4	187	9	0.1	0.1	-	-	-	(Longvah et al., 2017)
**Hyacint beans (red)**		*Ri Saw*	52	5.2	0.1	30.7	0.8	0.4	-	0.5	0.1	0.1	0.6	0.04	27	(Chyne et al., 2019)
**Hyacint beans (curve, green)**		*RiKdor*	57	5.02		43.8	0.8	0.2	-	0.5	0.1	0.1	0.5	0.02	28	(Chyne et al., 2019)

Common name	Botanical name	Vernacular name	Energy (kcal /100g)	Protein (g/100g)	Fat (g/ 100g)	Ca (mg /100g)	Iron (mg/ 100g)	Zinc (mg/ 100g)	Vit A (µg/ 100g)	Vit C (mg /100g)	Vit B1 (mg/ 100g)	Vit B2 (mg/ 100g)	Vit B3 (mg/ 100g)	Vit B6 (mg/ 100g)	Vit B9 (µg/ 100g)	Reference
**Hyacint beans (green)**		*RiJyrngam*	61	5.4	0.9	30.6	0.8	0.3	-	0.5	0.1	0.1	0.5	0.1	30	(Chyne et al., 2019)
**Bitter tomato**	*Solanum aethiopicum* L.	*Sohngang heh/ Soh-ngang*	-	11	3.6	590	3.7	1.5	-	-	-	-	-	-	-	(Agrahar-Murugkar and Subbulakshmi, 2005)
			43	4.2	0.5	40.1	1.02	0.3	-	1.2	0.1	0.01	0.6	0.04	106	(Chyne et al., 2019)
**Wild banana stem**	*Musa acuminata* Colla	*Nudkait*	53	2.7	2.6	25.2	1.2	0.5	-	12.6	0.2	0.1	0.5	0.04	123	(Chyne et al., 2019)
**Tree tomato**	*Cyphomandra betacea*	*Soh baingon dieng*	61	1.8	1	20.4	0.8	0.4	-	21	0.1	0.1	0.7	0.1	156	(Chyne et al., 2019)
**Hairy-fruited eggplant**	*Solanum lasiocarpum* Dunal	*Soh ngang rit*	54	4	2.8	23.7	0.9	0.2	-	2.9	0.2	0.1	0.7	0.1	131	(Chyne et al., 2019)
	Solanum indicum L.	*Sohngang rit*	-	14.1	18.8	1300	4.5	3.5	38	826.4	-	-	-	-	-	(Agrahar-Murugkar and Subbulakshmi, 2005)
**Thai eggplant**	*Solanum virginianum* L	*Sohthang*	-	11.3	1.5	990	3.2	1.8	-	321.5	-	-	-	-	-	(Agrahar-Murugkar and Subbulakshmi, 2005)
**Ridge gourd**	*Luffa acutangula* (L.) Roxb.	*Jhinga*	13	0.9	0.1	13.7	0.4	0.2	348	5.4	0.0	0.0	-	-	29	(Longvah et al., 2017)
**Ridge gourd, smooth skin**	*Luffa acutangula* (L.) Roxb.	*Dodo/ Doro*	15	1	0.1	14.9	0.5	0.2	349	8.1	0.0	0.0	-	-	27	(Longvah et al., 2017)
**Bamboo, Tender**	*Bambusa vulgaris* Schrad. ex J.C. Wendl.	*Maas adro/Karu/Bans*	16	1.3	0.4	10	0.3	0.4	-	15.7	0.1	0.1	-	-	17	(Longvah et al., 2017)
**Ash gourd**	*Benincasa hispida* (Thunb.) Cogn.	*Ketha/Zarkunda*	17	0.8	0.1	19.3	0.5	0.1	-	11.4	0.0	0.0	0.12	0.18	14	(Longvah et al., 2017)
**Spine gourd**	.*Momordica diocia* Roxb ex Willd	*Kokri*	52	3.1	1.0	-	4.6	-	-	-	-	-	-	-	-	(National Institute of Nutrition et al., 1978)
			387	3.5	5.1	234	34.1	1.2	-	-	-	-	-	-	-	(Mohan and Kalidass, 2010)
**Kovai**	*Coccinia grandis* (L.) Voigt	*Kundri/Kundur/Bon Kudri/Kundru*	19	1.4	0.2	37.1	0.3	0.1	147	21.1	0.0	0.0	-	-	50	(Longvah et al., 2017)
			-	1.7	0.2	-	-	-	-	18	-	-	-	-	-	(Jana, 2004)
			-	-	-	125	12	-	115	86	-	-	-	-	-	(Singh et al., 2018)
**Turkey Berry**	*Solanum torvum* Swartz.	*Kutumba/ Hanjen*	54	3.5	-	29.8	0.6	0.2	24	1.5	1.3	2.0	-	-	1	(Ghosh-Jerath et al., 2021)

Common name	Botanical name	Vernacular name	Energy (kcal /100g)	Protein (g/100g)	Fat (g/ 100g)	Ca (mg /100g)	Iron (mg/ 100g)	Zinc (mg/ 100g)	Vit A (µg/ 100g)	Vit C (mg /100g)	Vit B1 (mg/ 100g)	Vit B2 (mg/ 100g)	Vit B3 (mg/ 100g)	Vit B6 (mg/ 100g)	Vit B9 (µg/ 100g)	Reference
	Caralluma adscendens var. *attenuata* (Wight) Grav. & Mayur.		422	7.0	10.1	120.3	52	6.8	-	-	-	-	-	-	-	(Mohan and Kalidass, 2010)
	Caralluma pauciflora (Wight) N.E.Br.		427	11.4	11.2	110.1	48	5.5	-	-	-	-	-	-	-	(Mohan and Kalidass, 2010)
**Indian shot**	Canna indica L.		385	6.3	4.3	154.1	11.5	0.9	-	-	-	-	-	-	-	(Mohan and Kalidass, 2010)
**Jackfruit**	*Artocarpus heterophyllus* Lam.	*Katahal*	-	-	-	43	4.2	-	418	73	-	-	-	-	-	(Singh et al., 2018)
			26	2.0	0.4	45.7	0.3	0.2	-	17.5	0.1	0.1	0.2	0.0	36	(Longvah et al., 2017)
**Breadfruit**	*Artocarpus altilis* (Parkinson ex F.A.Zorn) Fosberg	*Bilayati katahal*	-	-	-	40	3.8	-	682	87	-	-	-	-	-	(Singh et al., 2018)
**Spiny bitter gourd**	*Momordica cochinchinensis* (Lour.) Spreng	*Jangli kakrol*	-	-	-	110	12.8	-	463	210	-	-	-	-	-	(Singh et al., 2018)
	Momordica subangulata subsp. renigera (Wall. ex G.Don) W.J.de Wilde	*Kakrol*	-	-	-	198	15.7	-	289	88	-	-	-	-	-	(Singh et al., 2018)
**Ghaf**	*Prosopis cineraria* (L.) Druce	*Shami, Kshenkar*	-	7.3	10.0	-	-	-	-	-	-	-	-	-	-	(Jain and Tiwari, 2012)
**Wild plantain, flower**	*Ensete Superbum* (Roxb.) Cheesuran	*Ran-keli, Chaveli-keli*	-	-	-	665.6	518	3.8	-	-	-	-	-	-	-	(Mahadkar et al., 2012)
**Dhawal**	*Woodfordia fruticosa* (L.) Kurz	*Dhayati*	-	-	-	219.4	55.1	1.6	-	-	-	-	-	-	-	(Mahadkar et al., 2012)
	Dendrocalamus hamiltonii Nees & Arn. ex Munro	*Soidon*	-	1.6	0.2	5.7	0.4	0.5	-	2.8	-	-	-	0.01	128	(Loukrakpam et al., 2019)
**Wild bean**	*Canavalia cathartica* Thouars	*Tebi*	-	2.9	0.3	98.2	0.9	0.4	-	8.5	-	-	-	0.02	193	(Loukrakpam et al., 2019)
**Fox nut**	*Euryale ferox* Salisb.	*Thangjing*	-	5.03	1.6	26.4	0.2	1.2	-	0.6	-	-	-	0.1	36	(Loukrakpam et al., 2019)

Common name	Botanical name	Vernacular name	Energy (kcal /100g)	Protein (g/100g)	Fat (g/ 100g)	Ca (mg /100g)	Iron (mg/ 100g)	Zinc (mg/ 100g)	Vit A (µg/ 100g)	Vit C (mg /100g)	Vit B1 (mg/ 100g)	Vit B2 (mg/ 100g)	Vit B3 (mg/ 100g)	Vit B6 (mg/ 100g)	Vit B9 (µg/ 100g)	Reference
**Broad bean**	*Vicia faba* L.	*Hawaimub*	-	7.1	0.7	68.8	2	1.2	-	4.4	-	-	-	0.1	413	(Loukrakpam et al., 2019)
**Tree bean**	*Parkia timoriana* (DC.) Merr.	*Yongchak*	-	7.4	0.5	196	1.4	1.2	-	13.4	-	-	-	0.03	55	(Loukrakpam et al., 2019)
Feija	*Wendlandia glabr ata* DC.	*Feija*	-	6.5	2.5	413	1.01	2.02	-	2.1	-	-	-	0.02	144	(Loukrakpam et al., 2019)
MUSHROOMS																
**Mushroom, dry**	*Agaricus bisporus*	*Kukkagodug u*	272	20.6	4.3	94	79.4	0.6	-	-	-	-	-	-	-	(Bhattacharjee et al., 2009)
	Amanita sp. 1		-	18.4	1.8	12	26.8	1.5	-	-	-	-	-	-	-	(Das et al., 2015)
	Amanita sp. 2		-	19.4	1.9	17.3	22.8	1.4	-	-	-	-	-	-	-	(Das et al., 2015)
	Amanita sp. 3		-	26.4	12.0	-	-	-	-	-	-	-	-	-	-	(Saha et al., 2014)
	Astraeus sp.		-	18.7	2.1	27.6	15.9	1.3	-	-	-	-	-	-	-	(Das et al., 2015)
	Termitomyces sp. 1		-	28.5	1.5	25.2	32.7	1.3	-	-	-	-	-	-	-	(Das et al., 2015)
	Termitomyces sp. 2		-	29.3	1.2	35	47.6	1.2	-	-	-	-	-	-	-	(Das et al., 2015)
	Termitomyces sp. 3		-	22.5	1.5	21.4	24.1	1.0	-	-	-	-	-	-	-	(Das et al., 2015)
	Termitomyces sp. 4		-	23.3	1.2	24.5	35.0	0.9	-	-	-	-	-	-	-	(Das et al., 2015)
	Volvariella sp		-	28.9	2.5	42	17.1	1.0	-	-	-	-	-	-	-	(Das et al., 2015)
	Agaricus sp.		-	26.9	2.0	39	14.4	0.9	-	-	-	-	-	-	-	(Das et al., 2015)
	Calvatia sp		-	15.8	1.2	23.4	14.7	1.4	-	-	-	-	-	-	-	(Das et al., 2015)
	Amanita sp. 3		-	18.6	1.6	15	34.6	1.8	-	-	-	-	-	-	-	(Das et al., 2015)
	Lentinus sp		-	23.8	1.9	12.5	39.7	1.0	-	-	-	-	-	-	-	(Das et al., 2015)
	Russula sp. 1		-	20.6	1.7	19	38.6	1.5	-	-	-	-	-	-	-	(Das et al., 2015)
	Russula sp. 2		-	23.3	3.0	18	13.5	0.7	-	-	-	-	-	-	-	(Das et al., 2015)
	Russula sp. 3		-	19.5	1.5	21	18.1	1.3	-	-	-	-	-	-	-	(Das et al., 2015)
	Russula sp. 4		-	20.7	1.7	22	20.1	1.2	-	-	-	-	-	-	-	(Das et al., 2015)
	Agaricus sp.	*Bali Chhatu*	-	25.6	0.2	-	-	-	82	-	-	-	-	-	-	(Jana, 2004)
	Marasmius sp	*Putca Chhatu*	-	26.3	0.3	-	-	-	9	-	-	-	-	-	-	(Jana, 2004)
**Field mushroom**	*Agaricus campestris*	*Parab Chhatu*	-	25.5	0.8	-	-	-	16	-	-	-	-	-	-	(Jana, 2004)

Common name	Botanical name	Vernacular name	Energy (kcal /100g)	Protein (g/100g)	Fat (g/ 100g)	Ca (mg /100g)	Iron (mg/ 100g)	Zinc (mg/ 100g)	Vit A (µg/ 100g)	Vit C (mg /100g)	Vit B1 (mg/ 100g)	Vit B2 (mg/ 100g)	Vit B3 (mg/ 100g)	Vit B6 (mg/ 100g)	Vit B9 (µg/ 100g)	Reference
**Branched Collybia**	*Agaricus racemosus*	*Patra Chhatu*	-	24.2	0.3	-	-	-	12	-	-	-	-	-	-	(Jana, 2004)
	Psalliota sp	*Mura Chhatu*	-	25.5	0.7	-	-	-	9	-	-	-	-	-	-	(Jana, 2004)
**Puffball mushroom**	*Calvatia gigantean*		-	27.3	1.0	630	10.7	10.3	-	14.9	-	-	-	-	-	(Agrahar-Murugkar and Subbulakshmi, 2005)
**Gray coral**	*Clavulina cinerea*		-	27.5	2.5	1910	75.2	11.1	-	41.8	-	-	-	-	-	(Agrahar-Murugkar and Subbulakshmi, 2005)
**Girolle**	*Cantharellus cibarius*		-	21.1	1.6	420	53.5	6.8	-	41.9	-	-	-	-	-	(Agrahar-Murugkar and Subbulakshmi, 2005)
	Ramaria brevispora Corner, K.S. Thind & Dev		-	24.1	1.3	530	7.2	6.8	-	28	-	-	-	-	-	(Agrahar-Murugkar and Subbulakshmi, 2005)
**Russula**	*Russula integra*		-	21.1	4.5	1270	56.2	10.5	-	19.6	-	-	-	-	-	(Agrahar-Murugkar and Subbulakshmi, 2005)
**Woolly chanterelle**	*Gomphus floccosus*		-	21.2	5.3	1370	22.3	13	-	25.8	-	-	-	-	-	(Agrahar-Murugkar and Subbulakshmi, 2005)
	Lactarius quieticolor Romag n.		-	19.0	2.6	1460	19.4	39.4	-	18.1	-	-	-	-	-	(Agrahar-Murugkar and Subbulakshmi, 2005)
Rugda	*Geastrum* sp.	*Rugda*	138	4.9	0.1	193.4	6.8	3.1	-	-	0.6	0.4	-	-	0	(Ghosh-Jerath et al., 2021)
Gitilud	NA	*Gitilud*	67	3.5	-	9.6	10.8	0.6	9	-	0.2	0.2	-	-	3	(Ghosh-Jerath et al., 2021)
Indiud	*Termitomyces albuminosa*	*Indiud*	38	2.2	0.2	4.8	4.0	0.3	-	-	1.5	0.3	-	-	5	(Ghosh-Jerath et al., 2021)
**White rot fungus**	*Termitomyces clypeatus*	*Koodeud/ Kundaud*	41	2.5	0.2	11.2	6.5	1	9	-	1.7	0.5	-	-	3	(Ghosh-Jerath et al., 2021)
	Pleurotus sajor-caju (Fr.) Singer		-	37.9	1.0	-	-	-	-	-	-	-	-	-	-	(Saha et al., 2014)
	Laetiporus sulphureus (Bull.) Murill		-	21.0	2.5	-	-	-	-	-	-	-	-	-	-	(Saha et al., 2014)
**Jew's ear**			-	15.3	10.1	-	-	-	-	-	-	-	-	-	-	(Saha et al., 2014)

Common name	Botanical name	Vernacular name	Energy (kcal /100g)	Protein (g/100g)	Fat (g/ 100g)	Ca (mg /100g)	Iron (mg/ 100g)	Zinc (mg/ 100g)	Vit A (µg/ 100g)	Vit C (mg /100g)	Vit B1 (mg/ 100g)	Vit B2 (mg/ 100g)	Vit B3 (mg/ 100g)	Vit B6 (mg/ 100g)	Vit B9 (µg/ 100g)	Reference
	Auricularia auricular-judae (Bull). J. Schrot	*Murukan Kumizh*	-	36.3	1.6	-	-	-	-	-	-	-	-	-	-	(Johnsy et al., 2011)
	Pleurotus roseus	*Vellathazan Kumizh*	-	30.3	2.0	-	-	-	-	-	-	-	-	-	-	(Johnsy et al., 2011)
**Oyster mushroom**	*Pleurotus ostreatus*	*Vellathazan Kumizh*	-	37.6	2.5	-	-	-	-	-	-	-	-	-	-	(Johnsy et al., 2011)
	Pleurotus sajor caju	*Vellathazan Kumizh*	-	39.1	1.2	-	-	-	-	-	-	-	-	-	-	(Johnsy et al., 2011)
	Termitomyces microcarpus	*Ari kumizh*	-	29.4	2.3	-	-	-	-	-	-	-	-	-	-	(Johnsy et al., 2011)
	Termitomyces heimii	*Puttu Kumizh*	-	34.2	2.1	-	-	-	-	-	-	-	-	-	-	(Johnsy et al., 2011)
**Straw mushroom**	*Volvariella volvacea*	*Vaikol Kumizh*	-	30.5	2.0	-	-	-	-	-	-	-	-	-	-	(Johnsy et al., 2011)
	Lentinus squarrosulus	*Kollaan Kumizh*	-	37.1	2.6	-	-	-	-	-	-	-	-	-	-	(Johnsy et al., 2011)
**King tuber mushroom**	*Lentinus tuberegium*	*Mulan Kumizh*	-	28.9	2.2	-	-	-	-	-	-	-	-	-	-	(Johnsy et al., 2011)
**Sheep's head**	*Grifola frondosa*	*Vella Murukan Kumizh*	-	31.5	1.5	-	-	-	-	-	-	-	-	-	-	(Johnsy et al., 2011)
**Flat Bulb mushroom**	*Agaricus abruptibulbus*	-	343	20.3	1.8	152	-	0.1	-	-	-	-	-	-	-	(Sudheep and Sridhar, 2014)
	Termitomyces globulus		373	23.8	4.3	101	0.2	1.2	-	-	-	-	-	-	-	(Sudheep and Sridhar, 2014)
**Splitgill mushroom**	*Schizophykm commune*	-	399	15.9	2	188	12.3	5.7	-	-	-	-	-	-	-	(Longvah and Deosthale, 1998)
**Shiitake**	*Lentinus edodes*	-	411	22.8	2.1	127	20.1	4.3	-	-	-	-	-	-	-	(Longvah and Deosthale, 1998)
							ROOTS & TUBERS									
**Shatavri**	*Asparagus racem osus* Willd.	-	402	6.7	10.3	120.3	21.2	2.1	-	45.8	-	-	70.7	-	-	(Arinathan et al., 2009)

Common name	Botanical name	Vernacular name	Energy (kcal /100g)	Protein (g/100g)	Fat (g/ 100g)	Ca (mg /100g)	Iron (mg/ 100g)	Zinc (mg/ 100g)	Vit A (µg/ 100g)	Vit C (mg /100g)	Vit B1 (mg/ 100g)	Vit B2 (mg/ 100g)	Vit B3 (mg/ 100g)	Vit B6 (mg/ 100g)	Vit B9 (µg/ 100g)	Reference
**Kali Musli**	*Curculigo orchioides* Gaertn.	-	367	9.6	4.4	440.3	124	2.5	-	14.4	-	-	23.9	-	-	(Arinathan et al., 2009)
**Potato Yam**	*Dioscorea bulbifera* L.	*Haranbho /Piski sanga Gethi Kanda/Pita Alu/Chedu dumpa/Pita Aalu*	428	5.2	9.1	238.2	4.9	1.3	-	106.5	-	-	23.7	-	-	(Arinathan et al., 2009)
			403	7.3	6.1	338.2	19.2	1.5	-	91	-	-	33.7	-	-	(Shajeela et al., 2011)
			44	2.1	-	4.9	1.8	0.1	-	-	1.8	5.1	-	-	1	(Ghosh-Jerath et al., 2021)
			95	2.4	-	20	4.1	0.4	30	4	-	-	-	-	3	(Ghosh-Jerath et al., 2015)
			-	1.1	0.1	-	-	-	-	5.1	-	-	-	-	-	(Jana, 2004)
			132	3.4	1.1	56	6.4	-	-	-	0.0	0.0	0.1	-	-	(Rajyalakshmi and Geervani, 1994)
			-	8.6	0.6	-	-	-	-	4.3	-	-	-	-	-	(Padhan et al., 2020)
**Indian yam**	*Dioscorea oppositifolia* L.	*Pan Alu/Arika tega/Paani Alu*	409	13.8	6.3	230	49.1	1.4	-	104.7	-	-	17.6	-	-	(Arinathan et al., 2009)
			406	13.4	7.4	294.1	32.1	1.6	-	96.4	-	-	37.1	-	-	(Shajeela et al., 2011)
			350	6.3	2.5	880.6	32	5.2	-	80.6	-	-	64.7	-	-	(Arinathan et al., 2009)
			369	8.4	4.4	646.2	40.8	6.3	-	90.5	-	-	44.3	-	-	(Shajeela et al., 2011)
			386	7.0	6.9	680.6	22.0	3.2	-	-	-	-	-	-	-	(Mohan and Kalidass, 2010)
			-	1.4	0.1	-	-	-	-	2	-	-	-	-	-	(Jana, 2004)
			100	1.8	1.1	45	4.7	-	-	-	0.0	0.0	0.3	-	-	(Rajyalakshmi and Geervani, 1994)
			-	9.5	1.6	-	-	-	-	5.7	-	-	-	-	-	(Padhan et al., 2020)
**Five leaf yam**	*Dioscorea pentaphylla* L.	*Nappe/Hasaer Sanga/Kanta Alu Pandimukku tega/Panja sanga*	395	5.4	6.0	640.1	113.4	3.2	-	91.7	-	-	53.5	-	-	(Arinathan et al., 2009)
			388	6.5	6.2	444.2	66.3	3.4	-	96.6	-	-	62.1	-	-	(Shajeela et al., 2011)
			72	4.4	-	33.2	55.9	0.6	-	3.1	1.1	-	-	-	-	(Ghosh-Jerath et al., 2020)
			384	9.2	4.8	632.1	103	3.1	-	-	-	-	-	-	-	(Mohan and Kalidass, 2010)
			-	1.5	0.3	-	-	-	-	7	-	-	-	-	-	(Jana, 2004)
			72	2.8	0.7	139	7.2	-	-	-	0.0	0.0	0.1	-	-	(Rajyalakshmi and Geervani, 1994)
			-	9.2	0.6	-	-	-	-	4.2	-	-	-	-	-	(Padhan et al., 2020)
**Nurai**	*Dioscorea tomentosa* J.Koenig ex Spreng.	-	409	8.5	5.9	240.3	23.7	6.2	-	55.7	-	-	88.4	-	-	(Arinathan et al., 2009)
			402	9.5	6.0	266.4	28.5	5.4	-	65.2	-	-	74.1	-	-	(Shajeela et al., 2011)
			389	8.3	6.8	272.1	24.6	5.2	-	-	-	-	-	-	-	(Mohan and Kalidass, 2010)
**Wild butter bean**	*Dolichos trilobus* L .	*Minnikishangu*	429	7.1	10.8	680	16.6	4.4	-	57.3	-	-	20.0	-	-	(Arinathan et al., 2009)
**Red Ginger**	*Zingiber* sp	*Kekir*	-	2.8	2.3	-	-	-	-	24.3	-	-	-	-	-	(Bhardwaj et al., 2009)

Common name	Botanical name	Vernacular name	Energy (kcal /100g)	Protein (g/100g)	Fat (g/ 100g)	Ca (mg /100g)	Iron (mg/ 100g)	Zinc (mg/ 100g)	Vit A (µg/ 100g)	Vit C (mg /100g)	Vit B1 (mg/ 100g)	Vit B2 (mg/ 100g)	Vit B3 (mg/ 100g)	Vit B6 (mg/ 100g)	Vit B9 (µg/ 100g)	Reference
**Ginger**	*Zingiber officinale* Roscoe	*Takeng*	-	2.1	2.1	-	-	-	-	18.1	-	-	-	-	-	(Bhardwaj et al., 2009)
**Junglikhand**	NA	Cooked	63	0.9	0.1	31	2.5	0.5	-	-	-	-	-	-	-	(Bhattacharjee et al., 2009)
		Raw	72	1.4	0.3	10	0.8	0.3	-	-	-	-	-	-	-	(Bhattacharjee et al., 2009)
**Alli**	NA	*Alli*	195	3.7	-	43.4	7	0.5	-	1.2	15.0	-	-	-	-	(Ghosh-Jerath et al., 2020)
**Chalangan/ Chalango**	NA	*Chalangan/ Chalango*	133	3.6	-	151.5	9.5	1.2	-	1.2	1.1	0.9	-	-	-	(Ghosh-Jerath et al., 2020)
**Elephant foot yam**	*Amorphophallusp aeoniifolius* (Denn st.) Nicolson	*Singla*	64	6.3	-	35.7	11.1	1.1	-	3.1	-	-	-	-	-	(Ghosh-Jerath et al., 2020)
**Alligator yam**	*Ipomea digitate*	*Bhui Kumro/Kha ma Alu*	-	3.8	0.7	-	-	-	-	92	-	-	-	-	-	(Jana, 2004)
			-	1.3	0.1	-	-	-	-	5	-	-	-	-	-	(Jana, 2004)
**Purple Yam**	*Dioscorea alata* L.	*Haatikata/ Aaru/ Jat sanga*	395	7.6	5.3	448.4	24.3	2.3	-	74.6	-	-	36.2	-	-	(Shajeela et al., 2011)
			126	3.3	0.3	10.7	3.8	0.4	11	5.6	6.0	11.1	-	-	-	(Ghosh-Jerath et al., 2021)
	Dioscorea floribun da M.Martens & Galeotti	*Khamba aalu/Chun Alu*	-	-	-	138.2	59.6	-	115	80.6	-	-	-	-	-	(Singh et al., 2018)
			-	9.0	1.0	-	-	-	-	5	-	-	-	-	-	(Padhan et al., 2020)
			-	2.3	0.1	-	-	-	-	3	-	-	-	-	-	(Jana, 2004)
Churka Alu	*Dioscorea glabra* Roxb.	*Churka Alu*	-	1.6	0.1	-	-	-	-	2.9	-	-	-	-	-	(Jana, 2004)
			-	8.6	0.6	-	-	-	-	4.1	-	-	-	-	-	(Padhan et al., 2020)
Mou Alu	*Dioscorea wallichi i* Hook.f.	*Mou Alu*	-	1.8	0.6	-	-	-	-	4	-	-	-	-	-	(Jana, 2004)
			360	10.8	3.3	748.3	20.1	6.7	-	88.3	-	-	52.4	-	-	(Shajeela et al., 2011)
			-	8.4	0.9	-	-	-	-	5.4	-	-	-	-	-	(Padhan et al., 2020)
Bon Ol	*Amorphophallus s ylvaticus* (Roxb.) Kunth	*Bon Ol*	-	1.2	0.3	-	-	-	-	24	-	-	-	-	-	(Jana, 2004)
**Floating lace plant**	*Aponogeton nata ns* (L.) Engl. & K.Krause		386	5.3	2.9	312.3	30.1	2.2	-	-	-	-	-	-	-	(Mohan and Kalidass, 2010)
	Boerhavia chinens is (L.) Rottb.		387	6.1	4.9	224.2	23.1	1.6	-	-	-	-	-	-	-	(Mohan and Kalidass, 2010)

Common name	Botanical name	Vernacular name	Energy (kcal /100g)	Protein (g/100g)	Fat (g/ 100g)	Ca (mg /100g)	Iron (mg/ 100g)	Zinc (mg/ 100g)	Vit A (µg/ 100g)	Vit C (mg /100g)	Vit B1 (mg/ 100g)	Vit B2 (mg/ 100g)	Vit B3 (mg/ 100g)	Vit B6 (mg/ 100g)	Vit B9 (µg/ 100g)	Reference
**Hadjod/Veldt grape**	*Cissus quadrangularis* L.		389	6.1	6.4	94.0	34.0	3.2	-	-	-	-	-	-	-	(Mohan and Kalidass, 2010)
**Kattumunthiri**	*Cissus vitiginea* L.		364	3.9	2.2	316.1	26.1	1.8	-	-	-	-	-	-	-	(Mohan and Kalidass, 2010)
**Colocasia**	*Colocasia esculent a* (L.) Schott	*Saaru /Toti /Ghuniya*	374	4.4	5.3	538.3	62.2	2.0	-	-	-	-	-	-	-	(Mohan and Kalidass, 2010)
			90	3.3	0.2	30.2	0.7	0.4	7	1.8	0.1	0.0	-	-	20	(Longvah et al., 2017)
			73	3.2	0.1	1.8	1.4	0.2	-	-	0.1	0.3	-	-	-	(Ghosh-Jerath et al., 2021)
			-	-	-	100	27	-	110	250	-	-	-	-	-	(Singh et al., 2018)
**Queen sago**	*Cycas circinalis* L.	-	387	9.2	4.1	418.3	31	1.5	-	-	-	-	-	-	-	(Mohan and Kalidass, 2010)
	Cyphostemma set osum (Roxb.) Alston		375	4.4	5.8	346.1	28	1.4	-	-	-	-	-	-	-	(Mohan and Kalidass, 2010)
**Swallow root**	*Decalepis hamilto nii* Wight & Arn.	-	399	4.4	10.2	386.1	51.1	2.2	-	-	-	-	-	-	-	(Mohan and Kalidass, 2010)
	Dioscorea hamilto nii Hook.f.		-	10.0	2.0	-	-	-	-	5.7	-	-	-	-	-	(Padhan et al., 2020)
Athikizhangu	*Dioscorea spicata* Roth	*Athikizhang u*	384	6.4	4.8	172	22.3	4.2	-	-	-	-	-	-	-	(Mohan and Kalidass, 2010)
			369	8.2	3.3	234.1	24.1	2.6	-	76	-	-	54.4	-	-	(Shajeela et al., 2011)
**Anantmul**	*Hemidesmus indic us* (L.) R. Br. ex Schult.	-	405	4.4	6.2	432	44.1	2.2	-	-	-	-	-	-	-	(Mohan and Kalidass, 2010)
	Ipomoea sumatra na (Miq.) Ooststr.		400	5.3	5.7	524.2	25.2	14.6	-	-	-	-	-	-	-	(Mohan and Kalidass, 2010)
	Kedrostis foetidiss ima (Jacq.) Cogn.		373	11.4	5.1	371	34	1.8	-	-	-	-	-	-	-	(Mohan and Kalidass, 2010)
	Maerua oblongifo lia (Forssk.) A.Rich.		410	7.9	7.8	218.1	29.1	1.4	-	-	-	-	-	-	-	(Mohan and Kalidass, 2010)
**Koka**	*Nymphaea pubes cens* Willd.		374	9.6	3.0	326.1	32.1	1.3	-	-	-	-	-	-	-	(Mohan and Kalidass, 2010)
**Water lily**	*Nymphaea rubra* Roxb. ex Andrews		394	8.3	5.1	354.1	28.1	1.6	-	-	-	-	-	-	-	(Mohan and Kalidass, 2010)
	Parthenocissus ne ilgherriensis Planc h.		374	5.3	4.9	372	29	1	-	-	-	-	-	-	-	(Mohan and Kalidass, 2010)

Common name	Botanical name	Vernacular name	Energy (kcal /100g)	Protein (g/100g)	Fat (g/ 100g)	Ca (mg /100g)	Iron (mg/ 100g)	Zinc (mg/ 100g)	Vit A (µg/ 100g)	Vit C (mg /100g)	Vit B1 (mg/ 100g)	Vit B2 (mg/ 100g)	Vit B3 (mg/ 100g)	Vit B6 (mg/ 100g)	Vit B9 (µg/ 100g)	Reference
**Indian three-leaved yam**	*Dioscorea hispida* Dennst.	*Pulidumpa/ Banya aalu*	134	5.2	4.3	86	4.7	-	-	-	0.1	-	0.4	-	-	(Rajyalakshmi and Geervani, 1994)
			-	5.4	0.6	-	-	-	-	1.7	-	-	-	-	-	(Padhan et al., 2020)
**Indiatic yam/Karen potato**	*Dioscorea esculenta* (Lour.) Burkill		375	9.8	4.7	314	11.5	1.8	-	84.1	-	-	41.4	-	-	(Shajeela et al., 2011)
	Panax bipinnatifidus Seem.		-	2.1	2.0	750	-	-	-	-	-	-	-	-	-	(Tag et al., 2014)
	Dioscorea pubera Blume	*Kukai sanga*	-	1.1	-	-	-	-	-	-	-	-	-	-	-	(Terangpi and Teron, 2015)
			-	10.3	1.6	-	-	-	-	9.4	-	-	-	-	-	(Padhan et al., 2020)
**Tapioca**	*Manihot esculenta* Crantz.	*Adel sanga*	80	1.0	0.2	25.9	0.8	0.1	-	17.6	0.1	-	-	-	26	(Longvah et al., 2017)
**Aromatic ginger**	*Kaempferia galanga* L.	*Sying smoh*	-	4.7	10.2	950	69.9	8.4	182	-	-	-	-	-	-	(Agrahar-Murugkar and Subbulakshmi, 2005)
**Flemingia root**	*Flemingia procumbens* Roxb	*Soh phlang*	-	5.4	0.4	1440	1.7	0.2	-	419	-	-	-	-	-	(Agrahar-Murugkar and Subbulakshmi, 2005)
			119	2.8	0.2	159	1.3	2.8	-	15.7	0.01	0.1	0.6	0.1	20.3	(Chyne et al., 2019)
**Fish mint**	*Houttuynia cordata* Thunb.	*Jamyrdoh*	161	12.2	2.1	825	98.4	9.9	-	-	-	-	-	-	-	(Seal, 2011)
	Potentilla lineata Trevir.	*Lynniang*	88.3	28.1	1.9	1080	63.6	6	-	-	-	-	-	-	-	(Seal, 2011)
**Potato**	*Solanum tuberosum* L.	*Phan san minit*	94	2.9	0.2	6.5	0.6	1.2		0.3	0.1	0.04	1.5	0.04	239	(Chyne et al., 2019)
	Alocasia acuminata Schott.	*Shriew khmat blang*	114	2.01	0.2	36.1	0.6	0.9		1.2	0.1	0.04	0.8	0.2	17.7	(Chyne et al., 2019)
**Cinquefoil roots**	*Potentilla polyphylla* Wall. ex Lehm.	*Lynniang*	121	3.2	0.6	194	9.1	0.5		7.4	1.02	0.1	0.9	0.1	19.9	(Chyne et al., 2019)
**Sweet potato (white)**	*Ipomoea batatas* (L.) Lam.	*Phan karo lieh*	139	1.9	0.3	20	0.5	0.5		0.3	0.1	0.1	0.6	0.04	426	(Chyne et al., 2019)
**Sweet potato (red)**	*Ipomoea batatas* (L.) Lam.	*Phan karo saw*	67	1.7	0.3	22.1	0.4	0.8		4.6	0.1	0.04	0.7	0.1	264	(Chyne et al., 2019)
FRUITS																
		Amla	24	0.3	0.2	20.1	1.3	0.1	2	252	0.0	0.0	0.1	-	8	(Longvah et al., 2017)

Common name	Botanical name	Vernacular name	Energy (kcal /100g)	Protein (g/100g)	Fat (g/ 100g)	Ca (mg /100g)	Iron (mg/ 100g)	Zinc (mg/ 100g)	Vit A (µg/ 100g)	Vit C (mg /100g)	Vit B1 (mg/ 100g)	Vit B2 (mg/ 100g)	Vit B3 (mg/ 100g)	Vit B6 (mg/ 100g)	Vit B9 (µg/ 100g)	Reference
**Indian goosberry**	*Phyllanthus emblica* L.	*Amlaki*	-	0.5	0.1	-	-	-	-	600	-	-	-	-	-	(Jana, 2004)
**Indian jujube**	*Ziziphus jujuba* Mill.	Bore/Godaari/Ilkarpu/Ber	49	1.3	0.4	46.6	0.4	0.1	2	60.9	0.0	0.0	0.3	-	6	(Longvah et al., 2017)
	Zizyphus sp	*Charkul*	-	0.8	0.3	-	-	-	-	76	-	-	-	-	-	(Jana, 2004)
	Zizyphus sp.	*Kankul*	-	3.2	1.3	-	-	-	-	96	-	-	-	-	-	(Jana, 2004)
**Sandpaper fig**	*Ficus exasperata* Vahl.	*Haani*	60	3.0	-	275.2	6.6	0.4	-	2.1	0.3	-	-	-	-	(Ghosh-Jerath et al., 2020)
**Monkey jack**	*Artocarpus lakoocha* Roxb.	*Dahu*	121	2.8	-	54.7	1.8	0.1	1843	8.9	0.3	1.3	-	-	-	(Ghosh-Jerath et al., 2020)
**Wild fig**	*Ficus racemosa* L.	*Dumari/Dumar/Loa/Umbur*	52	3.2	-	84	1.5	0.4	-	-	-	-	-	-	1	(Ghosh-Jerath et al., 2020)
**Kusum**	*Schleichera oleosa* (Lour.) Merr.	Pusra/Baru	144	6.3	-	134.9	44.2	0.8	6238	3.1	-	-	-	-	-	(Ghosh-Jerath et al., 2020)
**Bhui-gular/ Khaina/ Khunia**	*Ficus semicordata* Buch.-Ham. Ex Sm.	*Anni/Aanri*	57	3.1	-	3.6	0.4	0.1	8.9	-	-	0.2	-	-	-	(Ghosh-Jerath et al., 2020)
	Euphorbia ingens E.Mey. ex Boiss**.**	*Ashfal*	-	1.4	5.4	-	-	-	-	209	-	-	-	-	-	(Jana, 2004)
**Wood apple**	*Aegle marmelos* (L.) Correa	*Bel*	-	1.8	0.3	-	-	-	-	8	-	-	-	-	-	(Jana, 2004)
		Sinju/Bel	136	2.6	0.6	47.9	0.2	0.1	3	7.5	0.0	0.0	-	-	55	(Longvah et al., 2017)
**Date**	*Phoenix* sp.	*Bon Khejur*	-	1.9	2.5	-	-	-	-	2	-	-	-	-	-	(Jana, 2004)
	Flacourtia amalotricha A.C.Sm.	*Bainch Kul*	-	0.5	0.1	-	-	-	-	89	-	-	-	-	-	(Jana, 2004)
**Cape jasmine**	*Gardenia gummifera* L.f.	Bhurur	-	2.0	0.3	-	-	-	-	225	-	-	-	-	-	(Jana, 2004)
**Java Plum**	*Syzygium cumini* (L.) Skeels	*Jam*	-	0.7	0.3	-	-	-	-	18	-	-	-	-	-	(Jana, 2004)
**Tumki**	*Diospyros melanoxylon* Roxb.	*Kendu*	-	0.8	0.2	-	-	-	-	1	-	-	-	-	-	(Jana, 2004)
		Tiril / Kendu/Kaanda	112	0.8	0.2	60	0.5	-	361	1	0.0	0.0	-	-	-	(National Institute of Nutrition et al., 1978)

Common name	Botanical name	Vernacular name	Energy (kcal /100g)	Protein (g/100g)	Fat (g/ 100g)	Ca (mg /100g)	Iron (mg/ 100g)	Zinc (mg/ 100g)	Vit A (µg/ 100g)	Vit C (mg /100g)	Vit B1 (mg/ 100g)	Vit B2 (mg/ 100g)	Vit B3 (mg/ 100g)	Vit B6 (mg/ 100g)	Vit B9 (µg/ 100g)	Reference
	Zanthoxylum rhetsa DC	*Tirphal/Chirphal.*	-	-	-	144.8	9.5	1.1	-	-	-	-	-	-	-	(Mahadkar et al., 2012)
**Himalayan strawberry**	*Cornus capitata* Wall.	-	-	2.6	2.5	-	-	-	-	-	-	-	-	-	-	(Saha et al., 2014)
**Malabar ebony**	*Diospyros malabarica* (Desr.) Kostel.		-	3.5	2.0	-	-	-	-	-	-	-	-	-	-	(Saha et al., 2014)
	Holboellia latifolia Wall.		-	8.4	7.5	-	-	-	-	-	-	-	-	-	-	(Saha et al., 2014)
**Lissi**	*Illicium griffithii* Hook.f. & Thomson		-	6.1	1.1	-	-	-	-	-	-	-	-	-	-	(Saha et al., 2014)
	Machilus robusta W.W. Sm.		-	12.7	22.5	-	-	-	-	-	-	-	-	-	-	(Saha et al., 2014)
	Ocotea lancifolia (Schott) Mez		-	15.2	12.5	-	-	-	-	-	-	-	-	-	-	(Saha et al., 2014)
**Sikkim crabapple**	*Malus sikkimensis* (Wenz.) Koehne ex C.K.Schneid.	-	-	1.8	0.4	-	-	-	-	-	-	-	-	-	-	(Saha et al., 2014)
**Autumn Olive**	*Elaeagnus umbellata* Thunb.	-	-	15.1	4.4	810	-	-	-	-	-	-	-	-	-	(Tag et al., 2014)
**Ambada**	*Spondias pinnata* (L. f.) Kurz.	Amda	48	0.7	3.0	36	3.9	-	-	-	-	-	-	-	-	(National Institute of Nutrition et al., 1978)
**Mahua, ripe**	*Madhuca longifolia* (J. Koenig. ex L.) J.F. Macbr.	Mahua/Mahvada	111	1.4	1.6	45	0.2	-	307	40	-	-	-	-	-	(National Institute of Nutrition et al., 1978)
**Marking nut (kernel)**	*Semecarpus anacardium* L.f.	*Kero/Keero Toso/Soso/Bhelua*	587	26.4	36.4	295	6.1	-	-	-	-	-	-	-	-	(National Institute of Nutrition et al., 1978)
**Palmyra fruit, ripe**	*Borassus flabellifer* L.	Talmi/Tamras	101	0.5	0.1	-	-	0.1	-	0.3	0.0	-	-	-	24	(National Institute of Nutrition et al., 1978)
**Banyan fruit**	*Ficus benghalensis* L.	*Pakkedi/Badi*	72	1.7	2.0	364	-	-	-	-	-	-	-	-	-	(National Institute of Nutrition et al., 1978)

Common name	Botanical name	Vernacular name	Energy (kcal /100g)	Protein (g/100g)	Fat (g/ 100g)	Ca (mg /100g)	Iron (mg/ 100g)	Zinc (mg/ 100g)	Vit A (µg/ 100g)	Vit C (mg /100g)	Vit B1 (mg/ 100g)	Vit B2 (mg/ 100g)	Vit B3 (mg/ 100g)	Vit B6 (mg/ 100g)	Vit B9 (µg/ 100g)	Reference
Zoge	*Melodinus cochin chinensis* (Lour.) Merr	*Zoge*	405	7.2	6.4	1207	22	43	-	-	-	-	-	-	-	(Seal et al., 2016)
**Black current**	*Antidesma ghaese mbilla* Gaertn.	*Nuniari /Kattu-pulinchi*	-	1.3	-	3.3	5.6	-	-	-	-	-	-	-	-	(Nayak and Basak, 2015)
			64	1.8	0.9	-	0.8	-	-	111.2	-	-	-	-	-	(Nazarudeen, 2010)
**Wild guava**	*Careya arborea* R oxb.	*Kumbhi*	-	0.9	-	2.7	4.2	-	-	-	-	-	-	-	-	(Nayak and Basak, 2015)
**Fukian tea**	*Ehretia microphyll a* Lam.	*Kujipana*	-	4.1	-	1.2	5.9	-	-	-	-	-	-	-	-	(Nayak and Basak, 2015)
Kanta beta	*Calamus guruba* B uch. -Ham. ex Mart.	*Kanta beta*	-	1.2	-	1.7	8.5	-	-	-	-	-	-	-	-	(Nayak and Basak, 2015)
**Ram Phal**	*Dillenia pentagyn a* Roxb.	*Rai*	-	0.3	-	4.5	16	-	-	-	-	-	-	-	-	(Nayak and Basak, 2015)
**Phalgu**	*Ficus hispida* L.f.	*Dimiri/Dimu r*	-	1.1	-	5	6.2	-	-	-	-	-	-	-	-	(Nayak and Basak, 2015)
**Malabar melastome**	*Melastoma malab athricum* L.	*Karati*	-	5.5	-	2.5	8	-	-	-	-	-	-	-	-	(Nayak and Basak, 2015)
**Sewra**	*Streblus asper* Lou r.	*Sahara*	-	1.5	-	3	10	-	-	-	-	-	-	-	-	(Nayak and Basak, 2015)
	Alangium salviifoli um subsp. *hexape talum* (Lam.) Wangerin	*Ottanghadi, Kilikhuti-ppazham*	55	3.9	0.2	-	-	-	-	117.0	-	-	-	-	-	(Nazarudeen, 2010)
	Aporosa cardiosp erma (Gaertn.) Merr.	*Ponvetti, Vetti*	34	0.0	1.2	-	3.7	-	-	-	-	-	-	-	-	(Nazarudeen, 2010)
	Baccaurea courtal lensis (Wight) Müll.Arg.	*Mootippuli, Mootti*	52	0.9	2.1	-	1.6	-	-	-	-	-	-	-	-	(Nazarudeen, 2010)
**Wild Rhea**	*Debregeasia longi folia* (Burm.f.) Wedd.	*Neerinch, Monilli*	73	3.0	2.3	-	7.3	-	-	3.9	-	-	-	-	-	(Nazarudeen, 2010)
		Jallatyrsim	342	11.9	2.4	1949	300	51	-	-	-	-	-	-	-	(Seal and Chaudhuri, 2014)

Common name	Botanical name	Vernacular name	Energy (kcal /100g)	Protein (g/100g)	Fat (g/ 100g)	Ca (mg /100g)	Iron (mg/ 100g)	Zinc (mg/ 100g)	Vit A (µg/ 100g)	Vit C (mg /100g)	Vit B1 (mg/ 100g)	Vit B2 (mg/ 100g)	Vit B3 (mg/ 100g)	Vit B6 (mg/ 100g)	Vit B9 (µg/ 100g)	Reference
**Malabar tamarind**	*Garcinia gummi-gutta* (L.) Roxb.	*Kodampuli, Pinampuli*	39	0.6	0.2	-	-	-	-	-	-	-	-	-	-	(Nazarudeen, 2010)
**Jungle geranium**	*Ixora coccinea* L.	*Thetti, Chethi, Thechi*	66	0.3	0.0	-	-	-	-	-	-	-	-	-	-	(Nazarudeen, 2010)
**Spanish cherry/Maulsari**	*Mimusops elengi* L.	*Elengi*	91	1.3	2.8	-	0.6	-	-	3.3	-	-	-	-	-	(Nazarudeen, 2010)
**Palai**	*Palaquium ellipticum* (Dalzell) Baill.	*Pali, Bali*	108	1.4	7.0	-	0.3	-	-	4.9	-	-	-	-	-	(Nazarudeen, 2010)
**Tamilnadia**	*Tamilnadia uliginosa* (Retz.) Tirveng. & Sastre	*Pindichakka*	40	0.5	0.2	-	1	-	-	62	-	-	-	-	-	(Nazarudeen, 2010)
Soh Priam Khlaw	*Helicia nilagirica* Bedd.	*Soh Priam Khlaw*	378	3.3	1.1	206	15	27	-	-	-	-	-	-	-	(Seal and Chaudhuri, 2014)
Soh Phoh Khlaw	*Ilex venulosa* Hook.f.	*Soh Phoh Khlaw*	374	3.3	1.3	918	22	48	-	-	-	-	-	-	-	(Seal and Chaudhuri, 2014)
Soh-mlum/Sohma	*Rhus chinensis* Mill.	*Soh-mlum/Sohma*	385	7.9	5.8	335	14	40	-	-	-	-	-	-	-	(Seal and Chaudhuri, 2014)
Raitung	*Rhus parviflora* Roxb	*Raitung*	-	5.1	24.5	-	-	-	-	-	-	-	-	-	-	(Jain and Tiwari, 2012)
**Gamari**	*Gmelina arborea* Roxb.	*Shivan*	-	-	-	170.4	14.7	2.7	-	-	-	-	-	-	-	(Mahadkar et al., 2012)
**Indian trumpet flower**	*Oroxylum indicum* (L.) Kurz	*Tetu*	-	-	-	731.2	9.5	1.6	-	-	-	-	-	-	-	(Mahadkar et al., 2012)
**Gurial**	*Bauhinia racemosa* Lam.	*Apata*	-	-	-	2264	2.5	2.4	-	-	-	-	-	-	-	(Mahadkar et al., 2012)
**Jaggery palm**	*Caryota urens* L.	*Ardhashishi*	-	-	-	144.8	46	1.1	-	-	-	-	-	-	-	(Mahadkar et al., 2012)
Sohlang	*Viburnum corylifolium* Hook. f. & Thomson	*Sohlang*	-	5.5	11.2	630	3.6	1.6	-	238.7	-	-	-	-	-	(Agrahar-Murugkar and Subbulakshmi, 2005)
**Bird cherry**	*Prunus bracteopadus* Koehne	*Sohlang/ Soh iong*	-	4.6	0.7	1220	10.7	1.5	257	608.9	-	-	-	-	-	(Agrahar-Murugkar and Subbulakshmi, 2005)

Common name	Botanical name	Vernacular name	Energy (kcal /100g)	Protein (g/100g)	Fat (g/ 100g)	Ca (mg /100g)	Iron (mg/ 100g)	Zinc (mg/ 100g)	Vit A (µg/ 100g)	Vit C (mg /100g)	Vit B1 (mg/ 100g)	Vit B2 (mg/ 100g)	Vit B3 (mg/ 100g)	Vit B6 (mg/ 100g)	Vit B9 (µg/ 100g)	Reference
			83	1.9	0.9	18.4	0.3	0.3	-	43.4	0.04	0.1	0.6	0.1	124	(Chyne et al., 2019)
**Maiyan**	*Meyna spinosa* Roxb. ex Link	*Sohmaten/ Heibimana*	-	2.7	0.4	520	2.1	22.9	-	-	-	-	-	-	-	(Agrahar-Murugkar and Subbulakshmi, 2005)
			-	3.5	0.6	192	4.4	0.9	-	13.3	-	-	-	0.1	235	(Loukrakpam et al., 2019)
Sohthliem	*Gomphogyne cissiformis*	*Sohthliem*	-	14.4	3.6	1170	6.2	3.8	-	273.5	-	-	-	-	-	(Agrahar-Murugkar and Subbulakshmi, 2005)
**Bayberry**	*Myrica esculenta* Buch.-Ham. ex D. Don	*Soh phie heh*	34	1.2	0.8	16.1	0.6	0.5	-	10.8	0.02	0.04	0.5	0.1	104	(Chyne et al., 2019)
	Myrica nagi Thunb.	*Soh phie nam*	47	1.6	1.6	14.1	0.5	0.4	-	28.2	0.02	0.02	0.8	0.1	120	(Chyne et al., 2019)
**Indian wild Pear**	*Pyrus pashia* Buch .-Ham. ex D.Don	*Soh jhur*	-	1.7	0.9	650	-	-	-	-	-	-	-	-	-	(Tag et al., 2014)
			49	0.4	0.8	23.4	12.1	0.1	-	16.2	0.01	0.01	0.8	0.04	114	(Chyne et al., 2019)
			-	3.3	0.5	-	-	-	-	-	-	-	-	-	-	(Saha et al., 2014)
**Cane fruit**	*Calamus erectus* Roxb.	*Soh thri*	82	1.5	1.2	14.2	0.2	0.2	-	36.2	0.01	0.02	0.7	0.1	111	(Chyne et al., 2019)
**Rough lemon**	*Citrus jambhiri*	*Soh jalia*	56	0.8	1.1	56	0.2	0.1	-	35	0.01	0.03	0.5	0.1	101	(Chyne et al., 2019)
**Himalayan evergreen viburnum**	*Viburnum foetidum* Wall.	*Soh lang*	83	1.5	0.9	58.8	1.9	0.9	-	40.1	0.01	0.01	0.9	0.03	378	(Chyne et al., 2019)
**Korean mulberry**	*Morus australis* Poir.	*Soh lang dkhur*	76	3.6	1.6	18.6	0.5	0.4	-	31.3	0.01	0.02	0.4	0.1	100	(Chyne et al., 2019)
							FLESH FOODS									
**Snail, big**	*Pila globoasa*	*Loa suti*	97	10.5	0.6	870.0	-	-	-	-	-	-	-	-	-	(National Institute of Nutrition et al., 1978)
**Red ants with eggs**	*Oceophylla smaragdina*	*Demta/Hau anda/ Chetado ka anda*	131	13.4	4.6	104	-	-	-	-	-	-	-	-	-	(National Institute of Nutrition et al., 1978)
**Field rat's meat**	*Rattus argentiventer*	*Moosa/ Gudu*	104	23.6	1.0	30	-	-	-	-	-	-	-	-	-	(National Institute of Nutrition et al., 1978)
**Pigeon**	*Columba livia domestica*	*Kabutar/ Pervan*	126	17.9	6.0	18.1	3.8	2.4	15	-	0.2	0.4	-	-	8	(Longvah et al., 2017)

Common name	Botanical name	Vernacular name	Energy (kcal /100g)	Protein (g/100g)	Fat (g/ 100g)	Ca (mg /100g)	Iron (mg/ 100g)	Zinc (mg/ 100g)	Vit A (µg/ 100g)	Vit C (mg /100g)	Vit B1 (mg/ 100g)	Vit B2 (mg/ 100g)	Vit B3 (mg/ 100g)	Vit B6 (mg/ 100g)	Vit B9 (µg/ 100g)	Reference
**Puti**	*Burbus* spp.	*Pothi hako/ Potha Hako*	106	18.1	2.4	110	1	-	15	-	-	0.3	-	-	-	(National Institute of Nutrition et al., 1978)
**Freshwater mussels**	*Margaritifera margaritifera*	*Setua/ Keyosuti/ Maako/jhinuk*	81	14.5	1.6	592	-	-	-	-	-	-	-	-	-	(National Institute of Nutrition et al., 1978)
**Prawn**	*Macrobrachium* sp	*Chingra*	80	13.1	0.8	68	0.9	0.9	3	-	-	-	0.5	207	1306	(Longvah et al., 2017)
**Singhi**	*Saccobranchus fossilis*	*Singhi*	124	22.8	0.6	670	2.3	-	-	-	-	0.8	-	-	-	(National Institute of Nutrition et al., 1978)
**Crab**	*Pachygrapsus* sp	*Kenkda*	78	13.2	0.9	199	1.1	2.5	12.55	-	0.01	0.1	1.5	202	1783	(Longvah et al., 2017)
**Turtle's meat**	*Testudinata*	*Kachua*	86	16.5	1.5	7	-	-	-	-	-	-	-	-	-	(National Institute of Nutrition et al., 1978)
**Beef, chops**	*Bos Taurus*	*Gai*	140	19.8	6.7	4.6	1.9	3.7	2	-	-	0.1	4.4	0.3	6	(Longvah et al., 2017)
**Beef (Lean Ground)**			-	27.4	-	-	3.5	-	-	-	-	-	-	-	-	(Das and Singson, 2016)
**Duck**	*Anas platyrhynchos*	*Batakh*	131	19.1	6.0	22.8	4.3	2.9	49	-	0.2	0.1	2.2	0.3	28	(Longvah et al., 2017)
**Egg, duck**	*Anas platyrhynchos*	*Batakh anda*	182	14.6	13.8	52.5	2.5	1.5	181	-	0.2	0.1	0.1	0.2	76	(Longvah et al., 2017)
**Bombay duck**	*Harpodon nehereus*	*Bubla*	90	13.5	1.0	159	0.8	0.4	3	-	-	-	0.6	98.0	2784	(Longvah et al., 2017)
**Walking catfish**	*Clarias batrachus*	*Magur/Mangri*	86	15.0	1.0	210	0.7	-	-	-	-	-	-	-	-	(National Institute of Nutrition et al., 1978)
**Wallago**	*Wallago attu*	*Boari*	116	15.4	2.7	160	1	-	-	8	-	-	-	-	-	(National Institute of Nutrition et al., 1978)
**Freshwater Eel**	*Anguilla Anguilla*	*Gacchi*	108	20.4	2.6	53	1.5	2.2	866	-	-	0.3	-	-	1294	(Longvah et al., 2017)
**Catfish**	*Mystus vittatus*	*Tengra/Tonger*	144	19.2	6.4	270	2	-	-	18	-	-	-	-	-	(National Institute of Nutrition et al., 1978)
**Shark**	*Carcharhinu ssorrah*	*Khari Machli*	95	21.6	0.8	8.4	0.4	0.5	1	-	-	-	2.8	110	857	(Longvah et al., 2017)
**Silhan**	*Silonia silondia*	*Silong*	156	22.7	6.7	28.8	0.6	0.6	3	-	-	0.1	-	-	2438	(Longvah et al., 2017)
**Bele fish**	*Glossogoboius giuris*	*Bale Machli*	75	14.5	0.6	370	1.0	-	-	3	-	-	-	-	-	(National Institute of Nutrition et al., 1978)

Common name	Botanical name	Vernacular name	Energy (kcal /100g)	Protein (g/100g)	Fat (g/ 100g)	Ca (mg /100g)	Iron (mg/ 100g)	Zinc (mg/ 100g)	Vit A (µg/ 100g)	Vit C (mg /100g)	Vit B1 (mg/ 100g)	Vit B2 (mg/ 100g)	Vit B3 (mg/ 100g)	Vit B6 (mg/ 100g)	Vit B9 (µg/ 100g)	Reference
**Guntea loach**	*Lepidocephalichthys guntea*	*Ngakijou*	-	-	-	2150	13.6	3.1	-	-	-	-	-	-	-	(Shantosh and Sarojnalini, 2018)
	Pangio pangia	*Nganap*	-	-	-	905	20.5	1.5	-	-	-	-	-	-	-	(Shantosh and Sarojnalini, 2018)
	Syncrossus berdmorei	*Sareng-Khoibi*	-	-	-	680.3	28.6	1.4	-	-	-	-	-	-	-	(Shantosh and Sarojnalini, 2018)
**Fowl/Rooster**	*Galloanserae*	*Jangli Murgi*	109	25.9	0.6	-	-	-	-	-	-	0.1	-	-	7	(Longvah et al., 2017)
**Quail**	*Coturnix coturnix*	*Bater*	138	20.9	6.0	20.6	1.9	1.1	13	-	0.1	0.2	-	-	9	(Longvah et al., 2017)
**Pig**	*Sus scrofa*	*Jangli suar//kissu*	179	19.4	11.3	8.1	1.0	1.3	2	-	0.3	0.1	-	-	8	(Longvah et al., 2017)
**Grasshopper**	*Chondacris rosea*	*Mirbo/'Takam/Kamrak*	373	68.8	7.8	340	7.8	10.8	-	-	-	-	-	-	-	(Chakravorty et al., 2014)
	Oedaleus abruptus (Thunberg)	-	587	60			0.1	0.2	4640	6.3	0.5	1	6	-	-	(Ganguly et al., 2013)
**Mole cricket**	*Brachytrupes orientalis*	*Takam hilak/'Takam pario tokcho'/'Komdruk*	381	65.7	6.3	76.3	18.6	8.5	-	-	-	-	-	-	-	(Chakravorty et al., 2014)
**Stink Bug**	*Aspongopus nepalensis* Westwood 1837	*Gandhipuk/tari/Gondhi bug*	-	10.6	38.4	120	20	7	34	-	-	-	-	-	-	(Chakravorty et al., 2011)
**Mulberry silkworm**	*Bombyx mori*		236	11.9	20.1	-	-	-	-	-	-	-	-	-	-	(Mishra et al., 2003)
**Non-mulberry silkworm**	*Attacus ricinii*	*Eri*	169	15.9	11.1	-	-	-	-	-	-	-	-	-	-	(Mishra et al., 2003)
	Antherae assama	*Muga*	216	14.4	16.9	-			-	-	-	-	-	-	-	(Mishra et al., 2003)
**Giant water bug**	*Lethocerus indicus* (Lepeletier and Serville)		632	22.6	13.8	96	410	29.5	-	-	-	-	-	-	-	(Shantibala et al., 2014)
**Water scorpion**	*Laccotrephes maculatus* (F.)		585	25.1	6.9	24.3	461	11.8	-	-	-	-	-	-	-	(Shantibala et al., 2014)

Common name	Botanical name	Vernacular name	Energy (kcal /100g)	Protein (g/100g)	Fat (g/ 100g)	Ca (mg /100g)	Iron (mg/ 100g)	Zinc (mg/ 100g)	Vit A (µg/ 100g)	Vit C (mg /100g)	Vit B1 (mg/ 100g)	Vit B2 (mg/ 100g)	Vit B3 (mg/ 100g)	Vit B6 (mg/ 100g)	Vit B9 (µg/ 100g)	Reference
**Water scavenger beetles**	*Hydrophilus olivaceous* (F.)		555	22.6	21.6	27.7	7.3	5.8	-	-	-	-	-	-	-	(Shantibala et al., 2014)
	Cybister tripunctatus (Olivier)		560	41.6	5.2	66.5	25	23.2	-	-	-	-	-	-	-	(Shantibala et al., 2014)
**Scarlet skimmer**	*Crocothemis servilia* (Drury)		497	70.5	4.9	86.5	11.3	9.3	-	-	-	-	-	-	-	(Shantibala et al., 2014)

NA-Not available

Names in italics are local names
