# Supplementary material for: Indigenous Foods of India: A Comprehensive Narrative Review of Nutritive Values, Antinutrient Content and Mineral Bioavailability of Traditional Foods Consumed by Indigenous Communities of India
Source: Front Sustain Food Syst. Author manuscript; Available in PMC 2022 May 22. (PMC7612755; doi:10.3389/fsufs.2022.696228)
Supplement: Table 3 [file EMS145028-supplement-Table_3.pdf]

Supplementary table 3: Anti-nutrient content in Indigenous foods of India

Common name	Botanical name	Total free phenols (mg/100g)	Tannins (mg/100g)	Hydrogen cyanide (mg/100g)	Amylase inhibitor activity (AIU 100g)	Total oxalate (mg/100g)	Phytate (mg/100g)	Saponins (mg/100g)	Trypsin inhibitor TIU/mg Protein	Reference
**Cereals**										
1. White Rice	*Oryza sativa* L					1.92	266			(Longvah et al., 2017)
2. Bajra	*Pennisetum typhoideum* Rich.					53.1	485			(Longvah et al., 2017)
3. Sorghum	*Sorghum vulgare* Pers.					28.3	549			(Longvah et al., 2017)
4. Maize,dry	*Zea mays* L.					15.3	646			(Longvah et al., 2017)
5. Ragi	*Eleusine coracana* (L.) Gaertn					39.5	306			(Longvah et al., 2017)
6. Little millet	*Panicum antidotale* Retz.					6.7	265			(Longvah et al., 2017)
7. Kodo millet	*Paspalum scrobiculatum* L.					3.5	452			(Longvah et al., 2017)
**Pulses**										
8. Cowpea, brown	*Vigna catjang* (L.) Walp.					14.3	550	299		(Longvah et al., 2017)
9. Cowpea, white	*Dolichos catjang* Burm.f					17.2	573	298		(Longvah et al., 2017)
10. Field bean, black	*Dolichos lablab* L.					1.28	759	163		(Longvah et al., 2017)
11. Field bean, brown	*Dolichos lablab* L.					1.26	799	171		(Longvah et al., 2017)
12. Field bean, white	*Dolichos lablab* L.					1.23	791	175		(Longvah et al., 2017)
13. Horse gram, whole	*Dolichos biflorus* L					181	339	230		(Longvah et al., 2017)
14. Lentil dal	*Lens culinaris* Medik.					10.5	218	163		(Longvah et al., 2017)
15. Rajmah, brown	*Phaseolus vulgaris* L.					43.4	481	285		(Longvah et al., 2017)
16. Black gram, dal	*Phaseolus mungo* L					43.7	579	120		(Longvah et al., 2017)
17. Red gram, dal	*Cajanus cajan* (L.) Millsp.					1.4	277	186		(Longvah et al., 2017)
18. Velvet bean	*Mucuna pruriens* (L.) DC.	5960	50						62.3	(Longvah et al., 2017)
19. Jack bean	*Canavalia ensiformis* (L.) DC.	1230	160						34.3	(Vadivel and Janardhanan, 2005)
20. Sword bean	*Canavalia gladiata* (Jacq.) DC.	1940	200						26.8	(Vadivel and Janardhanan, 2005)
21.	*Cassia floribunda* Collad.	410	420						16.8	(Vadivel and Janardhanan, 2005)
22. Pot Casia Beans	*Senna obtusifolia* (L.) H.S.Irwin & Barneby	660	600						13.5	(Vadivel and Janardhanan, 2005)
23.	*Mucuna pruriens* (L.) DC.	2290	550						65.4	(Vadivel and Janardhanan, 2005)

Common name	Botanical name	Total free phenols (mg/100g)	Tannins (mg/100g)	Hydrogen cyanide (mg/100g)	Amylase inhibitor activity (AIU 100g)	Total oxalate (mg/100g)	Phytate (mg/100g)	Saponins (mg/100g)	Trypsin inhibitor TIU/mg Protein	Reference
24.	*Mucuna pruriens* var. *utilis* (Wall. ex Wight) L.H.Bailey	4480	40						48.2	(Vadivel and Janardhanan, 2005)
**Green leafy vegetables**										
25. Colocasia leaves	*Colocasia esculenta* (L.) Schott					701	14.4	390		(Longvah et al., 2017)
						530				(Pradeepkumar et al., 2015)
						30	1			(National Institute of Nutrition (India) et al., 1978)
26. Drumstick leaves	*Moringa oleifera* Lam.					120	128	500		(Longvah et al., 2017)
						16	18	113		(Singh et al., 2018)
27. Amaranth leaves, red	*Amaranthus gangeticus* L.					823	4.9			(Longvah et al., 2017)
28. Amaranth spinosus, leaves, green	*Amaranthus spinosus* L.					1073				(Longvah et al., 2017)
29. Agathi leaves	*Sesbania grandiflora* (L.) Pers.					179	63.3	600		(Longvah et al., 2017)
						34	14	880		(Singh et al., 2018)
30. Ponnaganni	*Alternanthera sessilis* (L.) R.Br. ex DC.					465	32.02	880		(Longvah et al., 2017)
						213				(Pradeepkumar et al., 2015)
31. Tamarind leaves , tender	*Tamarindus indica* L.					150	33.8			(Longvah et al., 2017)
32. Gogu leaves, red	*Hibiscus sabdariffa* L.					187	43.2			(Longvah et al., 2017)
						29	26	370		(Singh et al., 2018)
33. Pumpkin leaves	*Cucurbita maxima* L.					13.6	38.3			(Longvah et al., 2017)
			157			200	9.23			(Gupta et al., 2005, 200)
34. Curry leaves	*Murraya koenigii* (L.) Spreng.					154	40.9	230		(Longvah et al., 2017)
						23	12	127		(Singh et al., 2018)
35. Basella leaves	*Basella alba* L.					170	49.5	60		(Longvah et al., 2017)
						40	19	184		(Singh et al., 2018)
36. Prickly Chaff Flower	*Achyranthes aspera* L.					180				(Pradeepkumar et al., 2015)
37. Pot Casia leaves	*Senna obtusifolia* (L.) H.S.Irwin & Barneby					598				(Pradeepkumar et al., 2015)
38. Beng leaves	*Centella asiatica (L.)* Urb.					408				(Pradeepkumar et al., 2015)
			132			60	2.13			(Gupta et al., 2005, 200)
						26	17	130		(Singh et al., 2018)
39. Vegetable fern	*Diplazium esculentum* (Retz.) Sw.					217				(Pradeepkumar et al., 2015)

Common name	Botanical name	Total free phenols (mg/100g)	Tannins (mg/100g)	Hydrogen cyanide (mg/100g)	Amylase inhibitor activity (AIU 100g)	Total oxalate (mg/100g)	Phytate (mg/100g)	Saponins (mg/100g)	Trypsin inhibitor TIU/mg Protein	Reference
40. Common Leucas	*Leucas aspera* (Willd.) Link.					222				(Pradeepkumar et al., 2015)
41. Kaattupaaval	*Momordica sahyadrica* Kattuk. and V.T.Antony					224				(Pradeepkumar et al., 2015)
42. Purslane	*Portulaca oleracea* L.					37				(Pradeepkumar et al., 2015)
						29	9.3	80		(Singh et al., 2018)
43. Black night shade	*Solanum nigrum* L.					637				(Pradeepkumar et al., 2015)
44. Chinese Spinach	*Amaranthus tricolor* L					772				(Pradeepkumar et al., 2015)
			106			1270	1.95			(Gupta et al., 2005, 200)
						40	18	169		(Singh et al., 2018)
45. Malabar spinach	*Basella rubra* L.					29	287			(Singh et al., 2018)
46. Water spinach	*Ipomoea aquatica* Forssk. [€]					38	27	650		(Singh et al., 2018)
47. Alligator weed	*Alternanthera philoxeroides* (M art.) Griseb.					48	19	191		(Singh et al., 2018)
48. Purple amaranth	*Amaranthus lividus* L.					37	14	153		(Singh et al., 2018)
49. Slender amaranth	*Amaranthus viridis* L.					32	22	319		(Singh et al., 2018)
						772	2			(National Institute of Nutrition (India) et al., 1978)
50. White jute	*Corchorus capsularis* L.					31	26	160		(Singh et al., 2018)
51. Male fern	*Dryopteris filix-mas* (L.) Schott.					41	16	250		(Singh et al., 2018)
52. Helencho	*Enhydra fluctuans* Lour.					26	31	550		(Singh et al., 2018)
53. Wild coriander	*Eryngium foetidum* L.					43	41	600		(Singh et al., 2018)
54. Kulekhara	*Hygrophila auriculata* (Schuma ch.) Heine					32.2	49.5	280		(Singh et al., 2018)
55. Water primrose	*Jussiaea repens* L.					20	17	690		(Singh et al., 2018)
56. Wild Betel	*Piper sarmentosum* Roxb.					15	56	510		(Singh et al., 2018)
57. Star Gooseberry	*Sauropus androgynus* (L.) Merr.					28	34	230		(Singh et al., 2018)
58. Black pigweed leaves	*Trianthema portulacastrum* L.		61			1080	2.02			(Gupta et al., 2005, 200)
59. Garkha leaves	*Celosia argentea* L.		113			920	2.95			(Gupta et al., 2005, 200)
60. Balae leaves	*Polygala erioptera* DC.		98			60	3.38			(Gupta et al., 2005, 200)
61. Gadhakand leaves	*Boerhavia diffusa* L.		94			1250	4.08			(Gupta et al., 2005, 200)
62. Mexican mint	*Plectranthus amboinicus* (Lour.) Spreng.		15			50	0.92			(Gupta et al., 2005, 200)
63. Lesua	*Digera muricata* (L.) Mart.		79			1410	2.49			(Gupta et al., 2005, 200)

Common name	Botanical name	Total free phenols (mg/100g)	Tannins (mg/100g)	Hydrogen cyanide (mg/100g)	Amylase inhibitor activity (AIU 100g)	Total oxalate (mg/100g)	Phytate (mg/100g)	Saponins (mg/100g)	Trypsin inhibitor TIU/mg Protein	Reference
64. Javanada leaves	*Cocculus hirsutus* (L.) W.Theob.		205			230	4.4			(Gupta et al., 2005, 200)
65. Kena leaves	*Commelina benghalensis* L.		105			390	2.4			(Gupta et al., 2005, 200)
66. Gandhuli	*Cleome gynandra* L.		136			20	13.06			(Gupta et al., 2005, 200)
67. Yellow Gulmohar leaves	*Delonix elata* (L.) Gamble		1330			90	5.11			(Gupta et al., 2005, 200)
68. Jarem/ Phuinam	*Clerodendrum glandulosum* Lin dl.	3098								(Seal, 2011)
69. Jatira	*Corydalis sibirica* (L.f.) Pers.	4059								(Seal, 2011)
70. Jaiur	*Zanthoxylum acanthopodium* D C.	6119								(Seal, 2011)
71. Perennial sow thistle	*Sonchus arvensis* L.	1732								(Seal, 2011)
**Other vegetables**										
72. Ash gourd	*Benincasa hispida* (Thunb.) Cogn.					4.89	14.6			(Longvah et al., 2017)
73. Bamboo shoot, tender	*Bambusa vulgaris Schrad. ex J.C. Wendl.*					85.7	19.8	50		(Longvah et al., 2017)
74. Bittergourd	*Momordica charantia* L..					48.5	14.3			(Longvah et al., 2017)
75. Kovai,big	*Coccinia grandis* (L.) Voigt					7.72	9.8			(Longvah et al., 2017)
						17	29	200		(Singh et al., 2018)
76. Ridge gourd	*Luffa acutangula* (L.) Roxb.					29.55	14.9			(Longvah et al., 2017)
77. Ridge gourd, smooth skin	*Luffa acutangula* (L.) Roxb.					35.85	14.9			(Longvah et al., 2017)
78. Spine gourd	Momordica diocia Roxb ex Willd	380	320	0.11	40.7	210			2.66	(Mohan and Kalidass, 2010)
79.	*Caralluma adscendens* var. *atte nuata* (Wight) Grav. & Mayur	550	250	0.13	2.3	130			1.26	(Mohan and Kalidass, 2010)
80.	*Caralluma pauciflora* (Wight) N.E.Br.	240	90	0.13	11.4	90			1.34	(Mohan and Kalidass, 2010)
81. Indian shot	Canna indica L.	1470	1830	0.03	16.9	940			1.47	(Mohan and Kalidass, 2010)
82. Plantain, flower	*Musa × paradisiaca* L.					43	8.9	237		(Singh et al., 2018)
						169	2.52			(Longvah et al., 2017)
83. Jackfruit	*Artocarpus heterophyllus* Lam.					41	24	234		(Singh et al., 2018)
						9.6	68.8			(Longvah et al., 2017)

Common name	Botanical name	Total free phenols (mg/100g)	Tannins (mg/100g)	Hydrogen cyanide (mg/100g)	Amylase inhibitor activity (AIU 100g)	Total oxalate (mg/100g)	Phytate (mg/100g)	Saponins (mg/100g)	Trypsin inhibitor TIU/mg Protein	Reference
84. Breadfruit	*Artocarpus altilis* (Parkinson ex F.A.Zorn) Fosberg					36	22	198		(Singh et al., 2018)
**Roots & Tubers**										
85. Shatavri	*Asparagus racemosus* Willd.	280	310	0.13						(Arinathan et al., 2009)
86. Kali Musli	*Curculigo orchioides* Gaertn.	390	80	0.33						(Arinathan et al., 2009)
87. Potato Yam	*Dioscorea bulbifera* L.	3370	2550	0.17	400					(Arinathan et al., 2009)
		220	1480	0.19	1.37	780			1.21	(Shajeela et al., 2011)
		900	70		17.3			200	3.6	(Padhan et al., 2020)
88. Indian yam	*Dioscorea oppositifolia* L.	240	90	0.09	80					(Arinathan et al., 2009)
		340	20	0.3						(Arinathan et al., 2009)
		280	100	0.14	41.3	380			2.1	(Mohan and Kalidass, 2010)
		560	360	0.33	21	460			11.26	(Arinathan et al., 2009)
		360	240	0.24	24.6	360			13.3	(Arinathan et al., 2009)
		350	40		18.1			170	3.4	(Padhan et al., 2020)
89. Five leaf yam	*Dioscorea pentaphylla* L.	310	60	0.17	240					(Arinathan et al., 2009)
		750	440	0.09	19.5	310			2.86	(Mohan and Kalidass, 2010)
		480	90	0.18	24.6	580			3.66	(Arinathan et al., 2009)
		560	60	15.1			170		3.1	(Padhan et al., 2020)
90. Nurai	*Dioscorea tomentosa* J.Koenig ex Spreng.	40	200	0.1						(Arinathan et al., 2009)
		230	70	0.05	15.4	30			0.87	(Mohan and Kalidass, 2010)
		410	60	0.34	46.4	310			1.41	(Shajeela et al., 2011)
91. Wild butter bean	*Dolichos trilobus* L.	80	120	0.32						(Arinathan et al., 2009)
92. Indian three-leaved yam	*Dioscorea hispida* Dennst.	430	60	14.6				110	5.3	(Padhan et al., 2020)
					80					(Rajyalakshmi and Geervani, 1994)
93. Colocasia	*Colocasia esculenta* (L.) Schott					48.73	13.57			(Longvah et al., 2017)
		140	20	0.03	40.4	610			2.44	(Mohan and Kalidass, 2010)
						40	24	269		(Singh et al., 2018)
94. Tapioca	*Manihot esculenta* Crantz.					16.86	64.42			(Longvah et al., 2017)
95. Floating lace plant	*Aponogeton natans* (L.) Engl. & K.Krause	240	10	0.04	92.3	230			11.34	(Mohan and Kalidass, 2010)
96. Boerhavia chinensis	*Boerhavia chinensis* (L.) Rottb.	100	20	0.03	21.4	70			0.58	(Mohan and Kalidass, 2010)
97. Hadjod/Veldt grape	Cissus quadrangularis L.	80	140	0.15	13.5	40			2.92	(Mohan and Kalidass, 2010)
98. Kattumunthiri	Cissus vitiginea L.	120	170	0.09	20.4	100			0.33	(Mohan and Kalidass, 2010)

Common name	Botanical name	Total free phenols (mg/100g)	Tannins (mg/100g)	Hydrogen cyanide (mg/100g)	Amylase inhibitor activity (AIU 100g)	Total oxalate (mg/100g)	Phytate (mg/100g)	Saponins (mg/100g)	Trypsin inhibitor TIU/mg Protein	Reference
99. Queen sago	*Cycas circinalis* L.	200	20	0.01	23.6	780			1.7	(Mohan and Kalidass, 2010)
100. Swallow root	*Decalepis hamiltonii* Wight & Arn.	270	20	0.04	15.4	280			1.26	(Mohan and Kalidass, 2010)
		320	30		13.5			160	3.5	(Padhan et al., 2020)
101. Athikizhangu	Dioscorea spicata Roth	380	340	0.09	33.4	330			1.39	(Mohan and Kalidass, 2010)
		260	10	0.18	33.1	440			1.26	(Shajeela et al., 2011)
102. Mou Alu	*Dioscorea wallichii* Hook.f.	440	50		19.4			160	2.7	(Padhan et al., 2020)
		330	40	0.16	52.7	260			2.48	(Arinathan et al., 2009)
103. Anantmul	*Hemidesmus indicus* (L.) R. Br. ex Schult.	40	20	0.05	23.6	180			1.04	(Mohan and Kalidass, 2010)
104. Ipomoea sumatrana	*Ipomoea sumatrana* (Miq.) Ooststr.	100	120	0.04	75.4	980			2.36	(Mohan and Kalidass, 2010)
105. Kedrostis foetidissima	*Kedrostis foetidissima* (Jacq.) Cogn.	120	20	0.05	23.6	80			1.37	(Mohan and Kalidass, 2010)
106. Maerua oblongifolia	*Maerua oblongifolia* (Forssk.) A.Rich.	100	20	0.09	11.7	260			0.38	(Mohan and Kalidass, 2010)
107. Koka	*Nymphaea pubescens* Willd.	210	180	0.12	38.6	380			2.41	(Mohan and Kalidass, 2010)
108. Water lily	Nymphaea rubra Roxb. ex Andrews	200	110	0.04	10.1	420			0.56	(Mohan and Kalidass, 2010)
109.	*Parthenocissus neilgherriensis* Planch.	480	30	0.06	29.4	2750			0.98	(Mohan and Kalidass, 2010)
110. Purple Yam	*Dioscorea alata* L.	680	410	0.17	62.1	580			3.65	(Shajeela et al., 2011)
						21.3	222.5			(Singh et al., 2018)
		360	40		11.4			160	0.4	(Padhan et al., 2020)
111. Kukai sanga	*Dioscorea pubera* Blume	330	50		13			210	3.5	(Padhan et al., 2020)
112. Churka Alu	*Dioscorea glabra* Roxb.	600	60		21			150	4	(Padhan et al., 2020)
113. Fish mint	*Houttuynia cordata* Thunb	2460								(Seal, 2011)
114. Lynniang	*Potentilla lineata* Trevir.	24802								(Seal, 2011)
**Fruits**										
115. Goosberry	*Phyllanthus emblica* L.					7.96	49.3	400		(Longvah et al., 2017)
116. Wood Apple	*Aegle marmelos* (L.) Correa					55.8	101	280		(Longvah et al., 2017)
117. Indian Jujube	*Ziziphus jujuba* Mill.					2.89	85.6			(Longvah et al., 2017)
**Flesh foods**										
118. Grasshopper	*Oedaleus abruptus* (Thunberg)		245			60	8.5			(Ganguly et al., 2013)

Common name	Botanical name	Total free phenols (mg/100g)	Tannins (mg/100g)	Hydrogen cyanide (mg/100g)	Amylase inhibitor activity (AIU 100g)	Total oxalate (mg/100g)	Phytate (mg/100g)	Saponins (mg/100g)	Trypsin inhibitor TIU/mg Protein	Reference
119. Giant water bug	*Lethocerus indicus* (Lepeletier and Serville)	160	372.3							(Shantibala et al., 2014)
120. Water scorpion	*Laccotrephes maculatus* (F.)	141	350.4							(Shantibala et al., 2014)
121. Water scavenger beetles	*Hydrophilus olivaceous* (F.)	202.6	528.6							(Shantibala et al., 2014)
122. Beetle	*Cybister tripunctatus* (Olivier)	268.6	301.6							(Shantibala et al., 2014)
123. Scarlet skimmer	*Crocothemis servilia* (Drury)	18	465.3							(Shantibala et al., 2014)
